# Discovery and Characterization of Synthesized and FDA-Approved Inhibitors of Clostridial and Bacillary Collagenases

**DOI:** 10.1021/acs.jmedchem.2c00785

**Published:** 2022-09-26

**Authors:** Alaa Alhayek, Ahmed S. Abdelsamie, Esther Schönauer, Virgyl Camberlein, Evelyn Hutterer, Gernot Posselt, Jamil Serwanja, Constantin Blöchl, Christian G. Huber, Jörg Haupenthal, Hans Brandstetter, Silja Wessler, Anna K. H. Hirsch

**Affiliations:** † Helmholtz Institute for Pharmaceutical Research Saarland (HIPS), Helmholtz Center for Infection Research (HZI), Campus Building E8.1, 66123 Saarbrücken, Germany; ‡ Department of Pharmacy, Saarland University, Campus Building C2. 3, 66123 Saarbrücken, Germany; § Department of Chemistry of Natural and Microbial Products, Institute of Pharmaceutical and Drug Industries Research, National Research Centre, El-Buhouth St., Dokki, 12622 Cairo, Egypt; ∥ Department of Biosciences and Medical Biology, University of Salzburg, Hellbrunner Str. 34, 5020 Salzburg, Austria

## Abstract

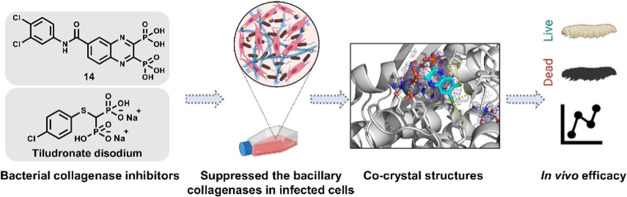

In view of the worldwide antimicrobial resistance (AMR) threat, new bacterial targets and anti-infective agents are needed. Since important roles in bacterial pathogenesis have been demonstrated for the collagenase H and G (ColH and ColG) from *Clostridium histolyticum*, collagenase Q1 and A (ColQ1 and ColA) from *Bacillus cereus* represent attractive antivirulence targets. Furthermore, repurposing FDA-approved drugs may assist to tackle the AMR crisis and was addressed in this work. Here, we report on the discovery of two potent and chemically stable bacterial collagenase inhibitors: synthesized and FDA-approved diphosphonates and hydroxamates. Both classes showed high *in vitro* activity against the clostridial and bacillary collagenases. The potent diphosphonates reduced *B. cereus*-mediated detachment and death of cells and *Galleria mellonella* larvae. The hydroxamates were also tested in a similar manner; they did not have an effect in infection models. This might be due to their fast binding kinetics to bacterial collagenases.

## Introduction

Bacterial resistance is on the rise, and a global economic and public health crisis might be on the horizon due to the high incidence of deaths caused by multidrug-resistant bacteria.^1,2^ This also comes together with the slow discovery of new antibiotics. If this trend continues, mild infections of today might become lethal in the future.^1,2^ To combat the rise in antibiotic resistance, alternative, nonantibiotic treatment approaches are urgently needed. Antivirulence agents (also called “pathoblockers”), which selectively inhibit pathogenicity factors of bacteria, and hence prevent or delay infection, are one potential strategy.^3,4^ This could—without exerting selective pressure—harness the host’s immune system to fight the infection.^3,4^ Finding new applications for existing FDA-approved drugs is gaining popularity to tackle the current antimicrobial-resistance crisis as it reduces the cost, time, and effort required for the regular discovery of new antibiotics.^5^ Pentamidine is an antiprotozoal medicine and an example of an FDA-approved drug that has been repurposed for antibacterial use.^6^ This approach holds promise as it may overcome the slow development of new antibiotics.

*Clostridium histolyticum* (*C. histolyticum*) and *Bacillus cereus* (*B. cereus*) are Gram-positive bacteria and the epitome of many serious opportunistic infections, including gas gangrene as well as wound, corneal, and gastro-intestinal infections.^7,8^ As these bacteria resist the activity of many antibiotics (such as penicillin), they are still susceptible to the activity of gentamycin, vancomycin, erythromycin, and other antibiotics.^9,10^ The pathogenicity of these microorganisms is linked to their secreted toxins and proteases, which assist them to elude defensive mechanisms, reach deep locations for nourishment, and consequently replicate and persist at the infection site.^11^ This might also enhance bacterial histotoxicity by promoting toxin diffusion.^11^ Bacterial collagenases are calcium- and zinc-dependent metalloproteases and the etiologic feature of the aforementioned pathogens; they destroy tissues by demolishing extracellular matrix (ECM) collagen.^12,13^ Fibrillar collagen is the most common protein in the ECM (up to 90%). It has a stable structure that resists proteolysis and can only be broken down by true bacterial and mammalian collagenases.^12,13^ Bacterial collagenases process triple helical collagen under physiological conditions into small peptides and amino acids by targeting multiple sites.^12,13^ Mammalian collagenases, on the other hand, such as the collagenolytic matrix metalloproteinases (MMPs), cleave collagen at a single site to generate the characteristic 3/4 and 1/4 fragments. After this initial cut, other enzymes assist in the further proteolysis of the collagen fragments.^13^ Collagen is involved in many vital physiological processes such as tissue regeneration and wound healing, in addition to its tissue-supporting functions.^13^ Therefore, any bacterial collagenase-induced imbalance in collagen structure or quantity will have detrimental effects on tissue regeneration and wound healing besides the formation of voids in the ECM, which allow the bacteria to invade and gain access to anaerobic areas.^14^ Consequently, protecting collagen from bacterial collagenase represents a promising approach for developing antivirulence agents that can be used to treat collagenase-positive infections.

In contrast to the homologous *B. cereus* collagenases, *C. histolyticum* collagenases have been well studied. They are composed of two main structural modules: the collagenase unit and collagen recruitment domains. The collagenase unit is composed of an activator and peptidase domains.^15,16^ In the latter, two histidine residues in a HEXXH motif and a downstream glutamate coordinate the catalytic zinc ion. The general-acid base glutamate and the zinc ion polarize the water molecule in the active site and activate it for the nucleophilic attack.^15−18^ The collagen recruitment domains are suggested to be involved in collagen swelling and binding to fibrillar and insoluble collagen.^15−18^ High-resolution crystal structures are available for *C. histolyticum* collagenases but not for *B. cereus* collagenases, which share a high sequence similarity (*i.e.*, 70%).^17,19−21^

We focused in this study on *C. histolyticum* collagenases H and G (ColH and ColG), as well as *B. cereus* collagenases Q1 and A (ColQ1 and ColA). These bacterial collagenases are attractive candidates for the development of antivirulence drugs due to their role in bacterial pathogenicity as well as their extracellular localization.^14^ The penetration of the bacterial cell wall is generally a key challenge for the development of antibacterial agents; however, in this instance, it can be avoided.

**Figure 1:**
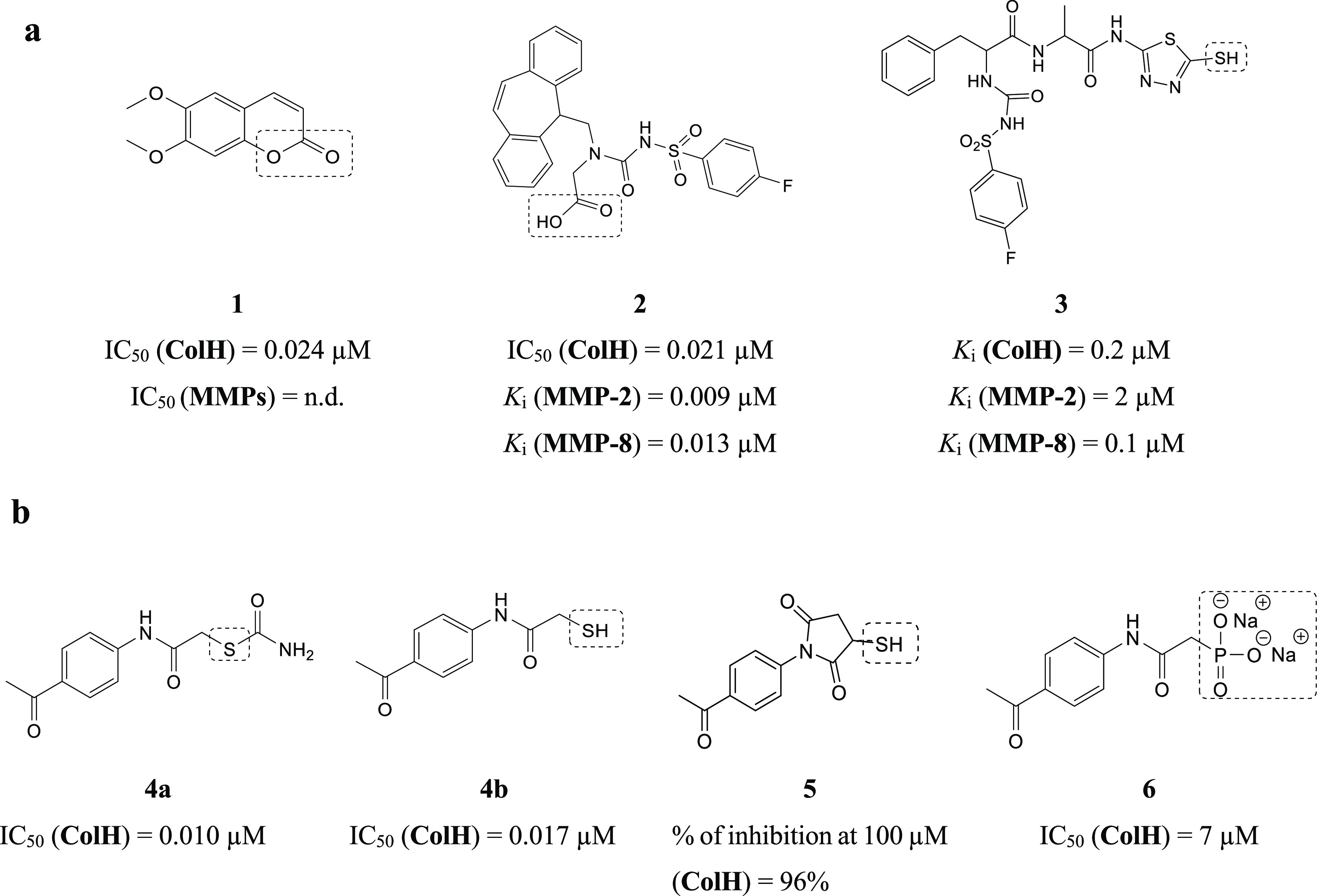
Examples of known bacterial collagenase H and Q1 (ColH and ColQ1) inhibitors. (a) Structures of bacterial ColH inhibitors.^22−24^ (b) Structures of the recently identified inhibitors of ColH and ColQ1.^25−28^ Zinc-binding groups are highlighted by dashed rectangles. n.d.: not determined.

Distinct collagenase inhibitors have been identified to date, the majority of which include a zinc-binding group (ZBG) that binds to the catalytic zinc ion, displacing the catalytic water molecule from the coordination sphere and rendering the enzyme inactive. Figure 1a shows examples of known ColH inhibitors.^22−24^ Their lack of selectivity over human MMPs is the primary drawback that prevents further development of these existing potent inhibitors.

Recently, we were able to develop selective inhibitors of bacterial collagenases. Compound **4a**, a thiocarbamate, serves as a prodrug. By conversion into a free thiol **4b**, it can bind to the zinc ion (Figure 1b).^25^ The rest of the molecule binds to the conserved clostridial nonprimed edge strand, explaining the selectivity for the bacterial collagenases over the human off-target MMPs.^25^ Succinimide **5** is a more rigid derivative of compound **4b** (Figure 1b).^26^ Similarly to thiocarbamates, this class has been validated for its high selectivity for several bacterial collagenases, including ColH and ColG from *C. histolyticum*, ColT from *C. tetani*, and ColQ1 from *B. cereus*, over the unwanted inhibition of human MMPs. The main drawback of these two compound classes is their chemical instability due to the oxidation of the thiol group to the corresponding disulfide, which leads to the loss of their activity.

Replacing the thiol group with another stable ZBG to maintain the chemical stability of the inhibitor is necessary, in addition to maintaining the high selectivity toward bacterial metalloproteases. The first stable and selective inhibitor (*i.e.*, compound **6**) (Figure 1b) of ColH was recently reported.^27,28^ Among various ZBGs, a phosphonate group has high stability and selectivity but moderate activities on ColH and ColQ1.^27,28^

In this work, we aimed to find chemically stable, potent, and selective bacterial collagenase inhibitors. Furthermore, we set out to characterize a range of compounds bearing several different ZBGs. We identified two chemical classes, namely, diphosphonates (including FDA-approved drugs) and hydroxamates with excellent selectivity, low cytotoxicity, and remarkable micro- to submicromolar activity *in vitro* against *B. cereus* ColQ1, ColA, and *C. histolyticum* ColH and ColG. This finding demonstrates the potential of these compounds to exert a broad-spectrum activity toward a variety of disease-causing bacteria. In addition, we show their activity in increasingly complex whole-cell assays and efficacy in a simple *Galleria mellonella* infection model. Hydroxamates were tested analogously. Surprisingly, they did not demonstrate the same inhibitory impact in the infection models, despite similar potencies in enzyme-inhibition studies.

## Results and Discussion

### Screening of Compounds with Various ZBGs on ColQ1 and ColH

**Figure 2:**
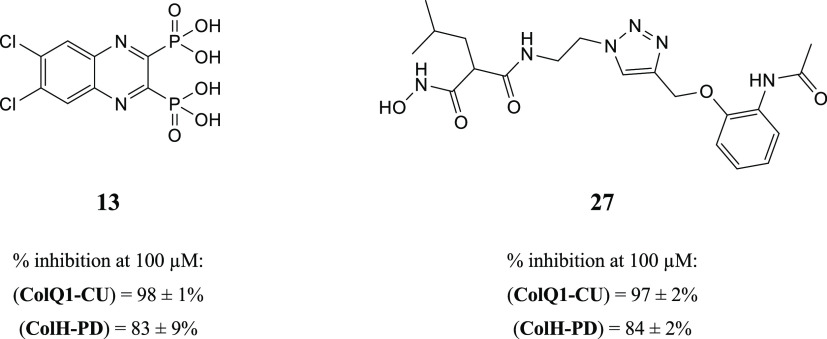
Chemical structures of compounds **13** and **27** and their activities against the collagenase unit (CU) of ColQ1 and peptidase domain (PD) of ColH.

To discover new small-molecule inhibitors with a stable ZBG, a total of 38 compounds with distinct ZBGs were tested at 100 μM in an *in vitro* peptidolytic assay using a custom-made collagenase-specific quenched fluorescent substrate (Table S1 and Figure S1). Two compounds (**13** and **27**) showed strong inhibition against the collagenase unit of ColQ1 (COlQ1-CU) (98 ± 1% inhibition and 97 ± 2% inhibition) and the peptidase unit of ColH (ColH-PD) (83 ± 9% inhibition and 84 ± 2% inhibition), respectively (Figure 2). Both compounds were selected for further studies, and a small library based on **13** and **27** structures was designed and synthesized (*i.e.*, 16 diphosphonates and 6 hydroxamates).

### Synthesis of New Anticollagenase Pathoblocker Agents

#### Diphosphonate Synthesis

**Scheme 1:**
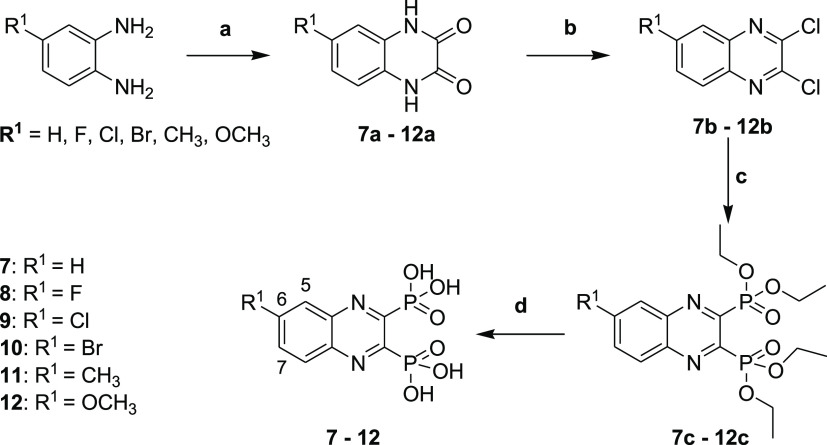
Synthesis of Compounds **7–12** Reagents and conditions: (a) oxalic acid, 4 N HCl, reflux, 6 h; (b) POCl_3_, DMF, 50 °C, 6 h; (c) triethyl phosphite, sealed tube, 150 °C, 18 h; and (d) bromotrimethylsilane, dry DCM, stirring, room temperature, 18 h.

To get access to the different diphosphonate compounds, a synthetic route composed of four steps starting from the corresponding diamine was implemented. As depicted in Scheme 1, the synthesis of the diphosphonate compounds **7–12** started by refluxing the corresponding diamine with oxalic acid in 4 N hydrochloric acid to achieve the 1,4-dihydroquinoxaline-2,3-diones **7a–12a**. These quinoxaline-dione intermediates were then reacted with POCl_3_ in DMF to provide the 2,3-dichloroquinoxalines **7b–12b**, which were used in the following step without additional purification.^29,30^ The resulting dichloroquinoxalines **7b–12b** were converted *via* an Arbuzov reaction to the corresponding diethyl phosphonate esters **7c–12** using triethyl phosphite and heated in a sealed tube at 150 °C. TMSBr was then utilized to cleave the diethyl phosphonate esters **7c–12c** to their corresponding phosphonic acids **7–12** in good yields.

**Scheme 2:**
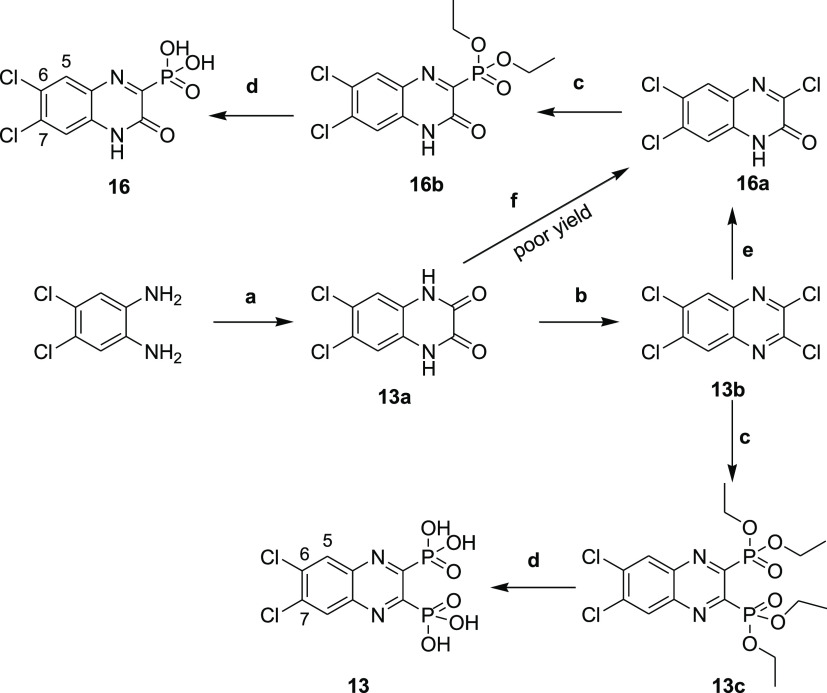
Synthesis of Compounds **13** and **16** Reagents and conditions: (a) oxalic acid, 4 N HCl, reflux, 6 h; (b) POCl_3_, DMF, 50 °C, 6 h; (c) triethyl phosphite, sealed tube, 150 °C, 18 h; (d) bromotrimethylsilane, dry DCM, stirring, room temperature, 18 h; (e) dioxane/H_2_O (1:1), LiOH, 55 °C, 24 h; and (f) POCl_3_, DMF, 0 °C to room temperature, 5 min.

Compound **13** was synthesized using the same general procedure for the synthesis of the diphosphonates (Scheme 2). To get further access to the monophosphonate compound **16**, intermediate **13b** was converted into **16a** using LiOH in a dioxane/water mixture, as described by Yang *et al.* (Scheme 2).^30^ The latter was then subjected to the Arbuzov reaction followed by ester hydrolysis using TMSBr to afford the corresponding phosphonic acid **16**.

**Scheme 3:**
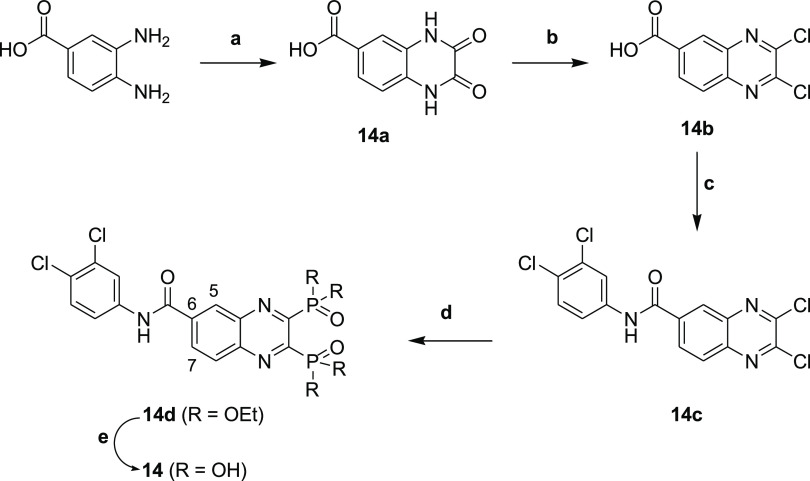
Synthesis of Compound **14** Reagents and conditions: (a) oxalic acid, 4 N HCl, reflux, 6 h; (b) POCl_3_, DMF, 50 °C, 6 h; (c) 3,4-dichloroaniline, EDC.HCl, DCM, 18 h; (d) triethyl phosphite, sealed tube, 150 °C, 18 h; and (e) bromotrimethylsilane, dry DCM, stirring, room temperature, 18 h.

Refluxing of 3,4-diaminobenzoic acid and oxalic acid in 4 N hydrochloric acid yielded **14a**, which reacted with POCl_3_ in DMF to afford the corresponding dichloro derivative **14b**. Compound **14c** was synthesized by reacting 2,3-dichloroquinoxaline-6-carboxylic acid **14b** with 3,4-dichloroaniline in DCM at room temperature for 18 h using EDC·HCl as a coupling reagent. This was followed by the Arbuzov reaction and ester hydrolysis to achieve the desired compound **14** (Scheme 3).

**Scheme 4:**
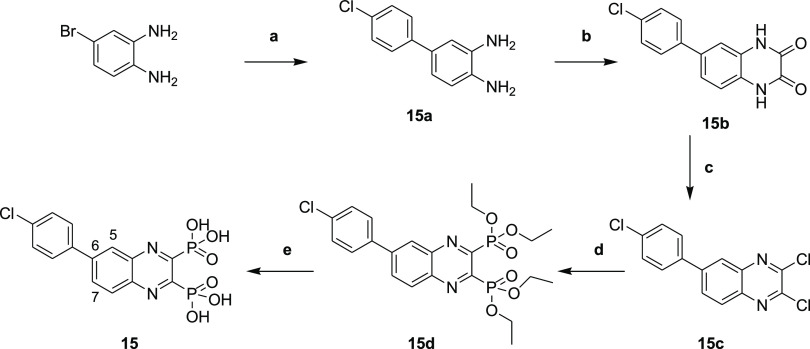
Synthesis of Compound **15** Reagents and conditions: (a) (4-chlorophenyl)boronic acid, dioxane/H_2_O (4:1), Na_2_CO_3_ (2 M), Pd(PPh_3_)_4_, microwave, 20 min; (b) oxalic acid, 4 N HCl, reflux, 6 h; (c) POCl_3_, DMF, 50 °C, 6 h; (d) triethyl phosphite, sealed tube, 150 °C, 18 h; and (e) bromotrimethylsilane, dry DCM, stirring, room temperature, 18 h.

Compound **15a** was obtained *via* a Suzuki cross-coupling reaction by reacting 4-bromobenzene-1,2-diamine and (4-chlorophenyl)boronic acid in the presence of 2 M sodium carbonate and (1,1′-bis(diphenylphosphino)ferrocene)palladium(II) dichloride [Pd(dppf)Cl_2_] as a catalyst in a mixture of dioxane/H_2_O (4:1) under microwave irradiation (150 °C, 150 W) for 20 min.^31^ This was followed by the general procedure for the diphosphonate synthesis to afford compound **15** (Scheme 4).

#### Hydroxamate Synthesis

**Scheme 5:**
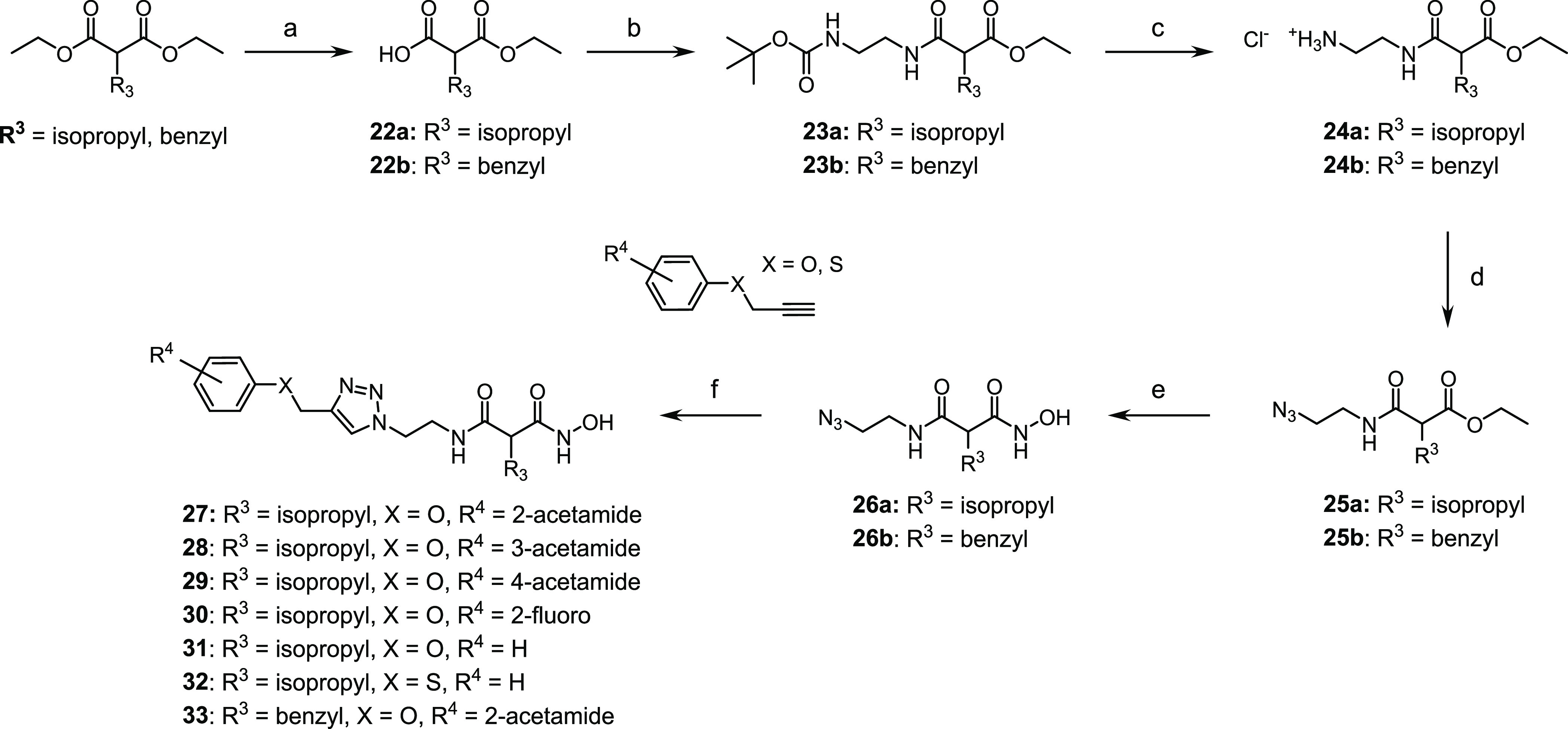
Synthesis of Hydroxamic Acid Compounds Reagents and conditions: (a) NaOH, EtOH/H_2_O (4:1), rt., 18 h; (b) tert-butyl *N*-(2-aminoethyl)carbamate, EDC.HCl, HOBt, DIPEA, CH_2_Cl_2_, rt., 18 h; (c) 4 N HCl, EtOH, 0 °C to rt., 18 h; (d) azide-*N*-diazoimidazole-1-sulfonamide hydrogen sulfate, K_2_CO_3_, ZnCl_2_, DIPEA, MeOH, rt., 18 h; (e) aq. hydroxylamine (50% in water *w*/*w*), KCN (cat.), MeOH, rt., 18 h; and (f) alkyne **34a**–**34d**, prop-2-ynoxybenzene or prop-2-ynylsulfanylbenzene, CuSO_4_ (5H_2_O), NaAsc, *N*,*N*-dimethylformamide/H_2_O (1.2:1), rt., 18 h.

As described in Scheme 5, the hydroxamates **27–33** were synthesized in six steps. The monocarboxylic acids **22a** and **22b** were first obtained through a monosaponification using sodium hydroxide in a mixture of ethanol and water. These intermediates were then activated using EDC·HCl and HOBt, in dichloromethane with diisopropylethylamine, to form the desired amides **23a** and **23b** by reacting them with the free amine. Then, an addition of hydrochloric acid (4 N in dioxane) afforded the free amines **24a** and **24b**, and these were reacted with the diazo transfer reagent to form the azides **25a** and **25b**. The ethyl esters were engaged in a KCN-catalyzed aminolysis reaction, which led to the formation of the azido hydroxamic acids **26a** and **26b**. The final step was a copper-catalyzed Huisgen 1,3-dipolar cycloaddition to give the desired 1,4-disubstituted 1,2,3-triazoles **27–33**, the needed alkynes **34a–34d** being previously synthesized by the nucleophilic substitution of phenols or thiophenol on propargyl bromide.

### Activity on *B. cereus* ColQ1

#### Structure–Activity Relationships of the Synthesized and FDA-Approved Diphosphonate Compounds on ColQ1

The initial testing of the diphosphonate **13** and its synthesized derivatives using ColQ1-CU showed that the presence of both phosphonic acid groups is indispensable for inhibition (Table 1), indicating that the phosphonate groups act as ZBG to the catalytic zinc ion. The unsubstituted diphosphonic acid quinoxaline **7** showed lower activity than the 3,4-dichloro analogue **13** (Table 1). Introducing an electron-withdrawing group such as fluorine at position 6 of the quinoxaline moiety of **8** led to no significant change in activity. Interestingly, increasing the lipophilicity by adding chlorine or bromine at position 6 of the quinoxaline of **9**, **10**, and **13** led to a significant boost in activity in comparison with the unsubstituted **7**, indicating the importance of the lipophilic groups at this part of the inhibitor (Table 1). This finding prompted us to further explore variable lipophilic moieties at this part of the molecule in an attempt to enhance the activity. Compounds **14** and **15** were synthesized and tested yielding less potent inhibitors, which could be an indication of inappropriate bulky groups at that part of the molecule. Adding an electron-donating group at position 6 of the quinoxaline moiety showed a slight improvement in the activity, as shown by the methyl derivative **11**, while the methoxy derivative **12** resulted in a decrease in activity (Table 1).

**Table 1:**
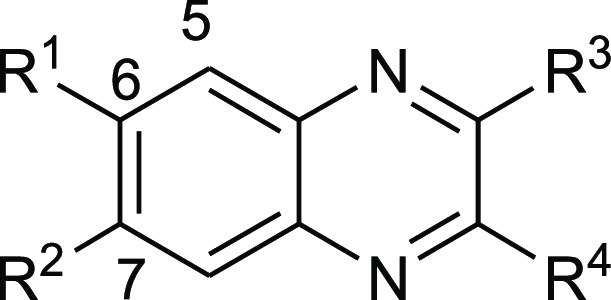

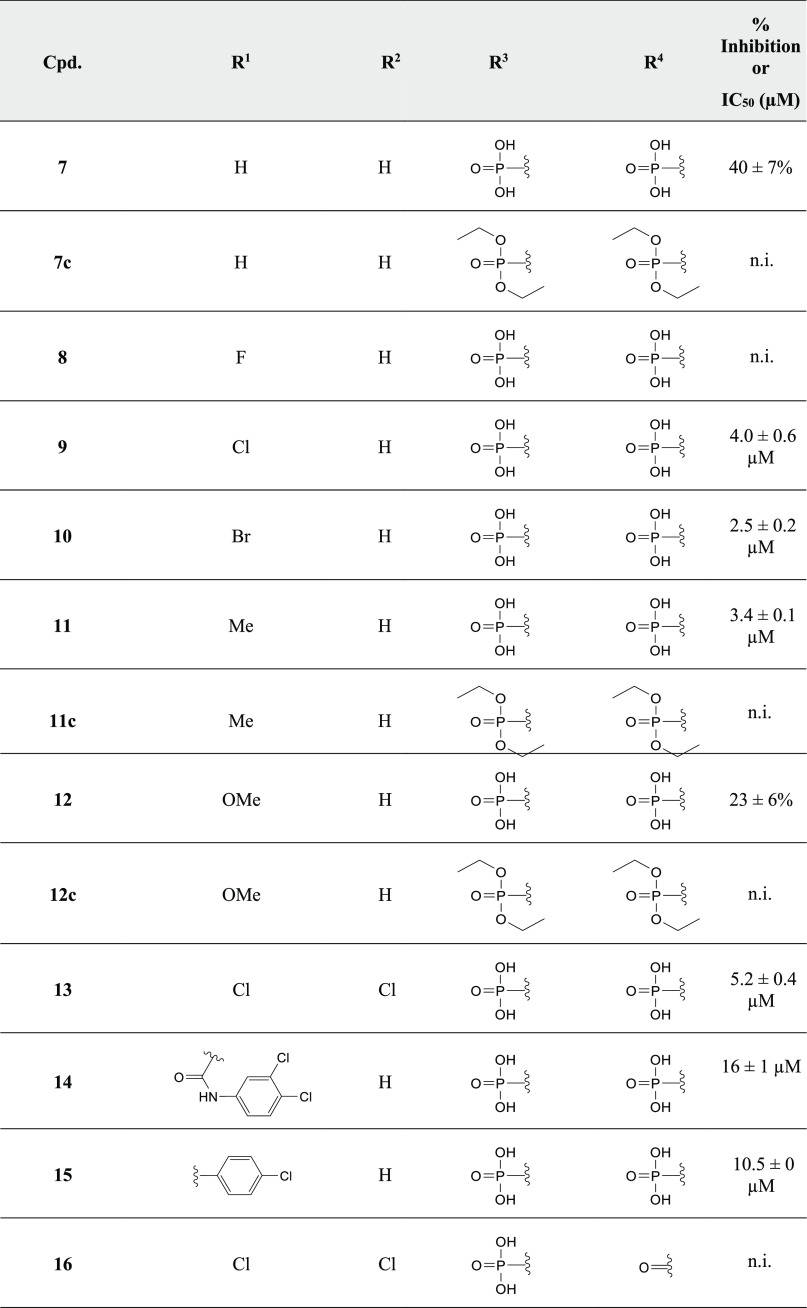
Percent Inhibition (at a Concentration of 100 μM) and IC_50_ Values of Diphosphonate Derivatives against ColQ1-CU^a^

a Means and SD of three independent experiments, n.i.: no inhibition (percent inhibition < 5%).

**Figure 3:**
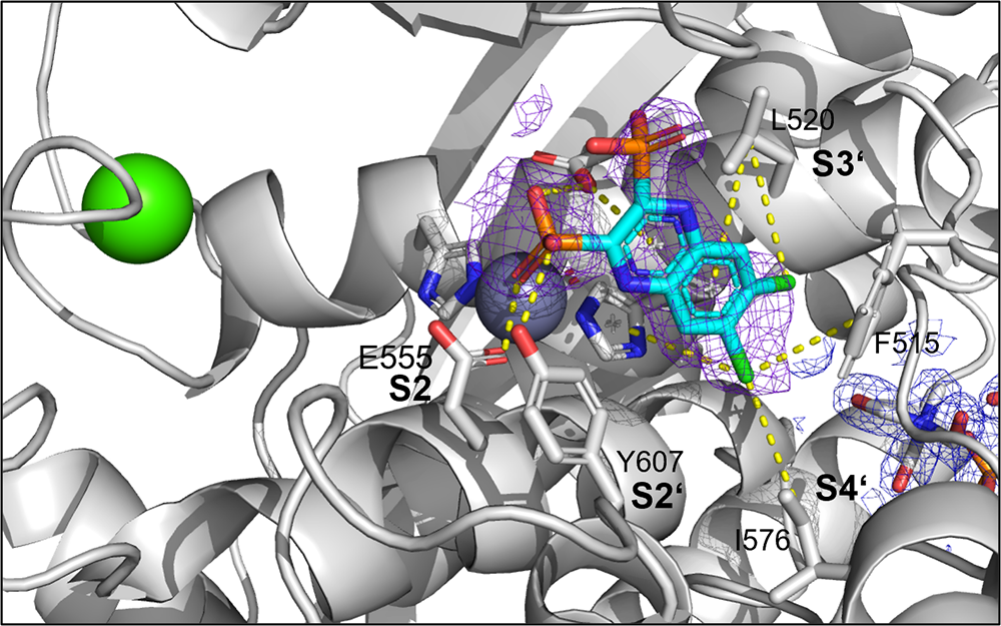
Crystal structure of ColG-PD in complex with **13** solved at a resolution of 1.95 Å. Close-up view of the active site in ball-and-stick representation. The inhibitor (cyan) is shown in sticks with a polder map contoured at 2.5 σ above the background. The catalytic zinc ion (dark gray) and the calcium ion (green) are shown as spheres (PDB code: 7ZBV).

In the case of ColG-PD (a close homologue of ColQ1), the SAR could be rationalized by a crystal structure in complex with compound **13** determined at 1.95 Å resolution (Figure 3 and Table S2). In addition to a nonfunctional binding site at the rear of the peptidase domain, we could detect **13** in the primed binding pocket, albeit at relatively low occupancy/high mobility. The inhibitor could be modeled into the active site with the help of a polder map. One of the phosphonate groups acted as ZBG and simultaneously interacted with Glu555 and Tyr607, while the aromatic scaffold of **13** and the chlorides established hydrophobic interactions in the primed binding pockets with Phe515, His523, and Ile576.

As drug repurposing is gaining attraction these days, especially for finding new antimicrobial agents,^5^ we tested a number of FDA-approved diphosphonates regarding their effect on ColQ1-CU (Table 2). **Tiludronate disodium** and **alendronate sodium** resulted in a moderate inhibition of ColQ1-CU (63 ± 3 and 76 ± 1%, respectively) at a concentration of 100 μM. Diphosphonate FDA-approved drugs are routinely used in the treatment of bone diseases and were never reported to have antibacterial activity.^32^ This calls for testing more diphosphonate FDA-approved drugs for their activity on bacterial collagenases to find potent inhibitors with known data regarding their safety, efficacy, and pharmacokinetics.

**Table 2:**
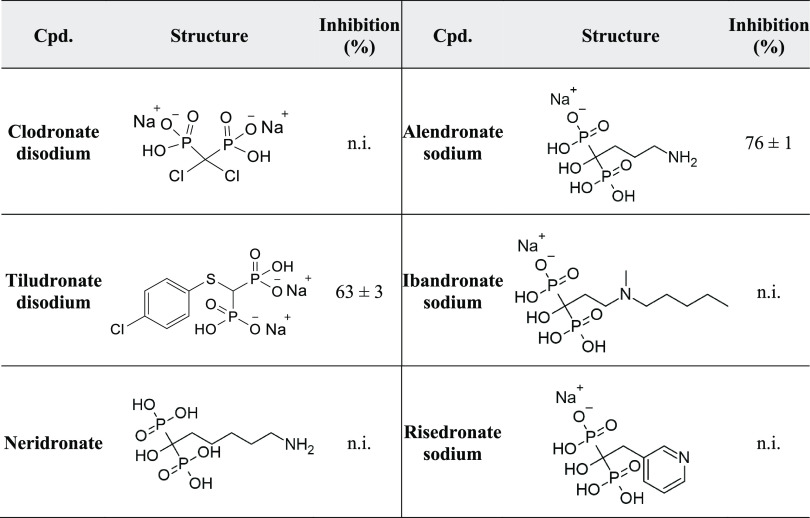
Percent Inhibition of ColQ1-CU by Selected FDA-Approved Diphosphonates at a Concentration of 100 μM^a^

a Means and SD of three independent experiments, n.i.: no inhibition (percent inhibition < 5%).

#### Structure–Activity Relationships of Hydroxamate Compounds on ColQ1

An initial screening led to the identification of hydroxamate **27**; the SAR study was performed using COLQ1-CU, which is articulated around a 1,4-disubstituted 1,2,3-triazole ring (Figure S2 and Table 3). To explore the *ortho*-acetamide phenoxy group, four derivatives were synthesized (**28–31**). The move of the *ortho*-acetamide to *meta*- and *para*-positions as in **28** and **29**, respectively, led to a slight decrease in activity, while its removal (compound **31**) or its replacement by a 2-fluorine motif (compound **30**) produced similar activities. The phenol replacement of **31** by thiophenol (compound **32**) led to a lower ColQ1-CU inhibition. Interestingly, compound **33** demonstrated that bulky substituents in the *α*-position of the hydroxamate could be tolerated.

**Table 3:**
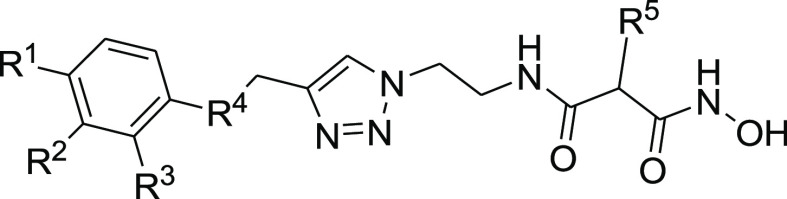

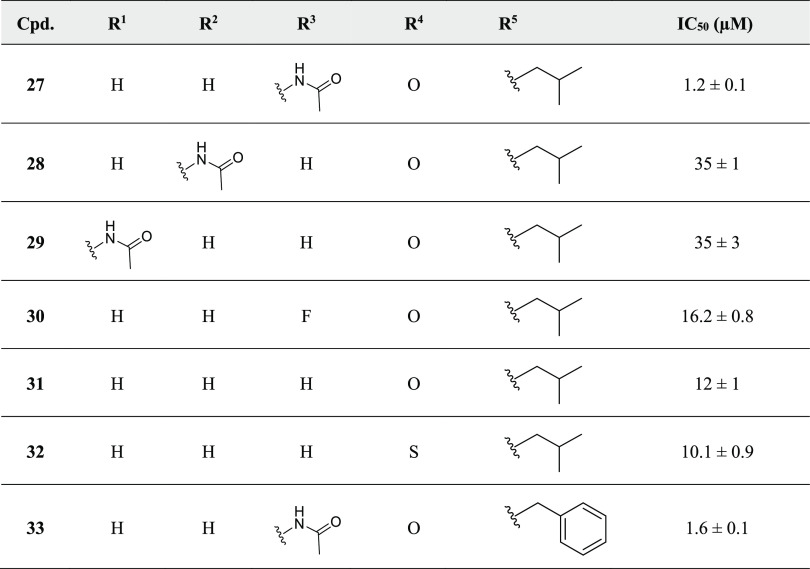
Percent Inhibition (at a Concentration of 100 μM) or IC_50_ Values of Hydroxamates against ColQ1-CU^a^

a Means and SD of three independent experiments.

Next, we evaluated the potential of the most effective compounds **27** and **33** as broad-spectrum inhibitors of bacterial collagenases and determined the inhibition constants using ColG-CU, ColH-PD, ColA-CU (from *B. cereus* strain ATCC14579), and ColQ1-CU. The results revealed that both compounds inhibited clostridial and bacillary collagenases in the low micro- to submicromolar range (Table 4).

**Table 4:** Inhibition Constant (*K_i_*) of **27** and **33** against Bacterial Collagenases^a^

		**27**	**33**
bacteria	protein	*K_i_* (μM)	*K_i_* (μM)
*C. histolyticum*	ColH-PD	11.6 ± 0.4	1.7 ± 0.2
	ColG-CU	31 ± 1	18.4 ± 0.6
*B. cereus*	ColQ1-CU	0.10 ± 0.02	0.82 ± 0.07
	ColA-CU	3.4 ± 4	4.7 ± 0.3

a Means and SD of three independent experiments.

**Figure 4:**
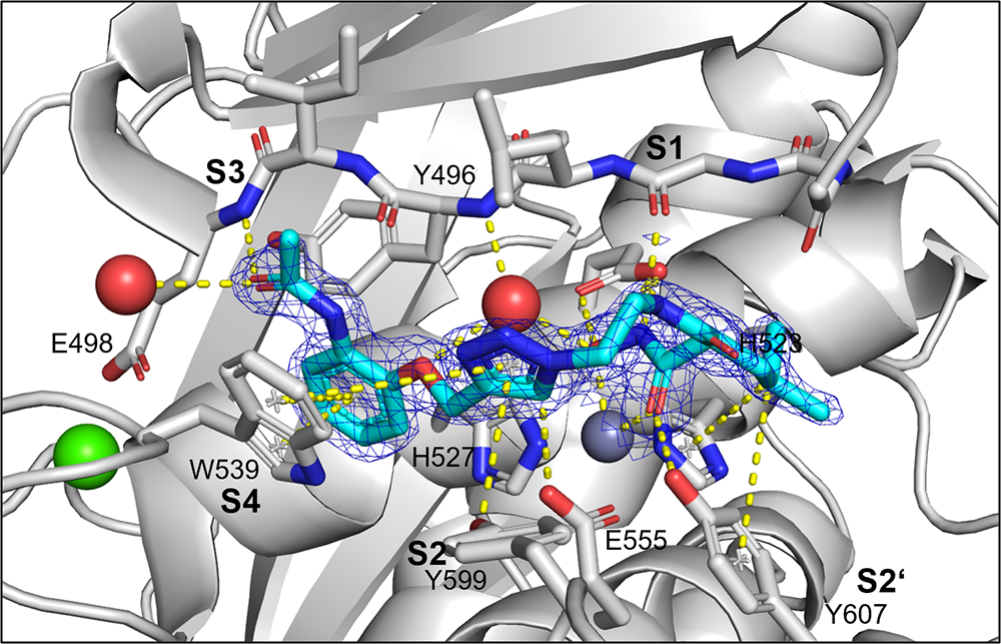
Crystal structure of ColG-PD in complex with **27** solved at 1.80 Å resolution. Close-up view of the active site in ball-and-stick representation. The inhibitor (cyan) is shown in sticks with the maximum likelihood weighted 2Fo–Fc electron density map contoured at 1σ. The catalytic zinc ion (dark gray) and the calcium ion (green) and water molecules (red) are shown as spheres (PDB code: 7Z5U).

We rationalized the screening results based on the crystal structure of **27** in complex with the ColG-PD, solved at a 1.80 Å resolution (Figure 4 and Table S2). The bound inhibitor occupied the active site from the S3 pocket to the S2′ binding site. In the S3 binding pocket, the *ortho*-acetamide group directly interacted with the edge strand *via* a hydrogen bond to the backbone amide of Glu498, while the aromatic phenoxy ring established π–π stacking interactions with the side chain of Trp539. This explains the observed preference for the *ortho*-configuration of the acetamide group.

The central triazole ring hydrogen bonded with Glu555 and formed a π–hydrogen interaction with Tyr599 in the S2 binding pocket. The hydroxamate group coordinated, as expected, the catalytic zinc ion. The isobutyl group in the *α-*position formed hydrophobic interactions with Tyr607 and His523 in the S2′ pocket. The hydrophobic benzyl group of **33** can be expected to fit similarly into the S2′ pocket.

#### Selectivity over Human MMPs and Activity against Other Bacterial Collagenases

To assess the selectivity of the compounds over human MMPs and on other bacterial collagenases, selected compounds were tested against ColG-CU and ColH-PD from *C. histolyticum* and ColA-CU from *B. cereus* strain ATCC14579. In addition, the compounds were tested on catalytic domains of three human MMPs, which are characterized by different depths of the S1′ binding pocket (MMP-1 (shallow), −2 (intermediate), and −3 (deep)). They were also tested against other important human off-targets, which are involved in gene expression and the processing of TNF-α; these include HDAC-3, -8, TACE (ADAM-17),^33,34^ and COX-1.

Our data showed that the compounds have high activity against most tested bacterial collagenases, which confirms their broad-spectrum inhibitory potency against bacterial targets (Table 5). This broad activity is comparable to that previously observed for compounds carrying thiol or phosphonate ZBGs.^25−27^

**Table 5:** Percent Inhibition of ColA-CU, ColH-PD, and ColG-CU in the Presence of 100 μM of Compounds **13 14, 15, 27**, **Tiludronate Disodium**, and **Alendronate Sodium**^a^

class	cpd.	ColA-CU	ColH-PD	ColG-CU
synthesized diphosphonates	**13**	71 ± 4	83 ± 9	68 ± 4
	**14**	82 ± 2	96 ± 2	72 ± 3
	**15**	86 ± 8	97 ± 5	70 ± 8
FDA-approved diphosphonates	**tiludronate disodium**	72 ± 2	91 ± 2	28 ± 7
	**alendronate sodium**	18 ± 4	25 ± 5	28 ± 7
hydroxamate	**27**	93 ± 2	84 ± 2	71 ± 7

a Means and SD of at least two independent experiments.

On the other hand, the compounds possess a high selectivity over most of the tested human off-targets (except for **13** against the tested MMPs and **tiludronate disodium** against MMP-1) (Tables 6 and S3).

**Table 6:** Inhibition of Compounds **13 14, 15, 27**, **Tiludronate Disodium**, and **Alendronate Sodium** against Three MMPs^a^

IC_50_ (μM)				
class	cpd.	MMP-1	MMP-2	MMP-3
synthesized diphosphonates	**13**	53 ± 3	79 ± 2	33 ± 11
	**14**	>100	>100	>100
	**15**	>100	>100	>100
FDA-approved diphosphonates	**tiludronate disodium**	50 ± 10	>100	>100
	**alendronate sodium**	>100	>100	>100
hydroxamate	**27**	>100	>100	>100

a Means and SD of two independent experiments, >100: IC_50_ is higher than 100 μM.

#### Cytotoxicity against Mammalian Cell Lines

Besides the selectivity, cytotoxicity is also an important criterion, especially when it comes to a potential therapeutic application in humans. In this context, we evaluated the cytotoxicity of **13**, **14**, **15**, and **27** against four mammalian cell lines, comprising HepG2 (hepatocellular carcinoma), HEK293 (embryonal kidney), NHDF (normal human dermal fibroblasts), and MDCK II (Madin–Darby canine kidney II) cells. Interestingly, the compounds did not show cytotoxic effects (IC_50_ values > 100 μM or 200 μM) against these cell lines (Table S4). This makes them suitable for further investigation to determine their ADMET profile.

#### Small-Molecule Collagenase Inhibitors Prevent Collagen I Cleavage

**Figure 5:**
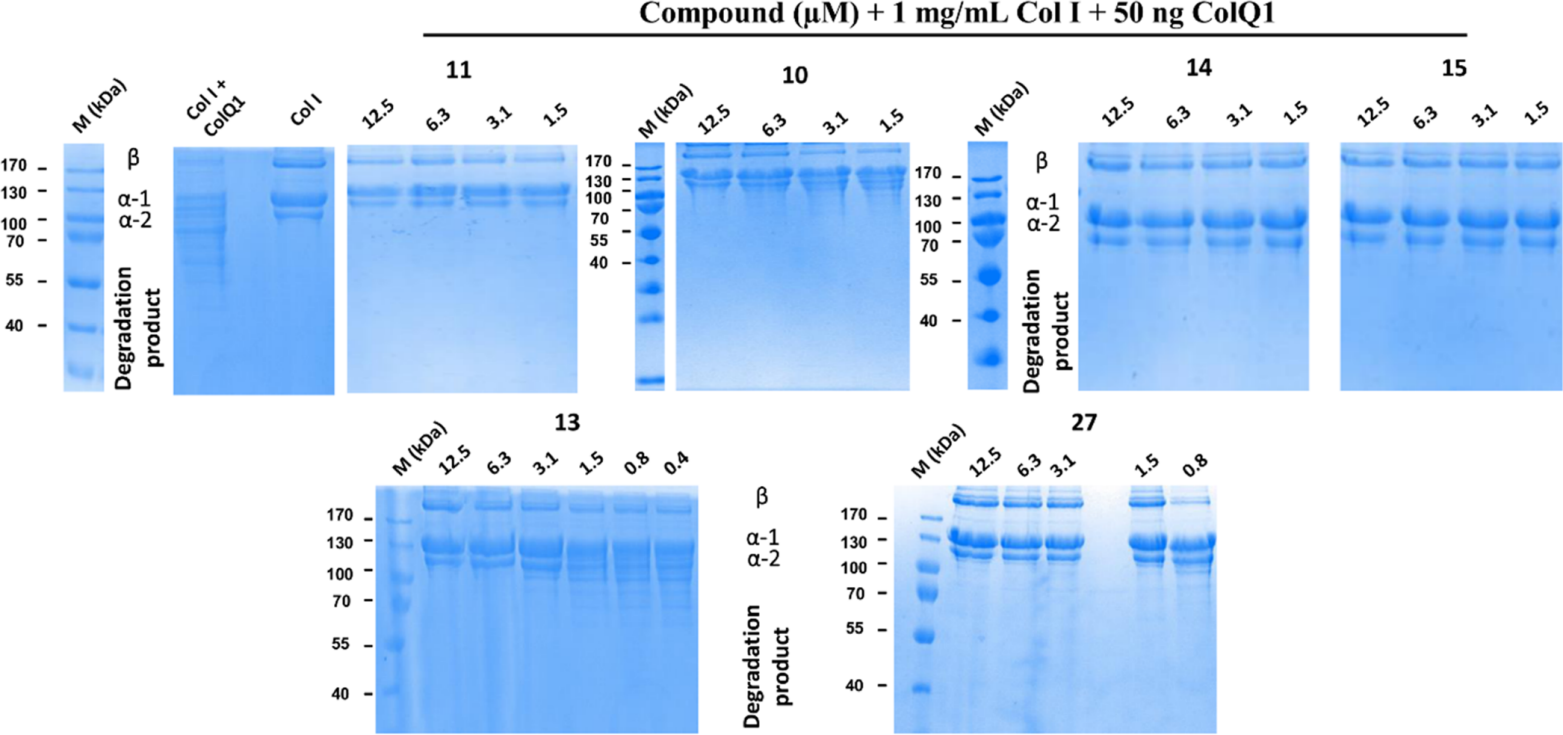
Activity of ColQ1 inhibitors against the collagenolytic activity of the full-length (FL) ColQ1. Inhibitors prevented the cleavage of 1 mg/mL of Col I chains (*i.e.,* β, α-1, and α-2). The *Bacillus cereus* ColQ1-FL (50 ng) was incubated with 1 mg/mL Col I for 3 h, and the degradation was then visualized by 12% SDS-PAGE. Col I: 1 mg/mL Col I without any protease. M (kDa): molecular weight standards, Col I: type I collagen, ColQ1: collagenase Q1.

We examined collagen I (Col I) cleavage induced by the full-length ColQ1-FL with and without inhibitors to investigate whether the compounds have an anticollagenolytic effect on the collagenase’s natural substrate. After 4 h of co-incubation of ColQ1-FL with Col I, in the absence and presence of inhibitor, Col I breakdown was investigated. On reducing polyacrylamide gels, the hallmark bands of Col I (*i.e.*, α1, α2, and β chains) were clearly visible. Compared to the negative control (no inhibitor), **13** and **27** displayed considerable anti-ColQ1 activity and maintained the chains of Col I, as shown at concentrations above 3 and 0.8 μM, respectively. Below these concentrations, substantial chain disintegration was detected (Figure 5). The diphosphonate derivatives **11**, **10, 14**, and **15** inhibited the collagenase activity at all concentrations tested (12.5–1.5 μM) (except for **10**, which showed inhibition only at 12.5 μM) and protected Col I chains from cleavage (Figure 4). Similar findings were observed for other diphosphonate derivatives **7**, **9**, and **11** and the hydroxamate derivative **33** (Figure S3). In contrast, severe degradation was visible at all tested concentrations (12.5–1.5 μM) of diphosphonate**16** and the hydroxamate derivatives **28**, **29**, **31**, and **32** (Figure S3), indicating their low activity on ColQ1.

These findings are corroborated by the previously determined inhibition data obtained with the peptidolytic assay. This highlights that several of our hydroxamate and diphosphonate compounds are capable *in vitro* of preventing cleavage of the large, structurally more complex physiological substrate of collagenases, *i.e.*, Col I, which accounts for 80–90% of the collagen in the body.^35^ Based on these findings, we next investigated whether this protective effect would also prevail in a more complex cellular setting.

### Small-Molecule Inhibitors Reduce Collagenase Activity and Preserve Fibroblast Cell Integrity

#### Collagenase Release during *B. cereus* Infection of NHDF Cells

To investigate the potential antivirulence activity of ColQ1 inhibitors, we developed an *in vitro* infection model using the common connective tissue cell line NHDF in the ECM. The fibroblasts have crucial functions in the ECM, as they synthesize their main components (such as collagen) and maintain their homeostasis.^36^ Previous reports revealed that bacillary ColQ1 and ColA have a prominent collagenolytic activity that is greater than or similar to the well-studied clostridial ColG and ColH.^20^ Therefore, *B. cereus* collagenases were used as model proteases to further evaluate the inhibitors’ effects in infection settings.

To establish the model, the release of *B. cereus* collagenases was investigated to determine the duration needed for the AH187 strain (expresses two collagenases: B7HV61 (sequence identical to ColQ1) and B7HZW5 (ColA)) to secrete considerable amounts of collagenase into the surrounding DMEM medium of NHDF cells. To inspect the release of collagenase, the DMEM medium was collected at various time points and investigated by gelatin zymography. The zymography analysis revealed that the bacteria required at least 4 h to release substantial amounts of collagenases, which increased over time, as seen in Figure S4b. In the zymograms, besides the bands of the full-length ColQ1 and ColA (109 kDa), also truncated isoforms (100–40 kDa) were detected (Figure S4b). This phenomenon has been previously reported for other bacterial collagenases.^19^ After 4 h of infection, we could also observe a massive reduction (>50%) in fibrillar collagen in the ECM quantified with the picrosirius red assay (Figure S4d).^37,38^ Concomitantly, NHDF cells started to detach and their morphology changed from spindle-shaped to round, as evidenced by light microscopy and SDS-PAGE analysis of the cell lysate (Figure S4a,c). In addition, we monitored the release of lactate dehydrogenase (LDH)^39^ into the DMEM to detect cells undergoing cell death. Significant amounts of LDH were excreted after 4 h, and the excretion increased over time (Figure S4e). Studies showed that *B. cereus* protein complexes hemolysin BL and nonhemolytic enterotoxin induced cytotoxicity and cell detachment, which means not only collagenases may induce cytotoxicity but other toxins also contribute.^40,41^ These results suggest that bacterial collagenases may play a role in inducing cellular necrosis. Based on these findings, we chose a time window of 4–6 h for testing the efficacy of collagenase inhibitors in the NHDF infection model.

#### Collagenase Inhibitors Suppress the Gelatinolytic Activity of *B. cereus* Collagenases Released in the NHDF Infection Model

**Figure 6:**
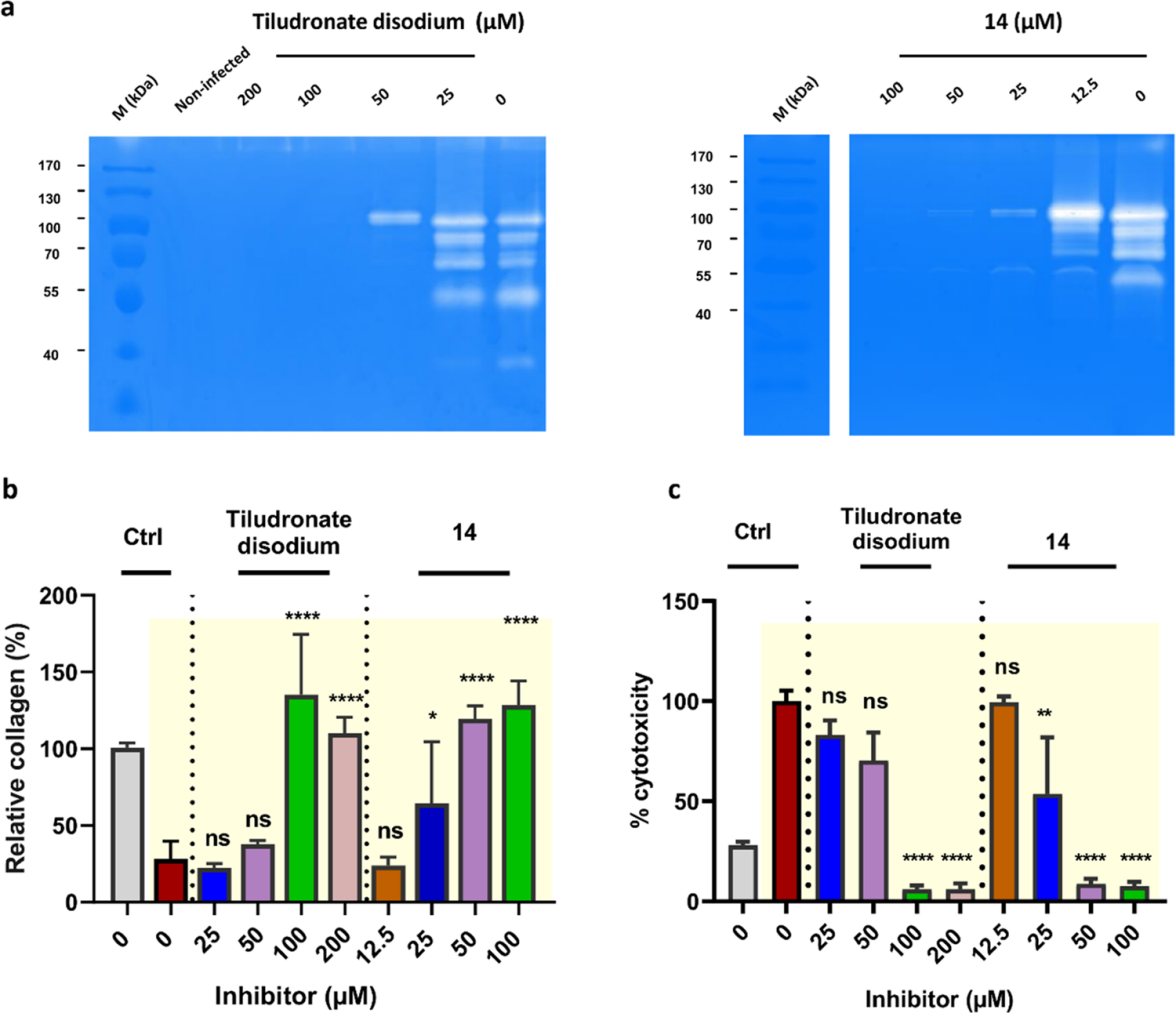
Activities of FDA-approved tiludronate disodium and compound **14** on the fibroblast (NHDF) cells infected with *Bacillus cereus*. (a) The antigelatinolytic activities of compounds **tiludronate disodium** and **14** against *B. cereus* collagenases. The DMEM medium of the infected NHDF cells was applied to the zymograms. Clear regions against blue background indicate that gelatin in the gel has been cleaved. (b) The amount of fibrillar collagens maintained by **tiludronate disodium** and **14** in the infected NHDF cells (highlighted in the yellow background). (c) The cytotoxicity of *B. cereus* infection (highlighted in the yellow background) in NHDF cells treated with and without **tiludronate disodium** and **14**. Ctrl represents the noninfected cells (gray column) and the infected cells and nontreated with inhibitors (red column). Statistical analysis was performed with one-way ANOVA, and statistical significance was analyzed by the Tukey test. Significance was calculated by comparing nontreated *vs* treated cells with compounds (mean ± SD, ****p < 0.0001, **p < 0.01, *p ≤ 0.05, ns: nonsignificant). Ctrl: control. M (kDa): molecular weight marker.

We investigated the most potent compounds from our *in vitro* cleavage assays in the NHDF infection model at concentrations ranging from 0, 25, 50, 100–200 μM. We observed a dose-dependent inhibitory effect on the activity of the secreted collagenases into the supernatant of infected cells detected by gelatin zymography. At 200 μM, **13** completely suppressed the gelatinolytic activity, while for **14** a complete inhibition was already observed at a concentration of 100 μM (Figures 6 and S5). The other diphosphonate derivatives (**10, 11**, and **15**) were also evaluated. Compound **15** also demonstrated a clear dose-dependent inhibitory effect on gelatin turnover (Figure S6), while this was less evident for **10** and **11**, which inhibited the collagenase activity toward gelatin only marginally at 100 μM (Figure S6).

The most promising FDA-approved diphosphonate drugs from the *in vitro* enzyme assays were also tested. **Tiludronate disodium** and **alendronate sodium** could completely inhibit gelatin turnover in the zymography at a concentration of 100 μM and 200 μM, respectively (Figures 6 and S9). Interestingly, the hydroxamate compounds (**27, 28, 29, 30, 31, 32**, and **33**) showed no inhibitory effect on the collagenase in the zymography at all tested concentrations (Figures S11 and S12).

#### Collagenase Inhibitors Prevent NHDF Cell Detachment and Cleavage of Fibrillar Collagens of the ECM

We further evaluated the effect of the diphosphonate and hydroxamate compounds on cell morphology, the fibrillar collagen content of the ECM, and cell viability in the infection model. As shown in Figures 6 and S5, the diphosphate compounds **13** and **14** showed a dose-dependent effect on the infected NHDF cells. Above a concentration of 25 μM, they were both able to sustain the cells during infection. The cells stayed attached and maintained their spindle shape (Figures S7 and S8), and above 50 μM, we observed a significant reduction in LDH release, indicating higher cell viability. The diphosphonate derivative **15** and the FDA-approved diphosphonate drugs **tiludronate disodium** and **alendronate sodium** also showed dose-dependent effects on NHDF morphology, fibrillar ECM collagen content, and LDH release, however, at a concentration of ≥100 μM (Figures 6 and S8–S10). In contrast, diphosphonates **10** and **11** showed a smaller effect at all concentrations tested (except for **10**, which resulted in higher cell viability and attachment at 100 μM) (Figures S6 and S8).

We also examined the hydroxamate compounds **27**, **28**, **29**, **31**, **32**, and **33**. Interestingly, none of them showed the expected effects. They were neither able to rescue cell morphology nor able to maintain the collagen content of the ECM. Also, no inhibition of the collagenase in the gelatin zymography was detected at all concentrations tested (Figures S11 and S12). Intrigued by the results of the hydroxamate compounds, we investigated their stability with LC-MS in the conditions of the NHDF infection model to obtain a potential explanation for the observed findings. We used **27** as a representative example for this compound class. The LC-MS spectra (Figure S13) confirmed, however, the stability of **27** under the assay conditions.

To sum up, in the NHDF infection model, the potent diphosphonate compounds from the *in vitro* enzyme assays were shown to have anticollagenolytic activity by inactivating the bacterial collagenases. Furthermore, they reduced the cytotoxicity induced directly or indirectly by the released collagenases and maintained the spindle-shaped morphology of the cells. In contrast, the hydroxamate-based compounds displayed almost no anticollagenolytic activity in the infection model, despite their inhibitory activity in the *in vitro* enzyme assays.

#### Rapid, Slow, or Very Slow Reversibility of Diphosphonate Inhibitors Depends on Target Collagenase

Next, we compared the mechanism inhibition of the diphosphonate and the hydroxamate compounds to the bacterial collagenases. For this purpose, we performed rapid dilution assays to test the reversibility of compound inhibition with ColA-CU and ColQ1-CU from *B. cereus* and ColH-PD and ColG-CU from *C. histolyticum*.^36^ Upon rapid dilution, rapidly reversible inhibitors quickly dissociate from the enzyme and progress curves similar to the uninhibited control are observed, while irreversible or very slowly dissociating inhibitors remain bound to the enzyme, which only very gradually recovers activity.

Intriguingly, as demonstrated in Figure S14, we observed clear differences between the compound classes. While hydroxamates **27** and **33** behaved toward both bacillary and clostridial collagenases like rapidly reversible inhibitors—as expected from active-site directed competitive inhibitors—the diphosphonate compounds showed a more varied response. In the case of ColQ1-CU, both **13** and **15** displayed progress curves with approximately less than 9% residual activity, which is typical for irreversible or very slowly dissociating inhibitors.^36^ Therefore, we examined ColQ1-CU treated overnight with **13** by mass spectrometry of the intact protein and could confirm that the compound did not result in a covalent modification (Figure S15). This leads to the conclusion that **13** must be a very slowly dissociating reversible binder of ColQ1. In the case of ColA-CU, the rapid dilution assay revealed only a minimal inhibition in the presence of **13**, but **15** displayed a progress curve typical for slowly dissociating inhibitors. In the case of ColH, the effect of **13** and **15** was reversed compared to ColA, and in the case of ColG, both diphosphonates behaved like rapidly reversible inhibitors (Figure S14).

Slowly and very slowly dissociating inhibition are interesting mechanisms for reducing enzyme activity, as they increase the lifetime of the enzyme–inhibitor complex. Its advantages appear in the latter phases of drug development when pharmacological properties (*i.e.*, dosing interval and patient safety) need to be optimized.^36,37^ We speculate that the differential behavior observed in the infection models between the diphosphonate and hydroxamate compounds is caused by their different dissociation properties, which results in different enzyme–inhibitor complex lifetimes. The exact mechanism underlying the slow dissociation behavior, however, remains currently elusive, as we could not get a crystal structure of a diphosphonate inhibitor complex with ColA or ColQ1. Other reasons could also be related to the limited activity of the hydroxamate in the infection experiments. For instance, the accumulation of the hydroxamates inside the infected cells or binding to the large substrate of collagenase (*i.e.*, collagen), which subsequently results in a reduced concentration available to bind with the extracellular bacterial collagenases.

### Small-Molecule Inhibitors Reduce Collagenase Activity and Maintain Epithelial Cell Integrity

#### Collagenase Inhibitors Maintain the TEER of MDCK II Cells

**Figure 7:**
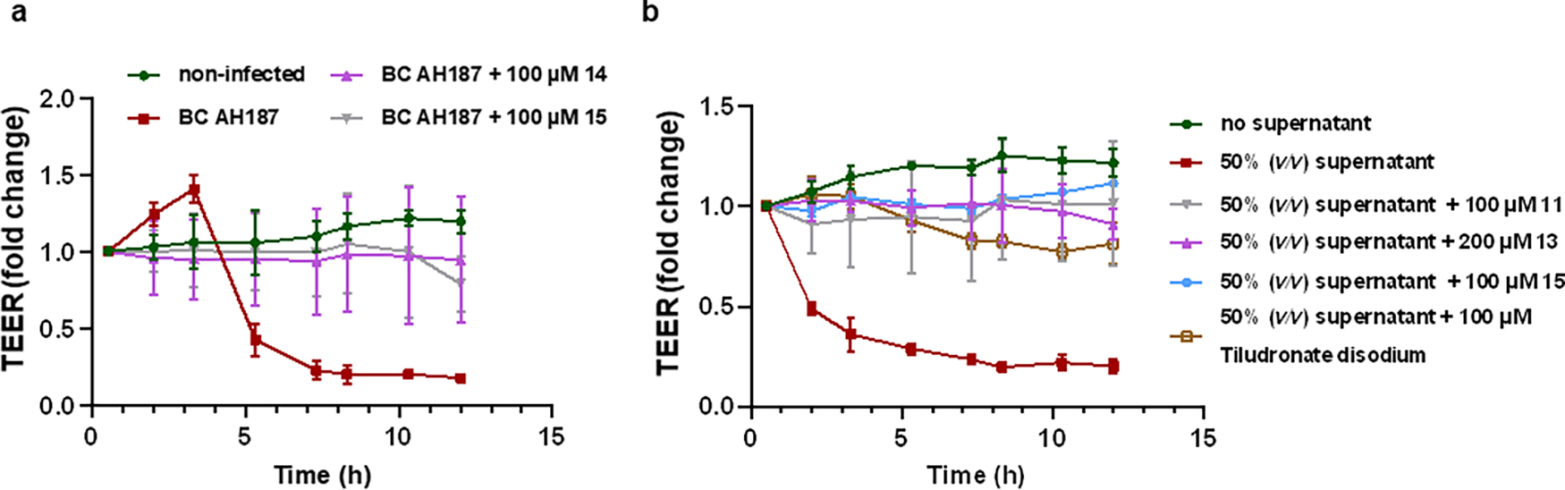
Change in the transepithelial electrical resistance (TEER) of the Madin–Darby canine kidney II (MDCK II) cells challenged with Bacillus cereus bacteria or 50% (*v/v)* culture supernatant and treated with or without collagenase inhibitors. (a) **14** and **15** preserve the TEER value of the MDCK II infected with *B. cereus* compared with the nontreated conditions with inhibitor. (b) Compounds **11, 13**, **15**, and **tiludronate disodium** maintained the TEER of MDCK II cells challenged with *B. cereus* supernatant. Each curve represents the average ± standard deviation of at least three independent experiments.

Epithelial cells form intercellular tight junctions establishing a sealed epithelium to control the diffusion of membrane proteins, the uptake of small molecules, and protect the body from hazardous substances.^42,43^*B. cereus*-mediated cell detachment severely destroys the epithelial barrier function, allowing bacterial access to deeper areas of the tissue. To quantify the effect on the epithelial barrier function, we investigated selected compounds regarding their effect on the transepithelial electrical resistance (TEER) of polarized MDCK II cells. We treated MDCK II cells with our target compounds followed by the infection with the *B. cereus* AH187 or coincubation with the supernatant from AH187 strain culture, which contains ColQ1 and ColA. In comparison to the control without inhibitor, the TEER of the infected MDCK cells (Figures 7 and S16) was maintained with compounds **14**, **15**, **tiludronate disodium**, and **alendronate sodium** at 100 μM. On the other hand, compounds **13** and **10** failed to sustain the TEER of the infected cells at 200 μM (Figure S16) but they retain the TEER of the challenged cells with 50% (*v*/*v*) supernatant (Figure 7b).

This discrepancy might be due to the lower amounts of collagenases secreted into the supernatant compared to the very high bacterial densities during infection that lead to a high collagenase secretion. This might have disrupted the optimum inhibitor/collagenase ratio and resulted in lower efficacy of some inhibitors. Thus, the effect of some inhibitors might be less in this case. Despite these variations in effect, our findings support the notion that collagenases are involved in attacking epithelial barriers and that our inhibitors can help to preserve the cellular junctions, thereby reducing bacterial invasion. These findings corroborate the theory that collagenases are one of the factors that might be involved in disturbing the TEER of epithelial cells. Our collagenase inhibitors proved their potential to preserve the cell attachment and the junction between them, subsequently reducing bacterial invasion.

#### Compounds Do Not Interfere with *B. cereus* Growth

The aim of this work was to develop an antivirulence agent that prevents bacteria from causing damage rather than killing them.^3^ Therefore, we tested the compounds on *B. cereus* growth to rule out antibacterial activity and ensure that the effects shown in the *in vitro* infection models were not caused by influencing bacterial viability. For this purpose, we selected the most potent compounds and tested them against the AH187 strain. As shown in Table S5, the compounds had no effect on AH187 growth and their minimum inhibitory concentration (MIC_50_) was >200 or >100 μM. These data support that the antivirulence activity, and not an antibacterial activity, was responsible for the observed effect in the infection experiments.

#### ColQ1 Inhibitors Maintain the Survival of *G. mellonella* Larvae

**Figure 8:**
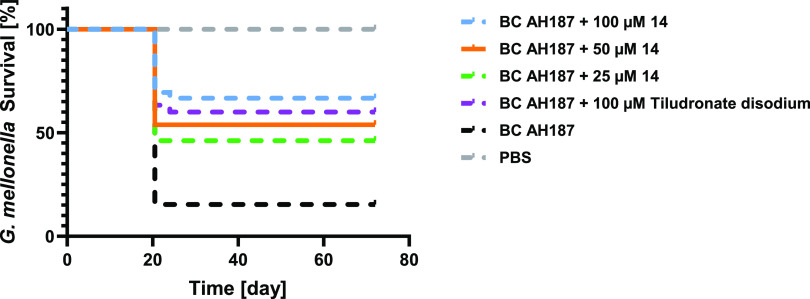
Survival analysis of *Galleria mellonella* larvae treated with *Bacillus cereus* AH187 with and without **14** and **tiludronate disodium**. Each curve represents results of three independent experiments; the statistical difference between groups treated with 100, 50, and 25 μM of compound **14** and *B. cereus* AH187 and with the group treated only with *B. cereus* AH187 is *p* < 0.0001, *p* = 0.0039, and *p* = 0.0173, respectively. The statistical difference between groups treated with 100 μM **tiludronate disodium** and with *B. cereus* AH187 is *p* = 0.0032 (log-rank test). The survival rate for the larvae treated with compound **14** and **tiludronate disodium** in PBS was 100%.

Finally, we evaluated selected compounds in an *in vivo* infection model. *G. mellonella* larvae are one of the most frequently used models to evaluate the effectiveness of newly discovered inhibitors and are well established for assessing *B. cereus* cytotoxicity.^44^ We reported previously that treating the *G. mellonella* larvae with ColQ1 caused death of the larvae.^28^ We established *B. cereus* infection of *G. mellonella* by injecting AH187 strain into the larvae in the presence or absence of our compounds. The larvae were incubated at 37 °C throughout the experiment, and their survival was monitored twice a day. Three synthesized inhibitors and two FDA-approved drugs were tested. Compounds **13**, **14**, **15**, **tiludronate disodium**, and **alendronate sodium** ameliorated the survival of the larvae in a dose-dependent manner when compared to a control where no inhibitor was administered. At 100 μM, compounds **14** and **15** boosted the survival by around 50% and 35%, respectively. At lower concentration (*i.e.*, 25 μM), both had a reduced effect (Figures 8 and S17). The FDA-approved **tiludronate disodium** and **alendronate sodium** increased the survival by 40% at 100 and 200 μM, respectively (Figures 8 and S17). A concentration of 200 μM of **13** showed the highest effect, increasing the survival by about 35%, while 50 μM showed the smallest effect and only improved survival by 5% (Figure S17). Overall, the data revealed the protective effect of the collagenase inhibition during *B. cereus* infection. As a result, these antivirulence compounds may be evaluated as a promising therapeutic agent in the future.

## Conclusions

To tackle the AMR crisis and the slow discovery of new anti-infectives, non-traditional therapies and FDA-approved drugs repurposing are promising strategies. One potential non-traditional approach is the use of antivirulence agents, which inhibit the pathogenicity factors of bacteria and thus prevent or delay infection without exerting selective pressure. Bacterial collagenases are one of the antivirulence targets that are currently gaining wide attention. In this study, we identified two classes of inhibitors (synthesized and FDA-approved diphosphonates and hydroxamates) that target clostridial and bacillary collagenases. Among these, compounds, **13**, **14**, **15**, **tiludronate disodium**, and **alendronate sodium** of the diphosphonate class and **27** and **33** of the hydroxamate class displayed high and broad-spectrum *in vitro* inhibition of clostridial collagenases ColH and ColG and on bacillary collagenases ColA and ColQ1. Furthermore, the majority of them demonstrated adequate selectivity over human MMPs and other off-targets. These compounds showed no cytotoxicity in four mammalian cell lines. We also studied the biological effects of these compounds in infection models using *B. cereus* as a representative bacterium-producing collagenase. In this context, we developed fibroblast- and epithelial cell infection models to characterize the effect of *B. cereus* collagenases and their inhibition. Our findings suggest that the inhibition of *B. cereus* collagenases by the most potent diphosphonate compounds maintained the fibroblast cell attachment, cell morphology, and cell viability, preserved the fibrillar collagen content, and sustained the TEER of epithelial cells. We also tested the compounds *in vivo* in the *G. mellonella* larvae infection model, where they enhanced the survival rate. The hydroxamates did not display any inhibition in the infection models; however, they showed similar potency as diphosphonates in the enzyme cleavage assays. This could be explained by their quick dissociation from the clostridial and bacillary collagenases. In contrast, the most active diphosphonate demonstrated high potency in both enzyme assays and infection models, which might be due to their slow to very slow dissociation from the bacillary collagenases. These findings offer insight into the role of bacterial collagenases in infections and the significance of their inhibition with small-molecule inhibitors and FDA-approved drugs, which might represent a potential treatment strategy in the future.

## Materials and Methods

### Chemistry

Chemical names follow the IUPAC nomenclature. Starting materials were purchased from Chempur, Sigma-Aldrich, Acros, Combi-Blocks, or Fluorochem and were used without purification. Column chromatography was performed using the automated flash chromatography system Combiflash Rf+ (Teledyne Isco) equipped with RediSepRf silica columns. The final products were dried in high vacuum. ^1^H NMR and ^13^C NMR spectra were measured on a Bruker AM500 spectrometer (at 500 and 125 MHz, respectively) at 300 K and on a Bruker Fourier 300 (at 300 and 75 MHz, respectively) at 300 K. Chemical shifts are reported in δ (parts per million: ppm) by reference to the hydrogenated residues of deuterated solvent as the internal standard: 2.05 ppm (^1^H NMR), 29.8, and 206.3 ppm (^13^C NMR) for acetone-*d*_6_, 2.50 ppm (^1^H NMR) and 39.52 ppm (^13^C NMR) for DMSO-*d*_6_. Signals are described as br (broad), s (singlet), d (doublet), t (triplet), dd (doublet of doublets), ddd (doublet of doublet of doublets), dt (doublet of triplets), and m (multiplet). All coupling constants (*J*) are given in Hertz (Hz). Mass spectrometry was performed on a TSQ Quantum (Thermo Fisher, Dreieich, Germany). The triple quadrupole mass spectrometer was equipped with an electrospray interface (ESI). Purity of compounds was determined by LC-MS using the area percentage method on the UV trace recorded at a wavelength of 254 nm and found to be >95%. The Surveyor-LC-system consisted of a pump, an auto sampler, and a PDA detector. The system was operated by the standard software Xcalibur. An RP C18 NUCLEODUR 100-5 (3 mm) column (Macherey-Nagel GmbH) was used as the stationary phase. All solvents were HPLC grade. In a gradient run, using acetonitrile and water, the percentage of acetonitrile (containing 0.1% trifluoroacetic acid) was increased from an initial concentration of 0% at 0 min to 100% at 13 min and kept at 100% for 2 min. The injection volume was 15 μL, and the flow rate was set to 800 μL/min. MS analysis was carried out at a needle voltage of 3000 V and a capillary temperature of 350 °C. Mass spectra were acquired in positive mode, using the electron spray ionization method, from 100 to 1000 *m*/*z*, and UV spectra were recorded at a wavelength of 254 nm and in some cases at 360 nm. High-resolution mass spectrometry (HRMS) measurements were recorded on a SpectraSystems-MSQ LC-MS system (Thermo Fisher).

### Experimental Procedures of Diphosphonates

The following compounds were prepared according to previously described procedures: **7a–16a** and **7b–14b**.^29−31^

#### General Procedure A: Preparation of 1,4-Dihydro-2,3-quinoxaline-dione Derivatives **7a–14a** and **15b**

A mixture of 1,2-phenylenediamine derivative (1 equiv) and oxalic acid (1.2 equiv) was refluxed in 4 N HCl (20 mL) for 6 h, cooled to RT, poured over ice, and filtered. The product was washed with water and dried to give the title compound. The product was used in the next step without further purification.

#### General Procedure B: Preparation of 2,3-Dichloroquinoxaline Derivatives **7b–14b** and **15c**

A mixture of 1,4-dihydro-2,3-quinoxalinediones **7a–14a** and **15b** (2 mmol) and POCl_3_ (10 mL) was stirred at 50 °C in DMF (30 mL) for 2 h, cooled to RT, poured over ice, and filtered. The product was washed with water and dried to give the title compound. The product was used in the next step without further purification.

#### General Procedure C: Preparation of Diethyl Phosphonate Derivatives **7c–13c**, **14d**, **15d**, and **16b**

2,3-Dichloroquinoxaline derivatives **7b–14b**, **15c**, and **16a** (1 equiv) were suspended in triethyl phosphite (10 equiv) and heated to 150 °C in a sealed tube for 18 h. Most of the unreacted triethyl phosphite was evaporated *in vacuo*, and the resultant oil was purified by column chromatography.

#### General Procedure D: Preparation of Phosphonic Acid Derivatives **7–16**

To a solution of diethyl phosphonate (1 equiv) in dry DCM, bromotrimethylsilane (10 equiv) was added dropwise over a period of 15 min. The reaction mixture was stirred at RT overnight. Then, MeOH was added and stirred at RT for 30 min. Solvents were concentrated *in vacuo*, and the resultant oil was purified by preparative HPLC.

##### 6-(4-Chlorophenyl)-1,4-dihydroquinoxaline-2,3-dione (**15b**)

Compound **15b** was synthesized according to general procedure A, using 4′-chloro-[1,1′-biphenyl]-3,4-diamine **15a** (110 mg, 0.5 mmol) and oxalic acid (54 mg, 0.6 mmol). The product was obtained as a yellow solid (90 mg, 66%). ^1^H NMR (500 MHz, DMSO-d_6_) δ: 11.96 (d, *J* = 10.7 Hz, 2H), 7.57 (d, *J* = 8.7 Hz, 2H), 7.53 (d, *J* = 8.7 Hz, 2H), 7.41 (dd, *J* = 8.5, 1.9 Hz, 1H), 7.37 (d, *J* = 1.9 Hz, 1H), 7.22 (d, *J* = 8.5 Hz, 1H). 13C NMR (126 MHz, DMSO-d_6_) δ: 155.1, 155.5, 138.4, 133.9, 132.1, 129.0, 127.8, 126.6, 126.2, 122.0, 116.4, 113.1. MS (ESI^+^) *m*/*z* 273 [*M* + H]^+^.
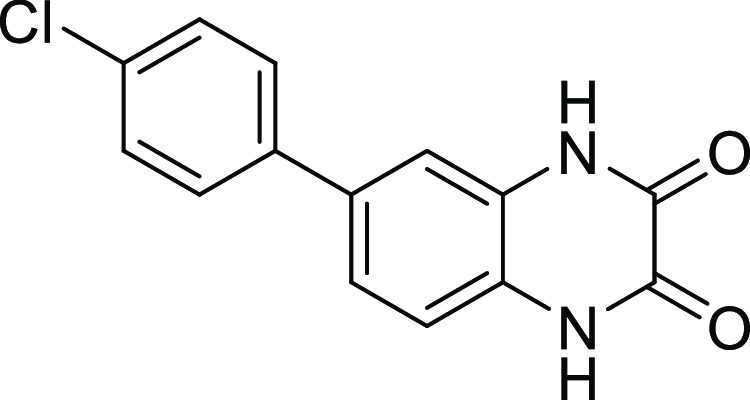


##### Tetraethyl Quinoxaline-2,3-diylbis(phosphonate) (**7c**)

Compound **7c** was synthesized according to general procedure C, using 2,3-dichloroquinoxaline **7b** (90 mg, 0.45 mmol) and triethyl phosphite (819 μL, 4.5 mmol). The residue was purified by automated column chromatography (Hex/EtOAc = 1/1) to give the desired product (white solid, 129 mg, 71%). ^1^H NMR (500 MHz, CDCl_3_) δ 8.12 (dd, *J* = 6.4, 3.5 Hz, 2H), 7.83 (dd, *J* = 6.4, 3.4 Hz, 2H), 4.56–3.98 (m, 8H), 1.37 (t, *J* = 7.1 Hz, 12H). ^13^C NMR (126 MHz, CDCl_3_) δ 149.9 (dd, *J*_C-P_ = 226.4, 30.0 Hz), 140.9–140.1 (m), 132.4, 129.8, 63.9 (t, *J*_C-P_ = 3.2 Hz), 19.3–9.9 (m). ^31^P NMR (202 MHz, CDCl_3_) δ 6.83. MS (ESI^+^) *m*/*z* 403.05 [*M* + H]^+^.
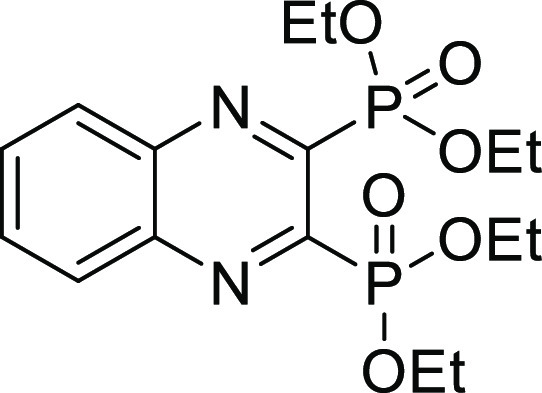


##### 2,3-Dichloro-*N*-(3,4-dichlorophenyl)quinoxaline-6-carboxamide (**14c**)

2,3-Dichloroquinoxaline-6-carboxylic acid (122 mg, 0.50 mmol) was dissolved in DCM (20 mL). EDC·HCl (191 mg, 1 mmol) was added, followed by 3,4-dichloroaniline (162 mg, 1 mmol). The reaction was stirred at room temperature for 24 h. The crude product was purified using column chromatography DCM to DCM/MeOH 1%. The product was obtained as a white solid (107 mg, 55%). ^1^H NMR (500 MHz, DMSO) δ 10.87 (s, 1H), 8.71 (s, 1H), 8.39 (d, *J* = 6.7 Hz, 1H), 8.29–8.13 (m, 2H), 7.74 (dd, *J* = 70.6, 7.2 Hz, 2H). ^13^C NMR (126 MHz, DMSO) δ 164.7, 147.0, 146.4, 141.8, 139.8, 139.4, 136.9, 131.4, 131.1, 130.6, 128.8, 127.9, 126.0, 122.0, 120.8. MS (ESI^+^) *m*/*z* 387.96 [*M* + H]^+^.
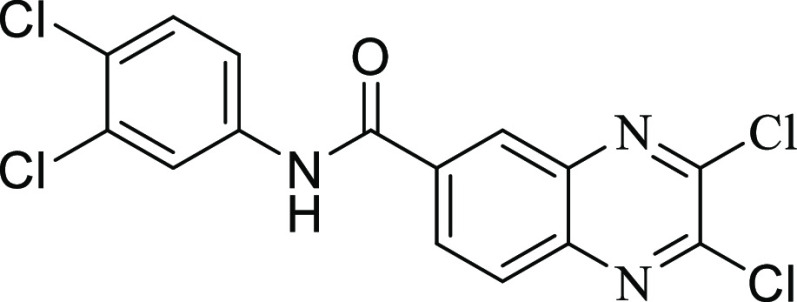


##### 2,3-Dichloro-6-(4-chlorophenyl)quinoxaline (**15c**)

Compound **15c** was synthesized according to general procedure B, using 6-(4-chlorophenyl)-1,4-dihydroquinoxaline-2,3-dione **15b** (85 mg, 0.31 mmol) and POCl_3_ (5 mL). The product was obtained as a white solid (69 mg, 72%). ^1^H NMR (500 MHz, DMSO) δ 8.38 (d, *J* = 1.9 Hz, 1H), 8.28 (dd, *J* = 8.8, 2.0 Hz, 1H), 8.16 (d, *J* = 8.8 Hz, 1H), 7.94 (d, *J* = 8.5 Hz, 2H), 7.61 (d, *J* = 8.5 Hz, 2H). ^13^C NMR (126 MHz, DMSO) δ 145.6, 145.0, 142.1, 140.8, 139.9, 137.2, 134.2, 131.0, 129.7, 129.6, 128.9, 125.3.
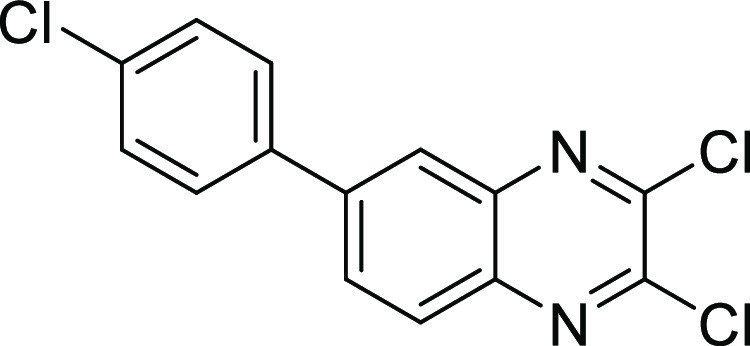


##### Quinoxaline-2,3-diylbis(phosphonic acid) (**7**)

Compound **7** was synthesized according to general procedure D, using tetraethyl quinoxaline-2,3-diylbis(phosphonate) **7c** (120 mg, 0.29 mmol), bromotrimethylsilane (444 μL, 2.9 mmol), and DCM (15 mL). The reaction was stirred at RT overnight. The crude product was purified using preparative HPLC (CH_3_CN (HCOOH 0.05%)/H_2_O (HCOOH 0.05%) = 0.2:9.8 to 10:0). The product was obtained as a white solid (53 mg, 61%). ^1^H NMR (500 MHz, DMSO) δ 8.23 (dd, *J* = 6.4, 3.4 Hz, 2H), 8.04 (dd, *J* = 6.4, 3.4 Hz, 2H). ^13^C NMR (126 MHz, DMSO) δ 153.1 (dd, *J*_C-P_ = 203.1, 30.4 Hz), 140.6 (dd, *J*_C-P_ = 20.1, 4.5 Hz), 133.0, 129.7. ^31^P NMR (202 MHz, DMSO) δ 5.24. Purity: 100%. HRMS (ESI^–^) calculated for C_8_H_7_N_2_O_6_P_2_ [*M* – H]^−^ 288.9784, found 288.9785.
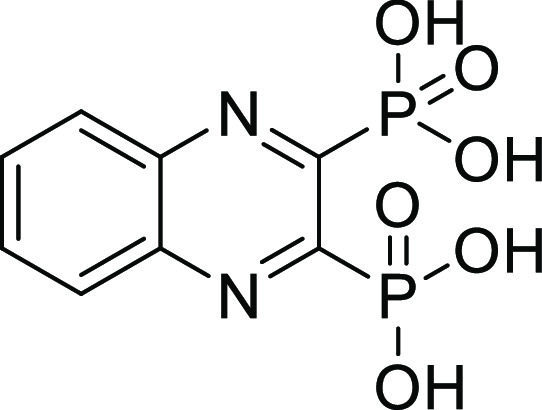


##### (6-Fluoroquinoxaline-2,3-diyl)bis(phosphonic acid) (**8**)

Compound **8** was synthesized according to general procedure D, using tetraethyl (6-fluoroquinoxaline-2,3-diyl)bis(phosphonate) **8c** (120 mg, 0.29 mmol), bromotrimethylsilane (444 μL, 2.9 mmol), and DCM (15 mL). The reaction was stirred at RT overnight. The crude product was purified using preparative HPLC (CH_3_CN (HCOOH 0.05%)/H_2_O (HCOOH 0.05%) = 0.2:9.8 to 10:0). The product was obtained as a white solid (63 mg, 72%). ^1^H NMR (500 MHz, MeOD) δ 8.31 (dd, *J* = 9.3, 5.7 Hz, 1H), 7.91 (dd, *J* = 8.9, 2.7 Hz, 1H), 7.85 (td, *J* = 8.8, 2.8 Hz, 1H). ^13^C NMR (126 MHz, MeOD) δ 165.6 (d, *J*_C-F_ = 255.4 Hz), 153.5 (dd, *J*_C-P,C-P_ = 208.9, 30.7 Hz), 151.9 (ddd, *J*_C-P,C-P_ = 210.6, 30.4, 3.3 Hz), 143.2 (ddd, *J*_C-P,C-F_ = 19.1, 13.8, 2.5 Hz), 139.7 (dd, *J*_C-P,C-F_ = 18.8, 2.2 Hz), 133.4 (d, *J*_C-F_ = 10.3 Hz), 124.3 (d, *J*_C-F_ = 26.9 Hz), 113.8 (d, *J*_C-F_ = 22.1 Hz). ^31^P NMR (202 MHz, MeOD) δ 6.89 (d, *J* = 7.1 Hz), 6.66 (d, *J* = 6.9 Hz). Purity: 96%. HRMS (ESI^–^) calculated for C_8_H_6_FN_2_O_6_P_2_ [*M*– H]^−^ 306.9690, found 306.9690.
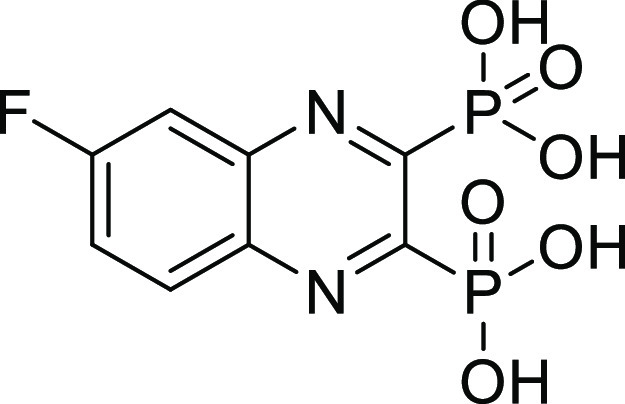


##### (6-Chloroquinoxaline-2,3-diyl)bis(phosphonic acid) (**9**)

Compound **9** was synthesized according to general procedure D, using tetraethyl (6-chloroquinoxaline-2,3-diyl)bis(phosphonate) **9c** (110 mg, 0.25 mmol), bromotrimethylsilane (326 μL, 2.5 mmol), and DCM (15 mL). The reaction was stirred at RT overnight. The crude product was purified using preparative HPLC (CH_3_CN (HCOOH 0.05%)/H_2_O (HCOOH 0.05%) = 0.2:9.8 to 10:0). The product was obtained as a white solid (48 mg, 59%). ^1^H NMR (500 MHz, DMSO) δ 8.28 (d, *J* = 2.3 Hz, 1H), 8.24 (d, *J* = 9.0 Hz, 1H), 8.04 (dd, *J* = 9.0, 2.3 Hz, 1H). ^13^C NMR (126 MHz, DMSO) δ 154.3 (dd, *J*_C-P_ = 201.5, 30.2 Hz), 153.6 (dd, *J*_C-P_ = 202.3, 30.3 Hz), 140.9 (dd, *J*_C-P_ = 18.4, 2.6 Hz), 139.3 (dd, *J*_C-P_ = 18.3, 2.7 Hz), 137.3, 133.5, 131.6, 128.3. ^31^P NMR (202 MHz, DMSO) δ 4.87 (d, *J* = 5.3 Hz), 4.72 (d, *J* = 6.1 Hz). Purity: 95%. HRMS (ESI^–^) calculated for C_8_H_6_ClN_2_O_6_P_2_ [*M* – H]^−^ 322.9395, found 322.9395.
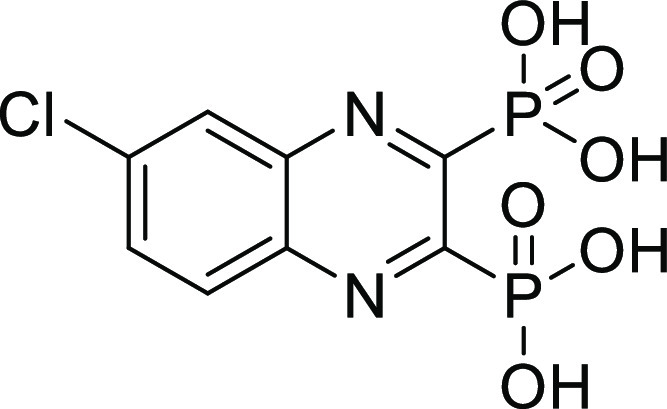


##### (6-Bromoquinoxaline-2,3-diyl)bis(phosphonic acid) (**10**)

Compound **10** was synthesized according to general procedure D, using tetraethyl (6-bromoquinoxaline-2,3-diyl)bis(phosphonate) **10c** (80 mg, 0.16 mmol), bromotrimethylsilane (209 μL, 1.6 mmol), and DCM (15 mL). The reaction was stirred at RT overnight. The crude product was purified using preparative HPLC (CH_3_CN (HCOOH 0.05%)/H_2_O (HCOOH 0.05%) = 0.2:9.8 to 10:0). The product was obtained as a white solid (46 mg, 75%). ^1^H NMR (500 MHz, DMSO) δ 8.42 (s, 1H), 8.14 (d, *J* = 0.8 Hz, 2H). ^13^C NMR (126 MHz, DMSO) δ 154.4 (dd, *J*_C-P_ = 200.9, 30.1 Hz), 153.8 (dd, *J*_C-P_ = 201.7, 30.0 Hz), 141.1 (dd, *J*_C-P_ = 18.3, 2.4 Hz), 139.5 (dd, *J*_C-P_ = 18.1, 2.2 Hz), 136.0, 131.6, 131.5, 126.1. ^31^P NMR (202 MHz, DMSO) δ 4.90 (d, *J* = 7.1 Hz), 4.71 (d, *J* = 7.1 Hz). Purity: 100%. HRMS (ESI^–^) calculated for C_8_H_6_BrN_2_O_6_P_2_ [*M* – H]^−^ 366.8890, found 366.8890.
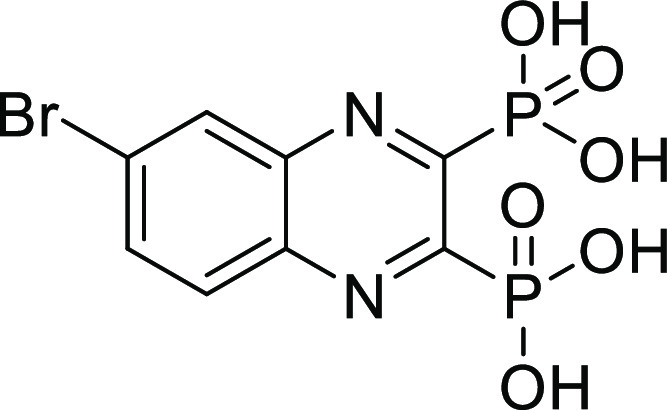


##### (6-Methylquinoxaline-2,3-diyl)bis(phosphonic acid) (**11**)

Compound **11** was synthesized according to general procedure D, using tetraethyl (6-methylquinoxaline-2,3-diyl)bis(phosphonate) **11c** (110 mg, 0.26 mmol), bromotrimethylsilane (340 μL, 2.6 mmol), and DCM (15 mL). The reaction was stirred at RT overnight. The crude product was purified using preparative HPLC (CH_3_CN (HCOOH 0.05%)/H_2_O (HCOOH 0.05%) = 0.2:9.8 to 10:0). The product was obtained as a white solid (64 mg, 80%). ^1^H NMR (500 MHz, DMSO) δ 8.10 (d, *J* = 8.6 Hz, 1H), 7.98 (s, 1H), 7.87 (dd, *J* = 8.6, 1.7 Hz, 1H), 2.63 (s, 3H). ^13^C NMR (126 MHz, DMSO) δ 152.9 (dd, *J*_C-P_ = 203.4, 30.5 Hz), 151.9 (dd, *J*_C-P_ = 204.2, 30.5 Hz), 143.6, 140.7 (dd, *J*_C-P_ = 17.4, 1.4 Hz), 139.2 (dd, *J*_C-P_ = 17.5, 1.4 Hz), 135.2, 129.2, 128.2, 21.9. ^31^P NMR (202 MHz, DMSO) δ 5.60 (d, *J* = 5.2 Hz), 5.48 (d, *J* = 7.0 Hz). Purity: 95%. HRMS (ESI^–^) calculated for C_9_H_9_N_2_O_6_P_2_ [*M* – H]^−^ 302.9941, found 302.9941.
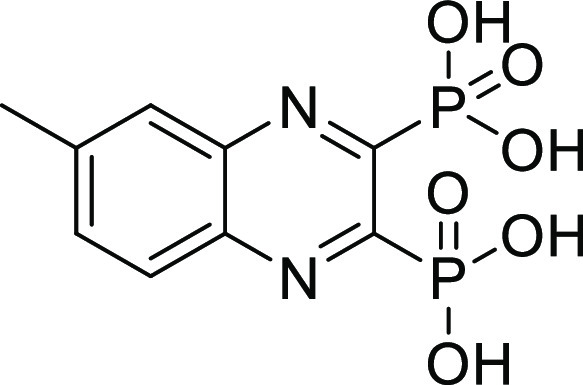


##### (6-Methoxyquinoxaline-2,3-diyl)bis(phosphonic acid) (**12**)

Compound **12** was synthesized according to general procedure D, using tetraethyl (6-methoxyquinoxaline-2,3-diyl)bis(phosphonate) **12c** (90 mg, 0.20 mmol), bromotrimethylsilane (260 μL, 2.0 mmol), and DCM (15 mL). The reaction was stirred at RT overnight. The crude product was purified using preparative HPLC (CH_3_CN (HCOOH 0.05%)/H_2_O (HCOOH 0.05%) = 0.2:9.8 to 10:0). The product was obtained as a white solid (46 mg, 70%). ^1^H NMR (500 MHz, DMSO) δ 8.11 (d, *J* = 9.2 Hz, 1H), 7.66 (dd, *J* = 9.2, 2.6 Hz, 1H), 7.52 (d, *J* = 2.4 Hz, 1H), 4.02 (s, 3H). ^13^C NMR (126 MHz, MeOD) δ 163.1, 152.9 (dd, *J*_C-P_ = 203.0, 30.3 Hz), 151.9 (dd, *J*_C-P_ = 204.1, 30.3 Hz), 142.6 (dd, *J*_C-P_ = 17.1, 1.4 Hz), 139.2 (dd, *J*_C-P_ = 17.4, 1.4 Hz), 130.3, 126.1, 106.0, 55.3. ^31^P NMR (202 MHz, DMSO) δ 5.99 (d, *J* = 4.6 Hz), 5.68 (d, *J* = 6.0 Hz). Purity: 100%. HRMS (ESI^–^) calculated for C_9_H_9_N_2_O_7_P_2_ [*M* – H]^−^ 318.9890, found 318.9890.
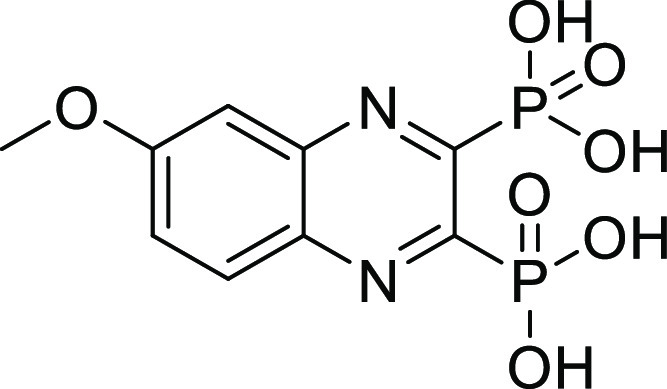


##### (6,7-Dichloroquinoxaline-2,3-diyl)bis(phosphonic acid) (**13**)

Compound **13** was synthesized according to general procedure D, using tetraethyl (6,7-dichloroquinoxaline-2,3-diyl)bis(phosphonate) **13c** (80 mg, 0.17 mmol), bromotrimethylsilane (222 μL, 1.7 mmol), and DCM (15 mL). The reaction was stirred at RT overnight. The crude product was purified using preparative HPLC (CH_3_CN (HCOOH 0.05%)/H_2_O (HCOOH 0.05%) = 0.2:9.8 to 10:0). The product was obtained as a white solid (39 mg, 65%). ^1^H NMR (500 MHz, DMSO) δ 8.51 (s, 2H). ^13^C NMR (126 MHz, DMSO) δ 155.1 (dd, *J*_C-P_ = 199.4, 29.9 Hz), 139.6 (dd, *J*_C-P_ = 19.9, 3.9 Hz), 135.58, 130.57. ^31^P NMR (202 MHz, DMSO) δ 4.49. Purity: 98%. HRMS (ESI^–^) calculated for C_8_H_5_Cl_2_N_2_O_6_P_2_ [*M* – H]^−^ 356.9005, found 356.9005.
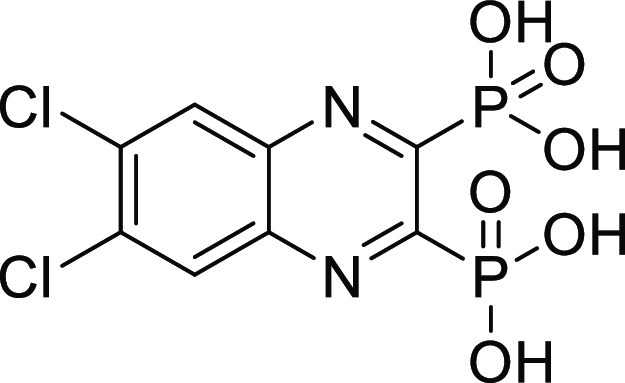


##### (6-((3,4-Dichlorophenyl)carbamoyl)quinoxaline-2,3-diyl)bis(phosphonic acid) (**14**)

Compound **14** was synthesized according to general procedure D, using tetraethyl (6-((3,4-dichlorophenyl)carbamoyl)quinoxaline-2,3-diyl)bis(phosphonate) **14d** (100 mg, 0.17 mmol), bromotrimethylsilane (222 μL, 1.7 mmol), and DCM (15 mL). The reaction was stirred at RT overnight. The crude product was purified using preparative HPLC (CH_3_CN (HCOOH 0.05%)/H_2_O (HCOOH 0.05%) = 0.2:9.8 to 10:0). The product was obtained as a white solid (43 mg, 53%). ^1^H NMR (500 MHz, DMSO) δ 10.99 (s, 1H), 8.87 (d, *J* = 1.8 Hz, 1H), 8.45 (dd, *J* = 8.8, 1.8 Hz, 1H), 8.33 (d, *J* = 8.8 Hz, 1H), 8.25 (d, *J* = 2.4 Hz, 1H), 7.84 (dd, *J* = 8.8, 2.4 Hz, 1H), 7.68 (d, *J* = 8.8 Hz, 1H). ^13^C NMR (126 MHz, DMSO) δ 164.9, 155.6 (dd, *J*_C-P_ = 70.9, 29.7 Hz), 154.0 (dd, *J*_C-P_ = 71.9, 30.0 Hz), 141.8 (dd, *J*_C-P_ = 18.3, 2.7 Hz), 139.9 (dd, *J*_C-P_ = 18.4, 2.6 Hz), 139.5, 137.3, 131.4, 131.2, 131.1, 130.1, 129.3, 126.0, 122.0, 120.8. ^31^P NMR (202 MHz, DMSO) δ 4.95 (d, *J* = 4.8 Hz), 4.71 (d, *J* = 6.0 Hz). Purity: 98%. HRMS (ESI^–^) calculated for C_15_H_10_Cl_2_N_3_O_7_P_2_ [*M* – H]^−^ 475.9376, found 475.9376.
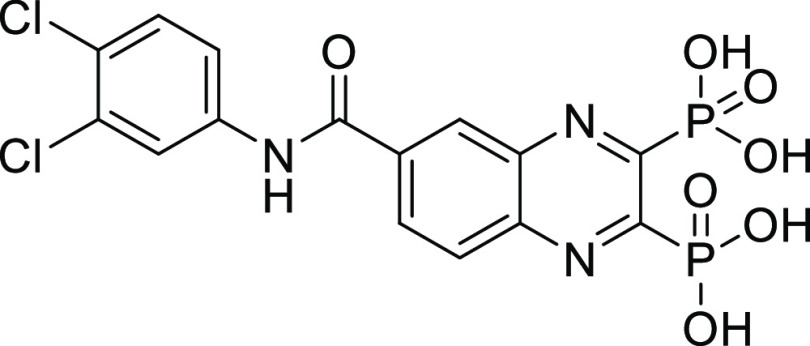


##### (6-(4-Chlorophenyl)quinoxaline-2,3-diyl)bis(phosphonic acid) (**15**)

Compound **15** was synthesized according to general procedure D, using tetraethyl (6-(4-chlorophenyl)quinoxaline-2,3-diyl)bis(phosphonate) **15d** (70 mg, 0.13 mmol), bromotrimethylsilane (170 μL, 1.3 mmol), and DCM (15 mL). The reaction was stirred at RT overnight. The crude product was purified using preparative HPLC (CH_3_CN (HCOOH 0.05%)/H_2_O (HCOOH 0.05%) = 0.2:9.8 to 10:0). The product was obtained as a white solid (24 mg, 44%). ^1^H NMR (500 MHz, DMSO) δ 8.45 (d, *J* = 1.9 Hz, 1H), 8.37 (dd, *J* = 8.8, 2.0 Hz, 1H), 8.29 (d, *J* = 8.8 Hz, 1H), 8.04–7.96 (m, 2H), 7.67–7.59 (m, 2H). ^13^C NMR (126 MHz, DMSO) δ 154.4 (dd, *J*_C-P_ = 88.9, 30.6 Hz), 152.8 (dd, *J*_C-P_ = 89.0, 29.9 Hz), 142.7, 140.9 (dd, *J*_C-P_ = 20.3, 5.2 Hz), 140.0 (dd, *J*_C-P_ = 20.4, 5.1 Hz), 137.4, 134.3, 131.8, 130.3, 129.8, 129.7, 126.5. ^31^P NMR (202 MHz, DMSO) δ 5.31, 5.27. Purity: 98%. HRMS (ESI^–^) calculated for C_14_H_10_ClN_2_O_6_P_2_ [*M* – H]^−^ 398.9708, found 398.9707.
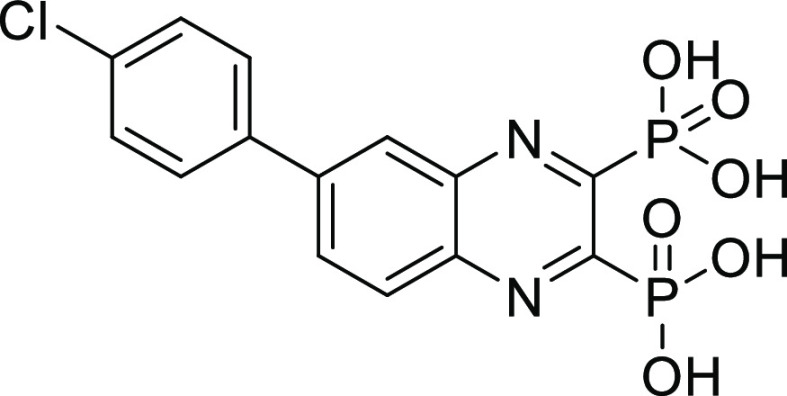


##### (6,7-Dichloro-3-oxo-3,4-dihydroquinoxalin-2-yl)phosphonic Acid (**16**)

Compound **16** was synthesized according to general procedure D, using diethyl (6,7-dichloro-3-oxo-3,4-dihydroquinoxalin-2-yl)phosphonate **16b** (90 mg, 0.25 mmol), bromotrimethylsilane (326 μL, 2.5 mmol), and DCM (15 mL). The reaction was stirred at RT overnight. The crude product was purified using preparative HPLC (CH_3_CN (HCOOH 0.05%)/H_2_O (HCOOH 0.05%) = 0.2:9.8 to 10:0). The product was obtained as a white solid (32 mg, 43%). ^1^H NMR (500 MHz, DMSO) δ 8.10 (s, 1H), 7.48 (s, 1H). ^13^C NMR (126 MHz, DMSO) δ 159.9 (d, *J*_C-P_ = 213.5 Hz), 154.0 (d, *J*_C-P_ = 28.6 Hz), 134.4, 132.9 (d, *J*_C-P_ = 2.3 Hz), 131.2 (d, *J*_C-P_ = 24.4 Hz), 130.7, 125.7, 117.2. ^31^P NMR (202 MHz, DMSO) δ 1.54. Purity: 95%. HRMS (ESI^–^) calculated for C_8_H_4_Cl_2_N_2_O_4_P [*M* – H]^−^ 292.9291, found 292.9290.
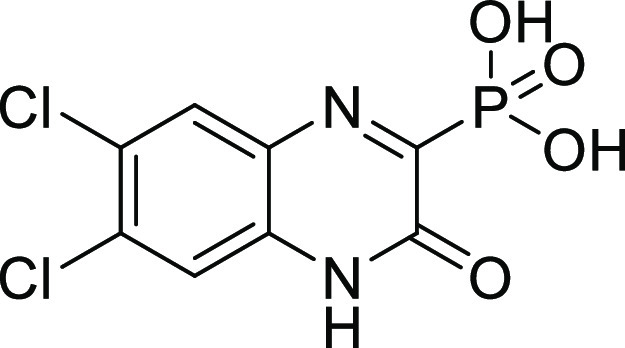


### Experimental Procedures of Hydroxamates

#### General Procedure A2: Monosaponification

Malonate diester (1 equiv) was dissolved in a mixture of ethanol/water (4:1, 0.43–0.47 M), and sodium hydroxide (1.2 equiv) was added. The reaction mixture was stirred at RT overnight. Then, solvents were evaporated under reduced pressure, and the aqueous mixture remaining was diluted with sat. aq. NaHCO_3_ and washed with CH_2_Cl_2_. The aqueous layer was then carefully acidified (pH ∼ 1) with aq. HCl and extracted with CH_2_Cl_2_. The combined organic layers were dried over MgSO_4_, filtered, and concentrated under reduced pressure affording the desired product.

#### General Procedure B2: Amide Formation

Carboxylic acid (1 equiv) and amine (1.1 equiv) were dissolved in CH_2_Cl_2_ (0.21 M). HOBt (0.1 equiv), EDC.HCl (1.5 equiv), and diisopropylethylamine (3 equiv) were then added, and the mixture was stirred at RT overnight. The mixture was then washed with diluted aq. HCl (1 M), sat. aq. NaHCO_3_, and sat. aq. NaCl. The combined organic layers were dried over MgSO_4_, filtered, and concentrated under reduced pressure or the residue was finally purified by flash chromatography to give the desired amide.

#### General Procedure C2: Boc Deprotection

The Boc-protected intermediate was dissolved in a mixture of ethanol and dichloromethane (1:1, 0.08–0.14 M), and the reaction was cooled down to 0 °C before the addition of 4 N HCl in dioxane (0.17–0.28 M). The mixture was stirred at RT overnight. Then, solvents were evaporated to give the desired compound.

#### General Procedure D2: Diazo Transfer

A suspension of amine (1 equiv), ZnCl_2_ (0.06 equiv), and K_2_CO_3_ (1 equiv) in ethanol (0.8–0.93 M) under an inert atmosphere was cooled down to 0 °C with an ice bath. Separately, diisopropylethylamine (1.1–3.5 equiv) was slowly added to a solution of 1*H*-imidazole-1-sulfonyl azide; hydrogen chloride (1.2 equiv) was dissolved in ethanol (0.33–0.74 M) under an inert atmosphere (solution A). The azide-containing solution A was immediately added dropwise to the first mixture at 0 °C. Then, the cooling bath was removed, and the white mixture was stirred at RT overnight. The mixture was then cooled down to 0 °C, diluted with water, and carefully acidified to pH = 2 with 1 N aq. HCl. It was finally extracted with ethyl acetate. The combined organic layers were dried over MgSO_4_, filtered, and concentrated under reduced pressure or the residue was finally purified by flash chromatography to give the desired azide.

#### General Procedure E2: Aminolysis

To an ester solution (1 equiv) in methanol (0.15–0.19 M) were added aq. hydroxylamine (50% *w*/*w* in water, 0.15–0.19 M) and KCN (0.1 equiv). The mixture was stirred overnight. Then, the solvents were removed at RT under reduced pressure and the residue was purified by flash chromatography to afford the desired hydroxamate.

#### General Procedure F2: Copper-Catalyzed Click Reaction

Azide (1 equiv) and alkyne (1.0 equiv) were dissolved in *N*,*N*-dimethylformamide or dioxane (0.05–0.08 M) before the addition of copper sulfate pentahydrate (0.2 equiv) in water (0.1–0.15 M) and sodium ascorbate (0.5 equiv). The resulting mixture was stirred at room temperature overnight. The mixture was then diluted in water and extracted with ethyl acetate. The combined organic layers were dried over MgSO_4_, filtered, and concentrated under reduced pressure. The residue was finally purified by flash chromatography affording the desired 1,4-triazole.

#### General Procedure G2: Alkyne Formation

To a phenol solution (1 equiv) in *N*,*N*-dimethylformamide (0.2–0.5 M) were added K_2_CO_3_ (2 equiv) and propargyl bromide (1.1 equiv). The resulting solution was heated to 60 °C and stirred overnight. The mixture was then diluted in water and extracted three times with ethyl acetate. The organic layers were combined, and solvents were evaporated under reduced pressure affording the desired alkyne.

##### 2-Ethoxycarbonyl-4-methyl-pentanoic Acid (**22a**)

Compound **22a** was synthesized according to the general procedure A2, using diethyl isopropylmalonate (4000 mg, 18.5 mmol) and sodium hydroxide (888 mg, 22.2 mmol) in EtOH/H_2_O (40 mL, 32:8 *v*/*v*) overnight. Compound **22a** was obtained as a colorless oil (2400 mg, 68%) and was used in the next step without further purification. ^1^H NMR (500 MHz, DMSO-*d*_6_) δ: 12.80 (br s, 1H), 4.10 (q, *J* = 7.1 Hz, 2H), 3.36–3.33 (m, 1H), 1.65–1.59 (m, 2H), 1.49 (sep, *J* = 6.7 Hz, 1H), 1.17 (t, *J* = 7.1 Hz, 3H), 0.86 (d, *J* = 6.6 Hz, 3H), 0.86 (d, *J* = 6.6 Hz, 3H). ^13^C NMR (126 MHz, DMSO-*d*_6_) δ: 170.6, 169.6, 60.7, 49.8, 37.2, 25.7, 22.2, 22.1, 14.0. MS (ESI^+^): *m*/*z* = 189 [*M* + H]^+^.
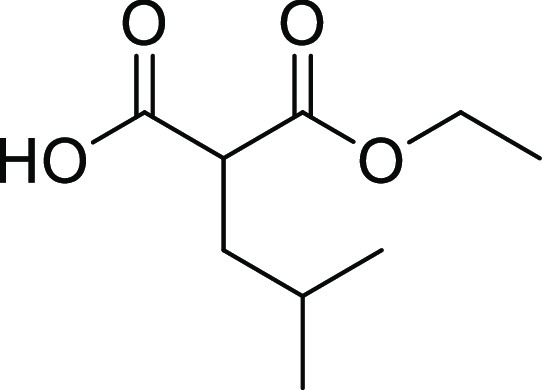


##### 2-Benzyl-3-ethoxy-3-oxo-propanoic Acid (**22b**)

Compound **22b** was synthesized according to the general procedure A2, using diethyl benzylmalonate (1070 mg, 4.3 mmol) and sodium hydroxide (205 mg, 5.1 mmol) in EtOH/H_2_O (10 mL, 8:2 *v/v*) overnight. Compound **22b** was obtained as a colorless oil (694 mg, 72%) and was used in the next step without further purification. ^1^H NMR (500 MHz, CDCl_3_) δ: 10.23 (br s, 1H), 7.36–7.31 (m, 2H), 7.30–7.26 (m, 3H), 4.22 (q, *J* = 7.1 Hz, 2H), 3.76 (t, *J* = 7.7 Hz, 1H), 3.30 (dd, *J* = 7.7 and 2.8 Hz, 2H), 1.26 (t, *J* = 7.1 Hz, 3H). ^13^C NMR (126 MHz, CDCl_3_) δ: 174.3, 168.9, 137.4, 128.9 (2C), 128.7 (2C), 127.1, 62.0, 53.6, 34.9, 14.1. MS (ESI^+^): *m*/*z* = no ionization [*M* + H]^+^.
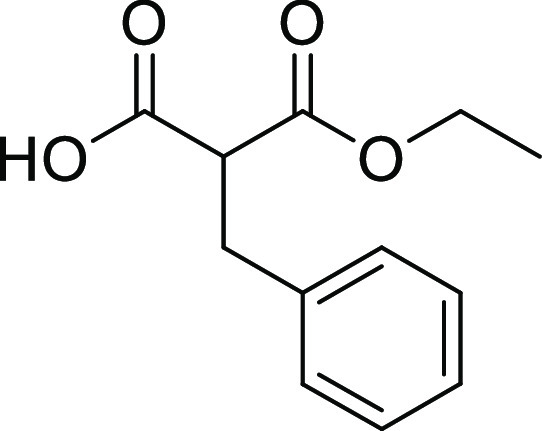


##### Ethyl 2-[2-(tert-Butoxycarbonylamino)ethylcarbamoyl]-4-methyl-pentanoate (**23a**)

Compound **23a** was synthesized according to the general procedure B2, using carboxylic acid **22a** (2000 mg, 10.6 mmol), *tert*-butyl *N*-(2-aminoethyl)carbamate (1876 mg, 11.6 mmol), HOBt (162.8 mg, 1.0 mmol), EDC.HCl (3060 mg, 16.0 mmol), and diisopropylethylamine (5560 μL, 31.8 mmol) in CH_2_Cl_2_ (50 mL) overnight. The crude product was purified by flash chromatography on silica gel (cHex to cHex/EtOAc 6:4) affording compound **23a** as a yellowish solid (1868 mg, 53%). ^1^H NMR (DMSO-*d*_6_) δ: 8.13 (t, *J* = 5.3 Hz, 1H), 6.71 (t, *J* = 5.4 Hz, 1H), 4.10–4.01 (m, 2H), 3.29–3.26 (m, 1H), 3.15–3.01 (m, 2H), 2.98–2.95 (m, 2H), 1.64–1.53 (m, 2H), 1.45–1.40 (m, 1H), 1.37 (s, 9H), 1.15 (t, *J* = 7.1 Hz, 3H), 0.85 (d, *J* = 6.6 Hz, 3H), 0.84 (d, *J* = 6.6 Hz, 3H). ^13^C NMR (DMSO-*d*_6_) δ: 170.0, 168.4, 155.6, 77.7, 60.4, 50.2, 39.52, 38.8, 37.4, 28.2 (3C), 25.5, 22.6, 22.0, 14.0. MS (ESI^+^): *m*/*z* = 331 [*M* + H]^+^, 275 [M–tBu+H]^+^, 231 [M–Boc+H]^+^.
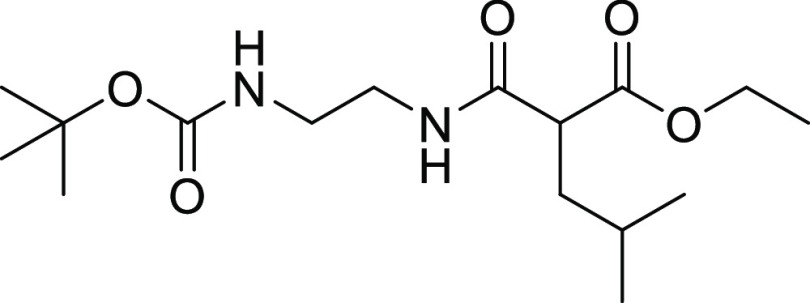


##### 2-[(2-Ethoxycarbonyl-4-methyl-pentanoyl)amino]ethylammonium Chloride (**24a**)

Compound **24a** was synthesized according to the general procedure C2, using the Boc-protected intermediate **23a** (1855 mg, 5.6 mmol) and 4 N HCl in dioxane (20 mL) in a mixture of CH_2_Cl_2_/EtOH (40 mL, 20:20 *v/v*) overnight. Compound **24a** was obtained as a colorless oil (1500 mg, quant. yield) and was used in the next step without further purification. ^1^H NMR (300 MHz, DMSO-*d*_6_) δ: 8.53 (t, *J* = 5.5 Hz, 1H), 8.13 (br s, 3H), 4.09 (dd, *J* = 7.2 and 1.8 Hz, 1H), 4.04 (dd, *J* = 7.2 and 1.8 Hz, 1H), 3.35–3.28 (m, 3H), 2.89–2.77 (m, 2H), 1.65–1.59 (m, 2H), 1.44 (sep, *J* = 6.7 Hz, 1H), 1.16 (t, *J* = 7.1 Hz, 3H), 0.86 (d, *J* = 6.5 Hz, 3H), 0.85 (d, *J* = 6.5 Hz, 3H). ^13^C NMR (75 MHz, DMSO-*d*_6_) δ: 169.9, 168.8, 60.5, 50.2, 38.2, 37.3, 36.6, 25.6, 22.4, 22.2, 14.0. MS (ESI^+^): *m*/*z* = 231 [*M* + H]^+^.
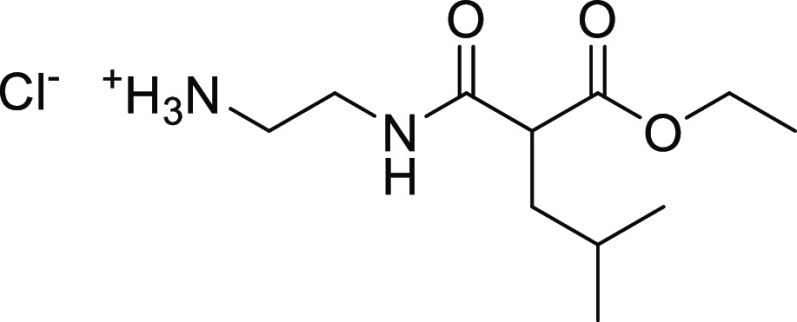


##### Ethyl 2-(2-Azidoethylcarbamoyl)-4-methyl-pentanoate (**25a**)

Compound **25a** was synthesized according to the general procedure D2, using amine **24a** (1500 mg, 5.6 mmol), ZnCl_2_ (46 mg, 0.34 mmol), K_2_CO_3_ (778 mg, 5.6 mmol), diisopropylethylamine (3420 μL, 19.6 mmol), and diazo transfer reagent (1415 mg, 6.7 mmol) in EtOH (15 mL). The mixture was stirred at room temperature overnight. The crude product was purified by flash chromatography on silica gel (cHex/EtOAc 9:1 to 6:4) affording compound **25a** as a colorless oil (1430 mg, quant. yield). ^1^H NMR (500 MHz, CDCl_3_) δ: 6.85–6.81 (m, 1H), 4.23–4.16 (m, 2H), 3.44–3.42 (m, 4H), 3.33–3.30 (m, 1H), 1.83–1.71 (m, 2H), 1.57 (sep, *J* = 6.7 Hz, 1H), 1.28 (t, *J* = 7.1 Hz, 3H), 0.93 (d, *J* = 6.6 Hz, 3H), 0.92 (d, *J* = 6.6 Hz, 3H). ^13^C NMR (126 MHz, CDCl_3_) δ: 172.6, 169.2, 61.7, 51.7, 50.9, 40.4, 39.3, 26.5, 22.6, 22.1, 14.2. MS (ESI^+^): *m*/*z* = 257 [*M* + H]^+^.
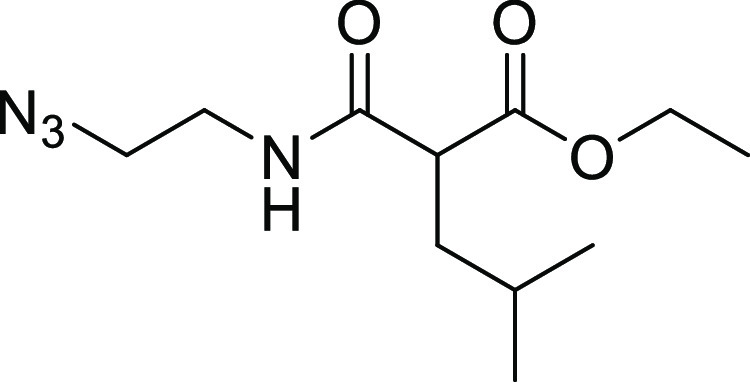


##### *N*-(2-Azidoethyl)-2-(hydroxycarbamoyl)-4-methyl-pentanamide (**26a**)

Compound **26a** was synthesized according to the general procedure E2, using ester **25a** (600 mg, 2.3 mmol), KCN (30.5 mg, 0.47 mmol), and aq. NH_2_OH (12 mL, 50% *w*/*w* in water) in MeOH (12 mL) overnight. The crude product was purified by flash chromatography on silica gel (CH_2_Cl_2_ to CH_2_Cl_2_/MeOH 9:1) affording compound **26a** as a white solid (366 mg, 65%). ^1^H NMR (500 MHz, DMSO-*d*_6_) δ: 10.50 (s, 1H), 8.93 (s, 1H), 7.85 (t, *J* = 5.1 Hz, 1H), 3.36–3.34 (m, 2H), 3.25–3.22 (m, 2H), 3.00–2.97 (m, 1H), 1.64–1.58 (m, 1H), 1.56–1.51 (m, 1H), 1.43 (sep, J = 6.6 Hz, 1H), 0.84 (d, *J* = 6.4 Hz, 6H). ^13^C NMR (126 MHz, DMSO-*d*_6_) δ: 169.4, 166.6, 49.9, 49.0, 38.6, 38.4, 25.6, 22.6, 22.1. MS (ESI^+^): *m*/*z* = 243 [*M* + H]^+^.
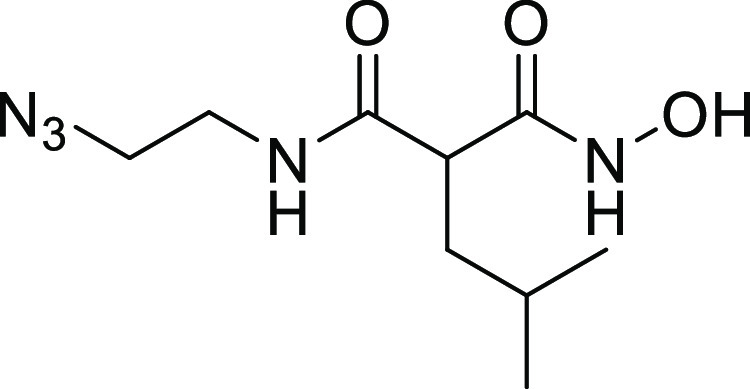


##### *N*-(2-Prop-2-ynoxyphenyl)acetamide (**34a**)

Compound **34a** was synthesized according to the general procedure G2, using *N*-(2-hydroxyphenyl)acetamide (150 mg, 0.99 mmol), K_2_CO_3_ (274 mg, 1.98 mmol), and propargyl bromide (103 μL, 1.09 mmol) in DMF (5 mL). The resulting solution was heated to 60 °C and stirred overnight. The mixture was then diluted in water and extracted three times with ethyl acetate. Organic layers were combined, and solvents were evaporated under reduced pressure affording compound **34a** as a brown solid (182 mg, 96%). ^1^H NMR (500 MHz, DMSO-*d*_6_) δ: 9.13 (s, 1H), 7.91 (d, *J* = 7.5 Hz, 1H), 7.12–7.11 (m, 1H), 7.06 (t, *J* = 7.5 Hz, 1H), 6.93 (t, *J* = 7.5 Hz, 1H), 4.86 (d, *J* = 2.0 Hz, 2H), 3.60 (t, *J* = 2.2 Hz, 1H), 2.08 (s, 3H). ^13^C NMR (126 MHz, DMSO-*d*_6_) δ: 168.5, 147.6, 127.9, 124.1, 122.6, 121.0, 112.9, 79.2, 78.6, 56.0, 23.8. MS (ESI^+^): *m*/*z* = 190 [*M* + H]^+^.
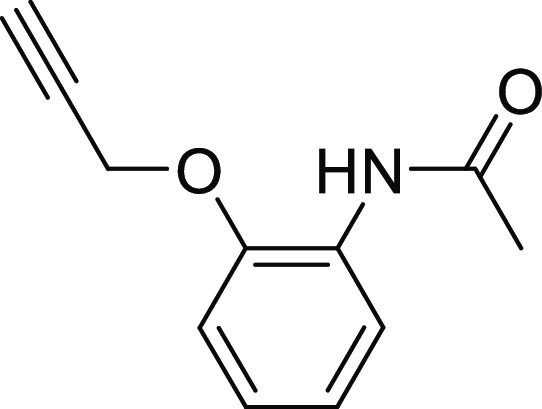


##### *N*-[2-[4-[(2-Acetamidophenoxy)methyl]triazol-1-yl]ethyl]-2-(hydroxycarbamoyl)-4-methyl-pentanamide (**27**)

Compound **27** was synthesized according to the general procedure F2, using azide **26a** (88 mg, 0.36 mmol), alkyne **34a** (69 mg, 0.36 mmol), copper (II) sulfate pentahydrate (18.1 mg, 0.07 mmol), and sodium ascorbate (35.8 mg, 0.18 mmol) in DMF (5.5 mL) and H_2_O (4.5 mL). The mixture was stirred at room temperature overnight. The crude product was purified by preparative HPLC (H_2_O + 0.05% FA/CAN + 0.05% FA 95:5 to 5:95) affording **27** as a white solid after lyophilization (82 mg, 51%). ^1^H NMR (500 MHz, DMSO-*d*_6_) δ: 10.52 (s, 1H), 9.01 (s, 1H), 8.95 (s, 1H), 8.15 (s, 1H), 7.91–7.90 (m, 1H), 7.84 (t, *J* = 5.7 Hz, 1H), 7.24–7.23 (m, 1H), 7.07–7.04 (m, 1H), 6.92–6.89 (m, 1H), 5.20 (s, 2H), 4.44 (t, *J* = 6.1 Hz, 2H), 3.52–3.50 (m, 2H), 2.94 (t, *J* = 7.7 Hz, 1H), 2.07 (s, 3H), 1.57–1.46 (m, 2H), 1.35 (sep, *J* = 6.6 Hz, 1H), 0.80 (d, *J* = 6.6 Hz, 3H), 0.79 (d, *J* = 6.6 Hz, 3H). ^13^C NMR (126 MHz, DMSO-*d*_6_) δ: 169.4, 168.4, 166.6, 148.5, 142.7, 127.9, 124.8, 124.2, 122.3, 120.8, 113.2, 62.3, 49.0, 48.6, 39.0, 38.4, 25.5, 23.9, 22.4, 22.1. Purity: 100%. HRMS–ESI^+^ (*m*/*z*): calculated for C_20_H_29_N_6_O_5_ [*M* + H]^+^ 433.2199, found 433.2190.
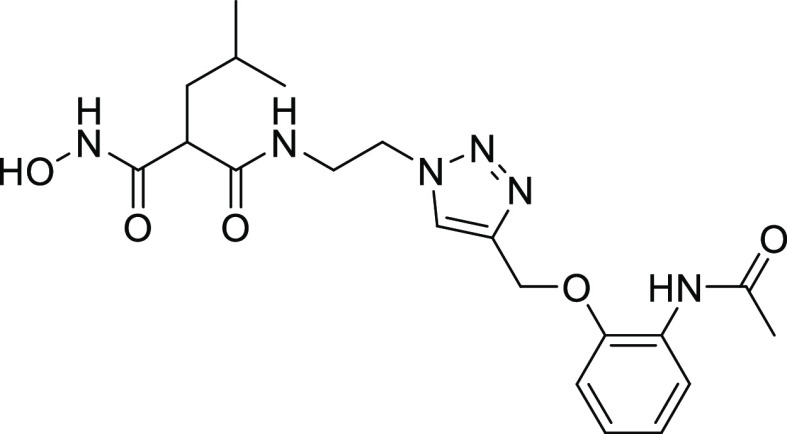


##### *N*-[2-[4-[(3-Acetamidophenoxy)methyl]triazol-1-yl]ethyl]-2-(hydroxycarbamoyl)-4-methyl-pentanamide (**28**)

Compound **28** was synthesized according to the general procedure F2, using azide **26a** (55 mg, 0.23 mmol), alkyne **34b** (43 mg, 0.23 mmol), copper (II) sulfate pentahydrate (11.3 mg, 0.05 mmol), and sodium ascorbate (22.4 mg, 0.11 mmol) in DMF (3 mL) and H_2_O (2 mL). The mixture was stirred at RT overnight. The crude product was purified by flash chromatography on silica gel (CH_2_Cl_2_ to CH_2_Cl_2_/MeOH 9:1) affording **28** as a white solid after lyophilization (22 mg, 22%). ^1^H NMR (500 MHz, DMSO-*d*_6_) δ: 10.50 (s, 1H), 9.91 (s, 1H), 8.94 (s, 1H), 8.13 (s, 1H), 7.85 (t, *J* = 5.3 Hz, 1H), 7.34 (s, 1H), 7.21–7.18 (m, 1H), 7.12–7.10 (m, 1H), 6.73–6.72 (m, 1H), 5.07 (s, 2H), 4.43 (t, *J* = 5.2 Hz, 2H), 3.53–3.49 (m, 2H), 2.94 (t, *J* = 7.6 Hz, 1H), 2.03 (s, 3H), 1.57–1.47 (m, 2H), 1.38–1.30 (m, 1H), 0.81 (d, *J* = 6.2 Hz, 3H), 0.80 (d, J = 6.2 Hz, 3H). ^13^C NMR (126 MHz, DMSO-*d*_6_) δ: 169.4, 168.3, 166.6, 158.3, 142.5, 140.5, 129.5, 124.7, 111.7, 108.9, 105.8, 61.0, 48.9, 48.6, 39.0, 38.3, 25.5, 24.1, 22.4, 22.2. Purity: 97%. HRMS–ESI^+^ (*m*/*z*): calculated for C_20_H_29_N_6_O_5_ [*M* + H]^+^ 433.2199, found 433.2190.
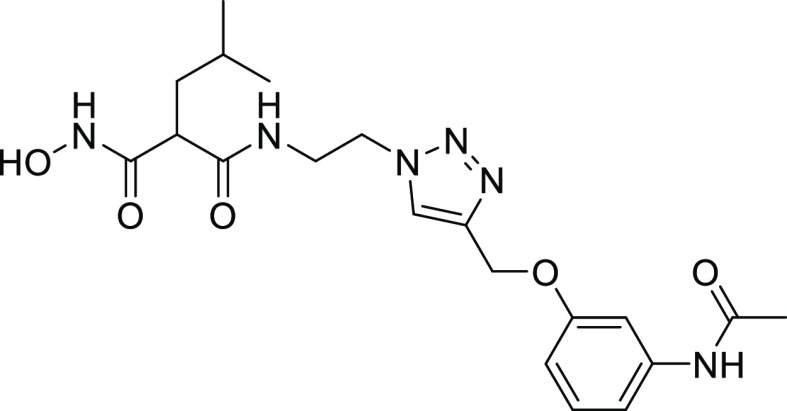


##### *N*-[2-[4-[(4-Acetamidophenoxy)methyl]triazol-1-yl]ethyl]-2-(hydroxycarbamoyl)-4-methyl-pentanamide (**29**)

Compound **29** was synthesized according to the general procedure F2, using azide **26a** (55 mg, 0.23 mmol), alkyne **34c** (43 mg, 0.23 mmol), copper (II) sulfate pentahydrate (11.3 mg, 0.05 mmol), and sodium ascorbate (22.4 mg, 0.11 mmol) in DMF (3 mL) and H_2_O (2 mL). The mixture was stirred at RT overnight. The crude product was purified by flash chromatography on silica gel (CH_2_Cl_2_ to CH_2_Cl_2_/MeOH 9:1) affording **29** as a white solid after lyophilization (43 mg, 43%). ^1^H NMR (500 MHz, DMSO-*d*_6_) δ: 10.52 (s, 1H), 9.81 (s, 1H), 8.97 (s, 1H), 8.13 (s, 1H), 7.87 (t, *J* = 5.5 Hz, 1H), 7.48 (d, *J* = 8.9 Hz, 2H), 6.96 (d, *J* = 8.9 Hz, 2H), 5.06 (s, 2H), 4.43 (t, *J* = 5.7 Hz, 2H), 3.52–3.48 (m, 2H), 2.94 (t, *J* = 7.6 Hz, 1H), 2.00 (s, 3H), 1.56–1.46 (m, 2H), 1.38–1.31 (m, 1H), 0.81 (d, *J* = 6.3 Hz, 3H), 0.80 (d, *J* = 6.3 Hz, 3H). ^13^C NMR (126 MHz, DMSO-*d*_6_) δ: 169.5, 167.8, 166.7, 153.9, 142.7, 132.9, 124.8, 120.5 (2C), 114.7 (2C), 61.3, 48.9, 48.7, 39.0, 38.4, 25.5, 23.9, 22.4, 22.2. Purity: 98%. HRMS–ESI^+^ (*m*/*z*): calculated for C_20_H_29_N_6_O_5_ [*M* + H]^+^ 433.2199, found 433.2166.
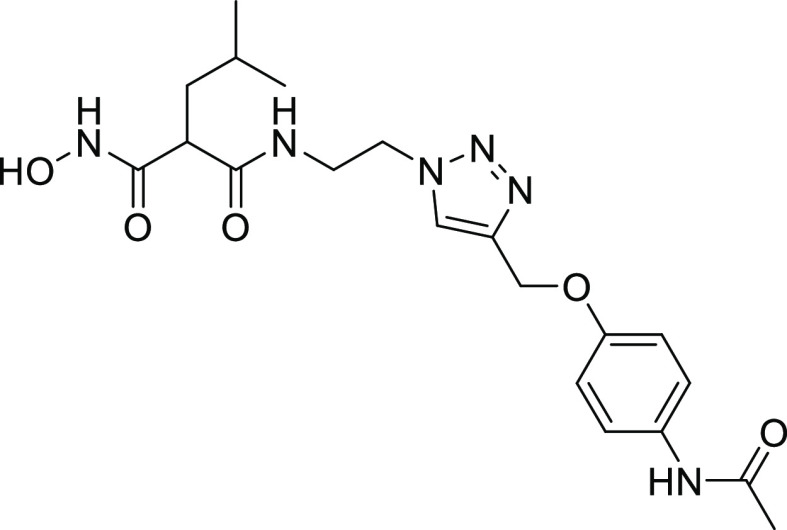


##### *N*-[2-[4-[(2-Fluorophenoxy)methyl]triazol-1-yl]ethyl]-2-(hydroxycarbamoyl)-4-methyl-pentanamide (**30**)

Compound **30** was synthesized according to the general procedure F2, using azide **26a** (70 mg, 0.29 mmol), alkyne **34d** (37 μL, 0.29 mmol), copper (II) sulfate pentahydrate (14.4 mg, 0.06 mmol), and sodium ascorbate (28.5 mg, 0.14 mmol) in dioxane (4 mL) and H_2_O (3 mL). The mixture was stirred at RT overnight. The crude product was purified by flash chromatography on silica gel (CH_2_Cl_2_ to CH_2_Cl_2_/MeOH 9:1) affording **30** as a white solid after lyophilization (74 mg, 65%). ^1^H NMR (500 MHz, DMSO-*d*_6_) δ: 10.52 (s, 1H), 8.97 (s, 1H), 8.17 (s, 1H), 7.86 (t, *J* = 5.5 Hz, 1H), 7.38–7.35 (m, 1H), 7.23–7.19 (m, 1H), 7.16–7.13 (m, 1H), 6.97–6.93 (m, 1H), 5.19 (s, 2H), 4.44 (t, *J* = 5.2 Hz, 2H), 3.53–3.49 (m, 2H), 2.93 (t, *J* = 7.6 Hz, 1H), 1.56–1.45 (m, 2H), 1.37–1.29 (m, 1H), 0.80 (d, *J* = 6.4 Hz, 3H), 0.79 (d, *J* = 6.4 Hz, 3H). ^13^C NMR (126 MHz, DMSO-*d*_6_) δ: 169.5, 166.6, 151.8 (d, *J* = 243.5 Hz), 146.0 (d, *J* = 10.6 Hz), 142.1, 125.2, 124.9 (d, *J* = 3.6 Hz), 121.4 (d, *J* = 6.0 Hz), 116.1 (d, *J* = 17.5 Hz), 115.4, 61.9, 48.9, 48.7, 39.0, 38.4, 25.5, 22.4, 22.2. ^19^F NMR (471 MHz, DMSO-*d*_6_) δ: −134.7. Purity: 100%. HRMS–ESI^+^ (*m*/*z*): calculated for C_18_H_25_FN_5_O_4_ [*M* + H]^+^ 394.1890, found 394.1862.
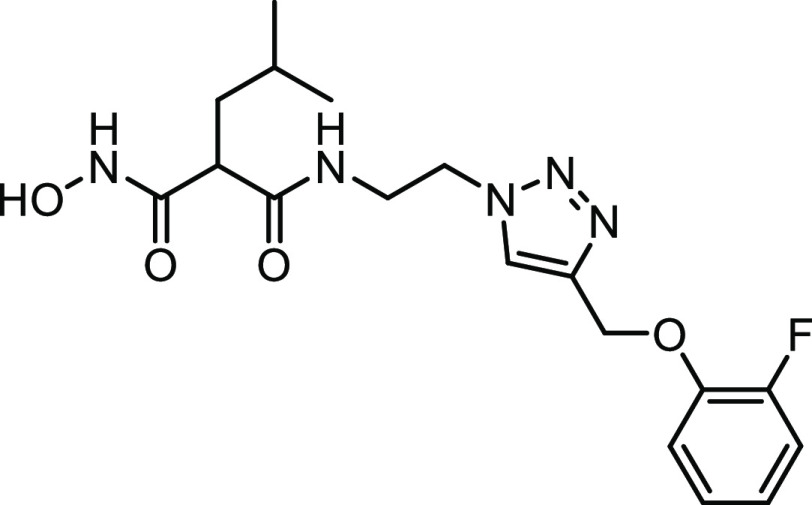


##### 2-(Hydroxycarbamoyl)-4-methyl-*N*-[2-[4-(phenoxymethyl)triazol-1-yl]ethyl]pentanamide (**31**)

Compound **31** was synthesized according to the general procedure F2, using azide **26a** (70 mg, 0.29 mmol), prop-2-ynoxybenzene (37 μL, 0.29 mmol), copper (II) sulfate pentahydrate (14.4 mg, 0.06 mmol), and sodium ascorbate (28.5 mg, 0.14 mmol) in dioxane (4 mL) and H_2_O (3 mL). The mixture was stirred at RT overnight. The crude product was purified by flash chromatography on silica gel (CH_2_Cl_2_ to CH_2_Cl_2_/MeOH 9:1) affording **31** as a white solid after lyophilization (56 mg, 51%). ^1^H NMR (500 MHz, DMSO-*d*_6_) δ: 10.51 (br s, 1H), 8.97 (s, 1H), 8.14 (s, 1H), 7.87 (t, *J* = 5.5 Hz, 1H), 7.32–7.28 (m, 2H), 7.04–7.02 (m, 2H), 6.96–6.93 (m, 2H), 5.10 (s, 2H), 4.43 (t, *J* = 5.1 Hz, 2H), 3.52–3.49 (m, 2H), 2.94 (t, *J* = 7.6 Hz, 1H), 1.57–1.46 (m, 2H), 1.38–1.30 (m, 1H), 0.81 (d, *J* = 6.4 Hz, 3H), 0.80 (d, *J* = 6.4 Hz, 3H). ^13^C NMR (126 MHz, DMSO-*d*_6_) δ: 169.5, 166.6, 158.1, 142.6, 129.6 (2C), 124.9, 120.9, 114.6 (2C), 61.0, 48.9, 48.7, 39.0, 38.4, 25.5, 22.4, 22.2. Purity: 100%. HRMS–ESI^+^ (*m*/*z*): calculated for C_18_H_26_N_5_O_4_ [*M* + H]^+^ 376.1985, found 376.1956.
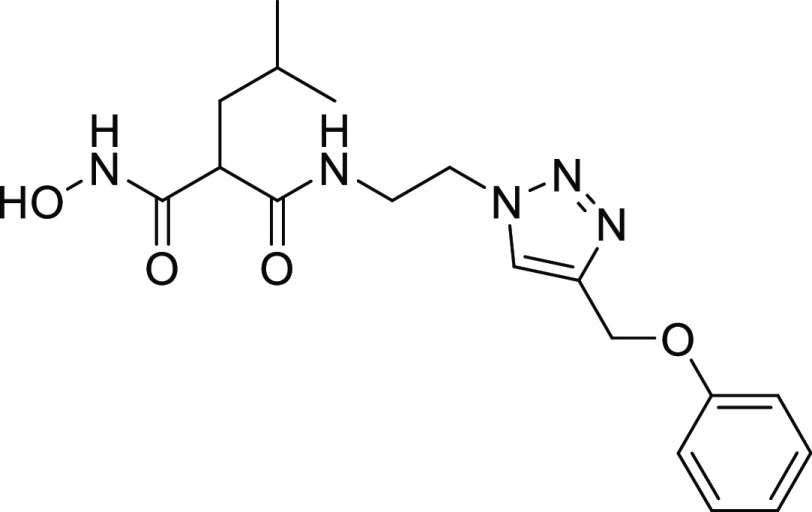


##### 2-(Hydroxycarbamoyl)-4-methyl-*N*-[2-[4-(phenylsulfanylmethyl)triazol-1 yl]ethyl]pentanamide (**32**)

Compound **32** was synthesized according to the general procedure F2, using azide **26a** (70 mg, 0.29 mmol), prop-2-ynylsulfanylbenzene (43 mg, 0.29 mmol), copper (II) sulfate pentahydrate (14.4 mg, 0.06 mmol), and sodium ascorbate (28.5 mg, 0.14 mmol) in dioxane (4 mL) and H_2_O (3 mL). The mixture was stirred at RT overnight. The crude product was purified by flash chromatography on silica gel (CH_2_Cl_2_ to CH_2_Cl_2_/MeOH 95:5) affording **27** as a light-green solid after lyophilization (64 mg, 56%). ^1^H NMR (500 MHz, DMSO-*d*_6_) δ: 10.49 (s, 1H), 8.94 (s, 1H), 7.89 (s, 1H), 7.80 (t, *J* = 5.9 Hz, 1H), 7.38–7.36 (m, 2H), 7.33–7.30 (m, 2H), 7.20–7.17 (m, 1H), 4.38–4.36 (m, 2H), 4.26 (s, 2H), 3.48–3.44 (m, 2H), 2.93 (t, *J* = 7.6 Hz, 1H), 1.57–1.45 (m, 2H), 1.40–1.32 (m, 1H), 0.82–0.81 (m, 6H). ^13^C NMR (126 MHz, DMSO-*d*_6_) δ: 169.4, 166.6, 143.2, 136.1, 129.0 (2C), 128.0 (2C), 125.8, 123.5, 48.9, 48.5, 38.9, 38.4, 27.2, 25.5, 22.4, 22.1. Purity: 100%. HRMS–ESI^+^ (*m*/*z*): calculated for C_18_H_26_N_5_O_3_S [*M* + H]^+^ 392.1756, found 392.1727.
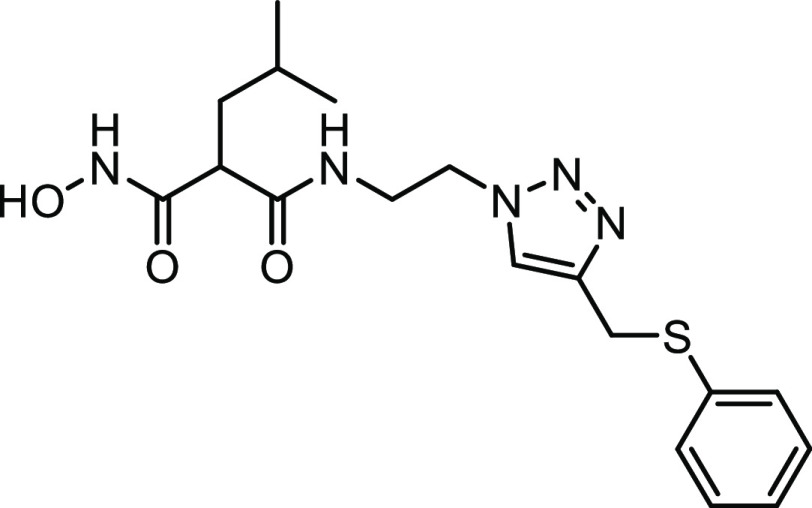


##### *N*-[2-[4-[(2-Acetamidophenoxy)methyl]triazol-1-yl]ethyl]-2-benzyl-3-(hydroxyamino)-3-oxo-propanamide (**33**)

Compound **33** was synthesized according to the general procedure F2, using azide **21b** (45 mg, 0.1 mmol), alkyne **29a** (43 mg, 0.1 mmol), copper (II) sulfate pentahydrate (5 mg, 0.02 mmol), and sodium ascorbate (9.9 mg, 0.05 mmol) in dioxane (2 mL) and H_2_O (1 mL). The mixture was stirred at RT overnight. The crude product was purified by flash chromatography on silica gel (CH_2_Cl_2_ to CH_2_Cl_2_/MeOH: 9/1) affording **28** as a white solid after lyophilization (7 mg, 15%). ^1^H NMR (500 MHz, DMSO-*d*_6_) δ: 10.45 (s, 1H), 9.00–8.93 (m, 2H), 8.04–7.91 (m, 3H), 7.24–6.91 (m, 8H), 5.19 (s, 2H), 4.39 (br s, 2H), 3.49 (br s, 2H), 3.21–3.17 (m, 1H), 2.99–2.96 (br s, 2H), 2.06 (s, 3H). ^13^C NMR (126 MHz, DMSO-*d*_6_) δ: 168.7, 168.4, 165.6, 148.5, 142.7, 138.8, 128.7 (2C), 128.2 (2C), 127.9, 126.2, 124.9, 124.2, 122.3, 120.8, 113.2, 62.2, 52.2, 48.6, 39.0, 34.6. Purity: 96%. HRMS–ESI^+^ (*m*/*z*): calculated for C_23_H_27_N_6_O_5_ [*M* + H]^+^ 467.2047, found 467.2013.
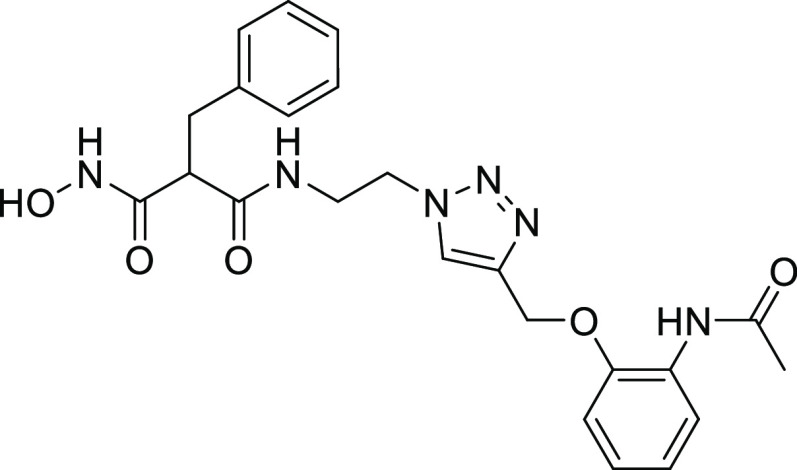


### Expression and Purification of ColQ1

ColQ1 was produced and purified in its complete length and collagenase unit from *B. cereus* strain Q1 (Uniprot: B9J3S4; Tyr94-Gly765), as previously described.^20^

### *In Vitro* FRET-Based Proteolytic Assay (ColQ1, ColA, ColG, and ColH)

For all targets, the percent of inhibition and IC_50_ measurements were carried out as previously described.^20,25,26^ Experiments were performed in triplicate, and the results are provided as means ± standard deviation. For the determination of the inhibition constant (*K*_i_), similar assay conditions were chosen. However, nominal final enzyme concentrations of 1 nM ColQ1-CU, 10 nM ColH-PD, 35 nM ColA, and 60 nM ColG were used and the reactions were monitored for 2 min 24 s. Regression analysis was performed using GraphPad Prism v 9.0.0 (GraphPad Software, San Diego, CA). The experiments were performed under first-order conditions ([S_0_] ≪ *K*_M_), which resulted in an approximation of the *K*_i_^app^ to the true inhibition constant (*K_i_*); therefore, the results are reported as *K_i_* values.

### Reversibility Assays by Rapid Dilution

The recovery of enzymatic activity after a rapid large dilution was performed following Copeland, 2013.^32^ In short, ColA, ColG, ColH, and ColQ1 were incubated for 30 min at 100-fold the concentration required for the activity assay (*i.e*., 4.375, 7.500, 1.250, and 0.125 μM, respectively) with a concentration of inhibitor equivalent to 10-fold the IC_50_. The mixture was then diluted 100-fold into the reaction buffer. The reaction was immediately initiated by the addition of the quenched-fluorescent substrate FS1-1 at a final concentration of 2 μM. The reaction was monitored for 2 min (excitation: 328 nm, emission: 392 nm) at 25 °C in an Infinite M200 plate reader (Tecan, Grödig, Austria).

### Mass Spectrometric Analysis of Collagenase–Ligand Interactions

The collagenase subunit of ColQ1 incubated with either Ilomastat, **13**, or no inhibitor was investigated by high-performance liquid chromatography coupled to mass spectrometry (HPLC-MS). Prior to HPLC-MS measurements, ColQ1-CU samples were buffer-exchanged to 150 mmol·L^–1^ ammonium acetate on 3 kDa molecular weight cutoff centrifugal filters (Amicon Ultra 0.5 mL, Merck, Darmstadt, Germany) and reconstituted to a concentration of 0.5 mg·mL^–1^. ColQ1-CU samples were separated on a capillary HPLC instrument (UltiMate U3000 RSLC, Thermo Fisher Scientific, Germering, Germany) equipped with a Supelco Discovery C18 column (150 × 2.1 mm^2^ i.d., 3 μm particle size, 300 Å pore size; Sigma-Aldrich, Vienna, Austria). The column was operated at a flow rate of 150 μL·min^–1^ and a column oven temperature of 70 °C. Using in-line split-loop mode, 7 microliters of sample [0.5 mg mL^–1^] was injected in triplicate. The separation was carried out employing a gradient of mobile phase A (H_2_O + 0.10% formic acid (98–100%; Sigma-Aldrich)) and B (acetonitrile (HiPerSolv; VWR chemicals, Radnor, PA) + 0.10% formic acid) as follows: 25.0% B for 5.0 min, 25.0–90.0% B in 20 min, 95.0% B for 5.0 min, and 25.0% B for 10 min. Water (H_2_O) was purified by a MilliQ Integral 3 system (Merck Millipore, Burlington, MA). Mass spectrometric data was acquired on a Thermo Scientific QExactive benchtop quadrupole-Orbitrap mass spectrometer employing an Ion Max source with a heated electrospray ionization probe (both from Thermo Fisher Scientific, Bremen, Germany) and an MXT715-000—MX Series II Switching Valve (IDEX Health & Science, LLC, Oak Harbor, WA), as described earlier.^45^ MS settings were optimized for the ColQ1-CU protein: the resolution was set to 17,500 at *m*/*z* 200 in a mass range of *m/z* 500–2700, the source heater temperature was lowered to 200 °C, and the in-source collision-induced dissociation was increased to 30.0 eV. Data was acquired between minutes 5 and 35. Mass spectra were deconvoluted employing the ReSpect algorithm implemented in the Chromeleon Chromatography Data System, version 7.2.10 (Thermo Fisher Scientific, Waltham, MA).

### Selectivity toward Human MMPs

The MMP inhibition assay (Sigma-Aldrich, Saint Louise, MO) was performed as previously reported and in accordance with the manufacturer’s instructions. The fluorescence signals were measured in a CLARIOstar plate reader (BMG LABTECH, Ortenberg, Germany) at a concentration of 100 μM.

### Compound Toxicity

Cytotoxicity assays on HepG2, HEK293, NHDF, and MDCK II cells were carried out as described previously.^25^

### *In Vitro* Collagen Cleavage Assay

The experiments were done as described before.^20,28^ Briefly, in a buffer containing 250 mM HEPES, 150 mM NaCl, 5 mM CaCl_2_, 5 μM ZnCl_2_, pH 7.5, 1 mg/mL acid-soluble type I collagen from bovine tail (Thermo Fischer Scientific) was incubated with 50 ng full-length ColQ1. Compounds were evaluated at various concentrations and incubated together with collagen and ColQ1 at 25 °C for 3 h. After stopping the reaction with 50 mM EDTA, the mixture was loaded on 12% SDS-PAGE gel and stained with colloidal coomassie G-250 dye.^46^ Two separate experiments were carried out for each compound.

### *In Vitro* NHDF Infection Model

NHDF cells (1 × 10^5^, Promo Cell C-12302) per well were seeded in 24-well plates (Greiner) with DMEM medium (Gibco) containing 10% (*v*/*v*) fetal calf serum (FCS, Gibco) and 1% (*v*/*v*) glutamine (Gibco). Prior to the treatment, the cells were cultured at 37 °C for 24 h with 5% CO_2_. In brain heart infusion (BHI) medium, *B. cereus* AH187 bacteria were cultivated to a mid-exponential phase. The culture was centrifuged at 4000*g* for 7 min at RT before being rinsed and diluted in PBS. Then, *B. cereus* suspension was added to the cells to give an MOI (multiplicity of infection) of 0.03. Before the infection, cells were starved for 1 h with DMEM containing only 1% (*v/v*) glutamine and no FCS. The cells were infected with *B. cereus* for 1, 2, 4, and 6 h to examine the kinetics of collagenase release. The DMEM supernatant was collected and harvested cleared by centrifugation at 4000*g* at 4 °C for 10 min. After the cells were washed, they were lysed using a lysis buffer (20 mM tris pH 7.5, 1 mM EDTA, 100 mM NaCl, 1% Triton 100, 0.5% Na-deoxycholate, 0.1% SDS, 1 × PIT, 1 mM Na_3_VO_4,_ 1 mM Na-molybdate, 20 mM NaF, 20 mM β-glycerophosphate). Cell debris was removed by centrifugation with 12,000*g* at 4 °C for 10 min. The DMEM supernatants and cell lysates were stored at −80 °C for further investigation. The cell morphology was monitored with a light microscope (Olympus) using a 20X objective. The kinetic study was performed in three independent experiments. To study the behavior of ColQ1 inhibitors in this model, the experiment was done as stated above with a few changes; the compounds were added to the NHDF cells along with the bacterial suspension, and all were incubated at 37 °C for 5 h and 5% CO_2_. Two controls were considered in each experiment, uninfected cells, and infected cells without inhibitor. Each experiment was repeated three times in total.

### Nonreducing Sodium Dodecyl Sulfate Polyacrylamide Gel Electrophoresis (SDS-PAGE) and Zymography

To perform the zymography, we collected supernatants from the NHDF infection experiments and mixed them with 1% nonreducing loading buffer. They were then electrophoretically separated after loading onto 10% SDS-PAGE gels containing 0.1% gelatin (Roth, Karlsruhe, Germany). Following separation, the gel was incubated at 4 °C for 2 × 30 min with gentle agitation in a renaturation buffer (50 mM HEPES pH 7.5, 200 mM NaCl, 10 mM CaCl_2_, 10 μM ZnCl_2_, 2.5% Triton X-100). The gel was then treated in a developing buffer (50 mM HEPES pH 7.5, 200 mM NaCl, 10 mM CaCl_2_, 10 μM ZnCl_2_, 0.02% Brij-35) at 37 °C overnight. By staining the gel with 0.1% Coomassie brilliant blue R-250 dye overnight, transparent bands of gelatinolytic activity could be seen. The ChemiDoc XRS+ imaging system (Biorad) was used to scan the gels, and image analysis was performed with Image lab software (Li-Cor Biosciences).

### Reducing Sodium Dodecyl Sulfate Polyacrylamide Gel Electrophoresis

The cell lysate was placed onto a 12% SDS-PAGE gel with similar total protein content and stained overnight with Coomassie brilliant blue G-250. The gel was then visualized with ChemiDoc XRS+ imaging system (Biorad), and the signal analysis was exerted with Image lab software.

### Picrosirius Red Assay

After infection, the NHDF cells were washed 3 × with PBS and then incubated with Bouin solution (Sigma-Aldrich) at RT for 20 min. The cells were incubated with 0.1% Picrosirius red dye (ab150681) at RT for 2 h. Then, they were washed 1 × with 0.01 N HCl and the matrix was dissolved in 0.01 N NaOH. The absorption was measured at 570 nm using a Tecan Infinite M200 plate reader (Tecan, Grödig, Austria). By dividing the absorbance of each sample by the absorbance of the healthy sample, the relative collagen quantity was determined. For each condition, the experiment was performed three times.

### Lactate Dehydrogenase (LDH) Release Assay

The manufacturer’s procedure was followed to measure the released LDH amount in the supernatant of NHDF cells. Briefly, in a 96-well plate (Grenier), 50 μL of the supernatant was combined with 50 μL of substrate. The plate was incubated at RT for 30 min in the dark, and then the reaction was stopped with 50 μL of the stop solution. The absorbance was measured at 490 nm with a Tecan Infinite M200 plate reader (Tecan, Grödig, Austria). The cytotoxicity was calculated relative to the control (no inhibitor).

### Stability of 27 with LC-MS

A concentration of 200 μM of compound **27** was incubated with DMEM medium at 37 °C for 5 h and 5% CO_2_. After the incubation, 2 μL of the compound was transferred to LC-MS vials containing 200 μL of acetonitrile and LC-MS spectra were measured. Three controls were included: (i) **27** in DMEO, (ii) **27** in DMEM without incubation, and (iii) DMEM medium.

### Transepithelial Electric Resistance (TEER) Experiment

MDCK II cells were seeded at a density of 3 × 10^4^ cells/mL onto a Millipore hanging cell culture insert at 37 °C for 12 days with 5% CO_2_. On day 5, the medium was changed. Prior to treatment, the cells were starved for 16 h in FCS-free RPMI medium (Gibco). The bacteria or bacterial-free supernatant was prepared; the bacteria was used at an MOI of 0.03, while 50% (*v*/*v*) of supernatant was added. The ColQ1 inhibitors were added into the inner compartment. The TEER of the cells was measured with Millicell ERS-2 (Electrical Resistance System) over time. Three readings were recorded for each well, and the unit area resistance (UAR) was calculated using the mean values of the TEER following the equation
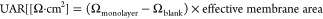
Changes in TEER were normalized to the initial UAR (*t* = 0), which was set to 100%.

### *B. cereus* Supernatant Production

*B. cereus* AH187 strain was cultured in FCS-free DMEM medium at 37 °C. The supernatant was harvested by centrifugation and kept at −80 °C until needed. The supernatant was sterile-filtered with a 0.22 μm filter (Greiner).

### *B. cereus* Growth Inhibition Assay

The effect of the compounds on *B. cereus* growth was carried out by growing the bacteria in BHI medium until the mid-growth phase. Next, the bacterial suspension was diluted until OD_600 nm_ was 0.2 and combined with compounds in a range of 200–3 μM in a 96-well plate. The plates were subsequently incubated at 37 °C for 48 h in a Tecan Infinite M200 plate reader (Tecan, Grödig, Austria). The MIC values presented are the average of at least two independent determinations.

### *In vivo Galleria Mellonella* Infection Model

*G. mellonella* larvae were purchased from a fishing store. Injections were carried out using an LA120 syringe pump (Landgraf Laborsysteme, Langenhagen, Germany) equipped with 1 mL Injekt-F tuberculin syringes (B. Braun, Melsungen, Germany) and Sterican 0.30 × 12 mm^2^, 30 G × 1.5 needles (B. Braun). The larvae were divided into five groups depending on their treatment: (i) sterile PBS, (ii) no injection, (iii) only compound, (iv) BC AH187 bacterial suspension, and (v) BC AH187 bacterial suspension with compound. Larvae were incubated at 37 °C for 3 days and inspected twice daily. The total larvae used in all three experiments were 40 larvae per group. When the larvae became black and did not move when simulated with a tweezer, they were deemed dead.

### Crystallization, X-ray Data Collection, and Analysis

Crystals of ColG-PD were grown in 0.1 M tris-Bicine pH 8.5, 0.04 M pentaethylene glycol, 0.04 M diethylene glycol, 0.04 M triethylene glycol, 0.04 M tetraethylene glycol, 10% (*w/v*) poly(ethylene glycol) 20,000, and 20% (*v*/*v*) poly(ethylene glycol) 550 monomethyl ether in sitting-vapor diffusion plates. Crystals were soaked with 10 mM **27** and **13** for 2 weeks. The crystals were cryoprotected with MiTeGen LV Cryo-oil (MiTeGen, Ithaca, NY) and immediately flash-frozen in liquid nitrogen. X-ray diffraction data was collected on beamline ID30 at the European Synchrotron Radiation Facility (ESRF) in Grenoble, France. The data sets were indexed, integrated, and scaled using XDS^47^ and AIMLESS.^48^ Molecular replacement was performed with PHASER^49^ using as search model PDB entry 2y6i (ligand and activator domain deleted). Ligand coordinates and restraints were generated using the Grade Web Server.^50^ Final structures were obtained using PHENIX^51^ together with model building in WinCoot.^52^ PyMOL v 4.0.0 was used for figure generation (The PyMOL Molecular Graphics System, Version 4.0.0 Schrödinger, LLC). The final refined structures were deposited in the Protein Data Bank (PDB) as entries 7Z5U and 7ZBV. Data collection and refinement statistics are listed in Table S2.

### Statistical Analysis

Graphical data in the manuscript is presented as the means ± SDs. Statistical comparisons are performed by Tukey one-way ANOVA test, which shows significant differences between conditions. Parametric/nonparametric statistical analysis used in the study was based on normality and homogeneity of variance. A value of *p* ≤ 0.05 was considered statistically significant, while *p* > 0.05 was considered nonsignificant. The normalized measurements were statistically compared between treated and nontreated groups using the generalized estimating equation model to account for correlated data arising from repeated measures. The survival of *G. mellonella* was computed using the Kaplan–Meier method, and the log-rank test was applied to calculate the significance of differences between conditions.

## Supporting Information Available

Molecular Formula Strings (CSV)
Screening results for ColH and ColQ1, inhibition of MMPs and other off-targets by selected compounds, and cytotoxicity and antibacterial activity of selected compounds (Tables S1–S5); inhibition of the screened compounds on ColQ1 and ColH, activity of the compounds on Col I cleavage, on NHDF infected cells, on TEER of MDCK II cells, and on *G. mellonella* infection model; ^1^H NMR and ^13^C NMR and LC-MS spectra for final compounds and data collection and refinement statistics (Figures S4–S17) (PDF)

## Notes

PDB codes: compound **13** (7ZBV) and compound **27** (7Z5U). The authors will release the atomic coordinates upon article publication.

## Abbreviations Used

BHI: brain heart infusion medium
Col I: collagen I
ColQ1 and ColA: collagenases from *Bacillus cereus* AH187 and Q1 strains
ColH: ColG *Clostridium histolyticum* collagenases
CU: collagenase unit
DMEM: Dulbecco’s modified Eagle’s medium
ECM: extracellular matrix
FCS: fetal calf serum
FRET: fluorescence resonance energy transfer
HEK293: embryonal kidney cell
HepG2: hepatocellular carcinoma cell
IC_50_: the half-maximal inhibitory concentration
LDH: lactate dehydrogenase
M (kDa): molecular weight standards in kilo Dalton
MDCK II: Madin–Darby canine kidney II
MIC: minimum inhibitory concentration
MMPs: human matrix metalloproteases
NHDF: normal human dermal fibroblast cell
PD: peptidase unit
RPMI: Roswell Park Memorial Institute Medium
SDS-PAGE: sodium dodecyl sulfate polyacrylamide
TEER: transepithelial electrical resistance
ZBG: zinc-binding group
