# Supplementary material for: Discovery and Characterization of Synthesized and FDA-Approved Inhibitors of Clostridial and Bacillary Collagenases
Source: J Med Chem. 2022 Sep 26;65(19):12933–55. doi: 10.1021/acs.jmedchem.2c00785 (PMC9574867; doi:10.1021/acs.jmedchem.2c00785)

Supporting Information

Discovery and Characterization of Synthesized and FDA-Approved Inhibitors of Clostridial and Bacillary Collagenases

Alaa Alhayek[1,2,⊥], *Ahmed S. Abdelsamie*[1,3,⊥], *Esther Schönauer*[4], *Virgyl Camberlein*[1], *Evelyn Hutterer*[4], *Gernot Posselt*[4], *Jamil Serwanja*[4], *Constantin Blöchl*[4], *Christian G. Huber*[4], *Jörg Haupenthal*[1], *Hans Brandstetter*[4], *Silja Wessler*[4,*], *and Anna K. H. Hirsch*[1,2,*]

[⊥, *] these authors contributed equally to this work

1. Helmholtz Institute for Pharmaceutical Research Saarland (HIPS) – Helmholtz Center for Infection Research (HZI), Campus Building E8.1, 66123 Saarbrücken, Germany
2. Department of Pharmacy, Saarland University Campus Building C2. 3, 66123 Saarbrücken, Germany
3. Department of Chemistry of Natural and Microbial Products, Institute of Pharmaceutical and Drug Industries Research, National Research Centre, El-Buhouth St., Dokki, 12622, Cairo, Egypt
4. Department of Biosciences and Medical Biology, University of Salzburg, Hellbrunner Str. 34, 5020 Salzburg, Austria

Corresponding authors:

anna.hirsch@helmholtz-hips.de

Silja.Wessler@sbg.ac.at

Table of Contents

Supplementary Tables **S3**

Supplementary Figures **S12**

- **Chemistry** **S30**
  - **Diphosphonates** S30
  - **Hydroxamates** S32
- **NMR-Spectra** **S36**
  - **Diphosphonate compounds** S36
  - **Hydroxamate compounds** S52
- **LC-MS Spectra** **S60**
  - **Diphosphonate compounds** S60
  - **Hydroxamate compounds** S66

Supplementary Tables

Table S1. Screening results for peptidase domain (PD) of ColH and collagenase unit (CU) of ColQ1 inhibitors discovery. The testing was performed at a concentration of 100 µM.

Compound	% Inhibition (ColH-PD)		% Inhibition (ColQ1-CU)		Structure
	Mean	SD	Mean	SD	
**13**	83	9	98	1	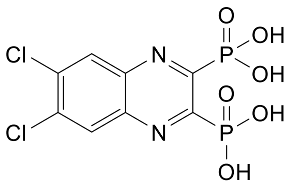
**27**	84	2	97	2	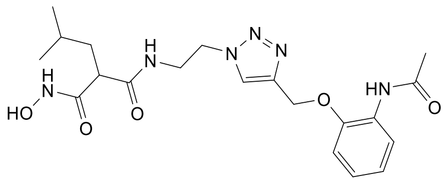
**35**	32	12	23	3	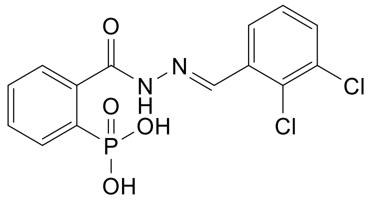
**36**	86	5	72	5	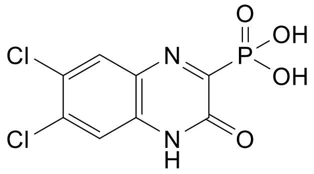
**37**	24	8	4	2	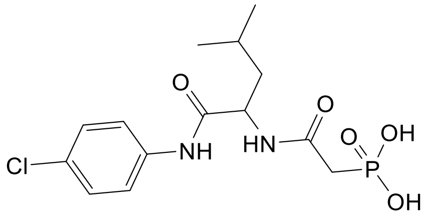
**38**	19	7	3	1	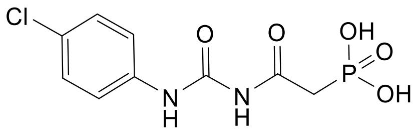

Compound	% Inhibition (ColH-PD)		% Inhibition (ColQ1-CU)		Structure
	Mean	SD	Mean	SD	
**39**	30	14	3	10	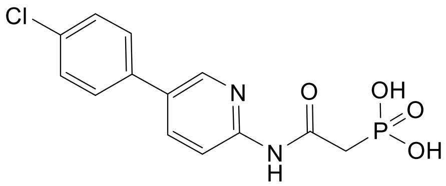
**40**	0	7	-1	1	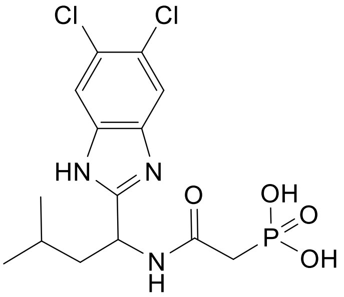
**41**	23	9	13	6	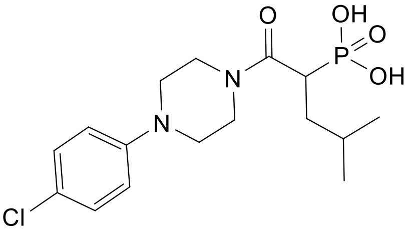
**42**	102	3	98	7	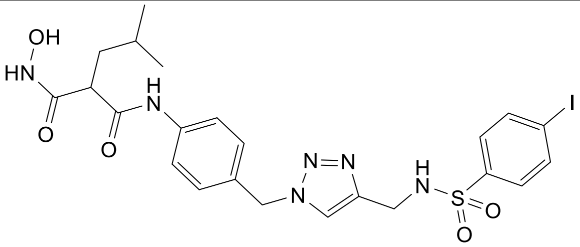
**43**	63	1	87	4	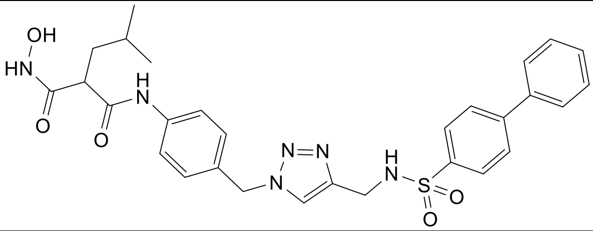
**44**	82	5	90	2	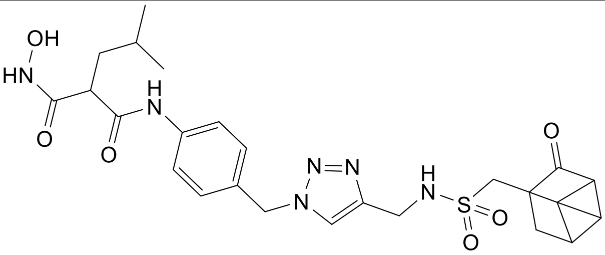

Compound	% Inhibition (ColH-PD)		% Inhibition (ColQ1-CU)		Structure
	**Mean**	**SD**	**Mean**	**SD**	
**45**	67	4	84	2	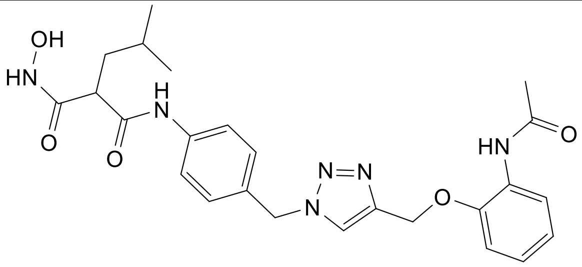
**46**	18	8	88	2	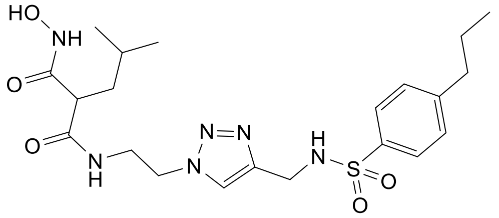
**47**	43	5	53	2	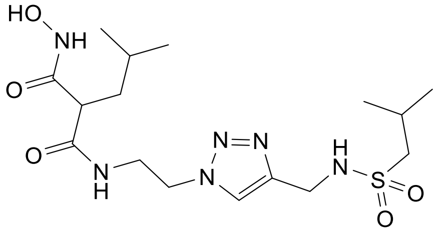
**48**	30	9	-4	5	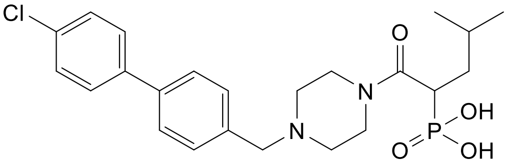
**49**	-5	6	10	7	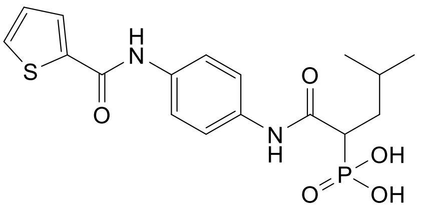
**50**	17	7	2	3	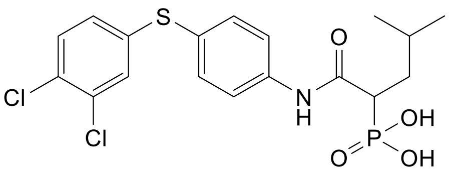
**51**	47	8	84	5	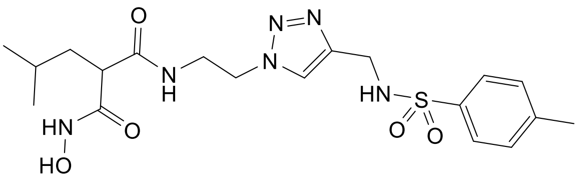

Compound	% Inhibition (ColH-PD)		% Inhibition (ColQ1-CU)		Structure
	**Mean**	**SD**	**Mean**	**SD**	
**52**	42	6	59	2	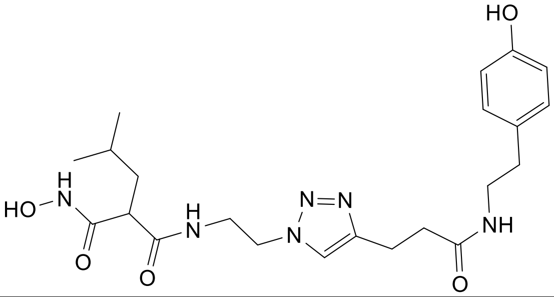
**53**	84	7	85	1	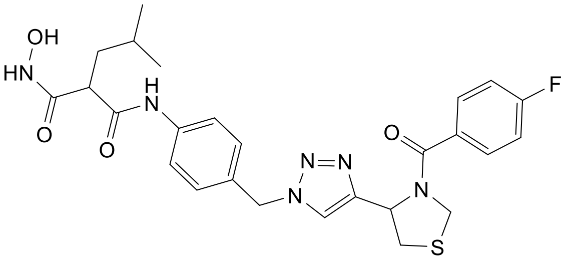
**54**	7	16	16	4	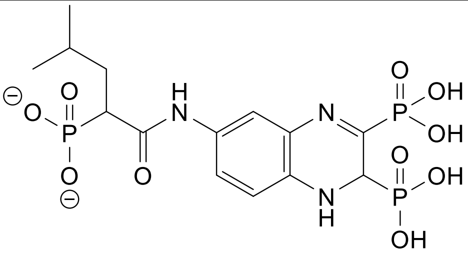
**55**	34	8	0	3	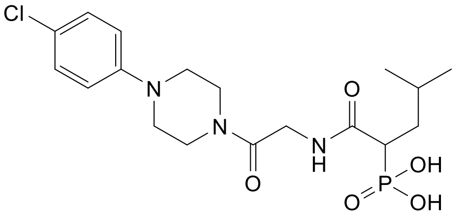
**56**	-16	3	0	2	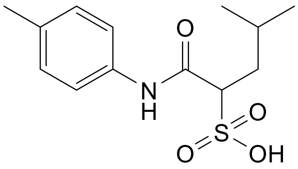
**57**	-8	21	0	3	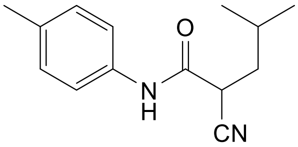

Compound	% Inhibition (ColH-PD)		% Inhibition (ColQ1-CU)		Structure
	**Mean**	**SD**	**Mean**	**SD**	
**58**	-16	16	-4	7	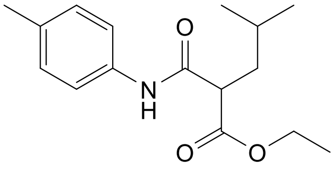
**59**	61	0	64	1	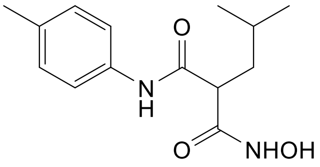
**60**	1	14	-3	1	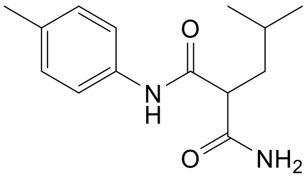
**61**	-5	6	-8	2	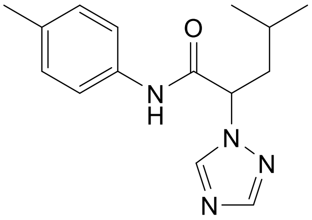
**62**	4	10	-2	1	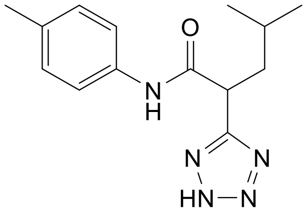
**63**	24	9	-4	5	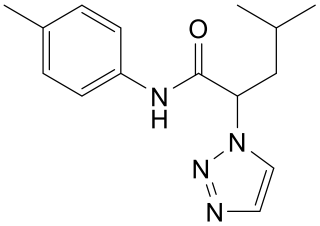
**64**	18	16	5	3	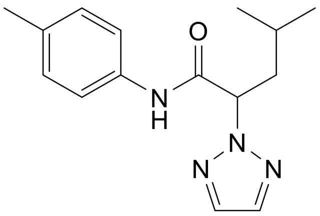

Compound	% Inhibition (ColH-PD)		% Inhibition (ColQ1-CU)		Structure
	**Mean**	**SD**	**Mean**	**SD**	
**65**	72	7	-3	4	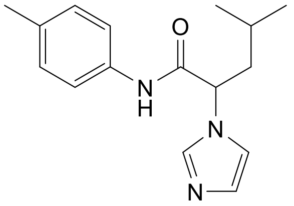
**66**	95	4	92	2	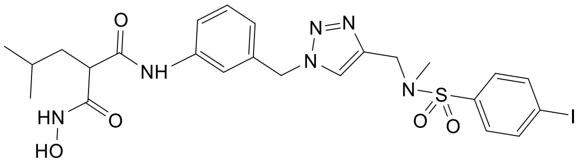
**67**	105	3	89	2	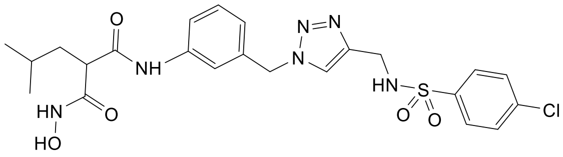
**68**	58	6	Ki 6.9	0.2	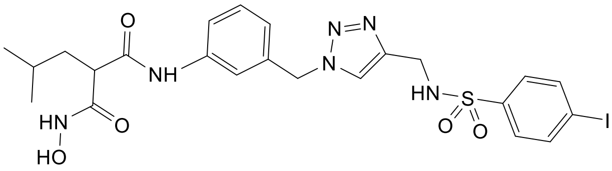
**69**	75	2	84	8	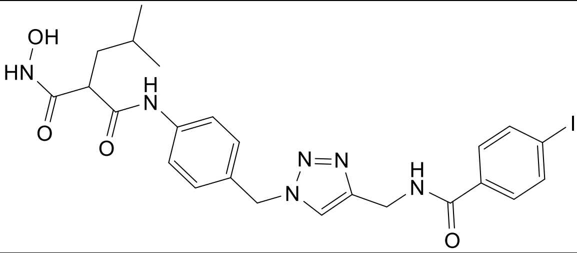
**70**	32	16	-8	3	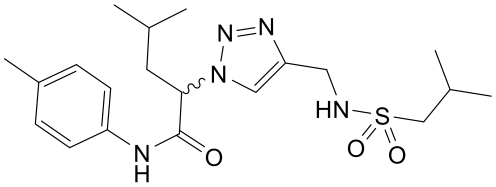
**71**	n.d.	n.d.	58	5	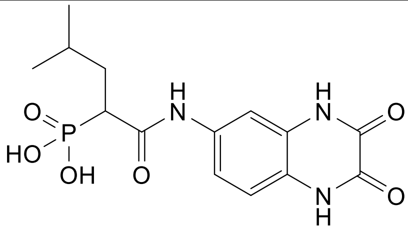

Compound	% Inhibition (ColH-PD)		% Inhibition (ColQ1-CU)		Structure
	**Mean**	**SD**	**Mean**	**SD**	
**72**	n.d.	n.d.	35	4	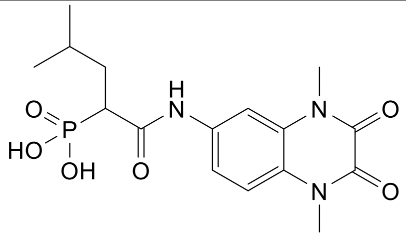

n.d.: not determined

Table S2. Data collection and refinement statistics for ColG-PD in complex with compound 27 and 13[a]

**Data collection**	**27**	**13**
Wavelength (Å)	0.976254	0.976254
Resolution range (Å)	42.46–1.80 (1.86–1.80)[a]	60.27–1.95 (2.02–1.95)
Space Group	P 2_1 2_1 2_1	P 2_1 2_1 2_1
	Cell dimensions	
a, b, c (Å)	59.31, 78.39, 96.41	60.27, 77.79, 97.32
α, β, γ (°)	90.00, 90.00, 90.00	90.00, 90.00, 90.00
No. of unique reflections	38367 (3832)	33936 (3303)
R_{merge}	0.0400 (0.3339)	0.0372 (0.3959)
Mean I/sigma(I)	11.09 (2.20)	12.33 (2.06)
Completeness (%)	90.52 (91.91)	99.69 (99.70)
Multiplicity	1.9 (1.9)	2.0 (2.0)
$CC_{1/2}$	0.998 (0.773)	0.998 (0.699)
CC*	0.999 (0.934)	1.000 (0.907)
Wilson B-factor	23.35	30.71
	**Refinement**	
Resolution range (Å)	42.46–1.80 (1.86–1.80)	41.25–1.95 (2.02–1.95)
R_{work} / R_{free}	0.1796 (0.2480) / 0.2073 (0.2909)	0.1865 (0.2746) / 0.2286 (0.3074)
No. of non-hydrogen atoms	3295	3284
Protein	3113	3161
Ligand	61	74
Solvent	149	73
B factors	34.04	37.46
Protein	34.11	37.20
Ligand	25.20	58.25

Data collection	27	13
Solvent	34.43	34.44
**RMSD**		
Bond lengths (Å)	0.014	0.012
Bond angles (°)	1.10	1.15
Ramachandran favored (%)	98.17	98.71
Ramachandran allowed (%)	1.57	1.29
Ramachandran outliers (%)	0.26	0.00
PDB code	7Z5U	7ZBV

[a]Statistics for the highest-resolution shell are shown in parentheses.

Table S3. Activity of compounds 13 14, 15, 27, tiludronate disodium, and alendronate sodium against HDAC-3, HDAC-8, TACE, and COX-1[a]

		IC$_{50}$ (μM)			
**Class**	Cpb.	HDAC-3	HDAC-8	TACE	COX-1
**Synthesized diphosphonates**	**13**	>100	>100	>100	>100
	**14**	>100	>100	>100	>100
	**15**	>100	>100	>100	>100
**FDA-approved diphosphonates**	**Tiludronate disodium**	>100	>100	>100	>100
	**Alendronate sodium**	>100	>100	>100	>100
**Hydroxamate**	**27**	>100	>100	>100	n.d.

[a]Means and SD of two independent experiments, n.d.: not determined, >100: IC$_{50}$ is higher than 100 μM

Table S4. Cytotoxicity of compounds 13 14, 15, and 27 at 100 µM or 200 µM against HepG2, HEK293, NHDF, and MDCK II cell lines[a]

IC$_{50}$ (µM)					
**Class**	**Cpb.**	**HepG2**	**HEK293**	**NHDF**	**MDCK II**
**Synthesized diphosphonates**	**13**	>100	=100	>200	>200
	**14**	>100	>100	>100	>100
	**15**	>100	>100	>200	>200
**Hydroxamate**	**27**	>100	>100	>200	>200

[a]Means and SD of two independent experiments, >100: IC$_{50}$ is higher than 100 µM, >200: IC$_{50}$ is higher than 200 µM.

Table S5. Antibacterial activity of selected compounds on *Bacillus cereus* AH187[a]

**Class**	**Cpd.**	**MIC$_{50}$ (µM) AH187**
**Synthesized diphosphonates**	**10**	>200
	**11**	>200
	**13**	>200
	**14**	>100
	**15**	>100
**FDA-approved diphosphonates**	**Tiludronate disodium**	>200
	**Alendronate sodium**	>200
**Hydroxamate**	**27**	>200

[a]Means and SD of two independent experiments.

Supplementary Figures

Inhibition of the compounds *vs* ColQ1-CU and ColH-PD

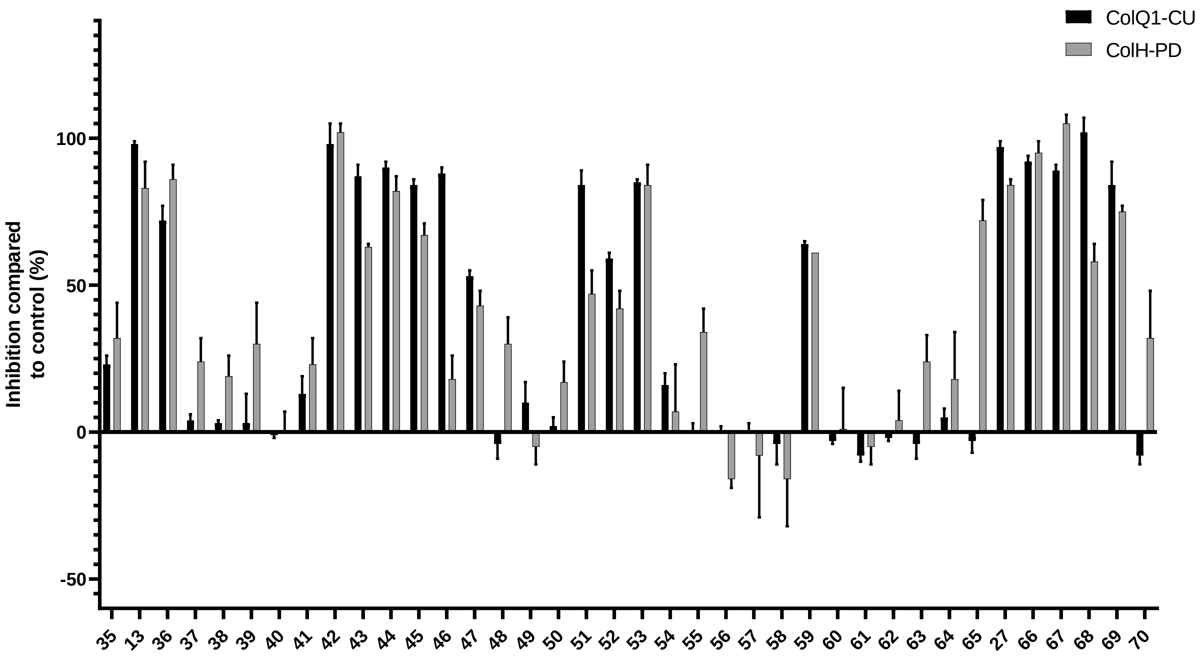

Figure S1. Inhibition of the screened compounds at 100 µM *vs* collagenase unit (CU) of ColQ1 and peptidase unit (PD) of ColH.

Inhibition of the hydroxamate compounds *vs* ColQ1-CU at 1, 10, and 100 μM concentrations.

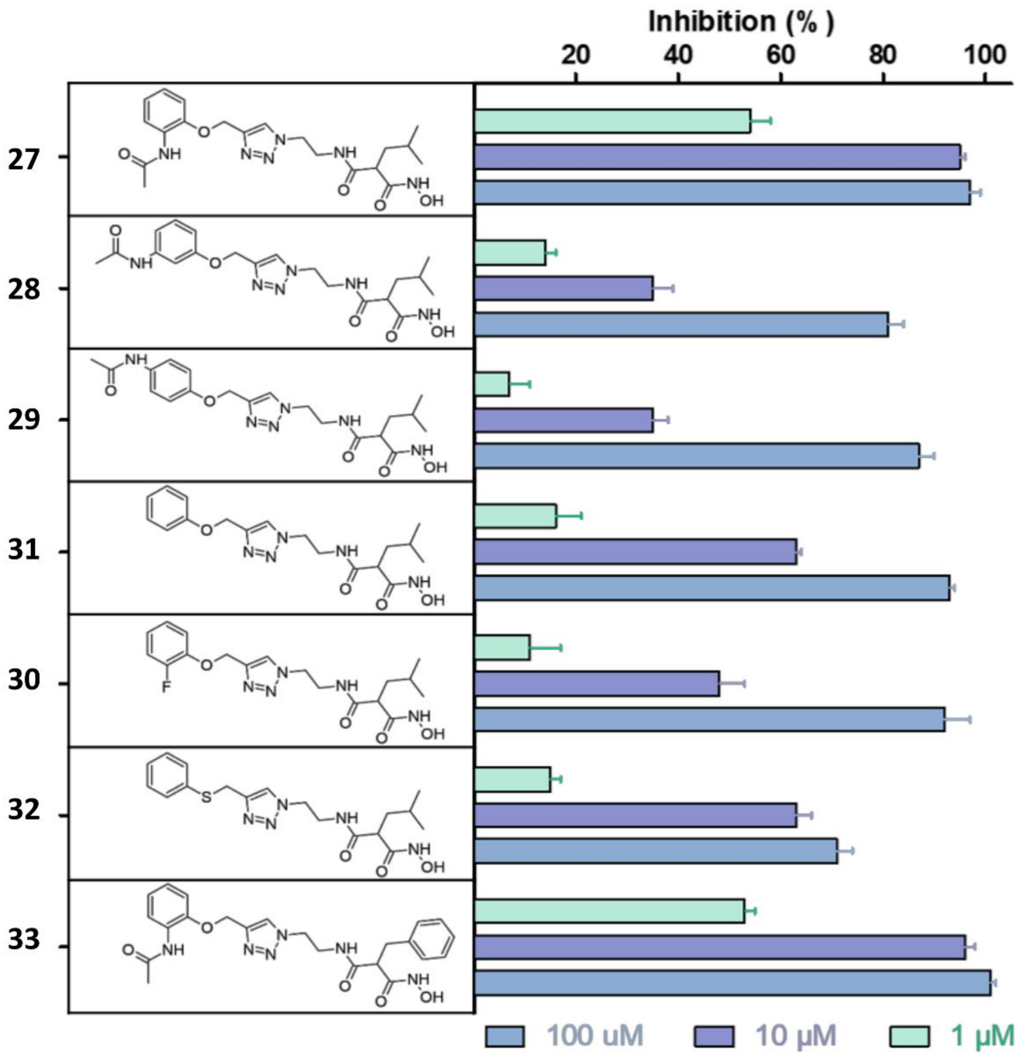

Figure S2. Inhibition of ColQ1-CU by hydroxamates at 1, 10, and 100 μM concentration.

ColQ1 inhibitors effect on collagen I cleavage

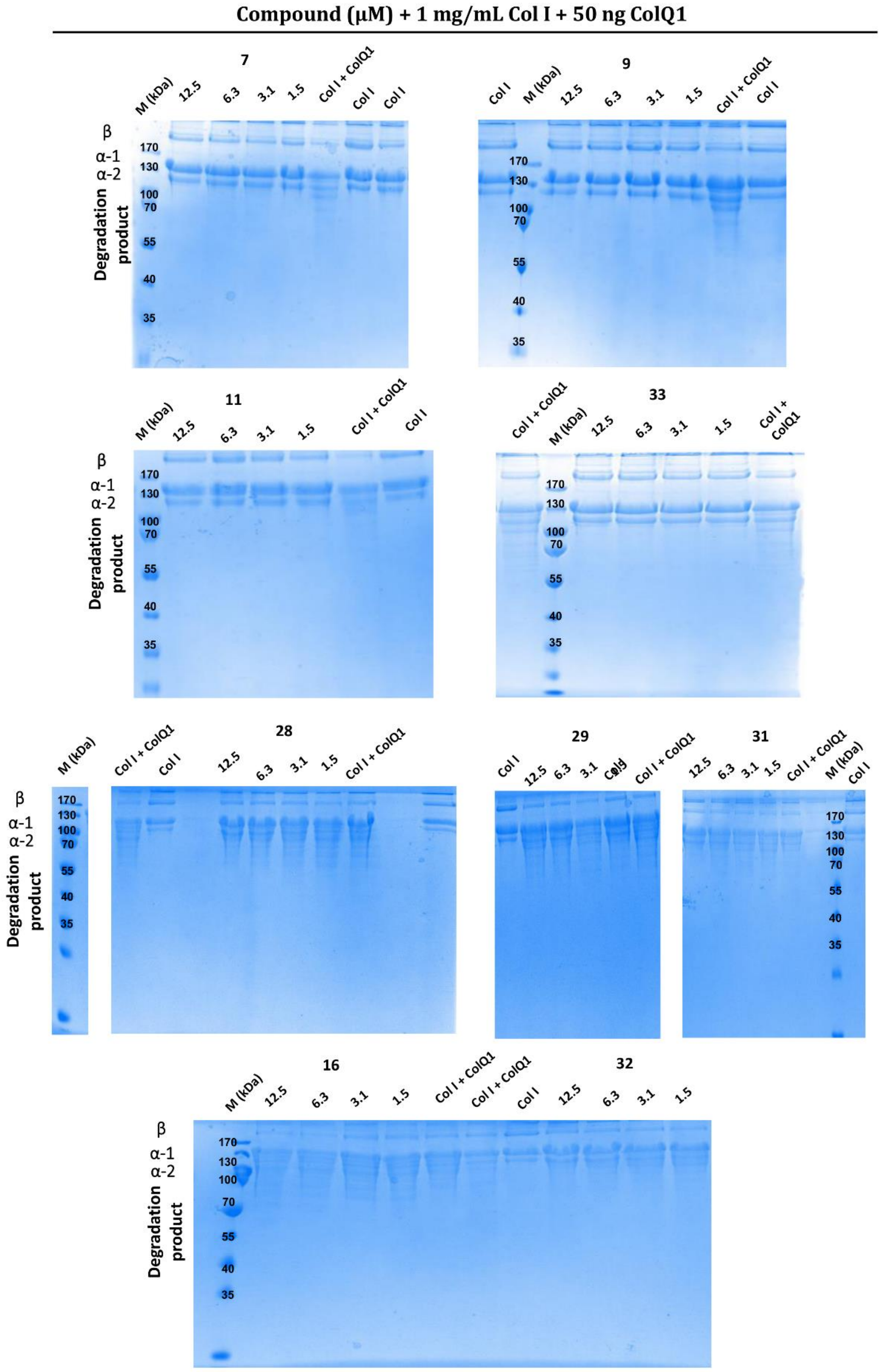

Figure S3. The effect of ColQ1 inhibitors on Col I cleavage. The *Bacillus cereus* collagenase Q1 **(**ColQ1) full length (50 ng) was incubated with 1 mg/mL Col I for 3 h, and the degradation was then visualized on 12% SDS-PAGE. Col I: 1 mg/mL Col I without any protease. M (kDa): molecular weight standards, Col I: type I collagen.

The release of *Bacillus cereus* AH187 collagenases over time

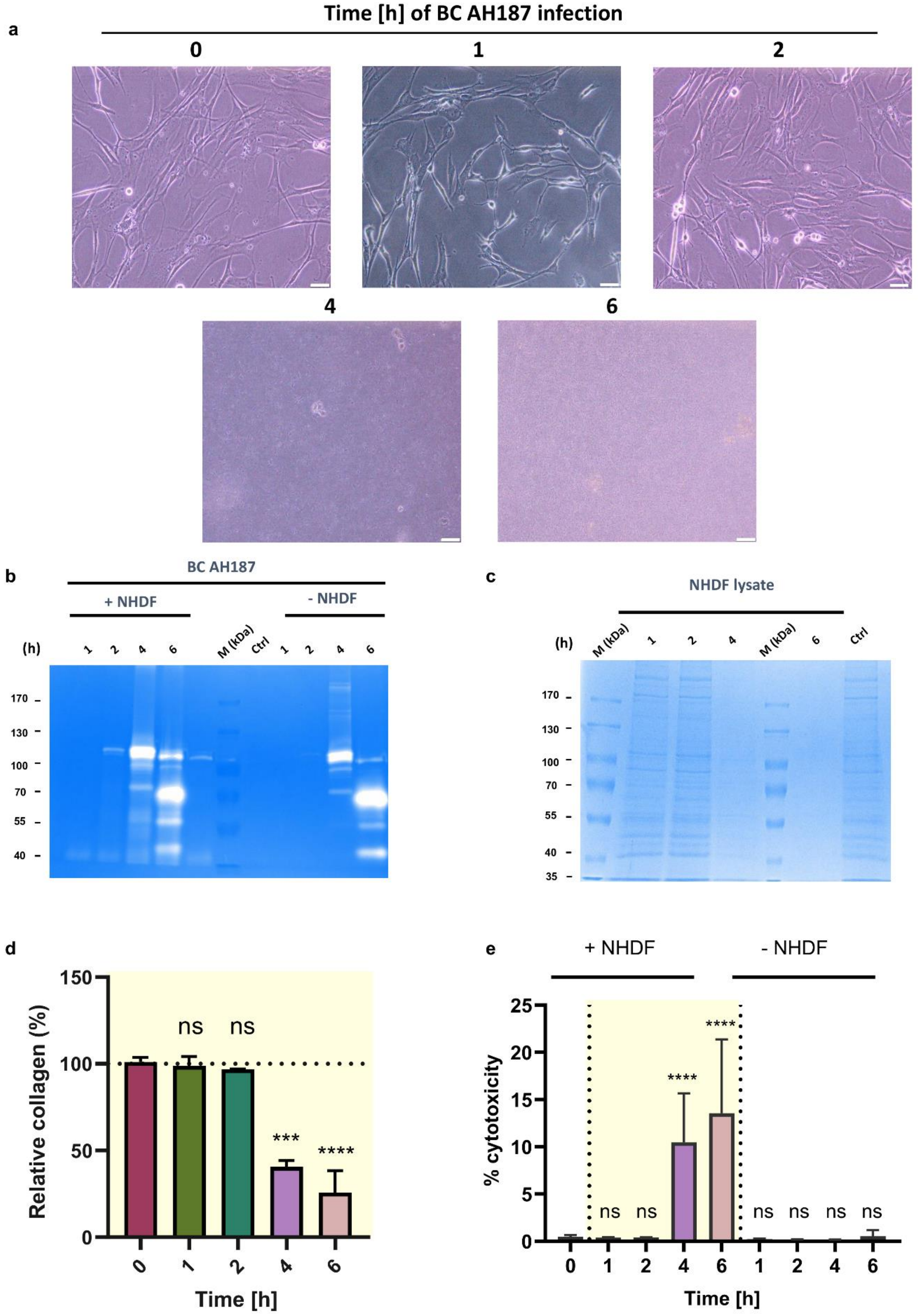

Figure S4. The release of collagenases of *Bacillus cereus* AH187 over 6 h period of time. a) The morphological changes in the fibroblast (NHDF) cells and their detachment over time. **b)** Gelatin zymogram represents the activity of *Bacillus cereus* collagenases released over 1, 2, 4, and 6 h in presence (+ NHDF) and absence of NHDF (-NHDF) cells. The DMEM medium of the infected NHDF cells were applied on the zymograms. Clear regions

against blue background indicate gelatin in the gel has been cleaved **c)** The depletion in the cell lysate over time indicating loss of cells, equal amount of protein was loaded**. d)** The reduction in fibrillar collagen content of the infected NHDF cells over incubation time. **e)** The cytotoxicity induced by toxins and proteases released by *Bacillus cereus* increased over time. The yellow background in **d** and **e** highlighted the infected cells with *B. cereus*. Bright-field images captured by 20x objective, scale bar: 50 µm. Statistical analysis was performed with one-way ANOVA and statistical significance was analyzed by Tukey test. Significance was calculated by comparing non-infected *vs* infected cells (mean ± SD, **** $p < 0.0001$, *** $p < 0.001$, ns: non-significant). M (kDa): molecular weight marker.

The activity of the diphosphonate compounds on NHDF infection model

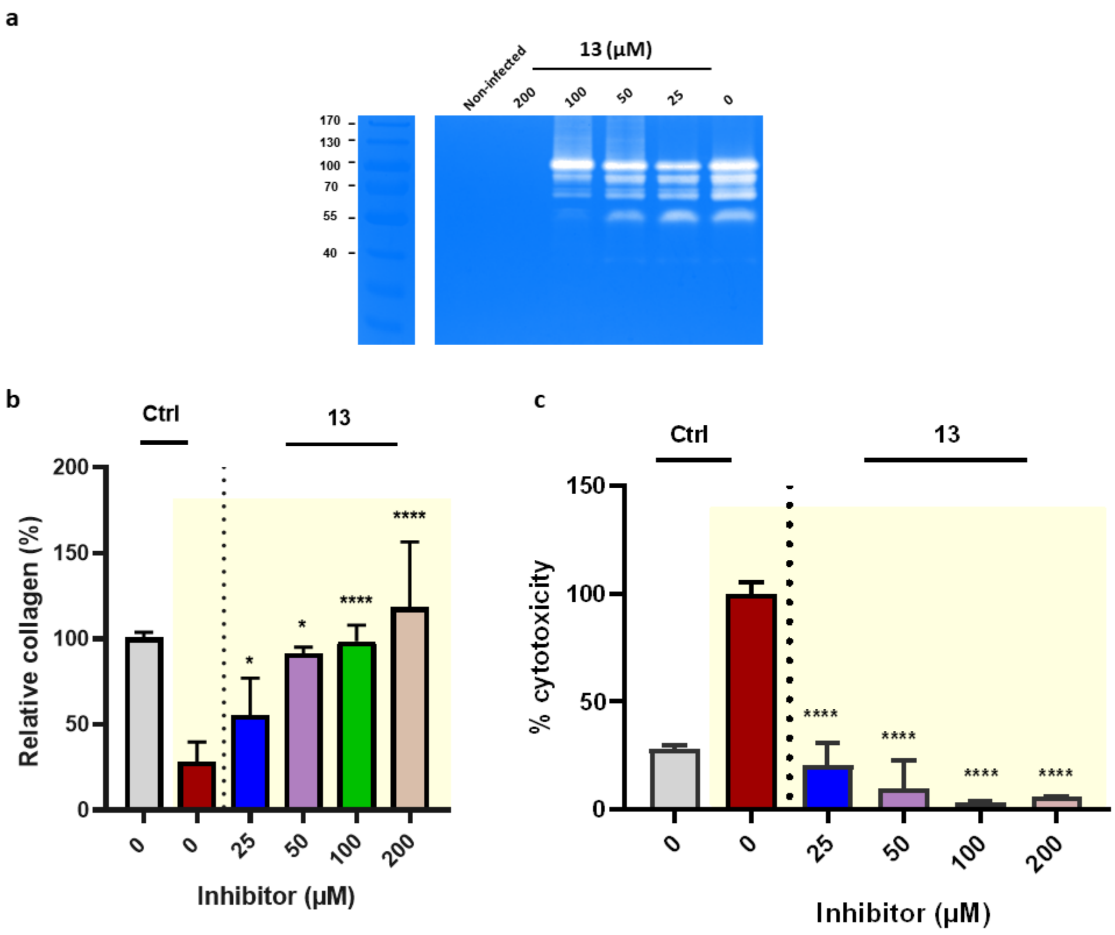

Figure S5. The activities of compound 13 on the fibroblast (NHDF) cells infected with *Bacillus cereus*. a) The anti-gelatinolytic activities of compound **13** against *B. cereus* collagenases. The DMEM medium of the infected NHDF cells were applied on the zymograms. Clear regions against blue background indicate gelatin in the gel has been cleaved. **b)** The amount of fibrillar collagens maintained by **13** in the infected NHDF cells (highlighted in yellow). **c)** The cytotoxicity of *B. cereus* infection (highlighted in yellow) in NHDF cells treated with and without **13**. Ctrl represents the non-infected cells (grey column) and the infected cells and non-treated with inhibitors (red column). Statistical analysis was performed with one-way ANOVA and statistical significance was analyzed by Tukey test. Significance was calculated by comparing non-treated *vs* treated cells with compounds (mean ± SD, **** $p < 0.0001$, * $p \leq 0.05$). Ctrl: control. M (kDa): molecular weight marker.

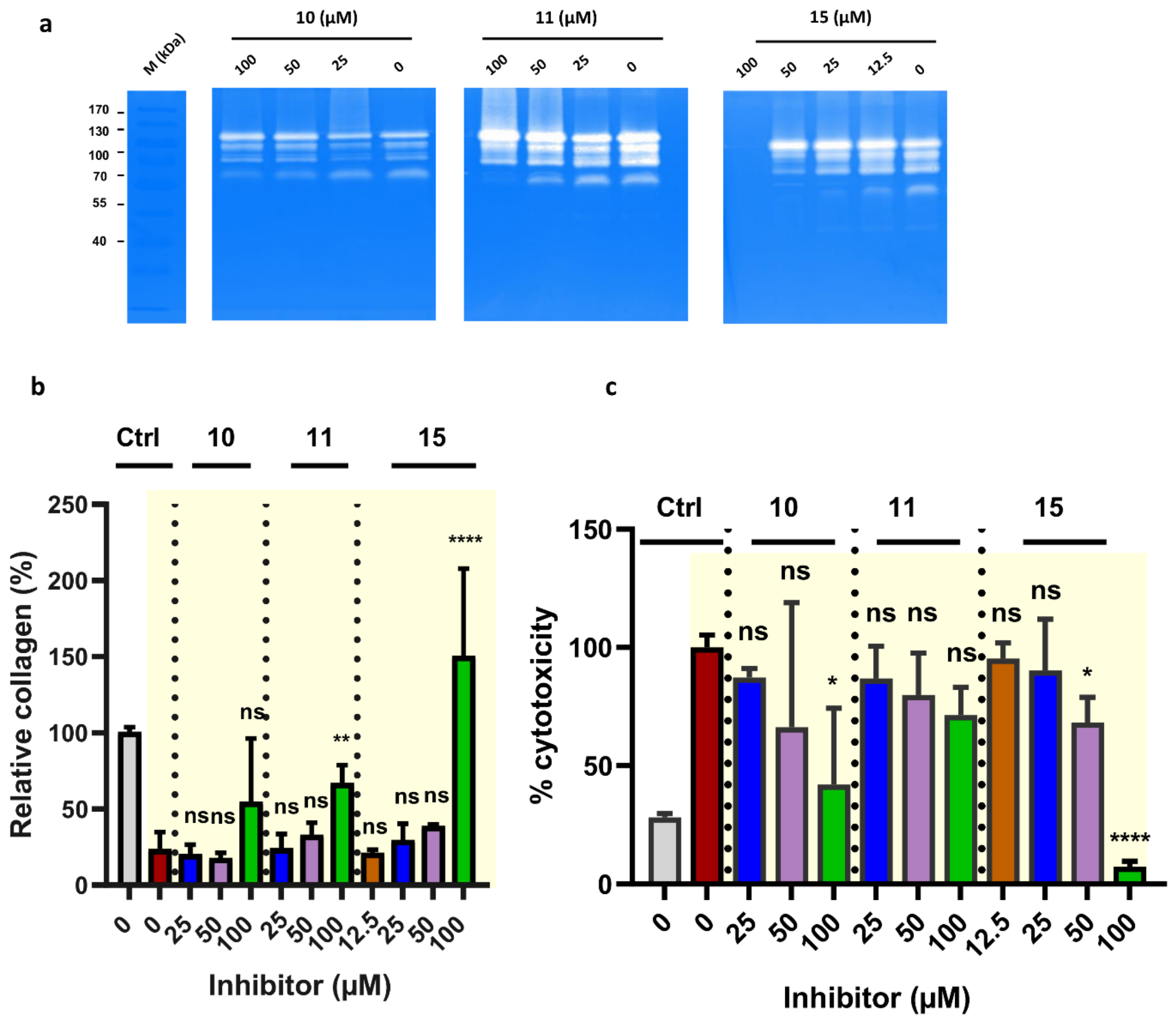

Figure S6. The activity of small-molecule inhibitors on collagenases secreted by *Bacillus cereus* during infection of fibroblast (NHDF) cells. a) The anti-gelatinolytic activity of **10**, **11**, and **15**. The DMEM medium of the infected NHDF cells were applied on the zymograms. Clear regions against blue background indicate gelatin in the gel has been cleaved. **b)** The quantity of fibrillary collagens preserved in the infected NHDF cells treated with **10**, **11**, and **15**. **c)** The change in the cytotoxicity upon the treatment with ColQ1 inhibitors. The yellow background in **b** and **c** highlighted the infected cells with *B. cereus*. Ctrl represents the non-infected cells (grey column) and the infected cells and non-treated with inhibitors (red column). One-way ANOVA was used for statistical analysis, and the Tukey test was used to determine statistical significance. The significance of the results was determined by comparing non-treated *vs* compound-treated cells (mean ± SD, **** $p \leq 0.0001$, ** $p \leq 0.01$, * $p \leq 0.05$, ns: non-significant). M (kDa): molecular weight marker, Ctrl: control.

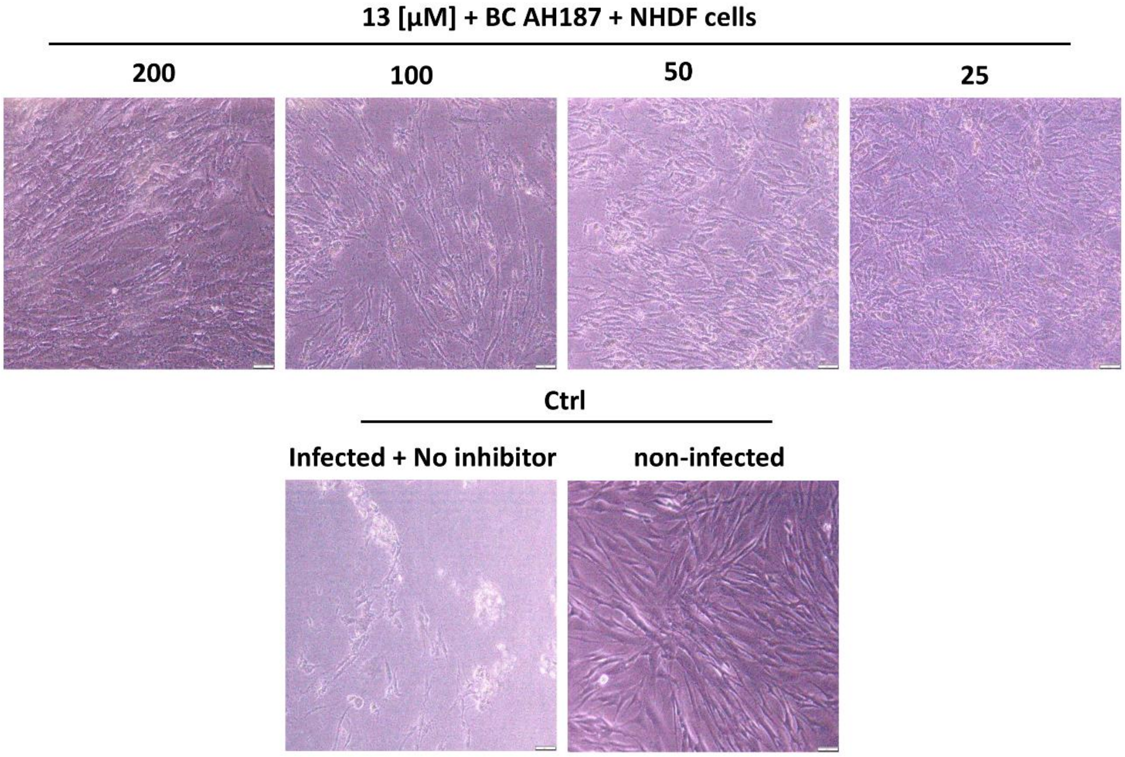

Figure S7. The bright-field images of infected fibroblast (NHDF) cells with *Bacillus cereus* AH187 and treated with and without various concentrations of 13. 20x objectives was used to take the images, scale bar: 50 µm, Ctrl: control.

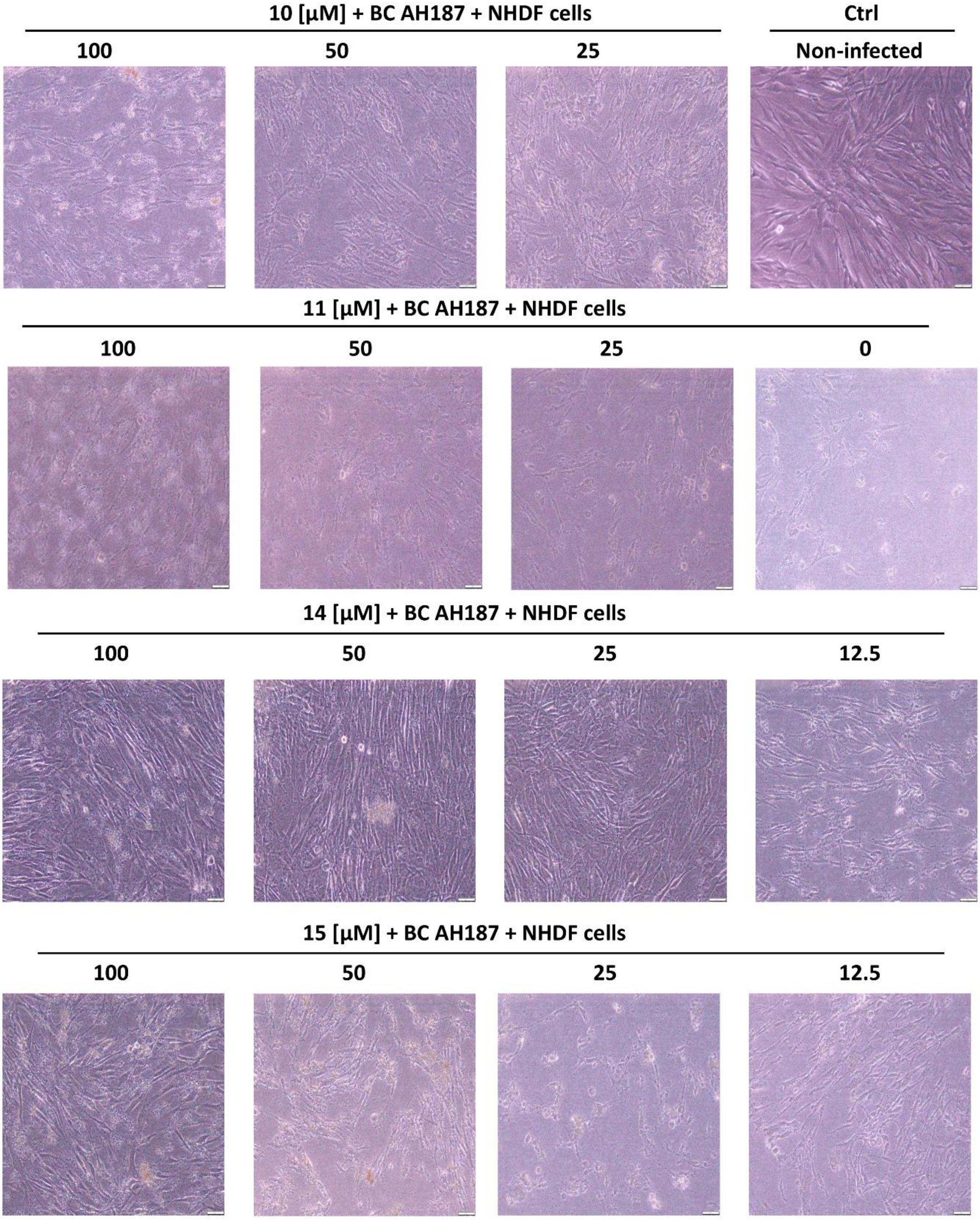

Figure S8. The bright-field images of infected fibroblast (NHDF) cells with *Bacillus cereus* AH187 and treated with and without various concentrations of 10, 11, 14, and 15. 20x objectives was used to take the images, scale bar: 50 µm.

The activity of the FDA-approved diphosphonate alendronate sodium on NHDF infection model

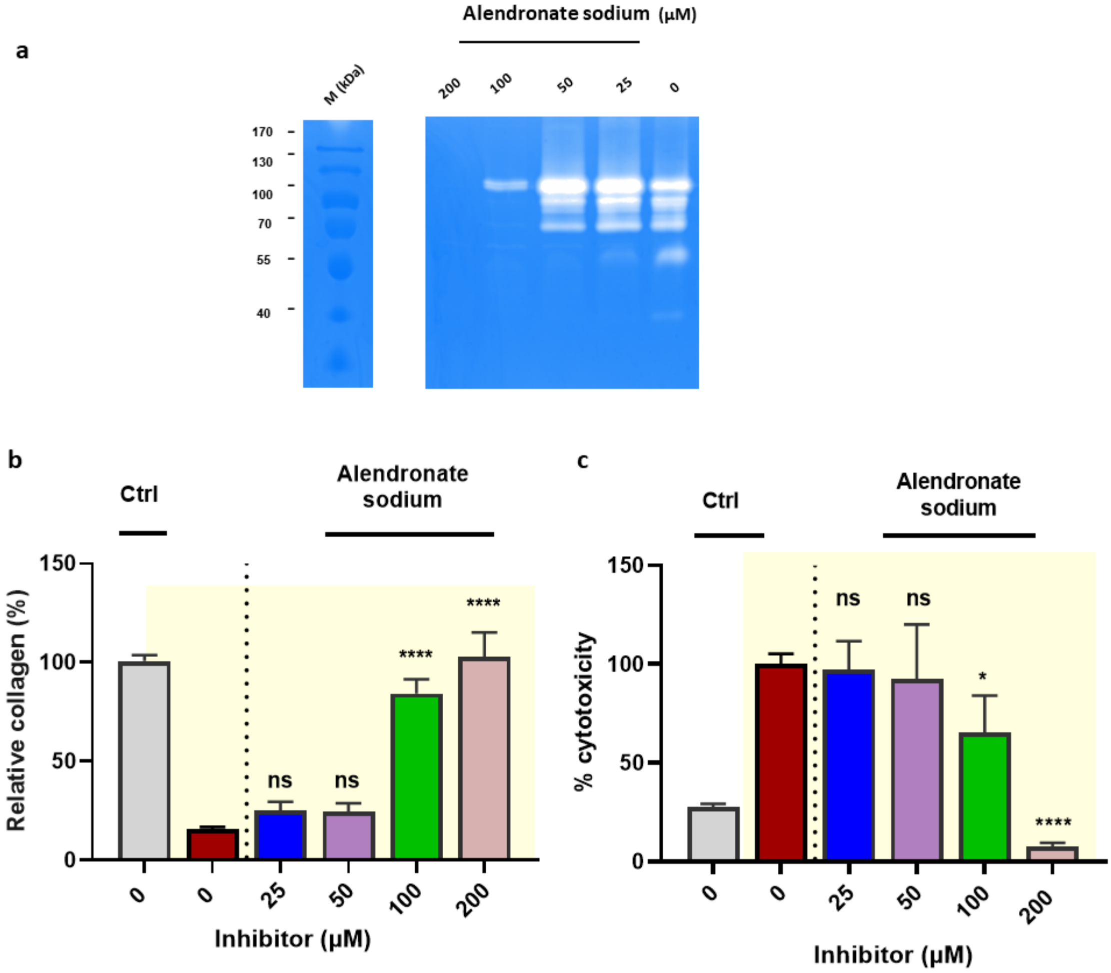

Figure S9. The activity of the FDA-approved diphosphonate drugs on collagenases secreted by *Bacillus cereus* during infecting fibroblast (NHDF) cells. a) The anti-gelatinolytic activity of **alendronate sodium**. The DMEM medium of the infected NHDF cells were applied on the zymograms. Clear regions against blue background indicate gelatin in the gel has been cleaved. **b)** The quantity of fibrillary collagens preserved in the infected NHDF cells treated with and without **alendronate sodium**. **c)** The change in the cytotoxicity upon the treatment with and without **alendronate sodium**. The yellow background in **b** and **c** highlighted that the cells were infected with *B. cereus*. Ctrl represents the non-infected cells (grey column) and the infected cells and non-treated with inhibitors (red column). One-way ANOVA was used for statistical analysis, and the Tukey test was used to determine statistical significance. The significance of the results was determined by comparing non-treated *vs* compound-treated cells (mean ± SD, **** $p \leq 0.0001$, * $p \leq 0.05$, ns: non-significant). M (kDa): molecular weight marker, Ctrl: control.

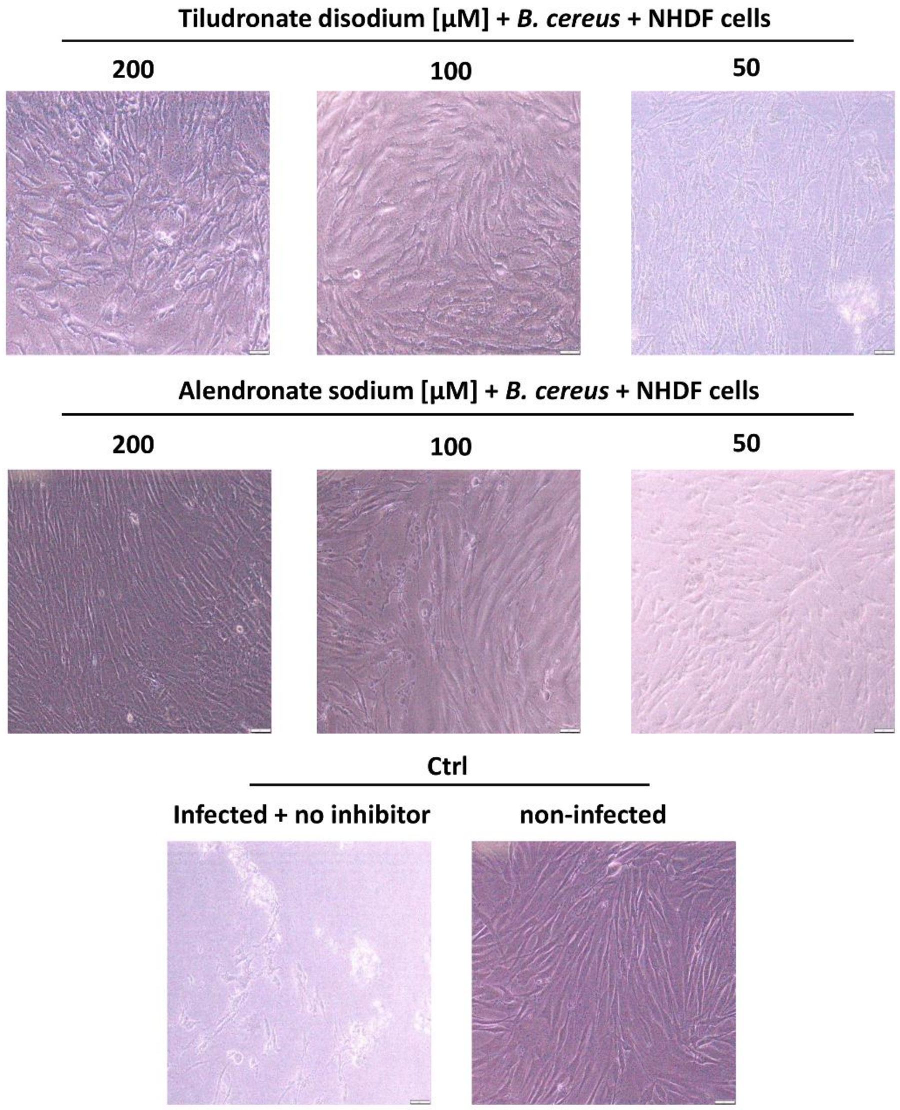

Figure S10. The bright-field images of infected fibroblast (NHDF) cells with *Bacillus cereus* AH187 and treated with and without various concentrations of tiludronate disodium and alendronate sodium. 20x objectives used to take the images, scale bar: 50 µm. Ctrl: control.

The activity of the hydroxamates on NHDF infection model

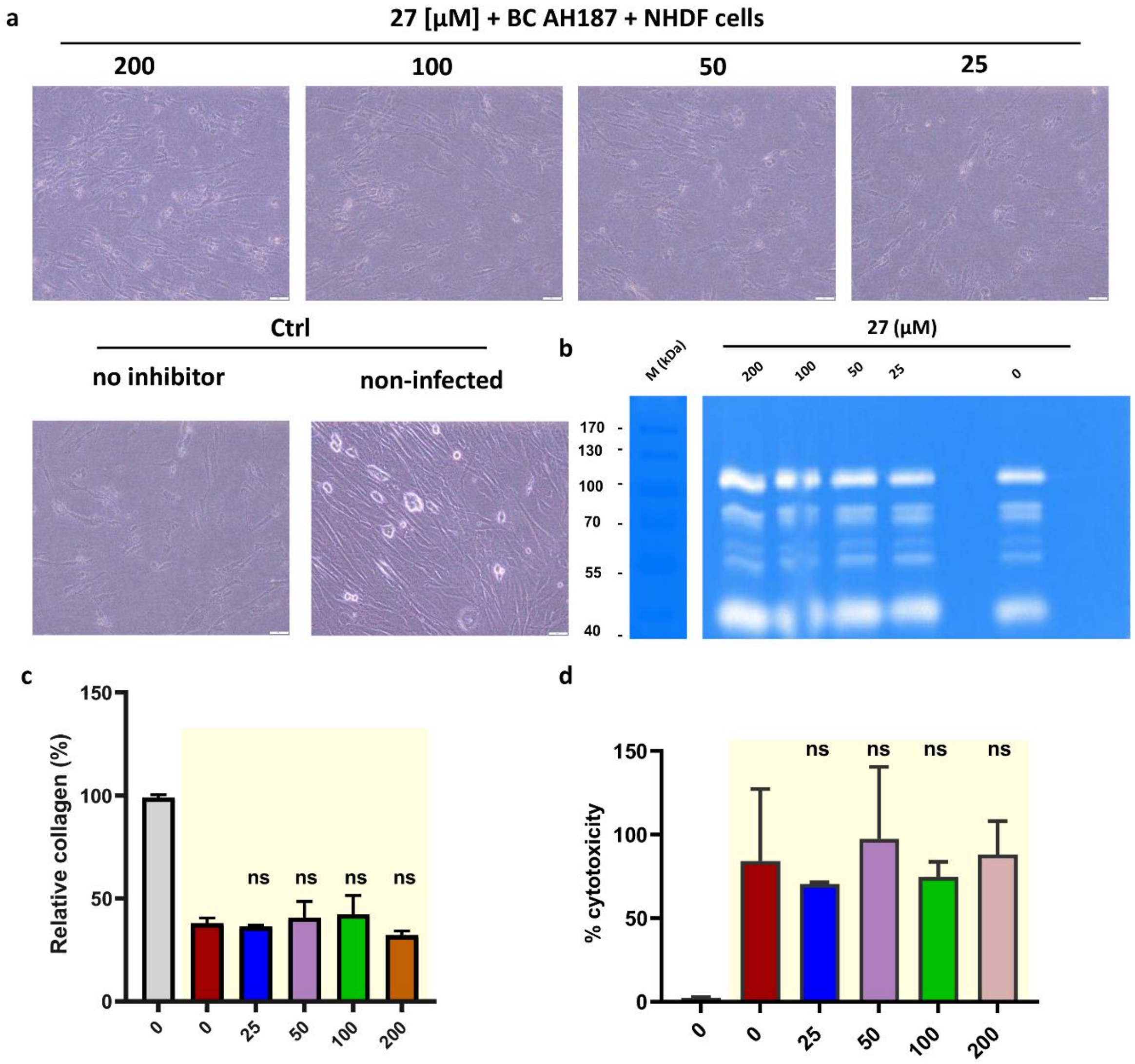

Figure S11. The effect of the hydroxamate compound 27 on *Bacillus cereus*-infected fibroblast (NHDF) cells. **a)** Bright-field images of infected and treated with **27** and non-infected NHDF cells. **b)** Gelatin zymogram of collagenases produced by *Bacillus cereus* during the treatment with **27**. The DMEM medium of the infected NHDF cells were applied on the zymograms. Clear regions against blue background indicate gelatin in the gel has been cleaved. **c)** Relative collagen amount left in the NHDF cells after the infection and the treatment with **27. d)** The cytotoxicity of *Bacillus cereus* released toxins and collagenases in presence of **27**. The yellow background in **c** and **d** highlighted that the cells were infected with *B. cereus*. Ctrl represents the non-infected cells (grey column) and the infected cells and non-treated with inhibitors (red column). One-way ANOVA was used for statistical analysis, and the Tukey test was used to determine statistical significance. The significance of the results was determined by comparing non-treated *vs* compound-treated cells (mean ± SD, ns non-significant). M (kDa): molecular weight marker, Ctrl: control.

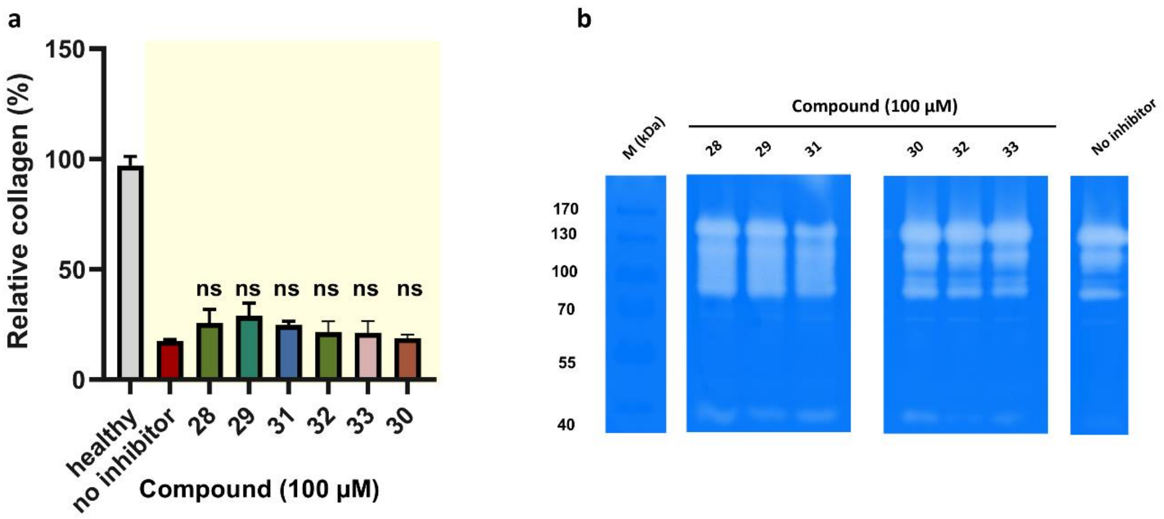

Figure S12. The effect of hydroxamic acid-based compounds on *Bacillus cereus*-infected fibroblast (NHDF) cells at a concentration of 100 µM. a) Relative collagen amount left in the NHDF cells after the infection and the treatment with or without the hydroxamates. The yellow background in **a** highlighted that the cells were infected with *B. cereus*. Ctrl represents the non-infected cells (grey column) and the infected cells and non-treated with inhibitors (red column). **b)** Gelatin zymograms of collagenases produced by *Bacillus cereus* during the treatment with and without the hydroxamates. The DMEM medium of the infected NHDF cells were applied on the zymograms. Clear regions against blue background indicate gelatin in the gel has been cleaved. One-way ANOVA was used for statistical analysis, and the Tukey test was used to determine statistical significance. The significance of the results was determined by comparing non-treated *vs* compound-treated cells (mean ± SD, ns non-significant). M (kDa): molecular weight standard.

Stability of the hydroxamate compound 27 in the conditions of NHDF infection model

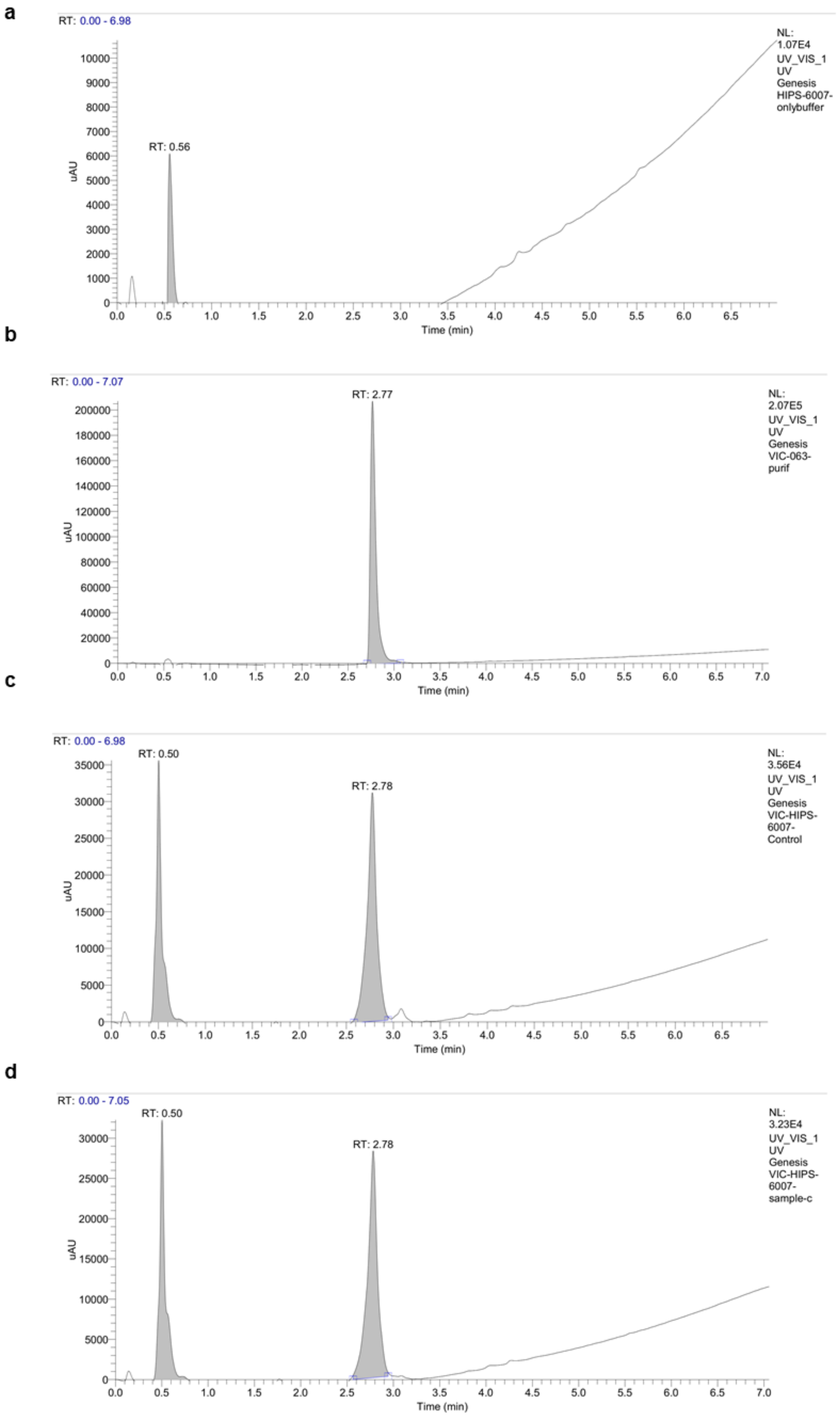

Figure S13. HPLC-MS UV spectra of a) DMEM medium control **b)** compound **27** in DMSO and without incubation **c)** compound **27** in DMEM medium without incubation **d)** compound **27** in DMEM medium with incubation at 37 °C for 5 h. No new peak was detected indicating no compound degradation under the assay conditions.

Reversibility assay results

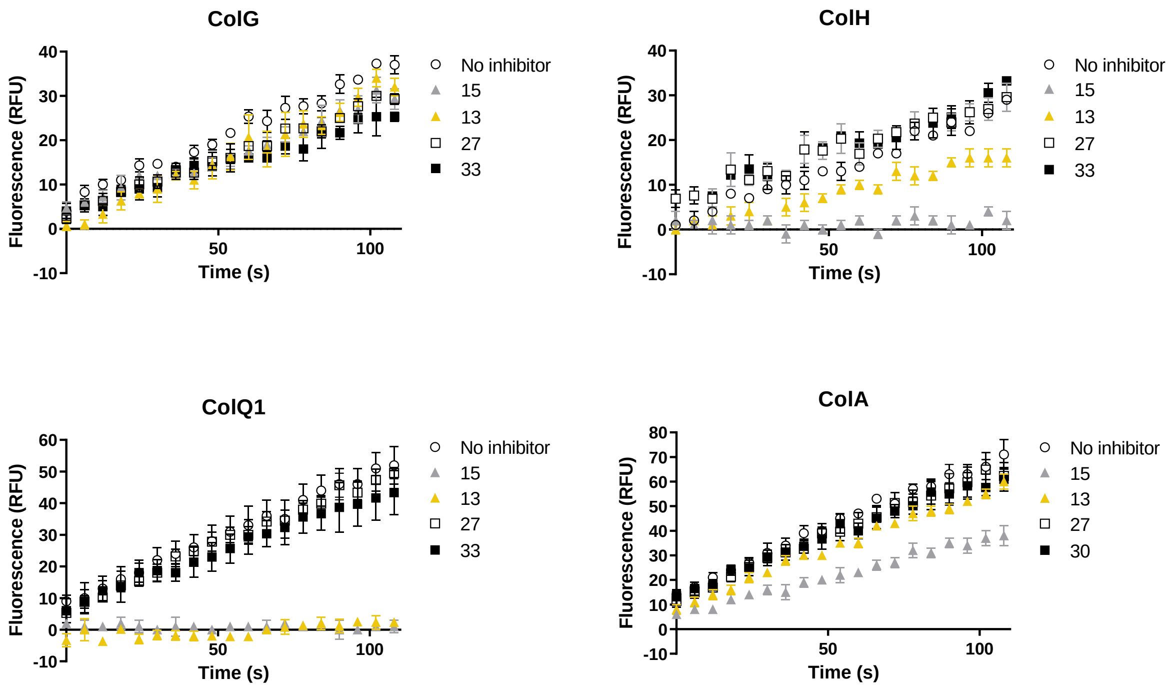

Figure S14. Reversibility of inhibition of ColG, ColH, ColQ1 and ColA by the hydroxamates 27 and 33 and the diphosphonates 13 and 15. Rapid dilution assay to test reversibility of peptidase interaction with compounds. Recovery of enzymatic activity was measured after incubation of each enzyme in the absence or presence of inhibitor. Progress curves were monitored for 2 min after 1:100 dilution of the enzyme-inhibitor complex and after addition of substrate to initiate the reaction. ColG, ColH, ColQ1, and ColA: collagenase G, H, Q1, and A.

Mass spectrometric analysis of protein-inhibitor interactions

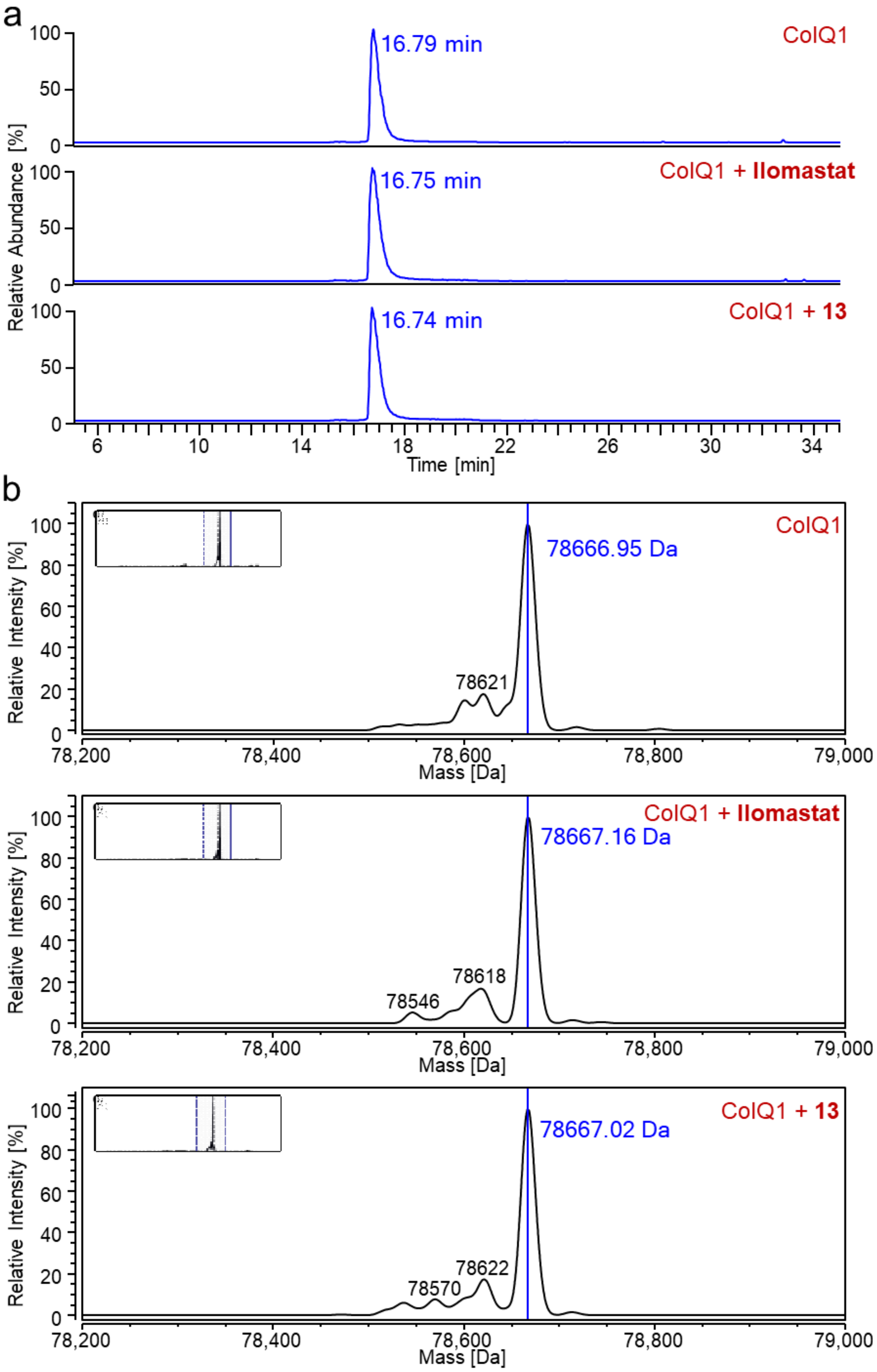

Figure S15. HPLC-MS analysis of ColQ1-CU-ligand interactions. a) Representative total ion current chromatograms of the collagenase unit of ColQ1 incubated with either no inhibitor, Ilomastat (a known reversible inhibitor), or **13**. The retention times of these three samples are indicated. **b)** The corresponding deconvoluted mass spectra of the collagenase unit of ColQ1 treated with the respective inhibitors as well as the control. The average mass of the main mass observed for ColQ1 was 78667.04 ± 0.1 Da in all cases and fits to the expected non-modified protein mass. No molecular masses displayed a mass shift indicating a covalently bound inhibitor.

Effect of the diphosphonates on the TEER of epithelial cells

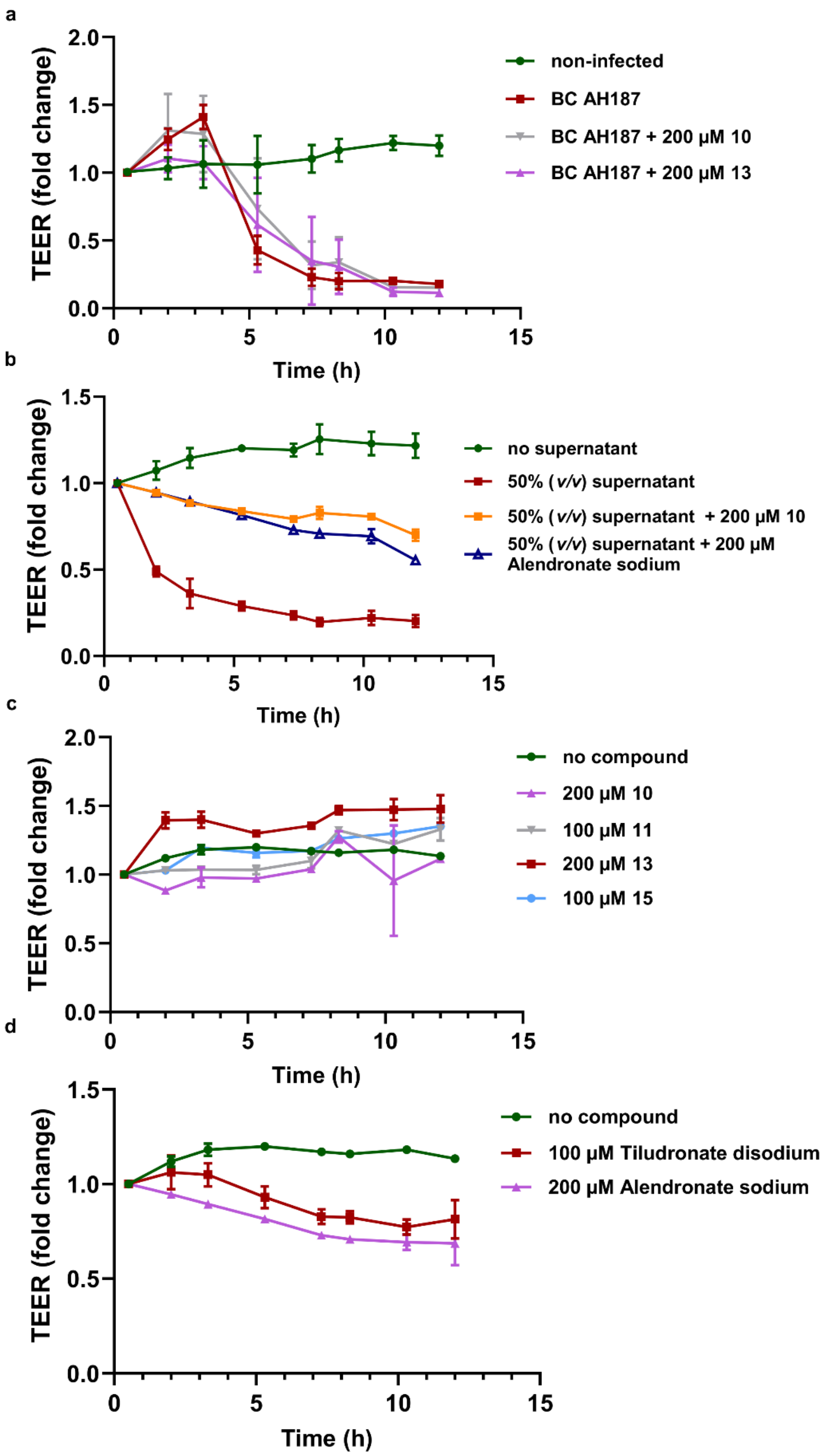

Figure S16. The effect of ColQ1 inhibitors on the transepithelial electrical resistance (TEER) of Madin–Darby Canine Kidney (MDCK) II cells. a) Effect of **10** and **13** on the TEER of MDCK cells infected with *Bacillus cereus* AH187. **b)** The influence of **10** and **alendronate sodium** on the TEER of non-infected and challenged with 50% (*v/v*) AH187 supernatant MDCK II cells. **c)** Effect of the synthesized diphosphonate compounds (**10, 11, 13,** and **15**) on the TEER of non-infected MDCK cells. **d)** The influence of the FDA-approved

drugs (**tiludronate disodium** and **alendronate sodium**) on the TEER of non-infected MDCK cells. Each curve represents average ± standard deviation of three independent experiments for **a** and **b** and two independent experiments for **c** and **d**.

Galleria mellonella infection model

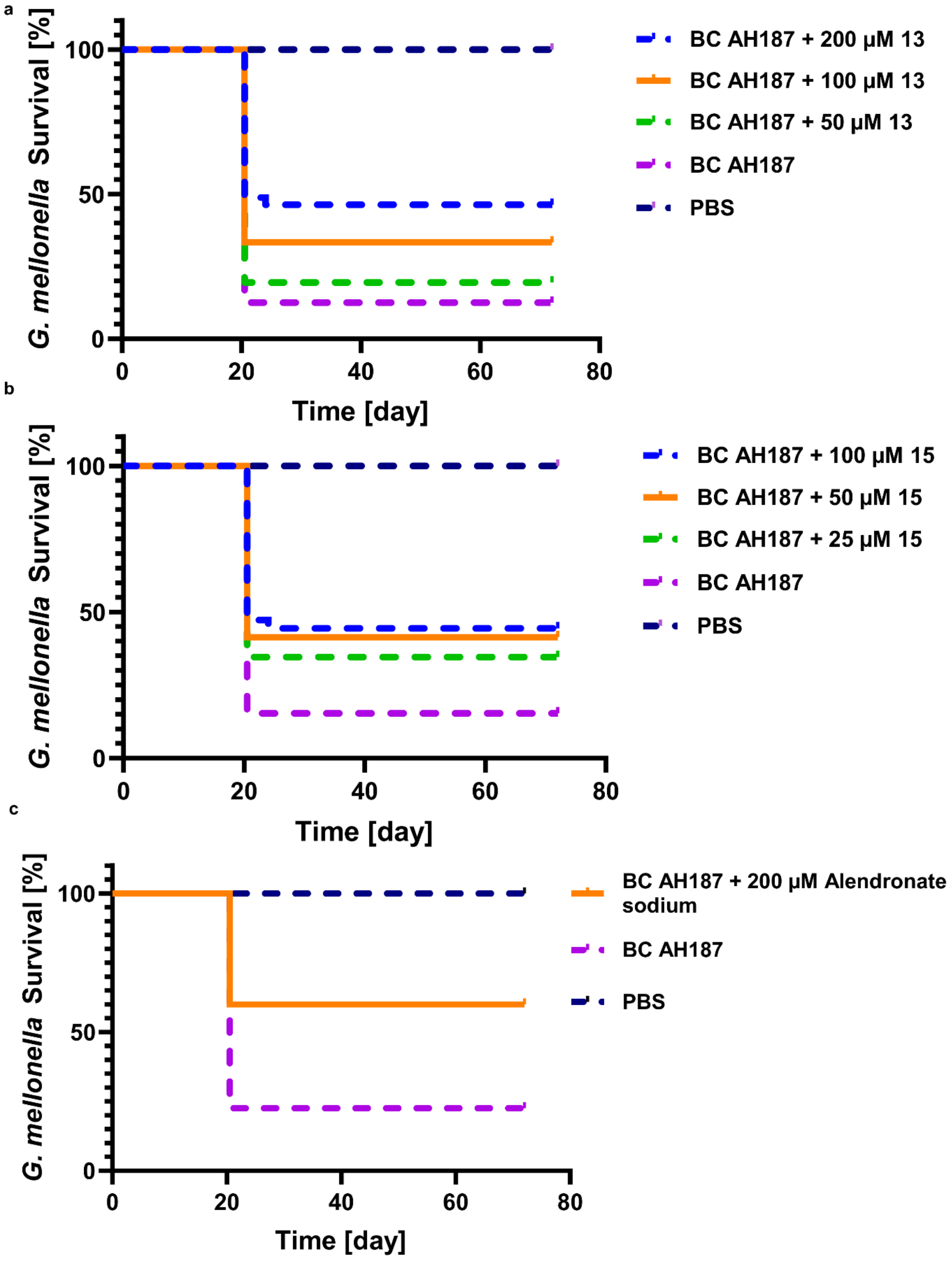

Figure S17. Probability of survival of the *Bacillus cereus*-infected *Galleria mellonella* larvae treated with and without compounds 13, 15, and alendronate sodium. a) The survival analysis of the infected larvae treated

with and without compound **13** various concentrations (50–200 µM) and **b)** compound **15** (25–100 µM). **c)** FDA-approved diphosphonate **alendronate sodium** at 200 µM. Each curve represents results of three independent experiments, statistical difference between groups treated with 200, 100 and 50 µM of compound **13** and with *B. cereus* AH187 and treated with only *B. cereus* AH187 is $p = 0.0008$, $p = 0.0283$ and $p = 0.393$, respectively. While, between groups treated with 100, 50, and 25 µM of **15** and treated with only *Bacillus cereus* AH187 is $p = 0.0149$, $p = 0.0357$ and $p = 0.1128$, respectively. The statistical difference with 200 µM **alendronate sodium** and with *B. cereus* AH187 is $p = 0.0032$ (log-rank test). The survival rate for the larvae treated with all tested compounds in PBS was 100%.

Chemistry

Diphosphonates

Diethyl (6,7-dichloro-3-oxo-3,4-dihydroquinoxalin-2-yl)phosphonate (16b). Compound **10b** was

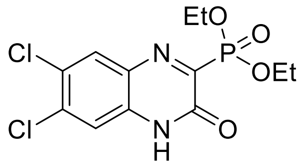

synthesized according to general procedure C, using 3,6,7-trichloroquinoxalin-2(1H)-one **16a** (112 mg, 0.45 mmol) and triethyl phosphite (819 µL, 4.5 mmol). The residue was purified by automated column chromatography (Hex/EtOAc = 1/1) to give the desired product (brown solid, 94 mg, 59%). ^{1}H NMR (500 MHz, DMSO) δ 12.90 (s, 1H), 8.05 (s, 1H), 7.44 (s, 1H), 4.49 (dd, *J* = 14.6, 7.4 Hz, 4H), 1.45 (t, *J* = 7.3 Hz, 6H). MS (ESI^{+}) m/z 350.9 [M+H]$^{+}$.

Tetraethyl (6-fluoroquinoxaline-2,3-diyl)bis(phosphonate) (8c). Compound **8c** was synthesized

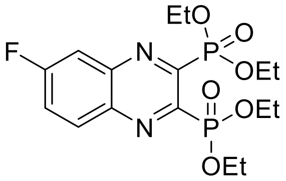

according to general procedure C, using 2,3-dichloro-6-fluoroquinoxaline **8b** (90 mg, 0.41 mmol) and triethyl phosphite (965 µL, 5.3 mmol). The residue was purified by automated column chromatography (Hex/EtOAc = 1:1) to give the desired product (white solid, 128 mg, 73%). ^{1}H NMR (500 MHz, $CDCl_3$) δ 8.16 (dd, *J* = 9.2, 5.6 Hz, 1H), 7.75 (dd, *J* = 8.6, 2.7 Hz, 1H), 7.68–7.52 (m, 1H), 4.58–3.99 (m, 8H), 1.38 (t, *J* = 7.1 Hz, 12H). ^{13}C NMR (126 MHz, $CDCl_3$) δ 164.1 (d, J_{C-F} = 257.4 Hz), 151.1 (dd, J_{C-P} = 202.4, 22.2 Hz), 149.3 (dd, J_{C-P} = 204.9, 22.7 Hz), 143.1–140.1 (m), 138.1 (d, J_{C-F} = 20.0 Hz), 132.2 (d, J_{C-F} = 9.6 Hz), 123.2 (d, J_{C-F} = 26.2 Hz), 113.2 (d, J_{C-F} = 21.5 Hz), 64.1 (d, J_{C-P} = 5.4 Hz), 64.0 (d, J_{C-P} = 6.3 Hz), 16.3 (d, J_{C-P} = 3.4 Hz). MS (ESI^{+}) m/z 421.08 [M+H]$^{+}$.

Tetraethyl (6-chloroquinoxaline-2,3-diyl)bis(phosphonate) (9c). Compound **9c** was

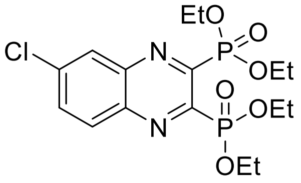

synthesized according to general procedure C, using 2,3,6-trichloroquinoxaline **9b** (80 mg, 0.34 mmol) and triethyl phosphite (619 µL, 3.4 mmol). The residue was purified by automated column chromatography (Hex/EtOAc = 1:1) to give the desired product (white solid, 120 mg, 80%). ^{1}H NMR (500 MHz, $CDCl_3$) δ 8.11 (d, *J* = 2.2 Hz, 1H), 8.08 (d, *J* = 9.0 Hz, 1H), 7.78 (dd, *J* = 9.0, 2.3 Hz, 1H), 4.34 (ddd, *J* = 11.2, 8.4, 2.2 Hz, 8H), 1.37 (td, *J* = 7.1, 2.1 Hz, 12H). ^{13}C NMR (126 MHz, $CDCl_3$) δ 151.5 (dd, J_{C-P} = 114.6, 29.7 Hz), 149.7 (dd, J_{C-P} = 115.7, 29.7 Hz), 140.7 (dd, J_{C-P} = 19.9, 2.4 Hz), 139.1 (dd, J_{C-P} = 20.0, 2.5 Hz), 138.4, 133.4, 130.9, 128.4, 64.1–63.7 (m), 16.3 (d, J_{C-P} = 5.7 Hz), 16.2 (d, J_{C-P} = 6.2 Hz). MS (ESI^{+}) m/z 437.06 [M+H]$^{+}$.

Tetraethyl (6-bromoquinoxaline-2,3-diyl)bis(phosphonate) (10c). Compound **10c** was synthesized

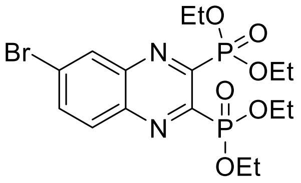

according to general procedure C, using 6-bromo-2,3-dichloroquinoxaline **10b** (75 mg, 0.27 mmol) and triethyl phosphite (491 µL, 2.7 mmol). The residue was purified by automated column chromatography (Hex/EtOAc = 1:1) to give the desired product (white solid, 89 mg, 68%). ^{1}H NMR (500

MHz, $CDCl_3$) δ 8.30 (d, *J* = 2.0 Hz, 1H), 8.01 (d, *J* = 9.0 Hz, 1H), 7.92 (dd, *J* = 9.0, 2.1 Hz, 1H), 4.38–4.28 (m, 8H), 1.37 (td, *J* = 7.1, 2.3 Hz, 12H). MS (ESI^+) m/z 481.07 $[M+H]^+$.

Tetraethyl (6-methylquinoxaline-2,3-diyl)bis(phosphonate) (11c). Compound **11c** was

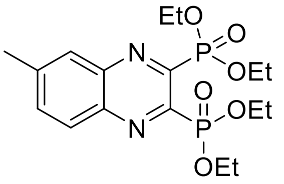

synthesized according to general procedure C, using 2,3-dichloro-6-methylquinoxaline **11b** (90 mg, 0.42 mmol) and triethyl phosphite (765 µL, 4.2 mmol). The residue was purified by automated column chromatography (Hex/EtOAc = 1:1) to give the desired product (white solid, 123 mg, 70%). ^{1}H NMR (500 MHz, $CDCl_3$) δ 8.02 (d, *J* = 8.6 Hz, 1H), 7.90 (s, 1H), 7.66 (dd, *J* = 8.6, 1.7 Hz, 1H), 4.39–4.30 (m, 8H), 2.57 (s, 3H), 1.38 (td, *J* = 7.1, 2.1 Hz, 12H). ^{13}C NMR (126 MHz, $CDCl_3$) δ 150.3 (dd, $J_{C\text{-}P}$ = 126.1, 29.8 Hz), 148.5 (dd, $J_{C\text{-}P}$ = 126.9, 30.1 Hz), 143.6 , 140.8 (dd, $J_{C\text{-}P}$ = 19.7, 2.3 Hz), 139.3 (dd, $J_{C\text{-}P}$ = 19.5, 2.6 Hz), 134.8 , 129.3, 128.5, 66.5–60.9 (m), 22.0, 16.3 (d, $J_{C\text{-}P}$ = 6.2 Hz). ^{31}P NMR (202 MHz, $CDCl_3$) δ 7.33 (d, *J* = 9.1 Hz), 7.13 (d, *J* = 8.0 Hz). MS (ESI^+) m/z 417.12 $[M+H]^+$.

Tetraethyl (6-methoxyquinoxaline-2,3-diyl)bis(phosphonate) (12c). Compound **12c** was synthesized

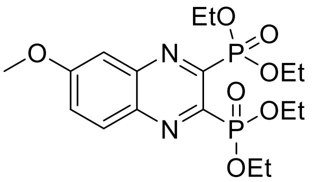

according to general procedure C, using 2,3-dichloro-6-methoxyquinoxaline **12b** (70 mg, 0.30 mmol) and triethyl phosphite (546 µL, 3.0 mmol). The residue was purified by automated column chromatography (Hex/EtOAc = 1:1) to give the desired product (white solid, 100 mg, 75%). ^{1}H NMR (500 MHz, $CDCl_3$) δ 8.06 (d, *J* = 9.2 Hz, 1H), 7.53 (dd, *J* = 9.2, 2.8 Hz, 1H), 7.44 (d, *J* = 2.7 Hz, 1H), 4.48–4.31 (m, 8H), 4.00 (s, 3H), 1.43 (t, *J* = 7.1 Hz, 12H). ^{13}C NMR (126 MHz, $CDCl_3$) δ 162.7, 149.9 (dd, $J_{C\text{-}P}$ = 227.6, 28.8 Hz), 146.9 (dd, $J_{C\text{-}P}$ = 230.4, 29.0 Hz), 142.8 (d, $J_{C\text{-}P}$ = 20.1 Hz), 137.3 (d, $J_{C\text{-}P}$ = 20.0 Hz), 130.9, 126.4, 106.6, 63.8 (d, $J_{C\text{-}P}$ = 3.2 Hz), 63.8 (d, $J_{C\text{-}P}$ = 3.2 Hz), 56.1, 16.4 (d, $J_{C\text{-}P}$ = 3.6 Hz), 16.3 (d, $J_{C\text{-}P}$ = 3.7 Hz). ^{31}P NMR (202 MHz, $CDCl_3$) δ 7.91 (d, *J* = 9.8 Hz), 7.48 (d, *J* = 8.4 Hz). MS (ESI^+) m/z 433.13 $[M+H]^+$.

Tetraethyl (6,7-dichloroquinoxaline-2,3-diyl)bis(phosphonate) (13c). Compound **13c** was

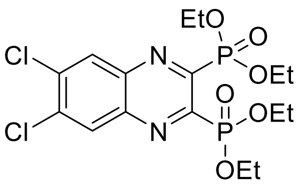

synthesized according to general procedure C, using 2,3,6,7-tetrachloroquinoxaline **13b** (80 mg, 0.29 mmol) and triethyl phosphite (528 µL, 2.9 mmol). The residue was purified by automated column chromatography (Hex/EtOAc = 1:1) to give the desired product (white solid, 89 mg, 63%). ^{1}H NMR (500 MHz, CDCl3) (δ, ppm): 8.12 (s, 2H), 4.34 (ddd, *J* = 11.2, 8.4, 2.2 Hz, 8H), 1.37 (td, *J* = 7.1, 2.1 Hz, 12H). MS (ESI^+) m/z 471.01 $[M]^+$.

Tetraethyl (6-((3,4-dichlorophenyl)carbamoyl)quinoxaline-2,3-diyl)bis(phosphonate) (14d).

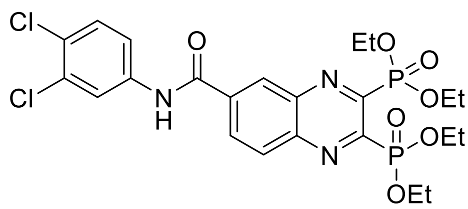

Compound **14d** was synthesized according to general procedure C, using 2,3-dichloro-N-(3,4-dichlorophenyl)quinoxaline-6-carboxamide **14c** (100 mg, 0.26 mmol) and triethyl phosphite (473 µL, 2.6 mmol). The residue was purified by automated column chromatography (Hex/EtOAc = 1:1) to give the desired product (white solid, 117 mg, 77%). ^{1}H NMR (500 MHz, $CDCl_3$) δ 10.57 (s, 1H), 8.57 (s, 1H), 8.32 (dd, J = 8.7, 1.5 Hz, 1H), 8.10 (d, J = 2.3 Hz, 1H), 7.86 (dd, J = 8.8, 2.3 Hz, 1H), 7.82 (d, J = 8.7 Hz, 1H), 7.36 (d, J = 8.7 Hz, 1H), 4.35–4.22 (m, 8H), 1.37–1.33 (m, 12H). ^{13}C NMR (126 MHz, $CDCl_3$) δ 164.9, 151.3 (dd, $J_{C\text{-}P}$ = 51.3, 29.7 Hz), 149.5 (dd, $J_{C\text{-}P}$ = 51.7, 29.6 Hz), 141.1 (dd, J = 19.7, 2.3 Hz), 139.5 (dd, $J_{C\text{-}P}$ = 20.1, 2.5 Hz), 138.9, 138.6, 132.3, 131.8, 130.3, 129.5, 129.3, 127.3, 122.1, 119.8, 64.4 (d, $J_{C\text{-}P}$ = 6.9 Hz), 63.6 (d, $J_{C\text{-}P}$ = 5.7 Hz), 61.8 (d, $J_{C\text{-}P}$ = 5.6 Hz), 61.5 (d, $J_{C\text{-}P}$ = 6.5 Hz), 16.4–16.2 (m), 16.1–15.9 (m). ^{31}P NMR (202 MHz, $CDCl_3$) δ 7.26, 5.92. MS (ESI$^+$) m/z 590.14 $[M+H]^+$.

Tetraethyl (6-(4-chlorophenyl)quinoxaline-2,3-diyl)bis(phosphonate) (15d). Compound

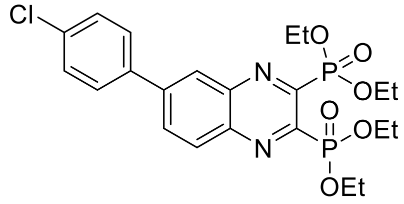

15d was synthesized according to general procedure C, using 2,3-dichloro-6-(4-chlorophenyl)quinoxaline **15c** (60 mg, 0.19 mmol) and triethyl phosphite (346 µL, 1.9 mmol). The residue was purified by automated column chromatography (Hex/EtOAc = 1:1) to give the desired product (white solid, 76 mg, 76%). ^{1}H NMR (500 MHz, $CDCl_3$) δ 7.98 (d, J = 1.8 Hz, 1H), 7.89 (d, J = 8.8 Hz, 1H), 7.82 (dd, J = 8.8, 2.0 Hz, 1H), 7.37 (d, J = 8.5 Hz, 2H), 7.12 (d, J = 8.5 Hz, 2H), 4.09–4.03 (m, 8H), 1.08 (t, J = 7.0 Hz, 12H). ^{13}C NMR (126 MHz, $CDCl_3$) δ 150.8 (dd, $J_{C\text{-}P}$ = 101.2, 30.0 Hz), 149.0 (dd, $J_{C\text{-}P}$ = 102.1, 29.9 Hz), 143.5, 140.5 (dd, $J_{C\text{-}P}$ = 19.3, 1.7 Hz), 139.7 (dd, $J_{C\text{-}P}$ = 19.3, 1.7 Hz), 136.8, 134.7, 131.5, 130.0, 129.0, 128.5, 126.4, 63.1 (d, $J_{C\text{-}P}$ = 5.7 Hz), 61.2 (d, $J_{C\text{-}P}$ = 5.6 Hz), 15.8 (d, $J_{C\text{-}P}$ = 6.2 Hz), 15.6 (d, $J_{C\text{-}P}$ = 6.8 Hz). MS (ESI$^+$) m/z 513.18 $[M+H]^+$.

Hydroxamates

Ethyl 2-benzyl-3-[2-(tert-butoxycarbonylamino)ethylamino]-3-oxo-propanoate (23b).

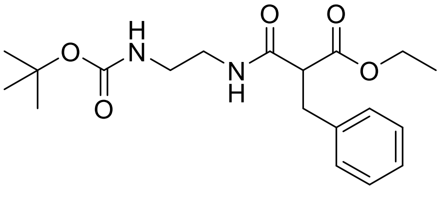

Compound **23b** was synthesized according to the general procedure B2, using carboxylic acid **22b** (340 mg, 1.5 mmol), *tert*-butyl *N*-(2-aminoethyl)carbamate (270 mg, 1.7 mmol), HOBt (23.4 mg, 0.1 mmol), EDC.HCl (440 mg, 2.3 mmol) and diisopropylethylamine (5560 µL, 31.8 mmol) in CH_2Cl_2 (7 mL) overnight. The crude product was purified by flash chromatography on silica gel (cHex to cHex/EtOAc 6:4) affording compound **23b** as a white solid (323 mg, 57%). ^{1}H NMR (500

MHz, DMSO-d_6) δ: 8.10 (t, J = 5.7 Hz, 1H), 7.27–7.24 (m, 2H), 7.19–7.17 (m, 2H), 6.62 (t, J = 5.5 Hz, 1H), 4.03 (q, J = 7.1 Hz, 2H), 3.54 (t, J = 7.7 Hz, 1H), 3.08–2.95 (m, 4H), 2.89–2.82 (m, 2H), 1.37 (s, 9H), 1.11 (t, J = 7.1 Hz, 3H). ^{13}C NMR (126 MHz, DMSO-d_6) δ: 169.3, 167.7, 155.6, 138.7, 128.8 (2C), 128.2 (2C), 126.3, 77.8, 60.5, 53.6, 39.4, 38.8, 34.3, 28.3 (3C), 14.0. MS (ESI$^+$): m/z= 365 [M+H]$^+$, 309 [M–tBu+H]$^+$, 265 [M–Boc+H]$^+$.

2-[(2-Benzyl-3-ethoxy-3-oxo-propanoyl)amino]ethylammonium;chloride (24b).

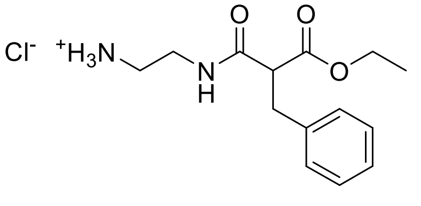

Compound **24b** was synthesized according to the general procedure C2, using the Boc-protected intermediate **23b** (315 mg, 0.9 mmol), 4 N HCl in dioxane (5 mL) in a mixture CH_2Cl_2/EtOH (10 mL, 5:5 *v/v*) overnight. Compound **24b** was obtained as a pale yellow oil (260 mg, quant. yield) and was used in the next step without further purification. ^{1}H NMR (500 MHz, DMSO-d_6) δ: 8.51 (t, J = 5.5 Hz, 1H), 8.08 (br s, 3H), 7.28–7.17 (m, 5H), 4.03 (q, J = 7.2 Hz, 2H), 3.61 (t, J = 7.8 Hz, 1H), 3.34–3.27 (m, 1H), 3.25–3.18 (m, 1H), 3.05–3.04 (m, 2H), 2.75–2.63 (m, 2H), 1.10 (t, J = 7.1 Hz, 3H). 0.86 (d, J = 6.5 Hz, 3H), 0.85 (d, J = 6.5 Hz, 3H). ^{13}C NMR (75 MHz, DMSO-d_6) δ: 169.9, 168.8, 60.5, 50.2, 38.2, 37.3, 36.6, 25.6, 22.4, 22.2, 14.0. MS (ESI$^+$): m/z= 231 [M+H]$^+$.

Ethyl 3-(2-azidoethylamino)-2-benzyl-3-oxo-propanoate (25b). Compound **25b** was synthesized according to the general procedure D2, using amine **24b** (250 mg, 0.8 mmol), $ZnCl_2$ (6.8 mg, 0.05 mmol), K_2CO_3 (115 mg, 0.8 mmol), diisopropylethylamine (507 µL, 2.9 mmol) and diazo transfer reagent (210 mg, 1.0 mmol) in EtOH (4 mL). The mixture was stirred at room temperature overnight. The crude product was purified by flash chromatography on silica gel (cHex/EtOAc: 9/1 to 6/4) affording compound **25b** as colorless oil (241 mg, 99%). ^{1}H NMR (500 MHz, $CDCl_3$) δ: 7.30–7.27 (m, 2H), 7.24–7.21 (m, 1H), 7.19–7.17 (m, 2H), 6.78–6.76 (m, 1H), 4.13–4.09 (m, 2H), 3.49 (dd, J = 8.5 and 6.7 Hz, 1H), 3.41–3.37 (m, 4H), 3.26 (dd, J = 13.7 and 6.7 Hz, 1H), 3.18 (dd, J = 13.7 and 8.5 Hz, 1H), 1.15 (t, J = 7.1 Hz, 3H). ^{13}C NMR (126 MHz, $CDCl_3$) δ: 171.4, 168.2, 137.7, 129.0 (2C), 128.7 (2C), 127.1, 61.8, 54.9, 50.8, 39.1, 36.8, 14.1. MS (ESI$^+$): m/z= 291 [M+H]$^+$.

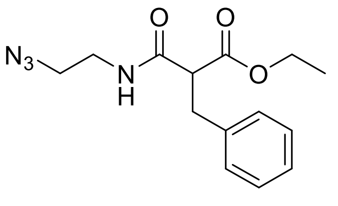

***N*-(2-Azidoethyl)-2-benzyl-3-(hydroxyamino)-3-oxo-propanamide (26b).** Compound **26b** was synthesized according to the general procedure E2, using ester **25b** (110 mg, 0.37 mmol), KCN (4.9 mg, 0.08 mmol) and aq. NH_2OH (2.5 mL, 50% *w/w* in water) in MeOH (2.5 mL) overnight. The crude product was purified by flash chromatography on silica

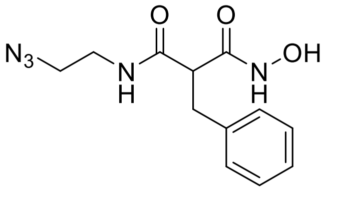

gel (CH_2Cl_2 to CH_2Cl_2/MeOH 9:1) affording compound **26b** as a white solid (53 mg, 51%). ^{1}H NMR (500 MHz, DMSO-d_6) δ: 10.41 (s, 1H), 8.91 (s, 1H), 7.94 (t, J = 5.5 Hz, 1H), 7.27–7.16 (m, 5H), 3.32–3.16 (m, 5H), 3.05–2.97 (m, 2H). ^{13}C NMR (126 MHz, DMSO-d_6) δ: 168.6, 165.6, 138.9, 128.7 (2C), 128.2 (2C), 126.2, 52.2, 49.8, 38.3, 34.8. MS (ESI$^+$): m/z= 278 $[M+H]^+$.

***N*-(3-Prop-2-ynoxyphenyl)acetamide (34b).** Compound **34b** was synthesized according to the

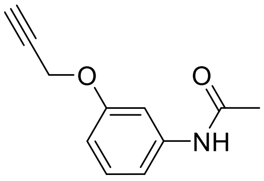

general procedure G2, using *N*-(3-hydroxyphenyl)acetamide (300 mg, 2.0 mmol), K_2CO_3 (550 mg, 4.0 mmol) and propargylbromide (207 µL, 2.2 mmol) in DMF (6 mL). The resulting solution was heated to 60 °C and stirred overnight. The mixture was then diluted in water and extracted 3x with ethyl acetate. The organic layers were combined and solvents were evaporated under reduced pressure affording compound **34b** as a brown solid (316 mg, 83%). ^{1}H NMR (500 MHz, DMSO-d_6) δ: 9.93 (s, 1H), 7.30 (t, J = 1.9 Hz, 1H), 7.20 (t, J = 8.0 Hz, 1H), 7.16–7.14 (m, 1H), 6.67–6.65 (m, 1H), 4.74 (d, J = 2.3 Hz, 2H), 3.56 (t, J = 2.3 Hz, 1H), 2.03 (s, 3H). ^{13}C NMR (126 MHz, DMSO-d_6) δ: 168.4, 157.4, 140.5, 129.5, 112.0, 109.0, 105.9, 79.3, 78.3, 55.3, 24.1. MS (ESI$^+$): m/z= 190 $[M+H]^+$.

***N*-(4-Prop-2-ynoxyphenyl)acetamide (34c).** Compound **34c** was synthesized according to the

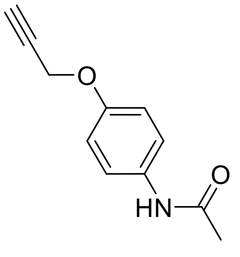

general procedure G2, using *N*-(4-hydroxyphenyl)acetamide (300 mg, 2.0 mmol), K_2CO_3 (550 mg, 4.0 mmol) and propargylbromide (207 µL, 2.2 mmol) in DMF (6 mL). The resulting solution was heated to 60 °C and stirred overnight. The mixture was then diluted in water and extracted three times with ethyl acetate. The organic layers were combined and solvents were evaporated under reduced pressure affording compound **34c** as a brown solid (372 mg, 98%). ^{1}H NMR (500 MHz, DMSO-d_6) δ: 9.80 (s, 1H), 7.49–7.47 (m, 2H), 6.92–6.90 (m, 2H), 4.73 (d, J = 2.3 Hz, 2H), 3.54 (t, J = 2.4 Hz, 1H), 2.00 (s, 3H). ^{13}C NMR (126 MHz, DMSO-d_6) δ: 167.8, 152.9, 133.2, 120.4 (2C), 114.9 (2C), 79.4, 78.1, 55.6, 23.8. MS (ESI$^+$): m/z= 190 $[M+H]^+$.

1-Fluoro-2-prop-2-ynoxy-benzene (34d). Compound **34d** was synthesized according to the

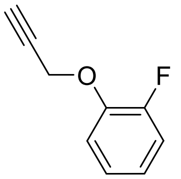

general procedure G2, using 2-fluorophenol (300 mg, 2.7 mmol), K_2CO_3 (740 mg, 5.4 mmol) and propargylbromide (279 µL, 2.9 mmol) in DMF (6 mL). The resulting solution was heated at 60°C and stirred overnight. The mixture was then diluted in water and extracted 3x with ethyl acetate. The organic layers were combined and solvents were evaporated under reduced pressure affording compound **34d**

as a brown oil (339 mg, 83%). ^{1}H NMR (500 MHz, DMSO-d_6) δ: 7.25–7.21 (m, 2H), 7.16–7.13 (m, 1H), 7.01–6.96 (m, 1H), 4.88 (d, J = 2.4 Hz, 2H), 3.62 (t, J = 2.4 Hz, 1H). ^{1}H NMR (500 MHz, DMSO-d_6) δ: 7.25–7.21 (m, 2H), 7.16–7.13 (m, 1H), 7.01–6.96 (m, 1H), 4.88 (d, J = 2.4 Hz, 2H), 3.62 (t, J = 2.4 Hz, 1H). ^{13}C NMR (126 MHz, DMSO-d_6) δ: 151.9 (d, J = 243.7 Hz), 145.0 (d, J = 10.7 Hz), 124.7 (d, J = 3.7 Hz), 121.9 (d, J = 6.4 Hz), 116.1 (d, J = 17.5 Hz), 115.7, 78.8, 78.8, 56.4. ^{19}F NMR (471 MHz, DMSO-d_6) δ: -134.8. MS (ESI$^+$): m/z= no ionization $[M+H]^+$.

NMR-Spectra

Diphosphonate compounds

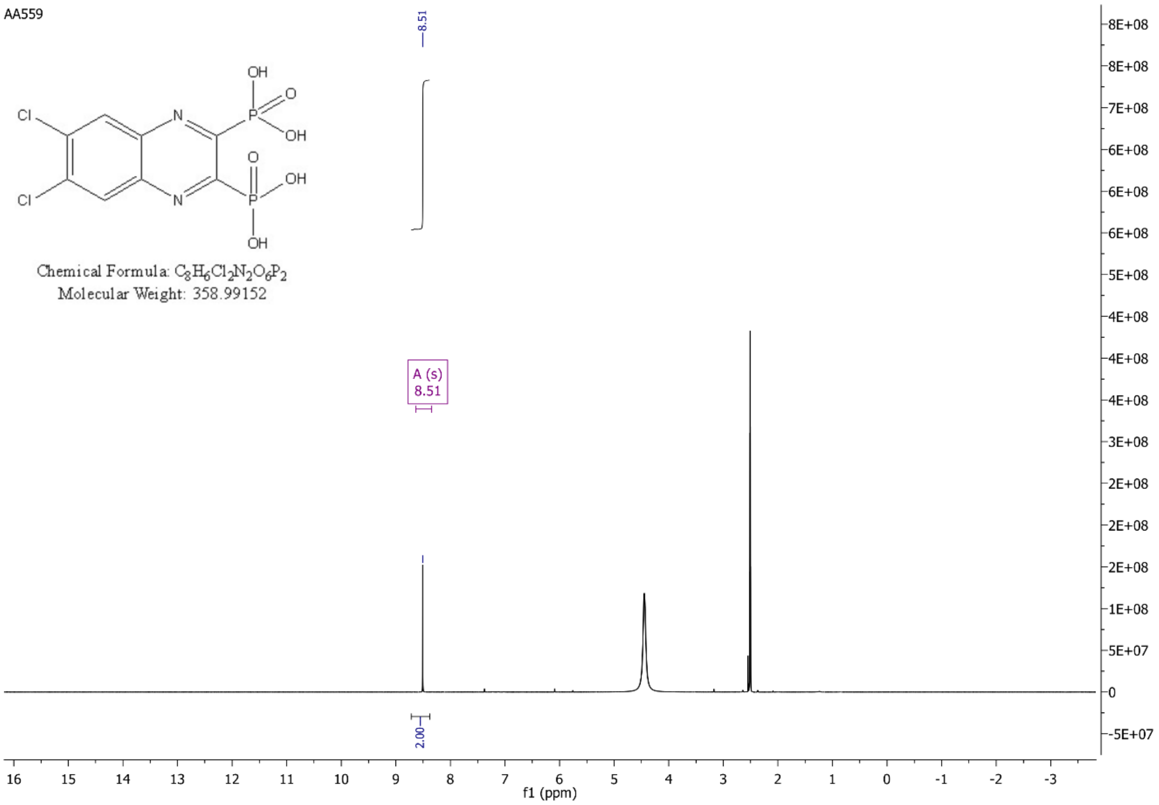

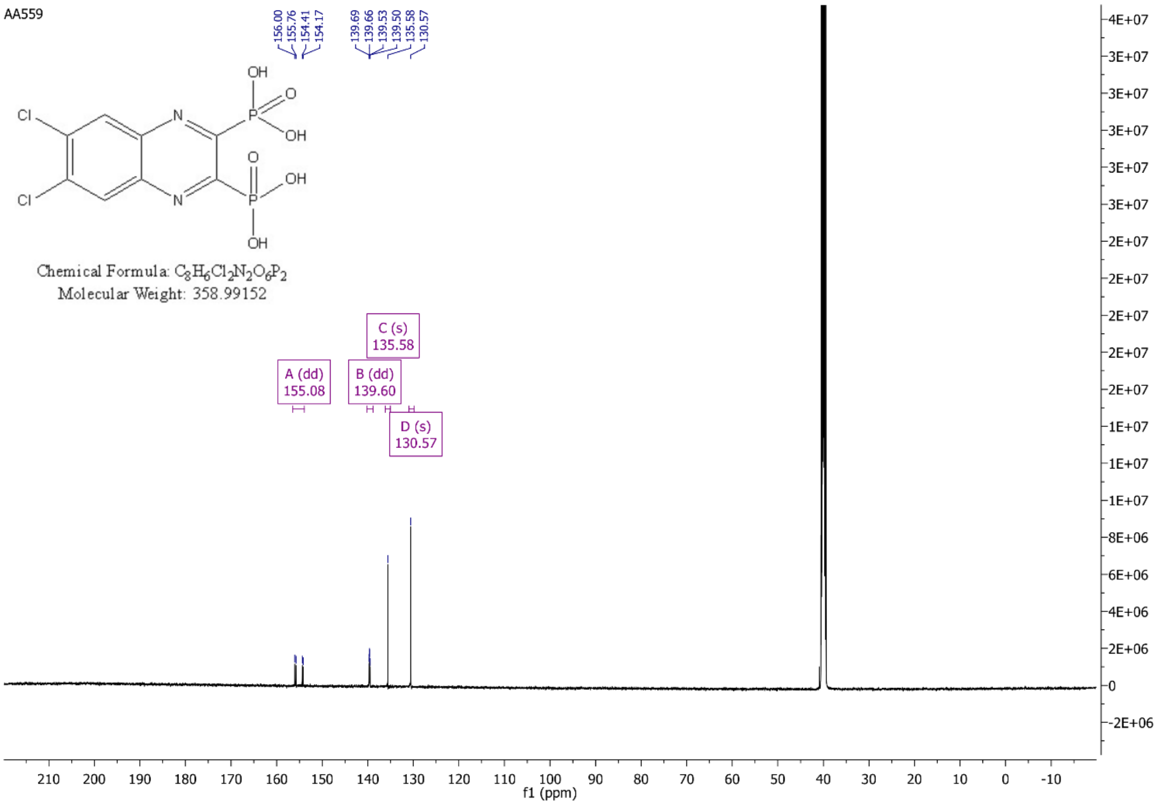

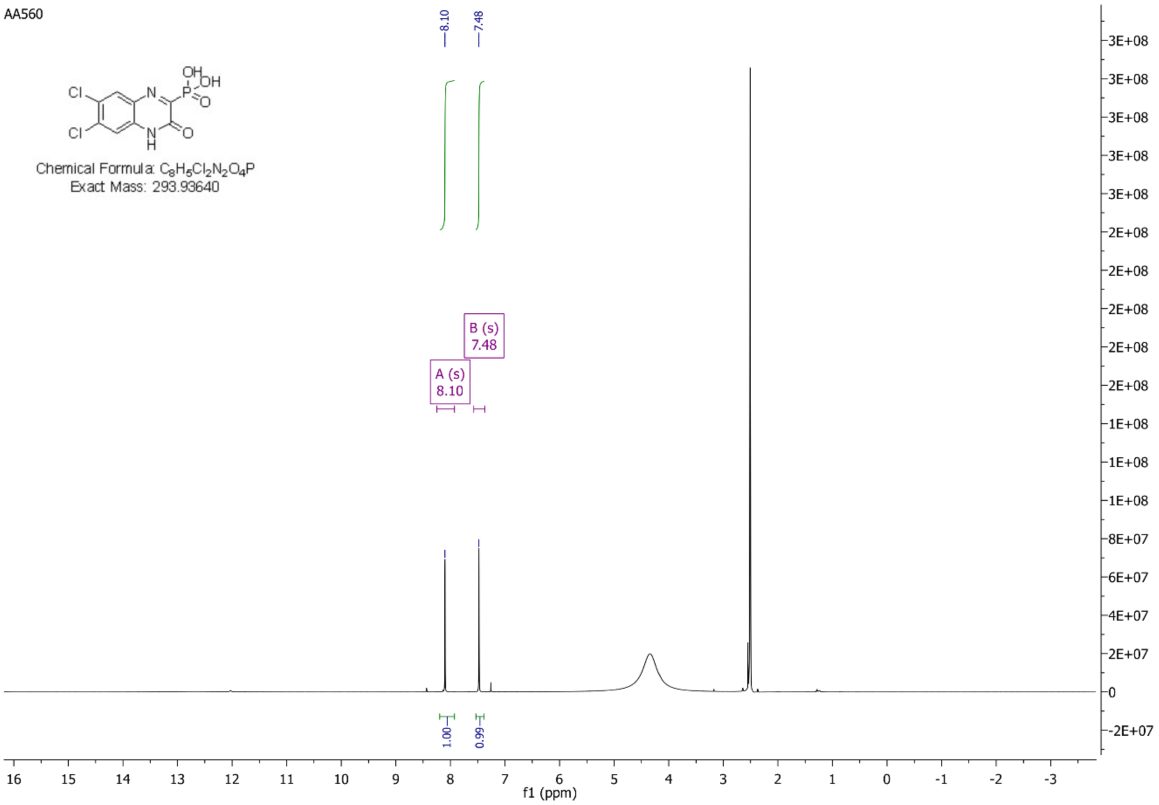
AA560
Chemical Formula: $C_8H_5Cl_2N_2O_4P$
Exact Mass: 293.93640
8.10
7.48
A (s)
8.10
B (s)
7.48
1.00
0.99
f1 (ppm)

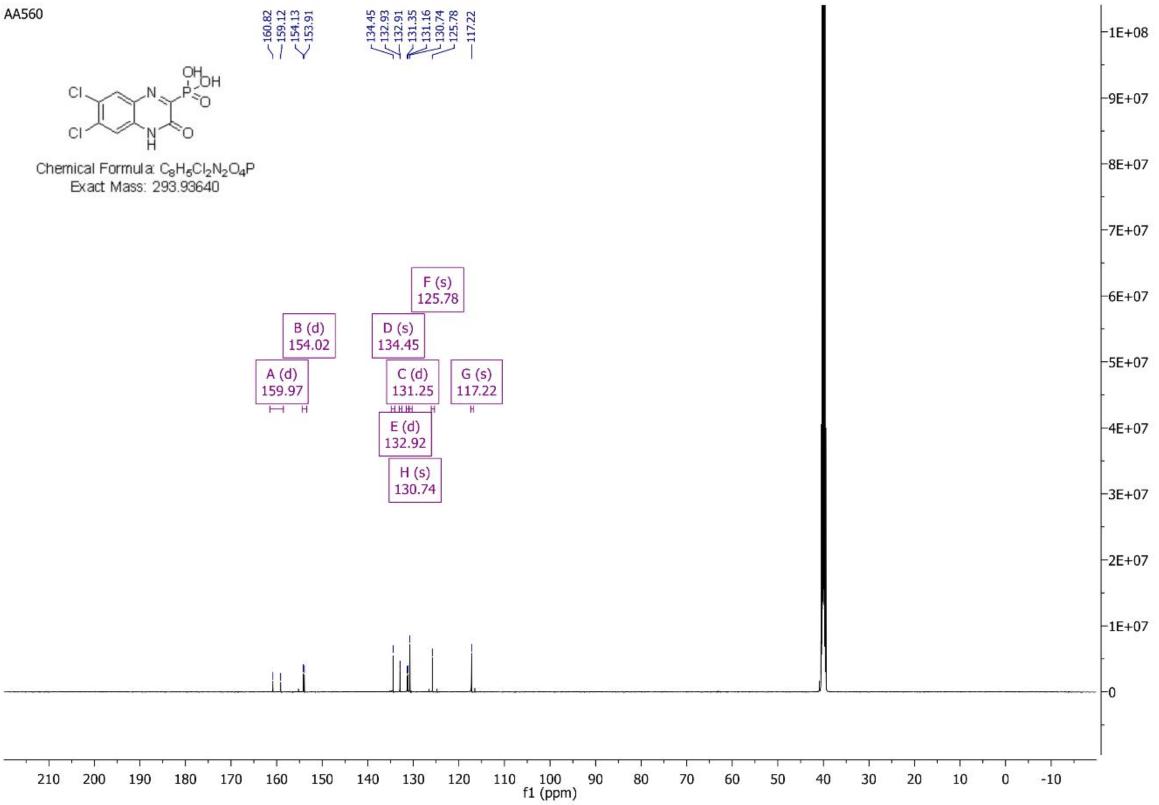
AA560
Chemical Formula: $C_8H_5Cl_2N_2O_4P$
Exact Mass: 293.93640
160.82
159.12
154.13
153.91
134.45
132.93
132.91
131.35
131.16
130.74
125.78
117.22
A (d)
159.97
B (d)
154.02
C (d)
131.25
D (s)
134.45
E (d)
132.92
F (s)
125.78
G (s)
117.22
H (s)
130.74
f1 (ppm)

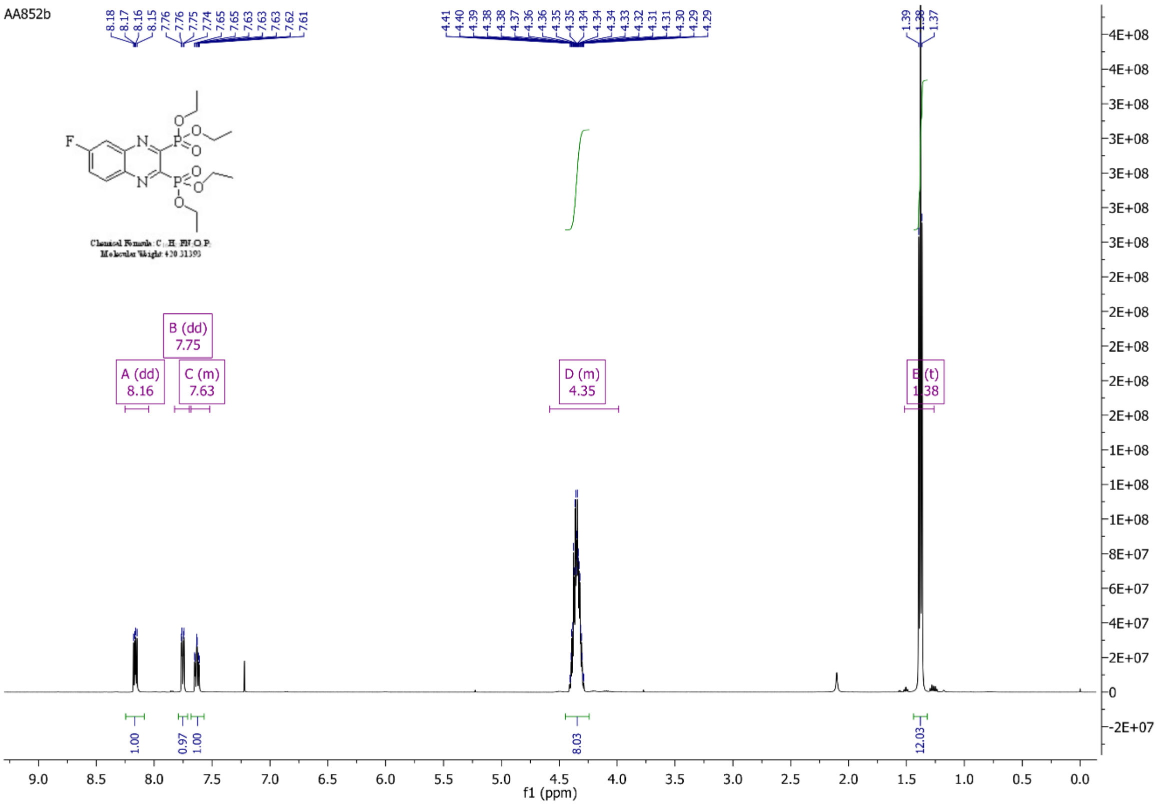

AA852b
A (dd)
8.16
B (dd)
7.75
C (m)
7.63
D (m)
4.35
E (t)
1.38
1.00
0.97
1.00
8.03
12.03
f1 (ppm)

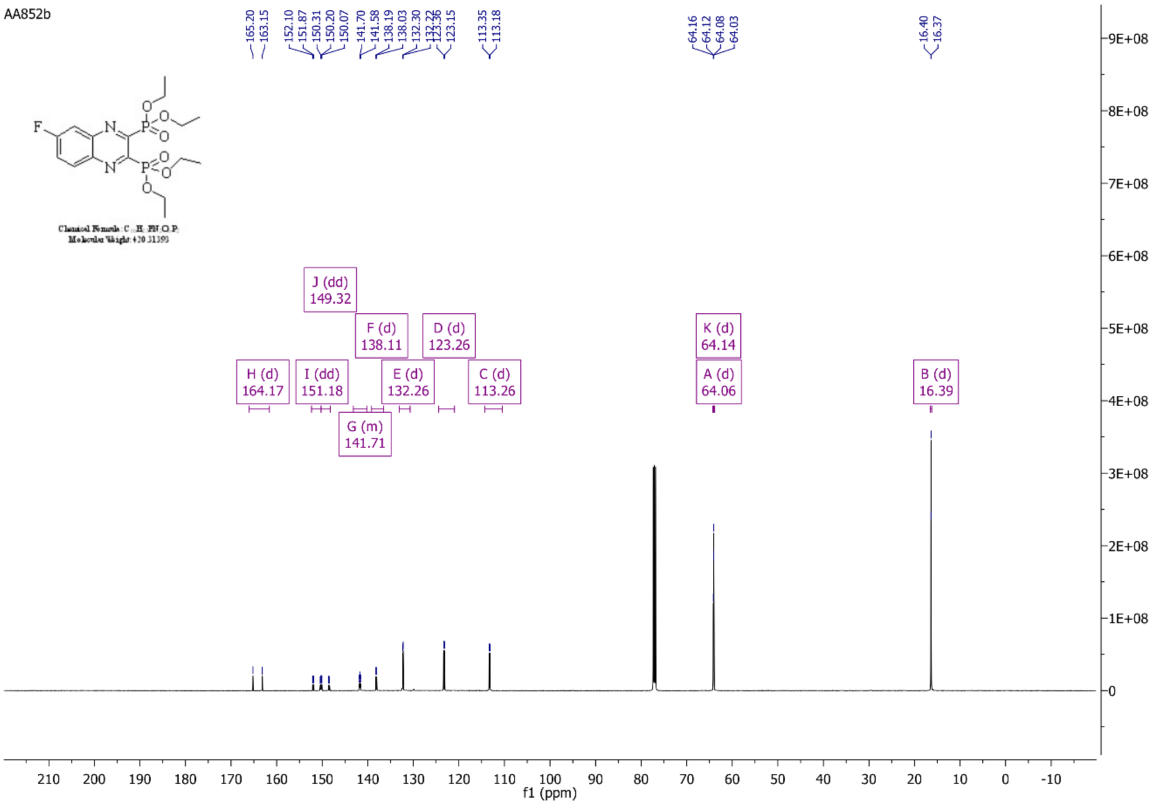

AA852b
J (dd)
149.32
F (d)
138.11
D (d)
123.26
H (d)
164.17
I (dd)
151.18
E (d)
132.26
C (d)
113.26
G (m)
141.71
K (d)
64.14
A (d)
64.06
B (d)
16.39
f1 (ppm)

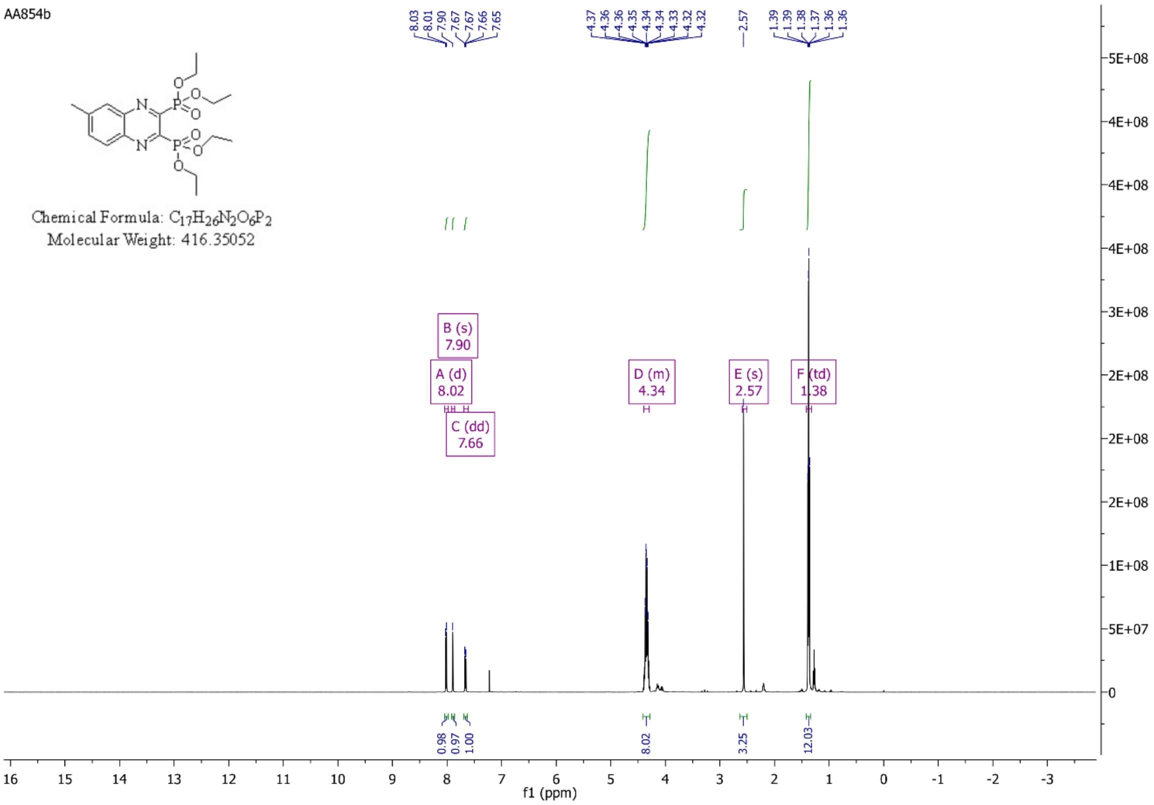
AA854b
8.03
8.01
7.90
7.67
7.67
7.66
7.65
4.37
4.36
4.36
4.35
4.34
4.34
4.34
4.33
4.32
4.32
2.57
1.39
1.39
1.38
1.37
1.36
1.36
Chemical Formula: $C_{17}H_{26}N_2O_6P_2$
Molecular Weight: 416.35052
B (s) 7.90
A (d) 8.02
C (dd) 7.66
D (m) 4.34
E (s) 2.57
F (td) 1.38
0.98
0.97
1.00
8.02
3.25
12.03
f1 (ppm)

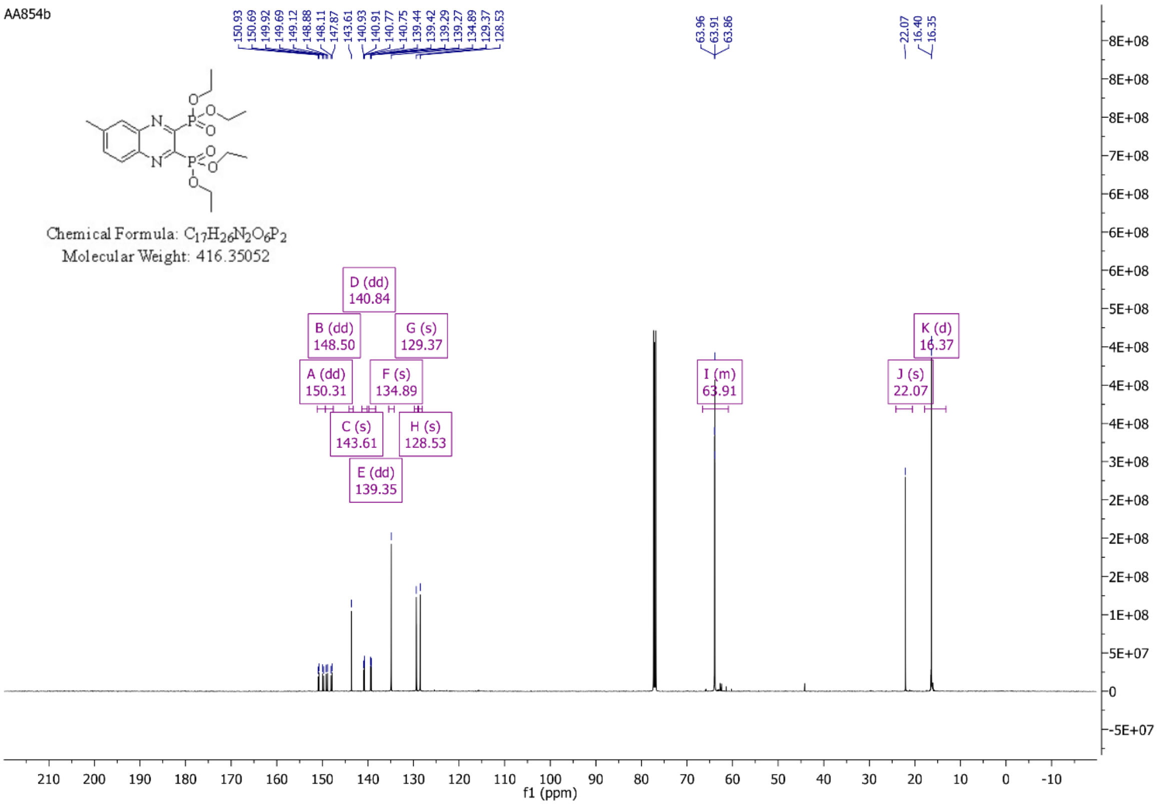
AA854b
150.93
150.69
149.92
149.69
149.12
148.88
148.11
147.87
143.61
140.93
140.91
140.77
140.75
139.44
139.42
139.29
139.27
134.89
129.37
128.53
63.96
63.91
63.86
22.07
16.40
16.35
Chemical Formula: $C_{17}H_{26}N_2O_6P_2$
Molecular Weight: 416.35052
D (dd) 140.84
B (dd) 148.50
G (s) 129.37
A (dd) 150.31
F (s) 134.89
C (s) 143.61
H (s) 128.53
E (dd) 139.35
I (m) 63.91
J (s) 22.07
K (d) 16.37
f1 (ppm)

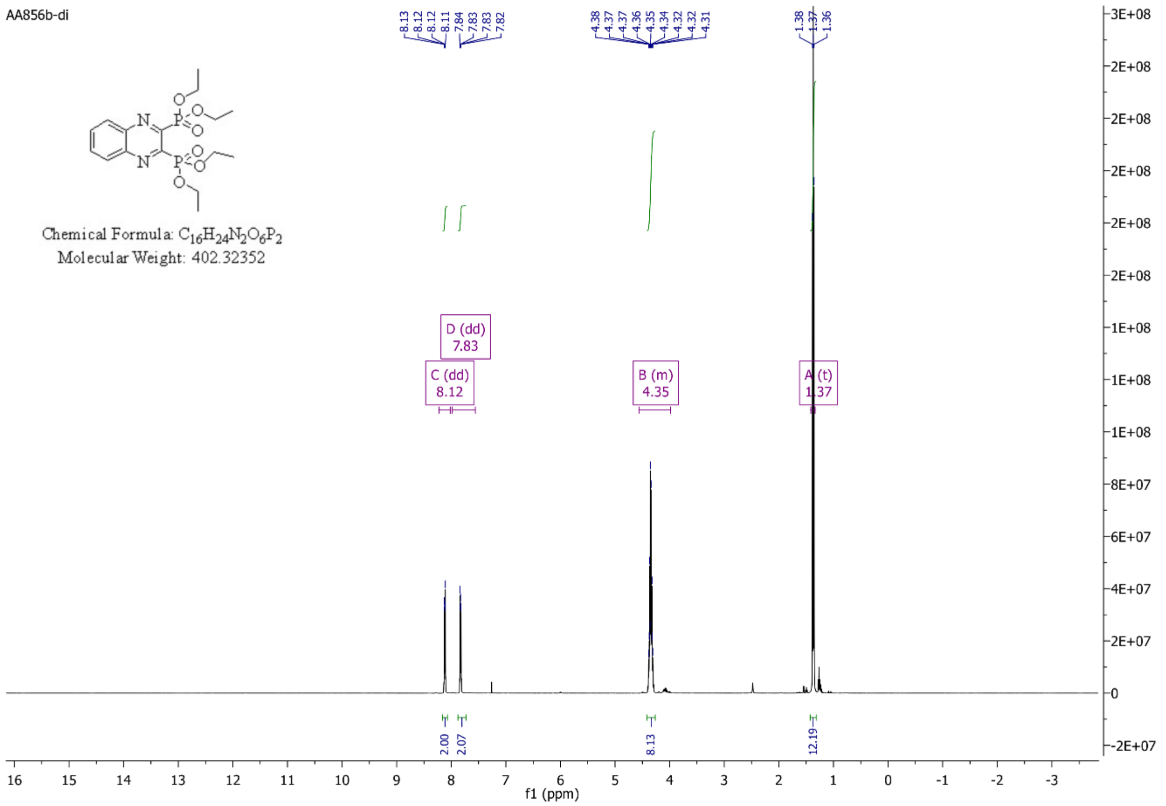
AA856b-di
Chemical Formula: C16H24N2O6P2
Molecular Weight: 402.32352
8.13 8.12 8.12 8.11 7.84 7.83 7.83 7.82
4.38 4.37 4.37 4.36 4.35 4.34 4.32 4.32 4.31
1.38 1.37 1.36
D (dd) 7.83
C (dd) 8.12
B (m) 4.35
A (t) 1.37
2.00 2.07 8.13 12.19
f1 (ppm)

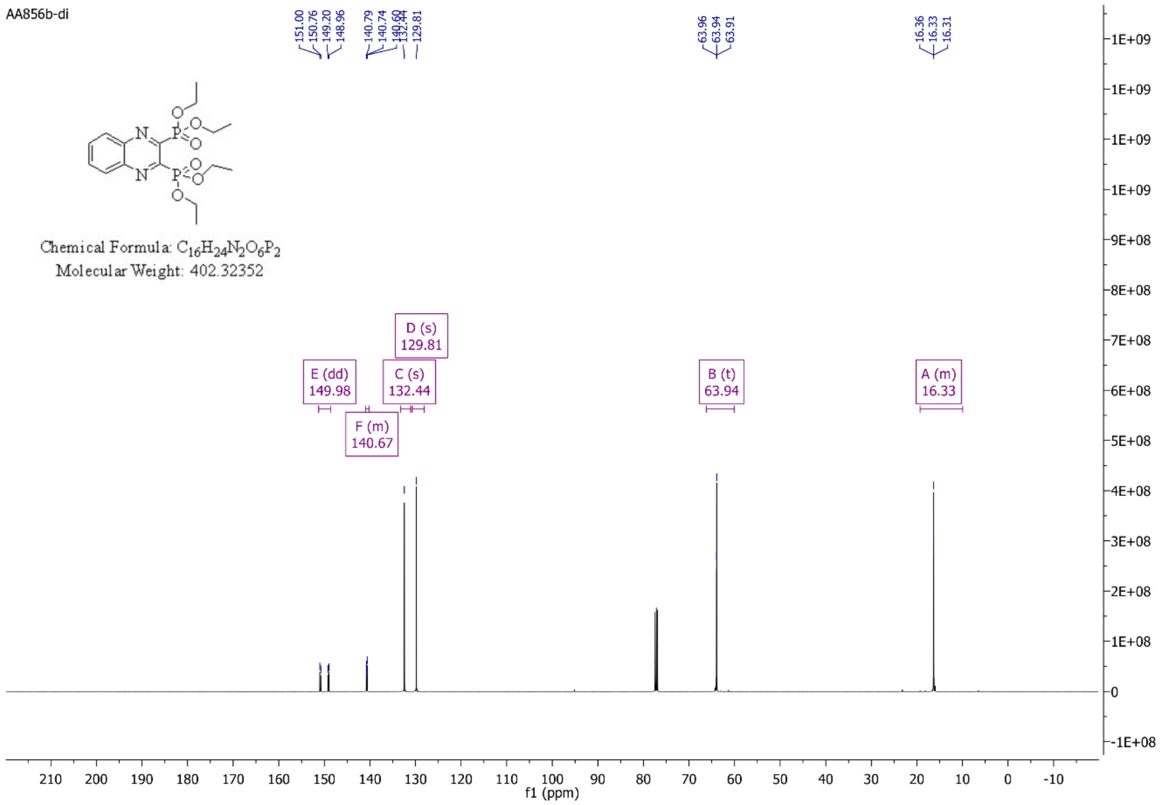
AA856b-di
Chemical Formula: C16H24N2O6P2
Molecular Weight: 402.32352
151.00 150.76 149.20 148.96 140.79 140.74 140.60 132.44 129.81
63.96 63.94 63.91
16.36 16.33 16.31
D (s) 129.81
E (dd) 149.98
C (s) 132.44
F (m) 140.67
B (t) 63.94
A (m) 16.33
f1 (ppm)

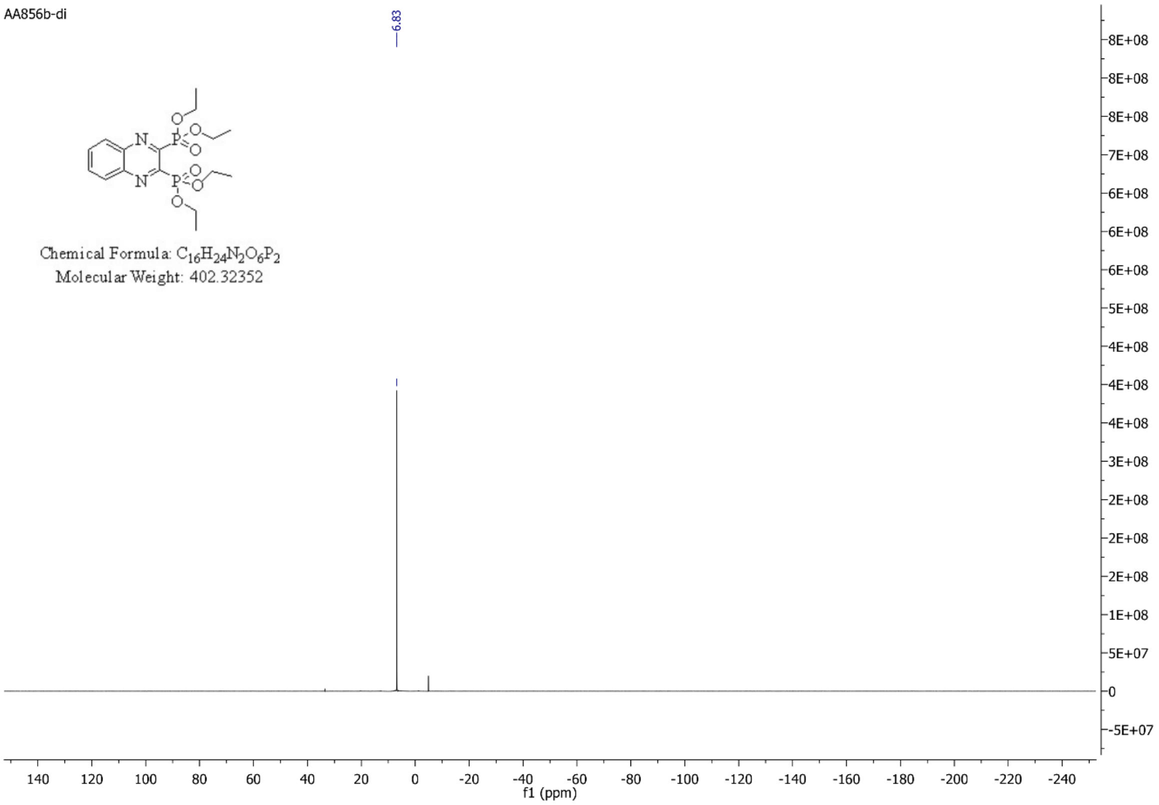
AA856b-di
6.83
Chemical Formula: C16H24N2O6P2
Molecular Weight: 402.32352
f1 (ppm)

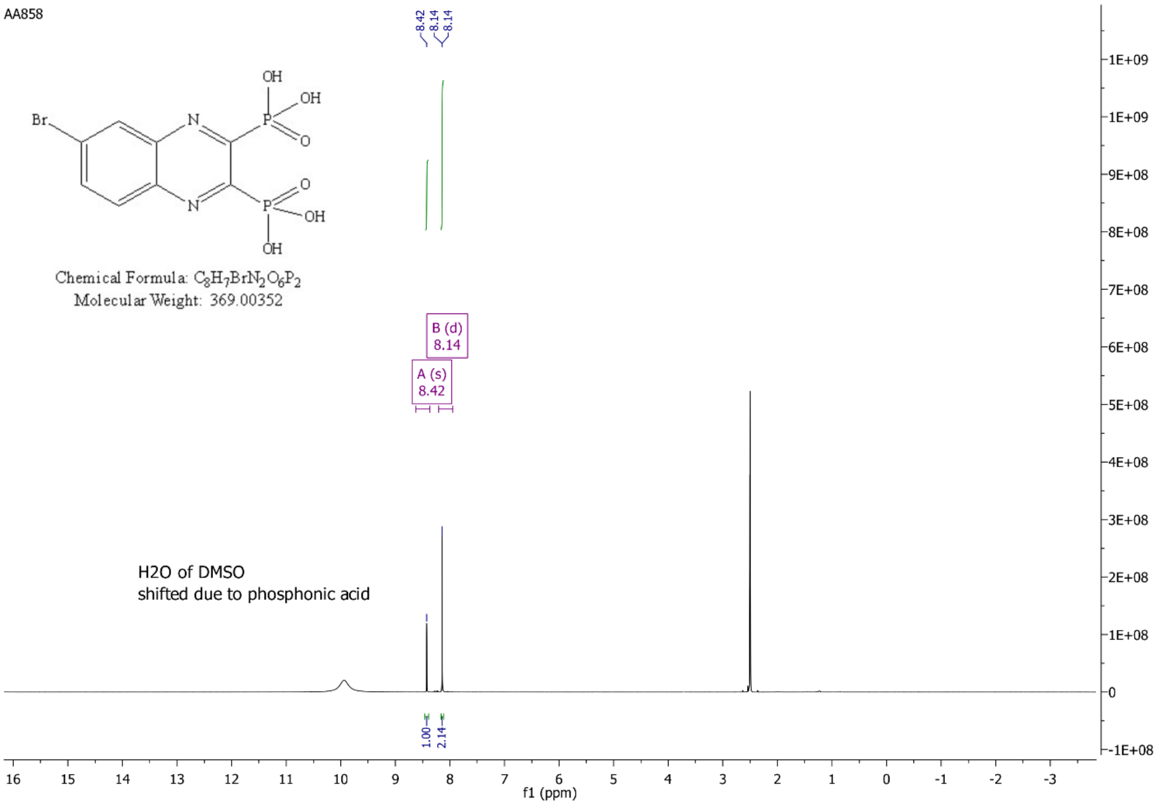
AA858
8.42
8.14
8.14
Chemical Formula: C8H7BrN2O6P2
Molecular Weight: 369.00352
B (d)
8.14
A (s)
8.42
H2O of DMSO
shifted due to phosphonic acid
1.00
2.14
f1 (ppm)

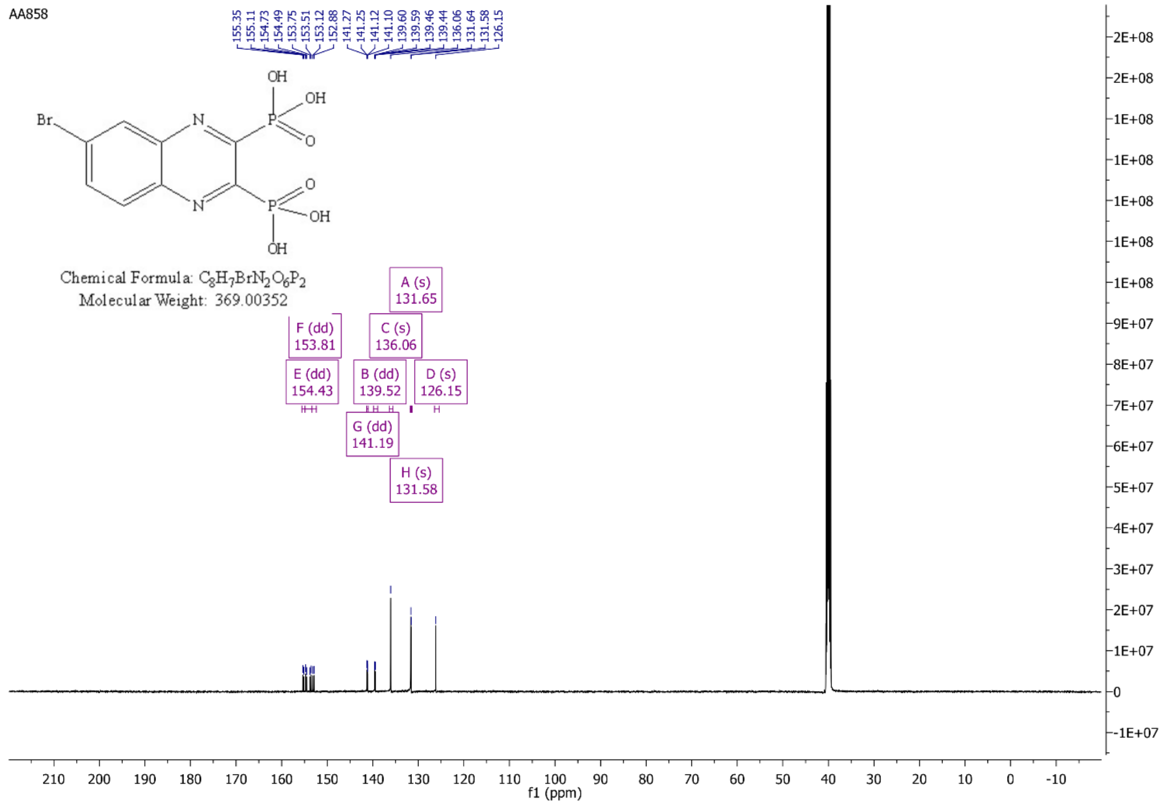
AA858
155.35
155.11
154.73
154.49
153.75
153.51
153.12
152.88
141.27
141.25
141.12
141.10
139.60
139.59
139.46
139.44
136.06
131.64
131.58
126.15
Br
N
N
P
P
OH
OH
O
O
OH
OH
Chemical Formula: C8H7BrN2O6P2
Molecular Weight: 369.00352
A (s)
131.65
F (dd)
153.81
C (s)
136.06
E (dd)
154.43
B (dd)
139.52
D (s)
126.15
G (dd)
141.19
H (s)
131.58
f1 (ppm)

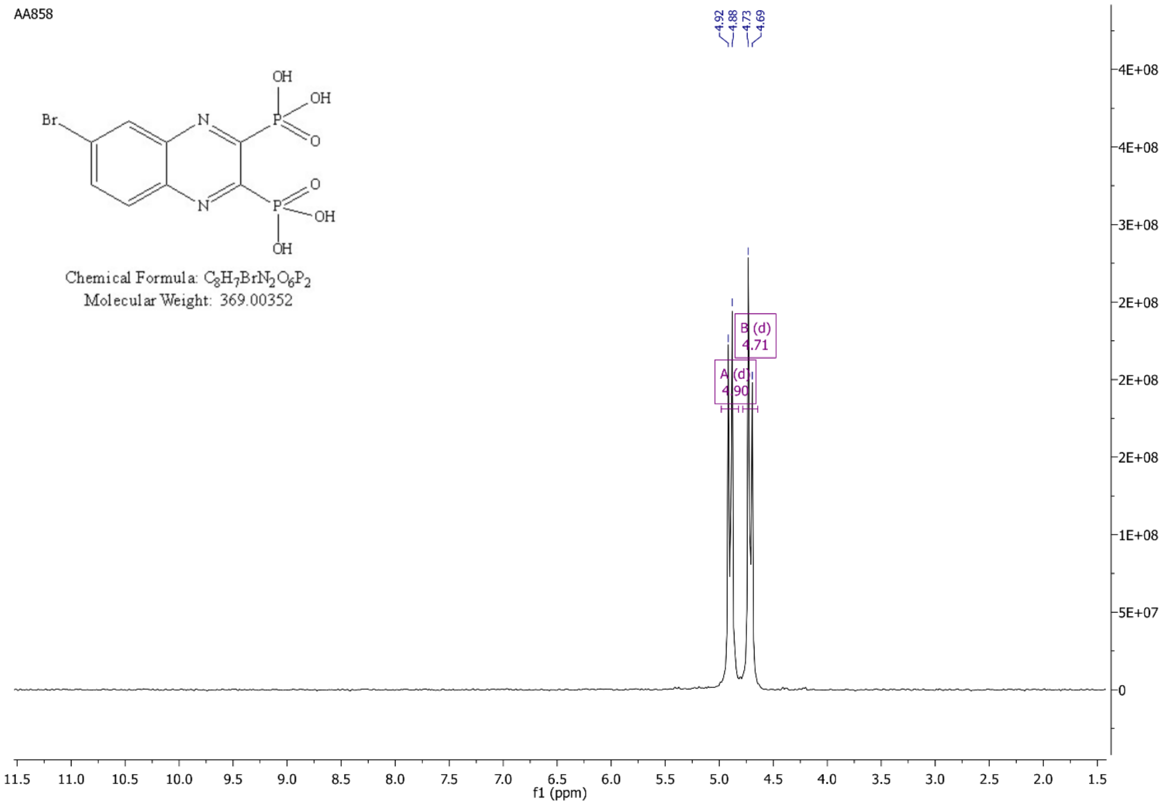
AA858
4.92
4.88
4.73
4.69
Br
N
N
P
P
OH
OH
O
O
OH
OH
Chemical Formula: C8H7BrN2O6P2
Molecular Weight: 369.00352
B (d)
4.71
A (d)
4.90
f1 (ppm)

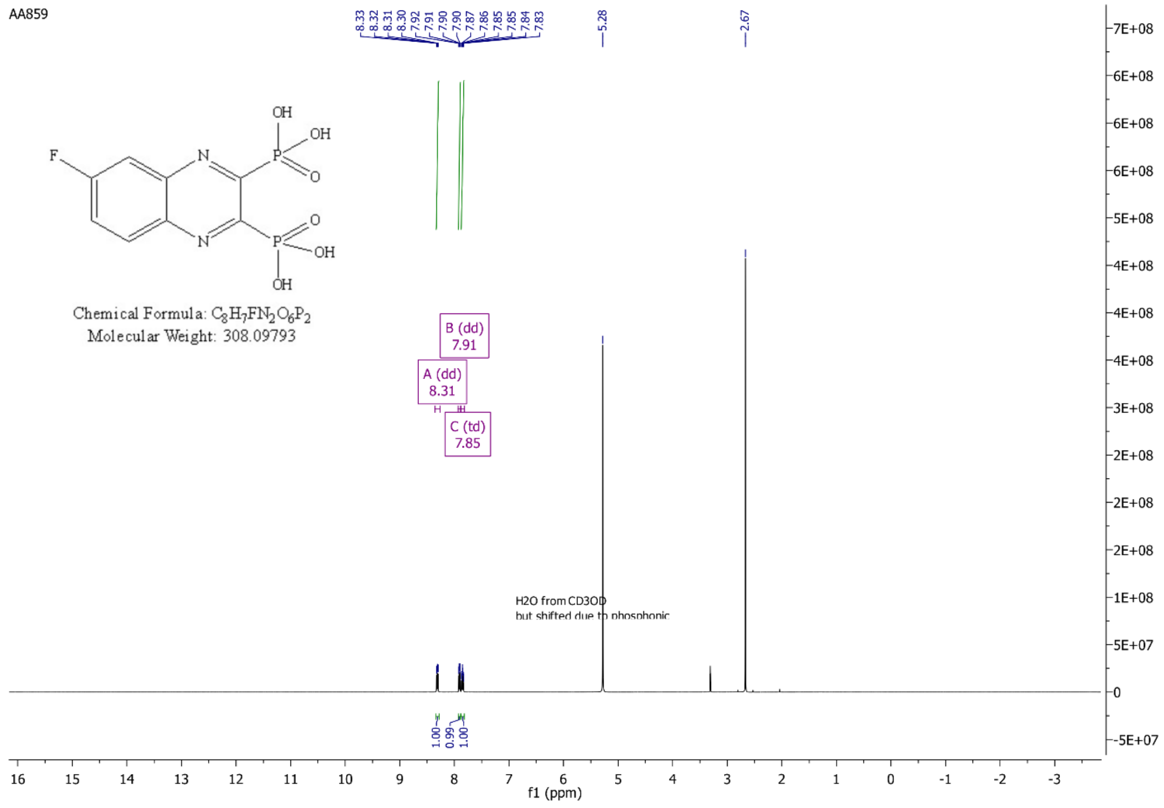
AA859
Chemical Formula: C8H7FN2O6P2
Molecular Weight: 308.09793
B (dd) 7.91
A (dd) 8.31
C (td) 7.85
H2O from CD3OD but shifted due to phosphonic
f1 (ppm)

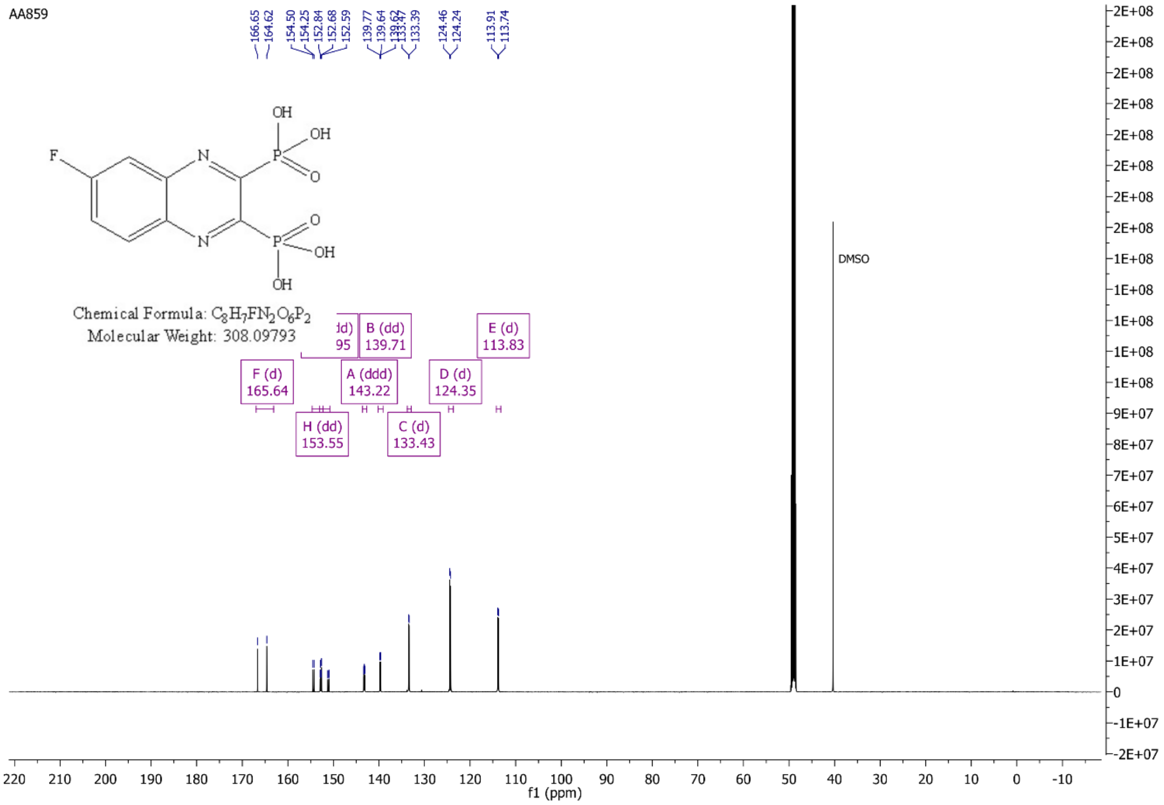
AA859
Chemical Formula: C8H7FN2O6P2
Molecular Weight: 308.09793
B (dd) 139.71
E (d) 113.83
F (d) 165.64
A (ddd) 143.22
D (d) 124.35
H (dd) 153.55
C (d) 133.43
DMSO
f1 (ppm)

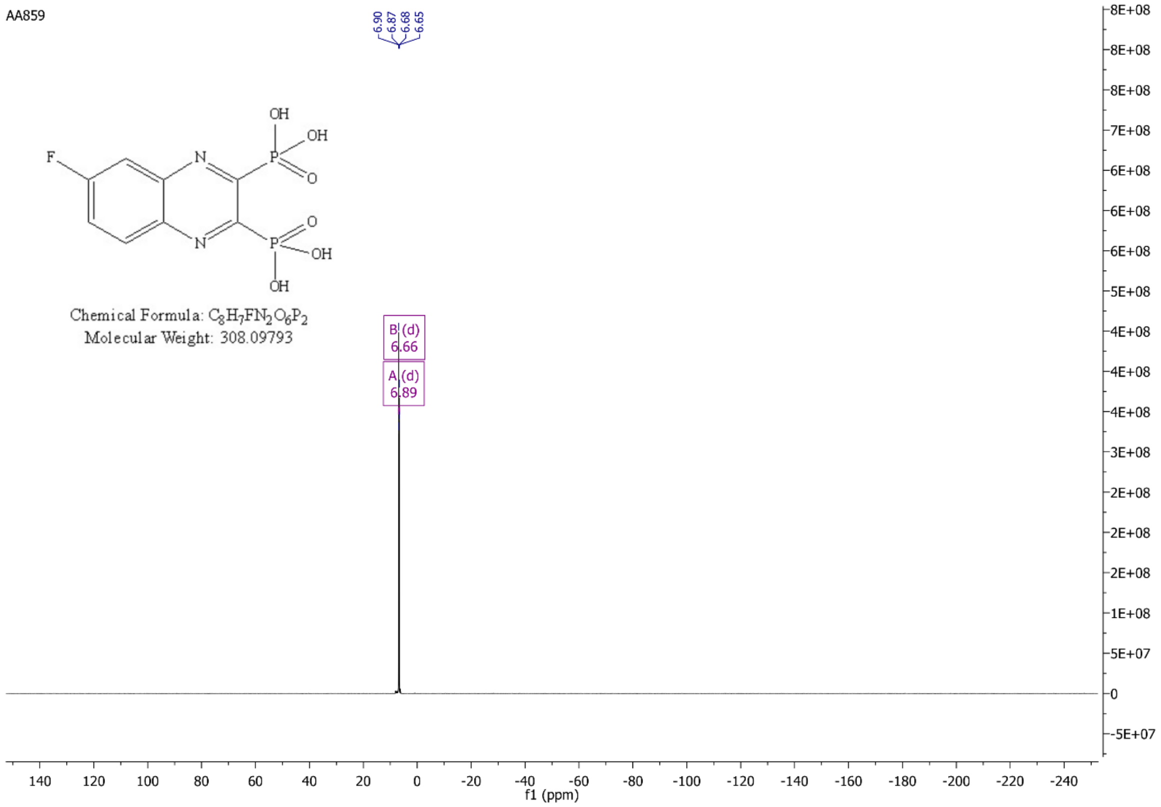

AA859
6.90
6.87
6.68
6.65
Chemical Formula: $C_8H_7FN_2O_6P_2$
Molecular Weight: 308.09793
B (d)
6.66
A (d)
6.89
f1 (ppm)

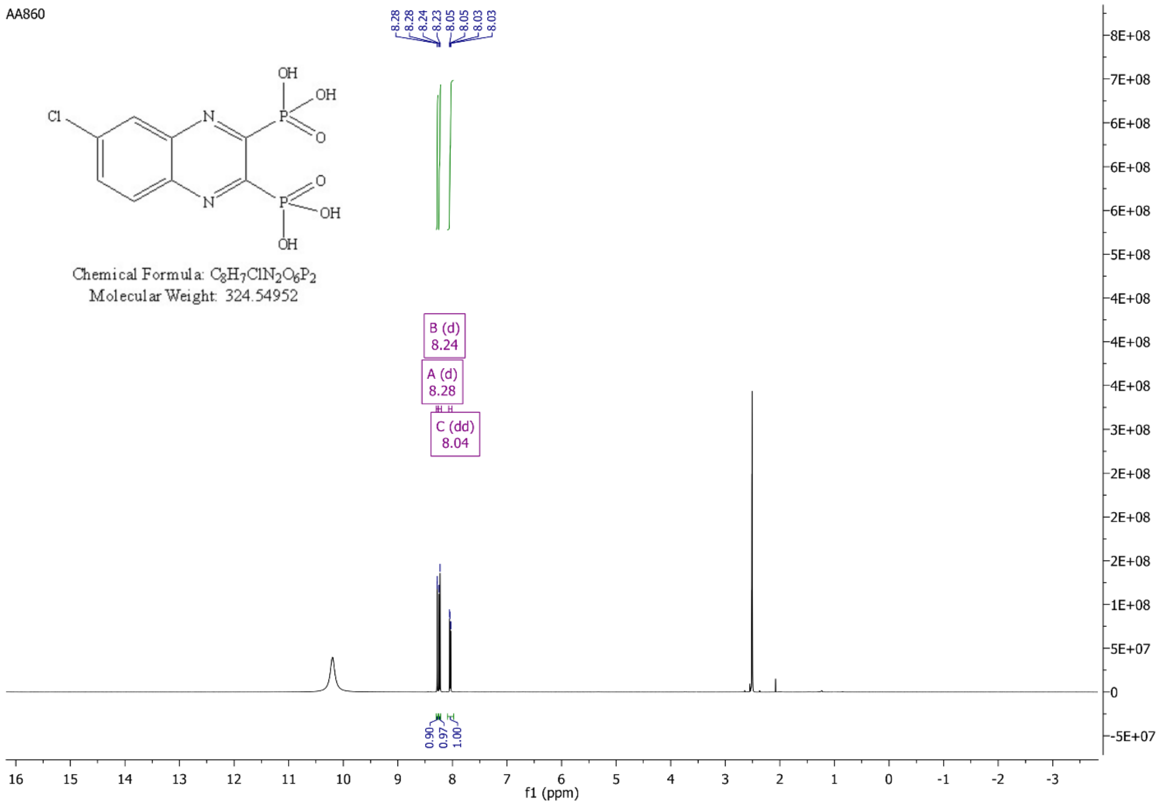

AA860
8.28
8.28
8.24
8.23
8.05
8.05
8.03
8.03
Chemical Formula: $C_8H_7ClN_2O_6P_2$
Molecular Weight: 324.54952
B (d)
8.24
A (d)
8.28
C (dd)
8.04
0.90
0.97
1.00
f1 (ppm)

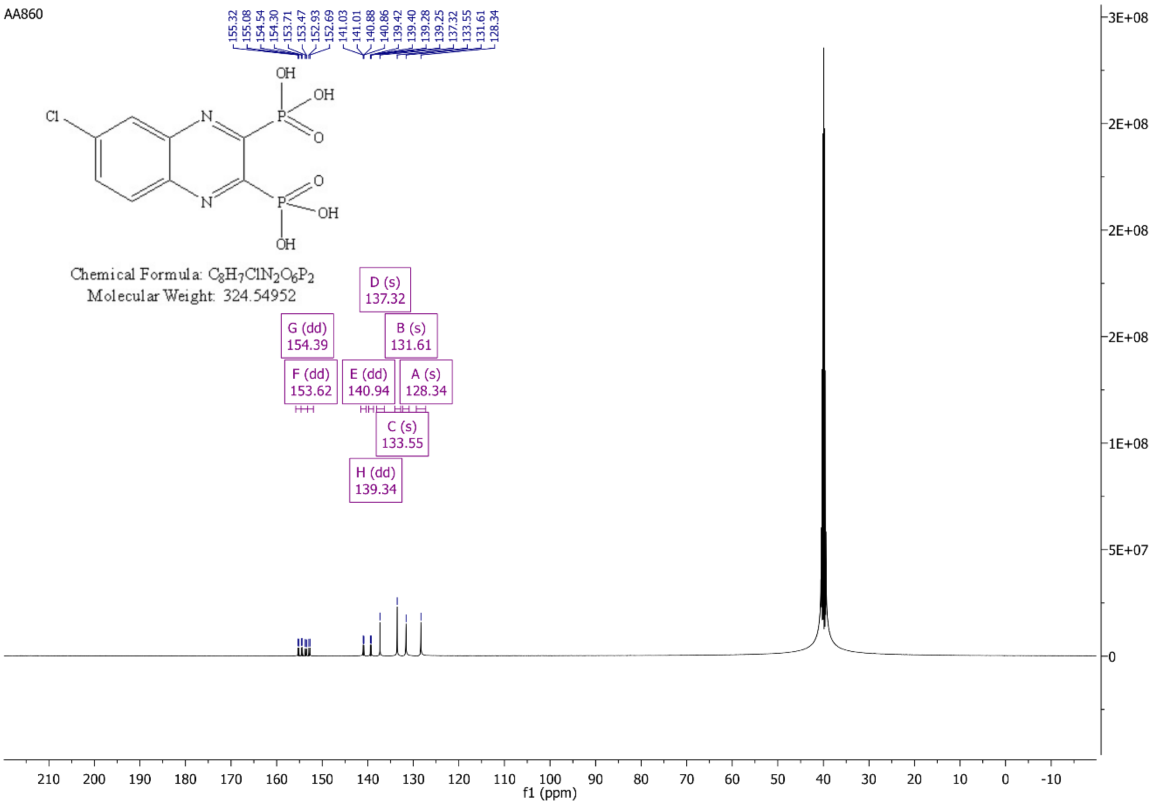
AA860
155.32
155.08
154.54
154.30
153.71
153.47
152.93
152.69
141.03
141.01
140.88
140.86
139.42
139.40
139.28
139.25
137.32
133.55
131.61
128.34
Chemical Formula: $C_8H_7ClN_2O_6P_2$
Molecular Weight: 324.54952
D (s) 137.32
G (dd) 154.39
B (s) 131.61
F (dd) 153.62
E (dd) 140.94
A (s) 128.34
C (s) 133.55
H (dd) 139.34
f1 (ppm)

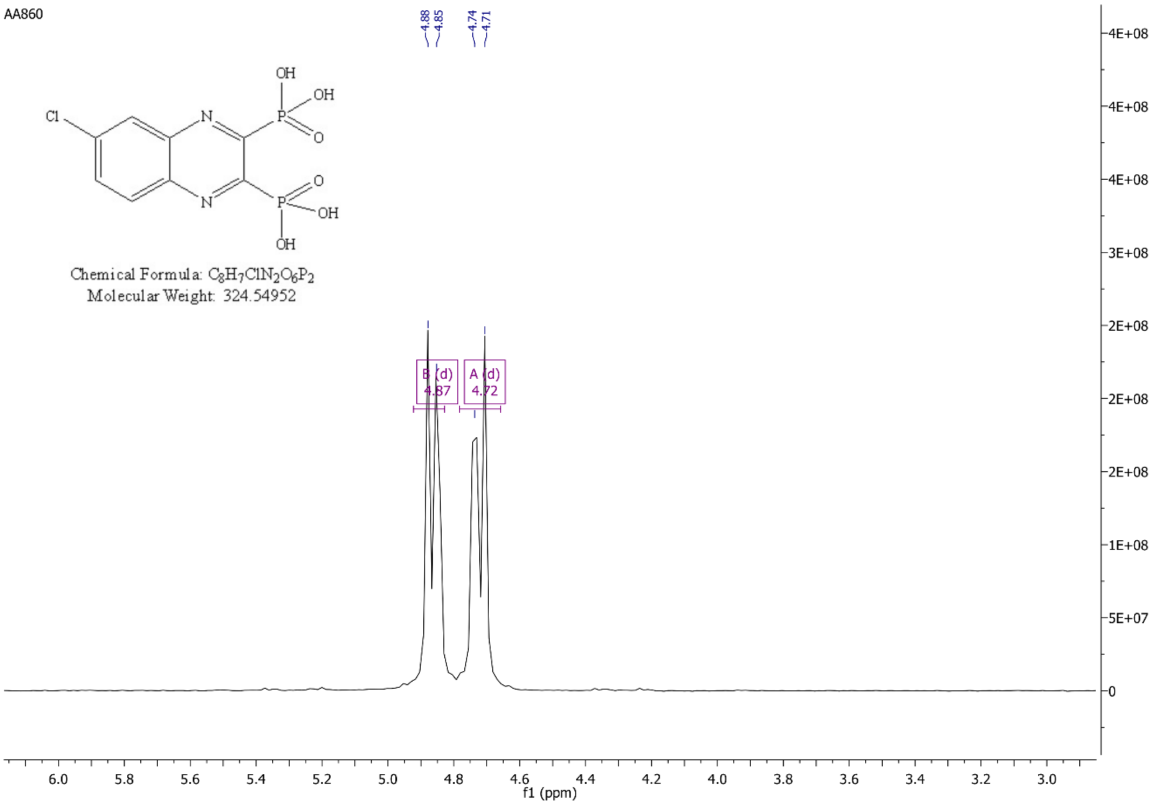
AA860
4.88
4.85
4.74
4.71
Chemical Formula: $C_8H_7ClN_2O_6P_2$
Molecular Weight: 324.54952
B (d) 4.87
A (d) 4.72
f1 (ppm)

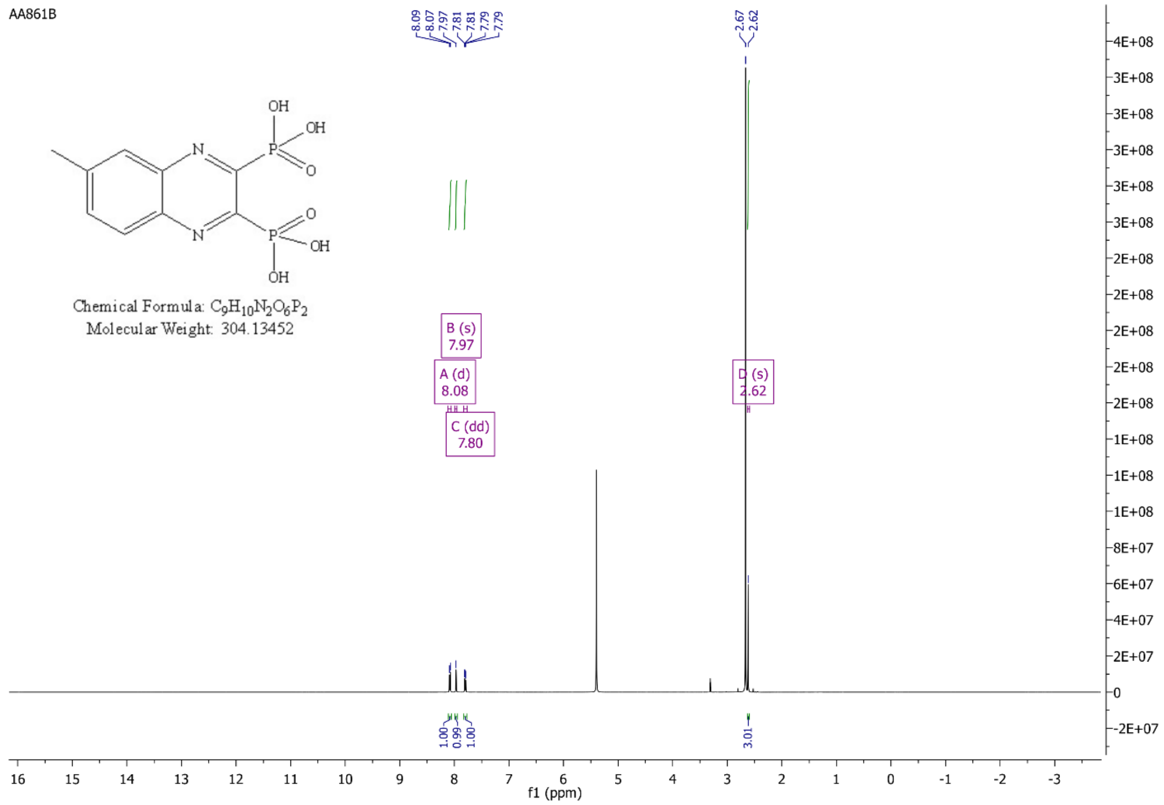
AA861B
8.09 8.07 7.97 7.81 7.81 7.79 7.79
2.67 2.62
Chemical Formula: C9H10N2O6P2
Molecular Weight: 304.13452
B (s) 7.97
A (d) 8.08
C (dd) 7.80
D (s) 2.62
1.00 0.99 1.00
3.01
f1 (ppm)

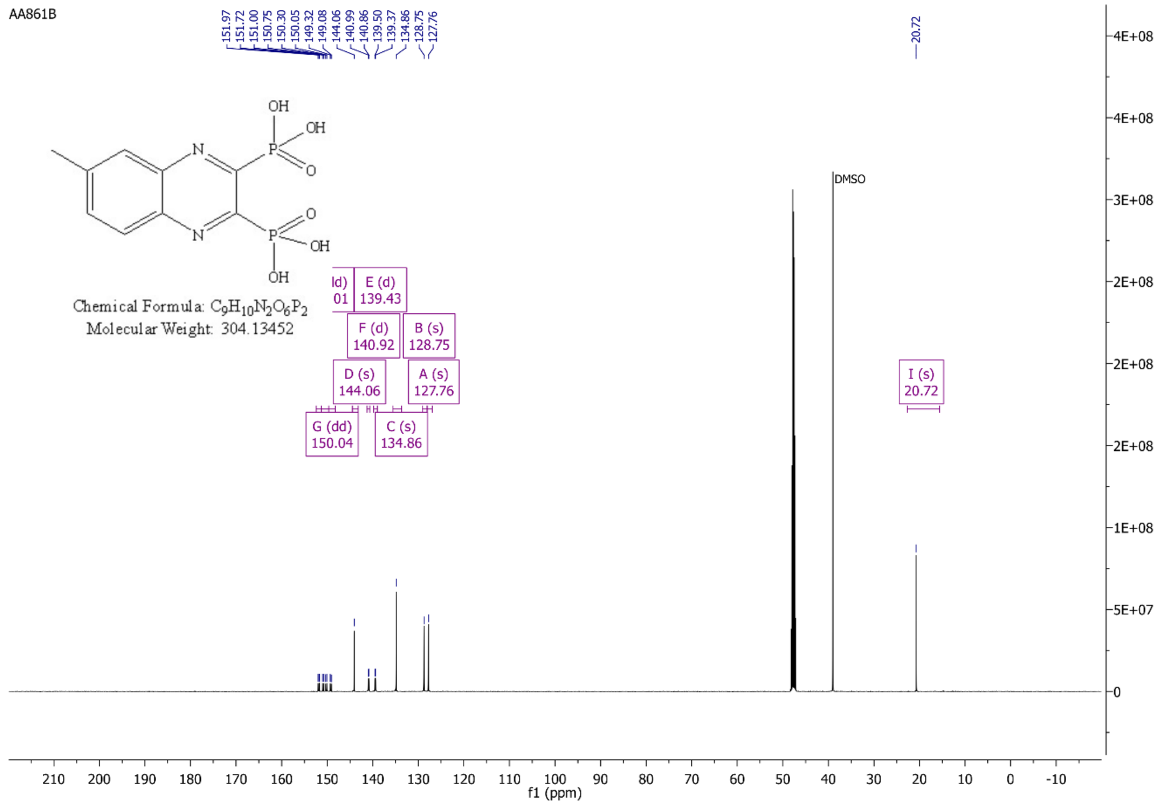
AA861B
151.97 151.72 151.00 150.75 150.30 150.05 149.32 149.08 144.06 140.99 140.86 139.50 139.37 134.86 128.75 127.76
20.72
Chemical Formula: C9H10N2O6P2
Molecular Weight: 304.13452
DMSO
E (d) 139.43
F (d) 140.92
B (s) 128.75
D (s) 144.06
A (s) 127.76
G (dd) 150.04
C (s) 134.86
I (s) 20.72
f1 (ppm)

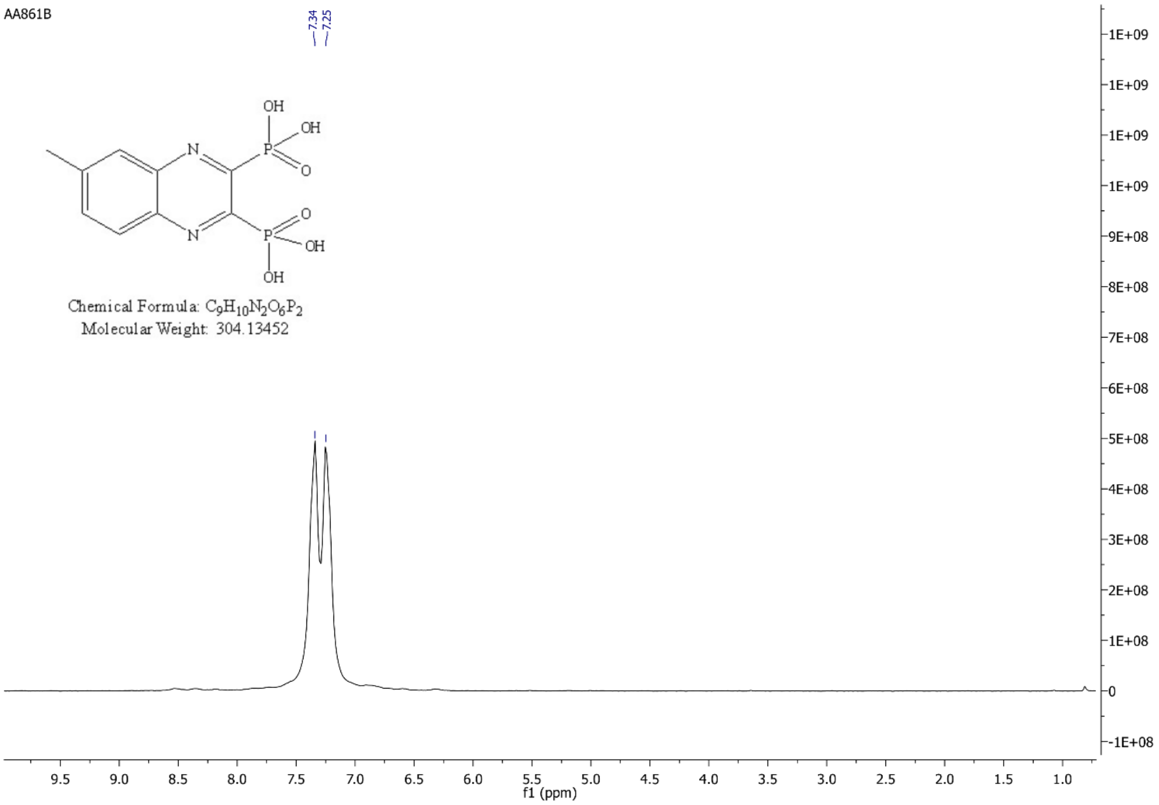
AA861B
7.34
7.25
OH
OH
O
O
OH
OH
N
N
P
P
Chemical Formula: C9H10N2O6P2
Molecular Weight: 304.13452
f1 (ppm)

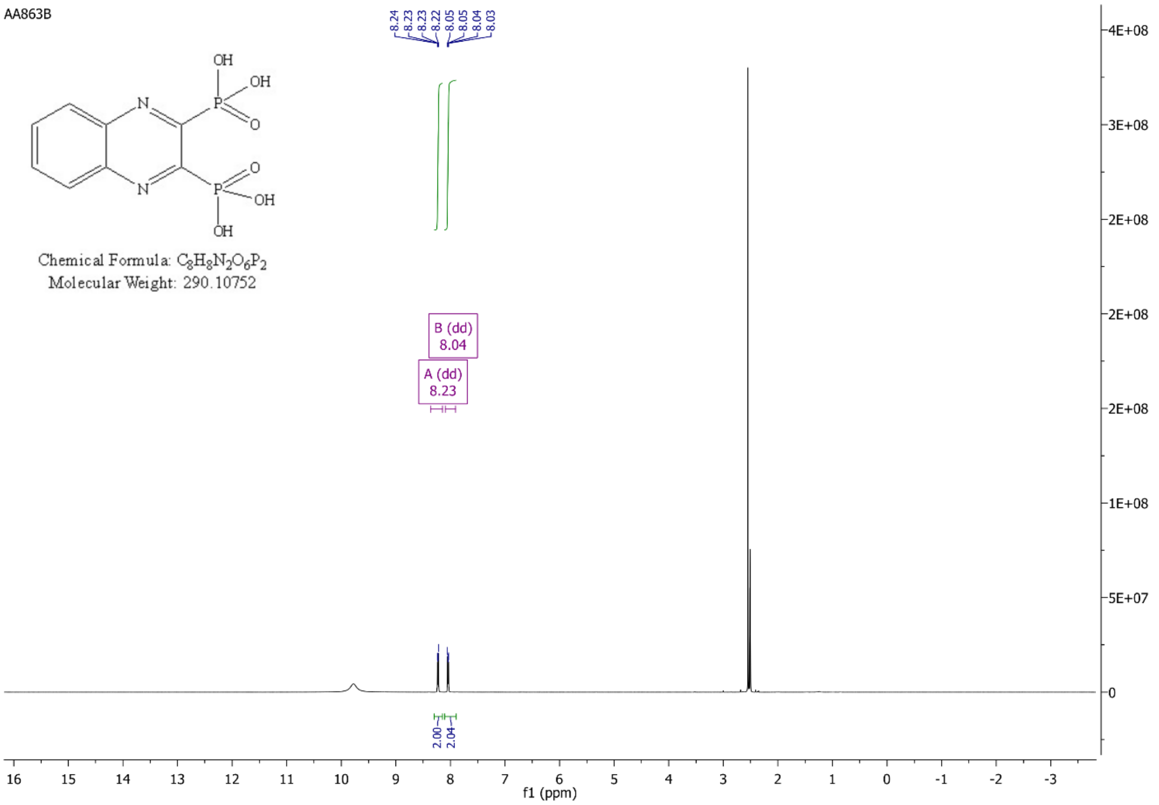
AA863B
8.24
8.23
8.23
8.22
8.05
8.05
8.04
8.03
Chemical Formula: C8H8N2O6P2
Molecular Weight: 290.10752
B (dd)
8.04
A (dd)
8.23
2.00
2.04
f1 (ppm)

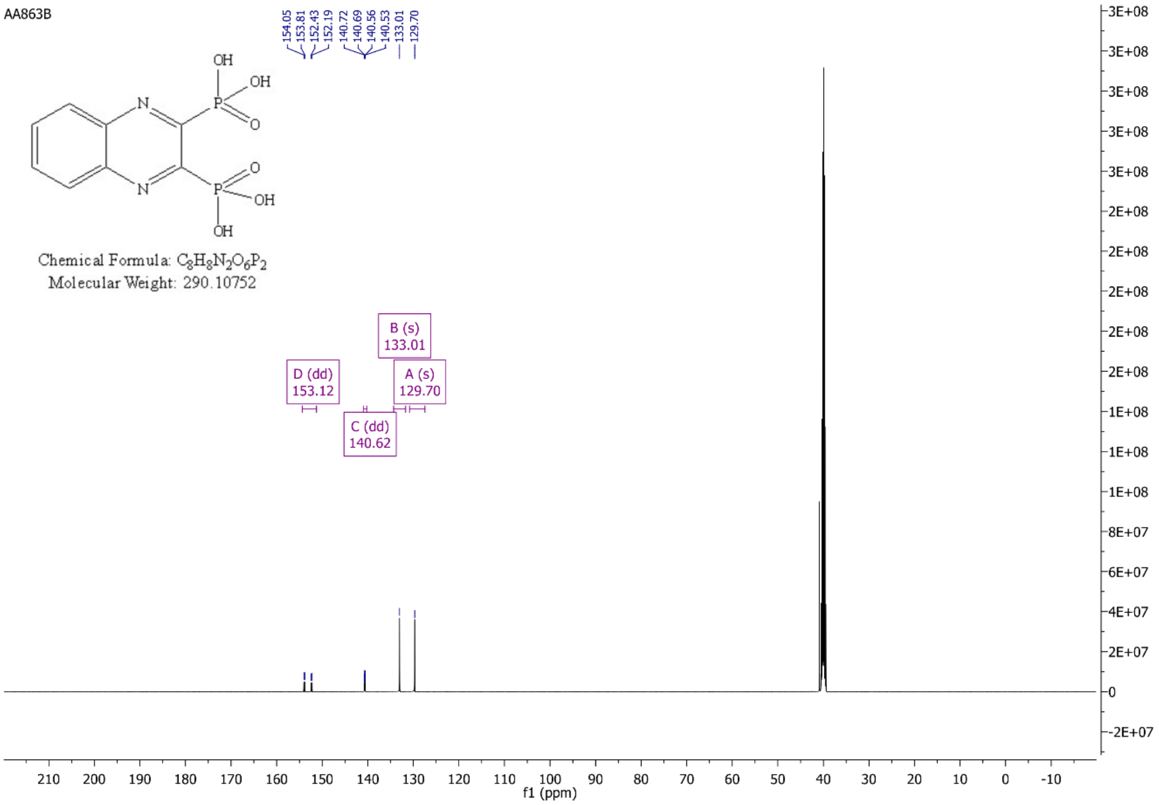
AA863B
154.05
153.81
152.43
152.19
140.72
140.69
140.56
140.53
133.01
129.70
Chemical Formula: $C_8H_8N_2O_6P_2$
Molecular Weight: 290.10752
B (s)
133.01
D (dd)
153.12
A (s)
129.70
C (dd)
140.62
f1 (ppm)

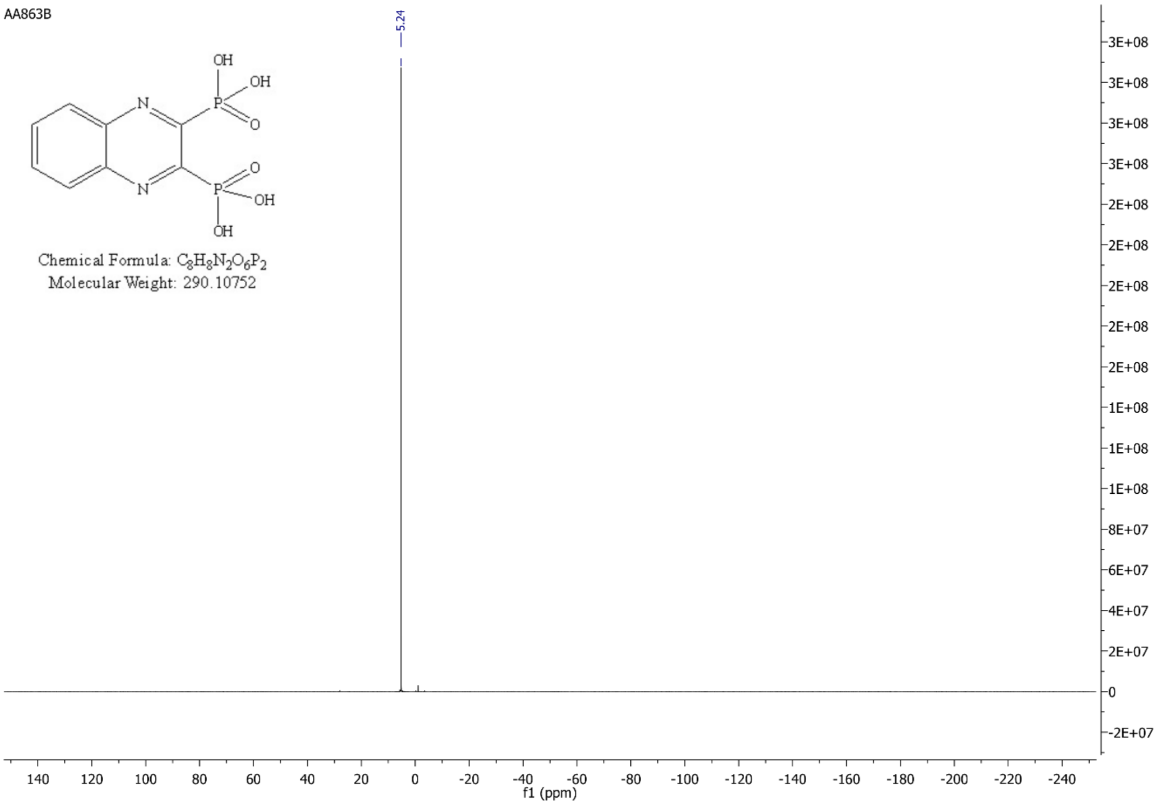
AA863B
5.24
Chemical Formula: $C_8H_8N_2O_6P_2$
Molecular Weight: 290.10752
f1 (ppm)

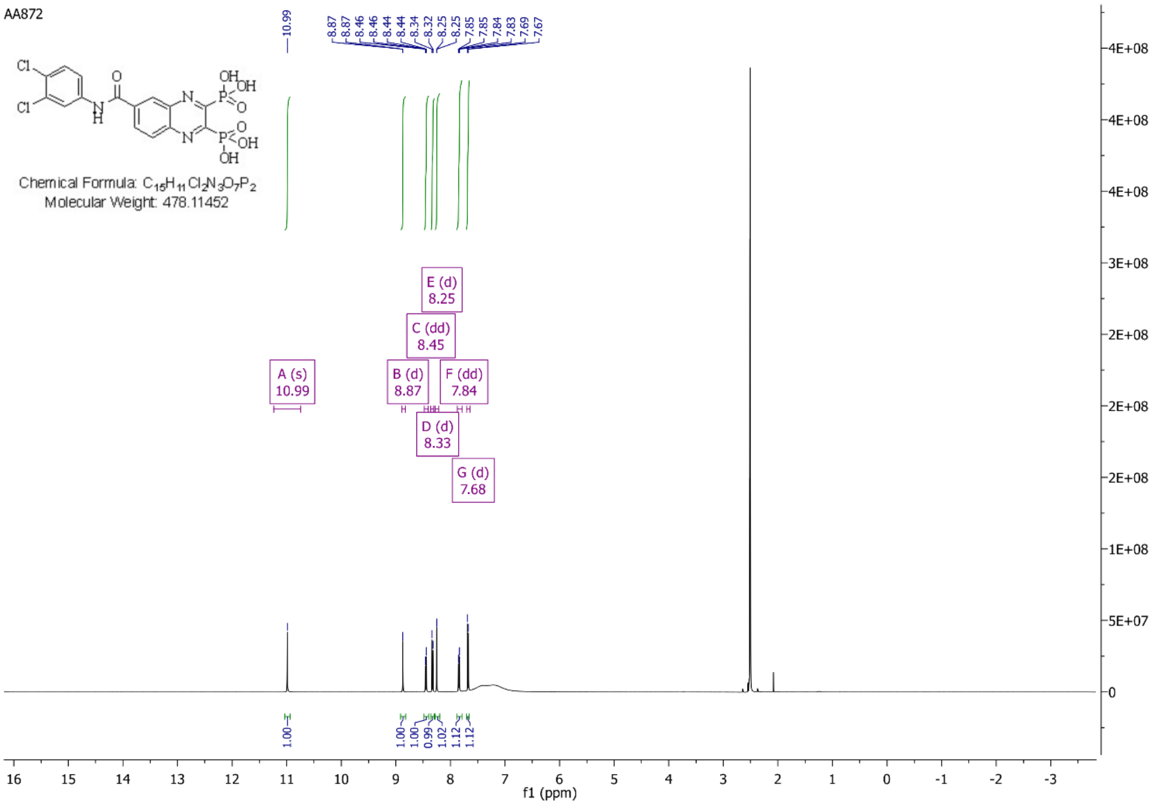
AA872
Chemical Formula: C15H11Cl2N3O7P2
Molecular Weight: 478.11452
A (s) 10.99
B (d) 8.87
C (dd) 8.45
D (d) 8.33
E (d) 8.25
F (dd) 7.84
G (d) 7.68
f1 (ppm)

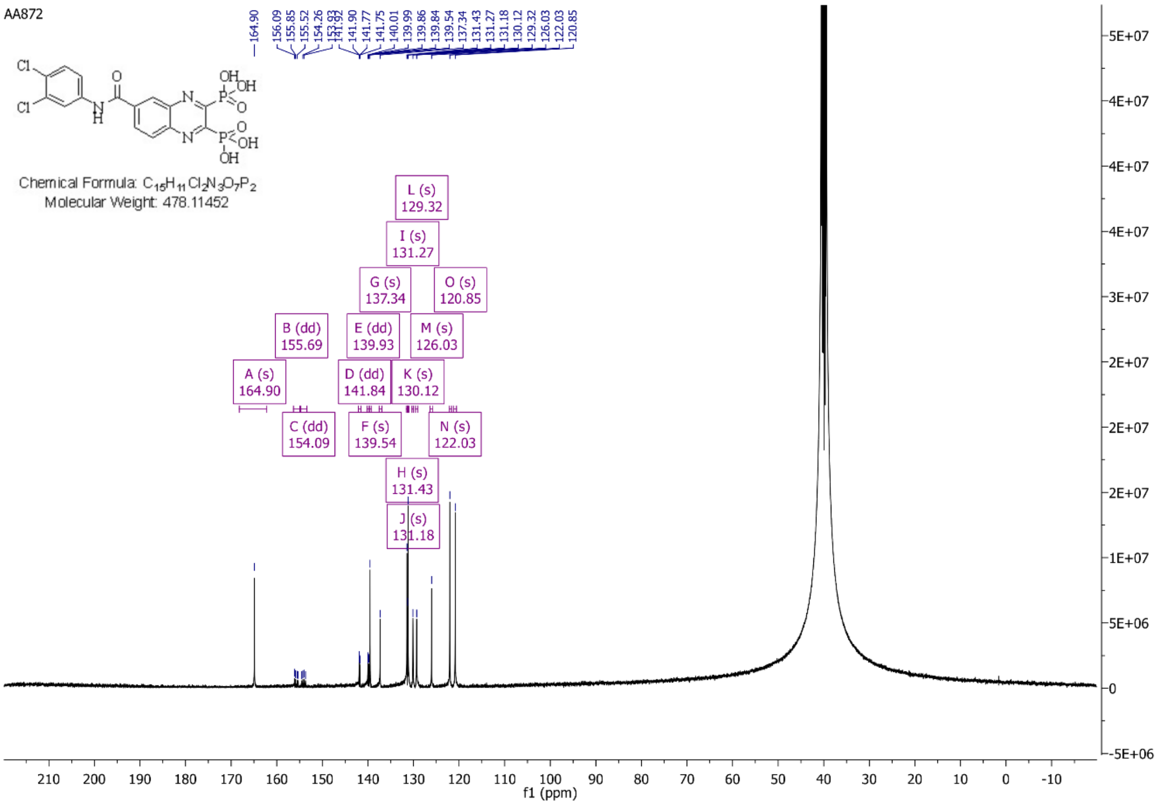
AA872
Chemical Formula: C15H11Cl2N3O7P2
Molecular Weight: 478.11452
A (s) 164.90
B (dd) 155.69
C (dd) 154.09
D (dd) 141.84
E (dd) 139.93
F (s) 139.54
G (s) 137.34
H (s) 131.43
I (s) 131.27
J (s) 131.18
K (s) 130.12
L (s) 129.32
M (s) 126.03
N (s) 122.03
O (s) 120.85
f1 (ppm)

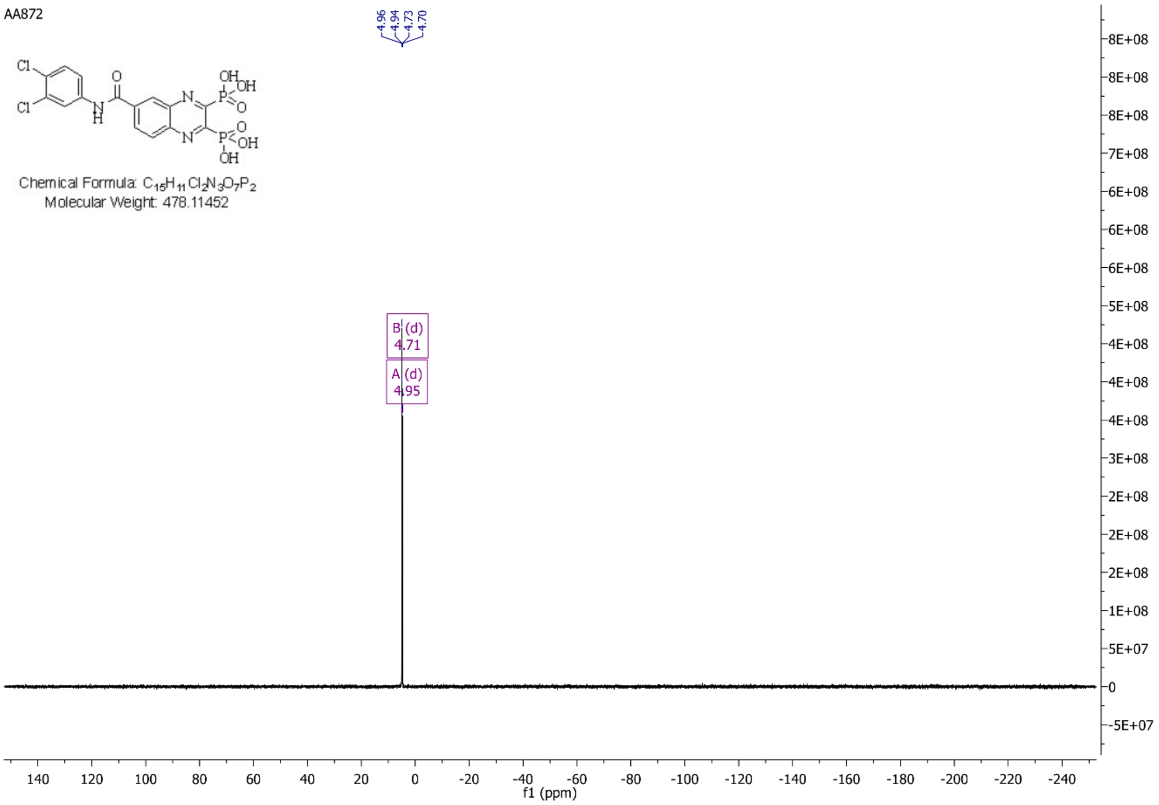
AA872
Chemical Formula: C15H11Cl2N3O7P2
Molecular Weight: 478.11452
4.96
4.94
4.73
4.70
B (d)
4.71
A (d)
4.95
f1 (ppm)

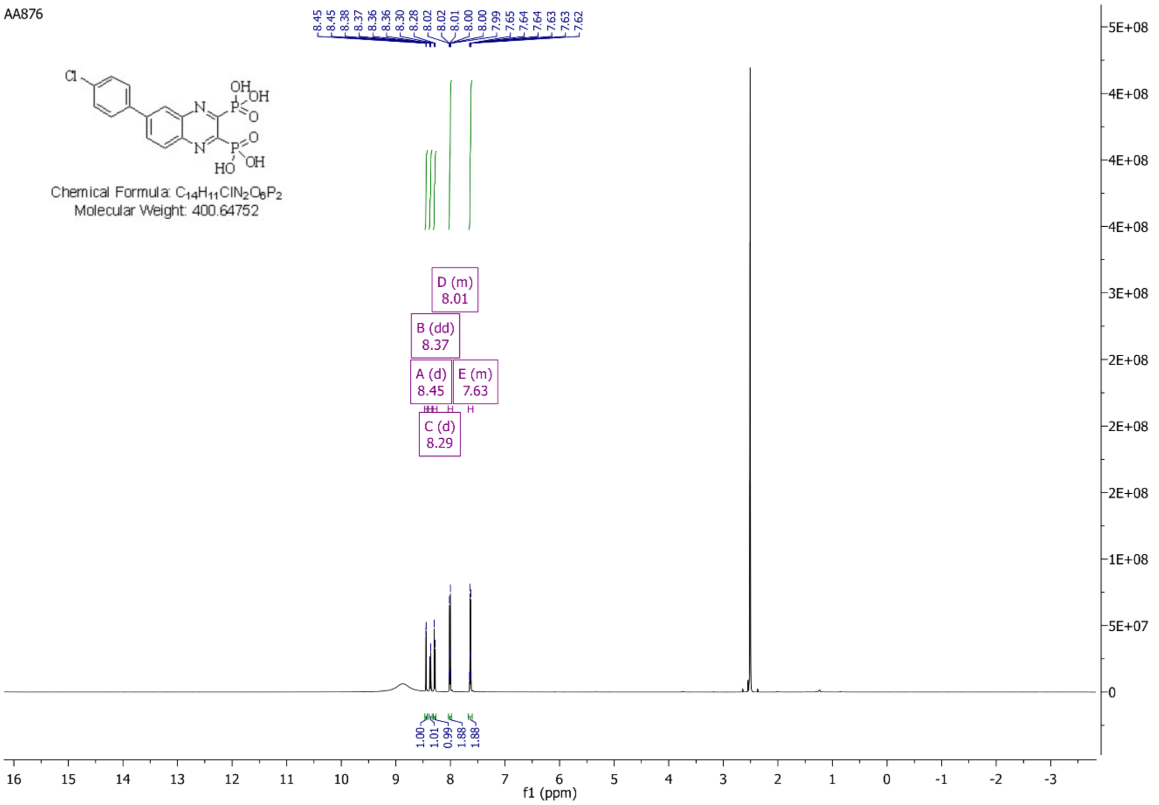
AA876
Chemical Formula: C14H11ClN2O6P2
Molecular Weight: 400.64752
8.45
8.45
8.38
8.37
8.36
8.36
8.30
8.28
8.02
8.02
8.01
8.00
8.00
7.99
7.65
7.64
7.64
7.63
7.63
7.62
D (m)
8.01
B (dd)
8.37
A (d)
8.45
E (m)
7.63
C (d)
8.29
1.00
1.01
0.99
1.88
1.88
f1 (ppm)

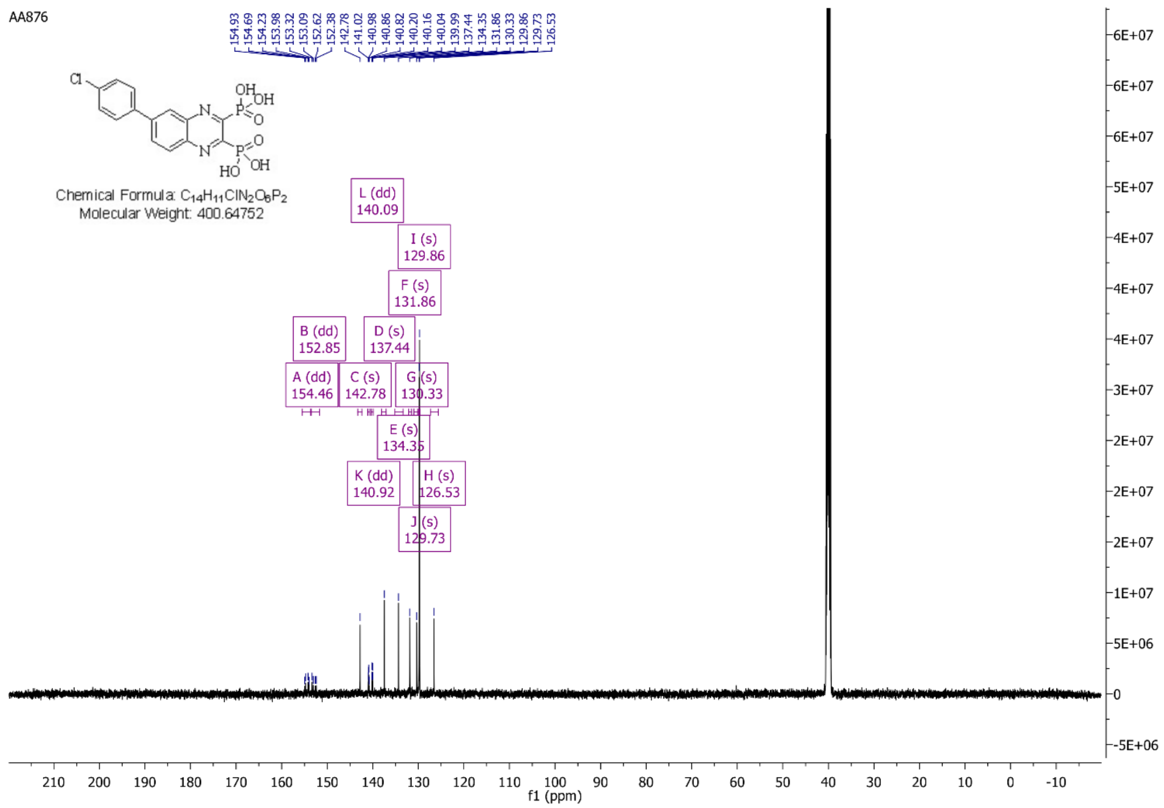
AA876
154.93
154.69
154.23
153.98
153.32
153.09
152.62
152.38
142.78
141.02
140.98
140.86
140.82
140.20
140.16
140.04
139.99
137.44
134.35
131.86
130.33
129.86
129.73
126.53
Chemical Formula: C14H11ClN2O6P2
Molecular Weight: 400.64752
L (dd) 140.09
I (s) 129.86
F (s) 131.86
B (dd) 152.85
D (s) 137.44
A (dd) 154.46
C (s) 142.78
G (s) 130.33
E (s) 134.35
K (dd) 140.92
H (s) 126.53
J (s) 129.73
f1 (ppm)

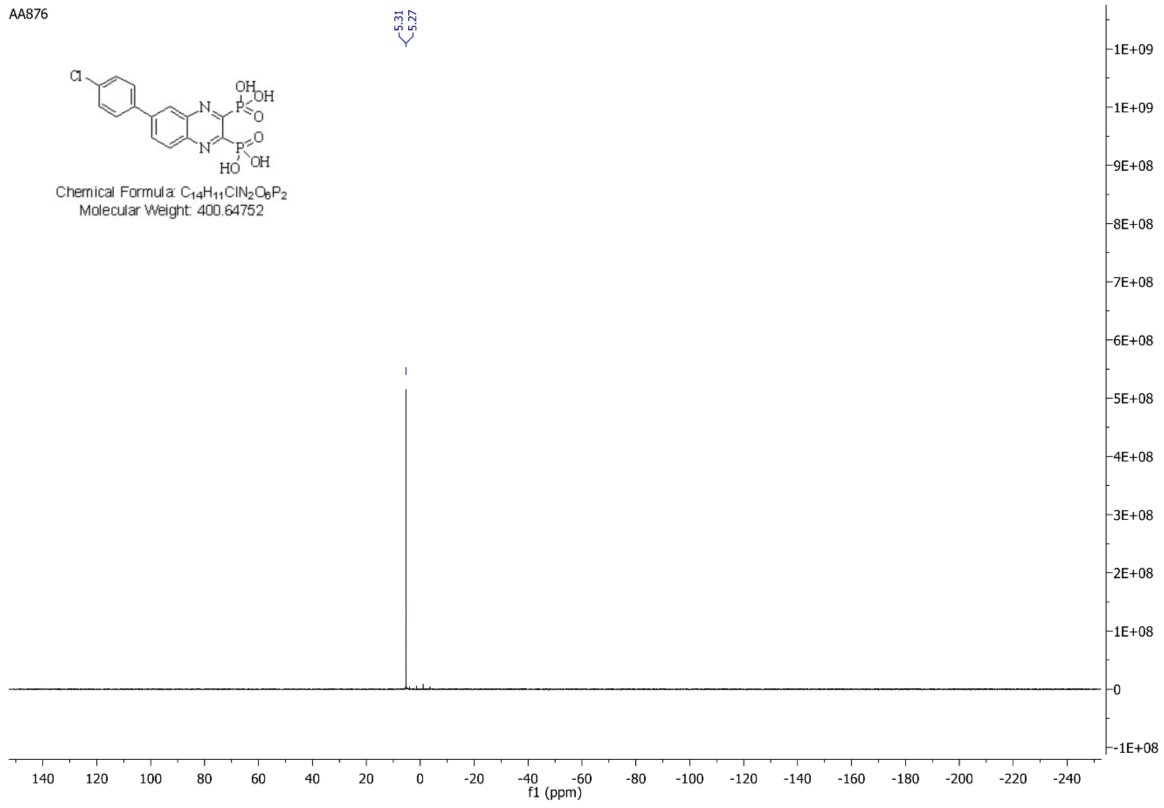
AA876
5.31
5.27
Chemical Formula: C14H11ClN2O6P2
Molecular Weight: 400.64752
f1 (ppm)

Hydoxamate compounds

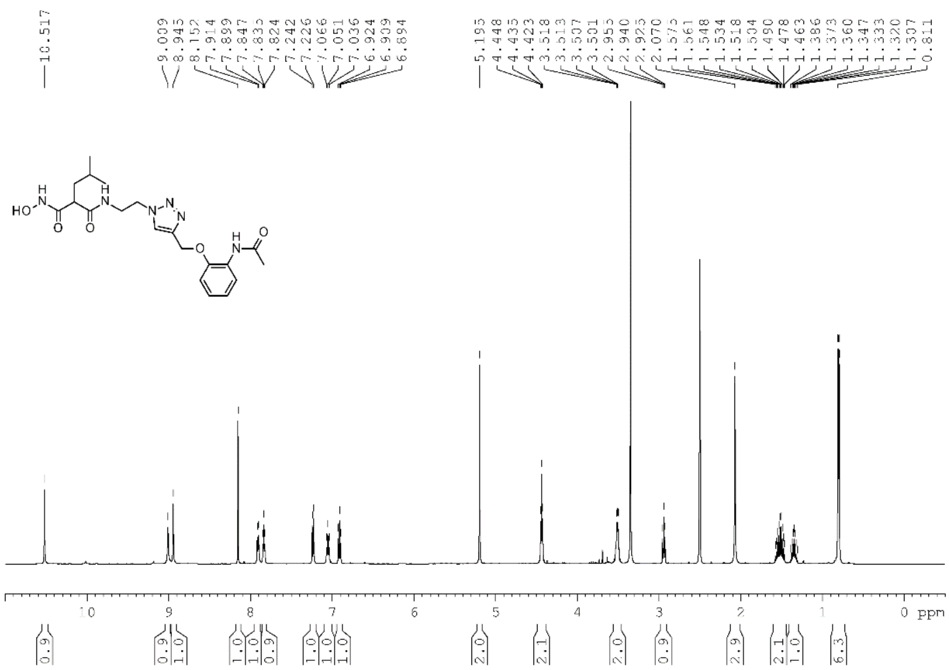

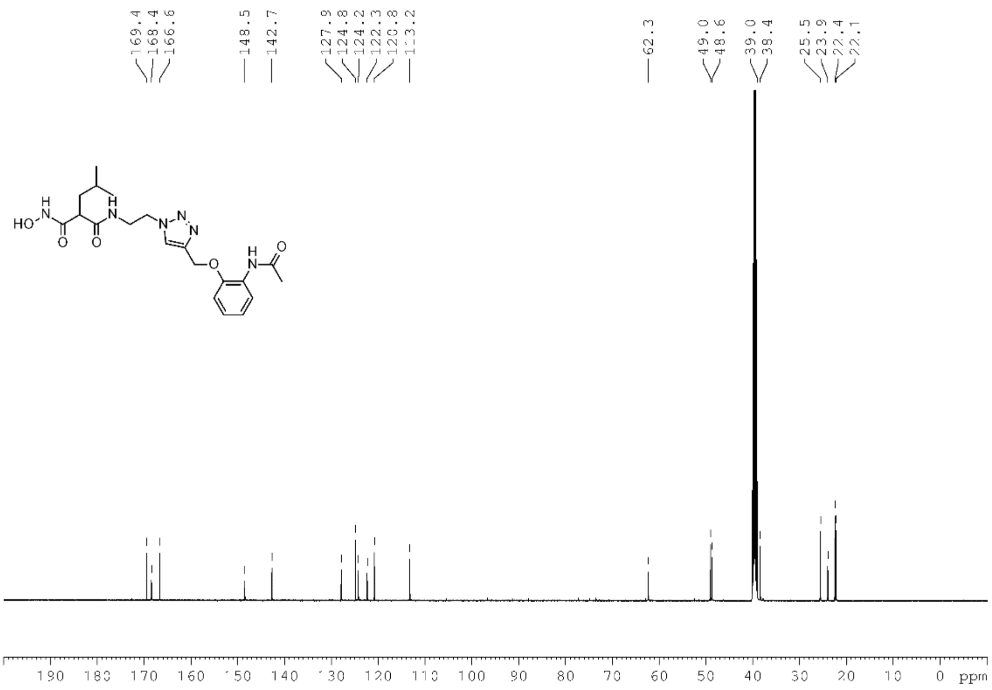

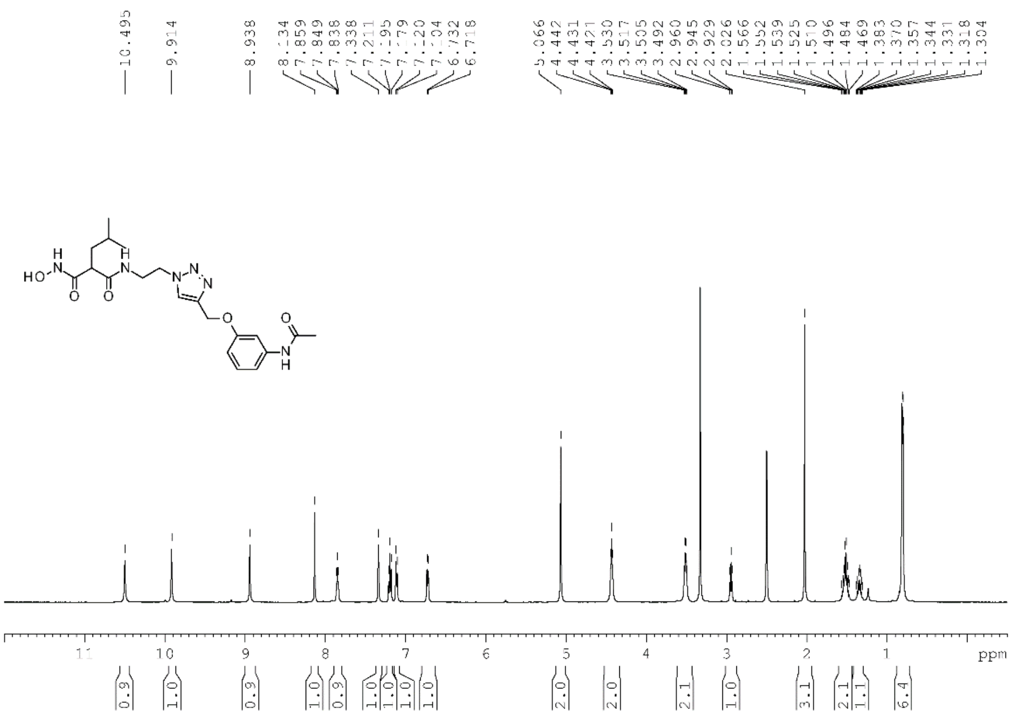

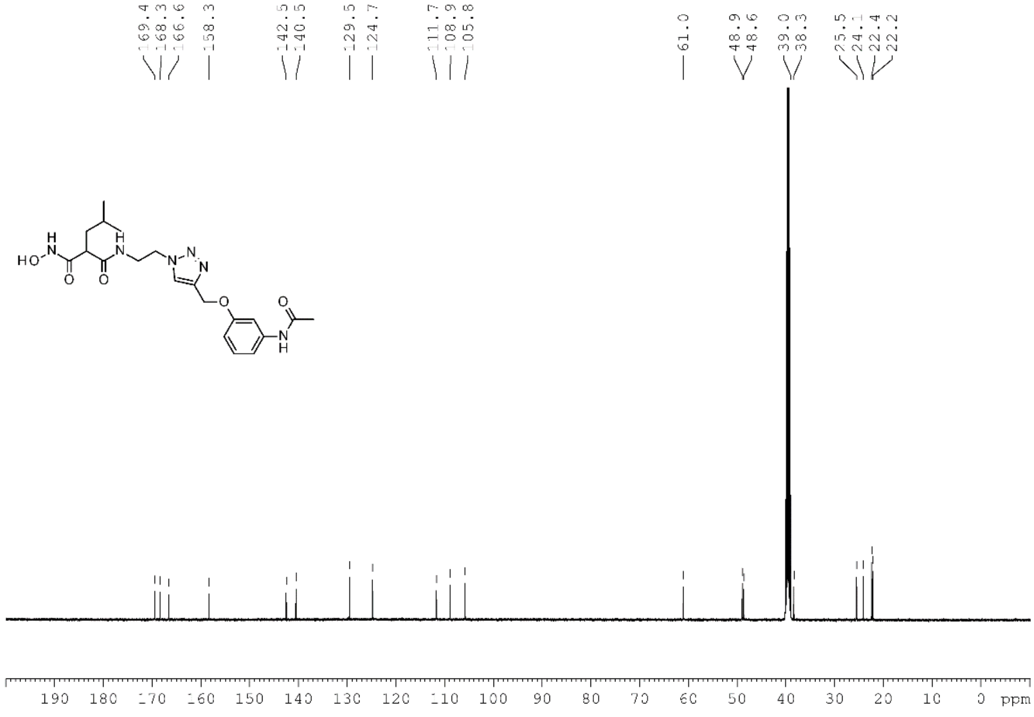

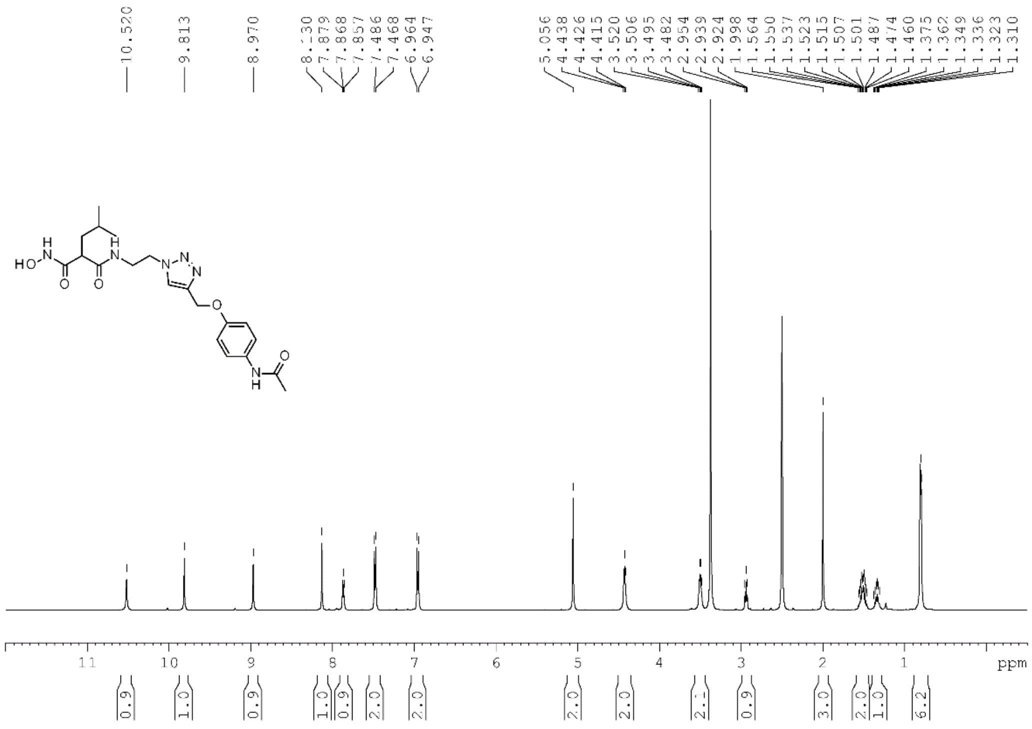

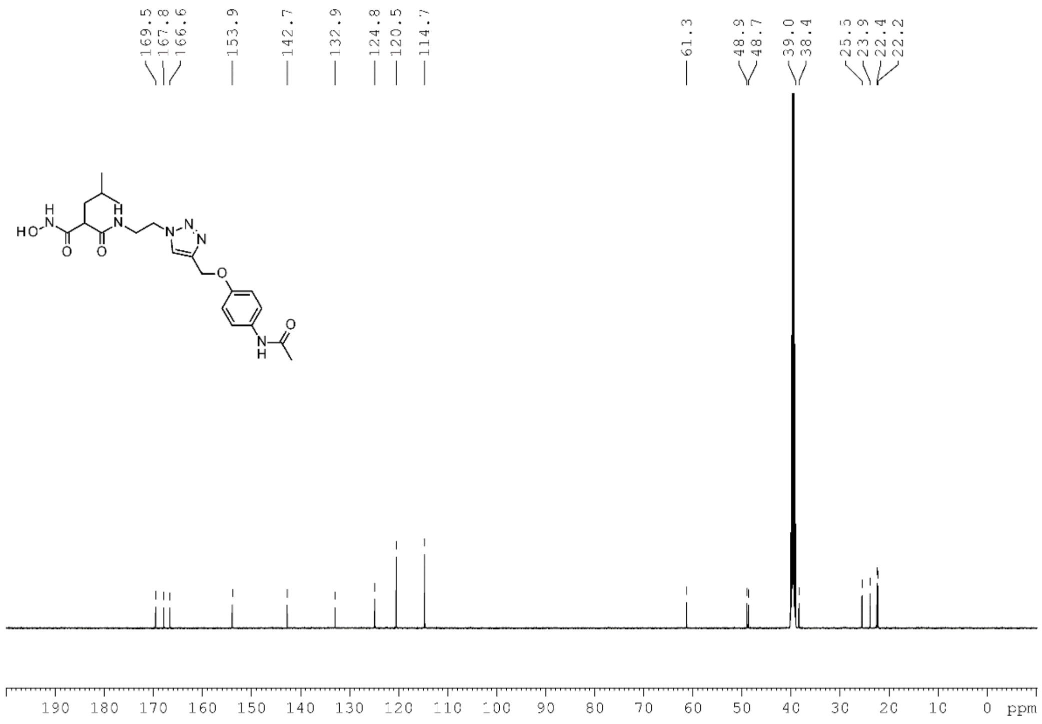

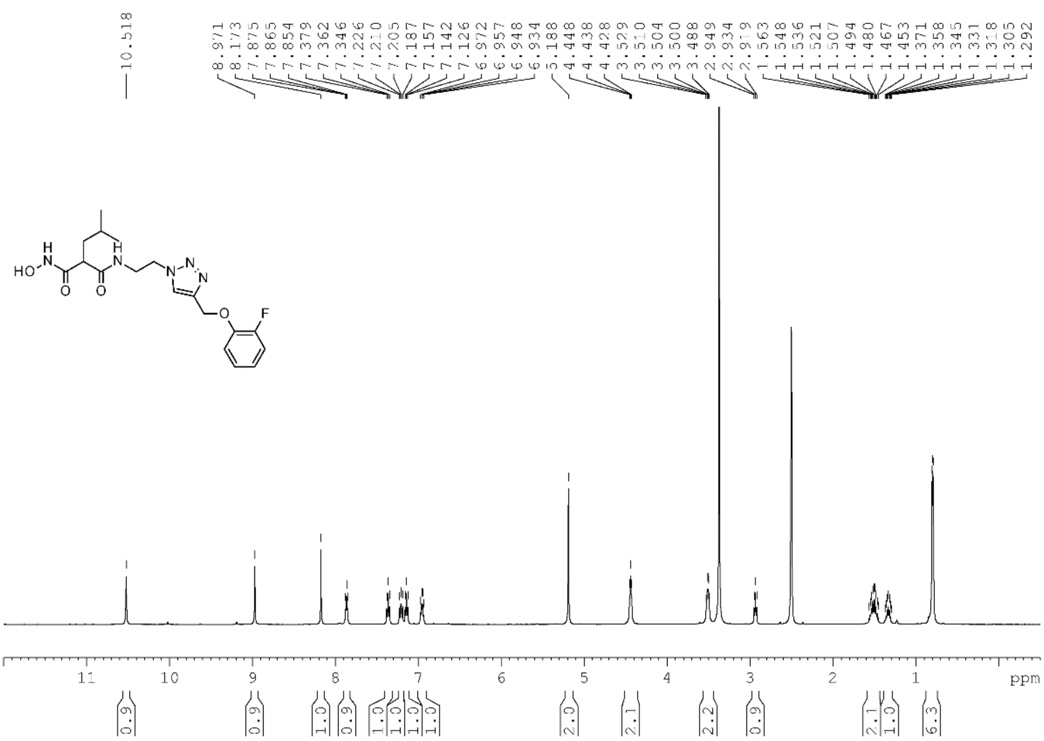
10.518
8.971
8.173
7.875
7.865
7.854
7.379
7.362
7.346
7.226
7.210
7.205
7.187
7.157
7.142
7.126
6.972
6.957
6.948
6.934
5.188
4.448
4.438
4.428
3.529
3.510
3.504
3.500
3.488
2.949
2.934
2.919
1.563
1.548
1.536
1.521
1.507
1.494
1.480
1.467
1.453
1.371
1.358
1.345
1.331
1.318
1.305
1.292
HO
N
H
O
F
11 10 9 8 7 6 5 4 3 2 1 ppm
0.9
0.9
1.0
0.9
1.0
1.0
1.0
1.0
2.0
2.1
2.2
0.9
2.1
1.0
6.3

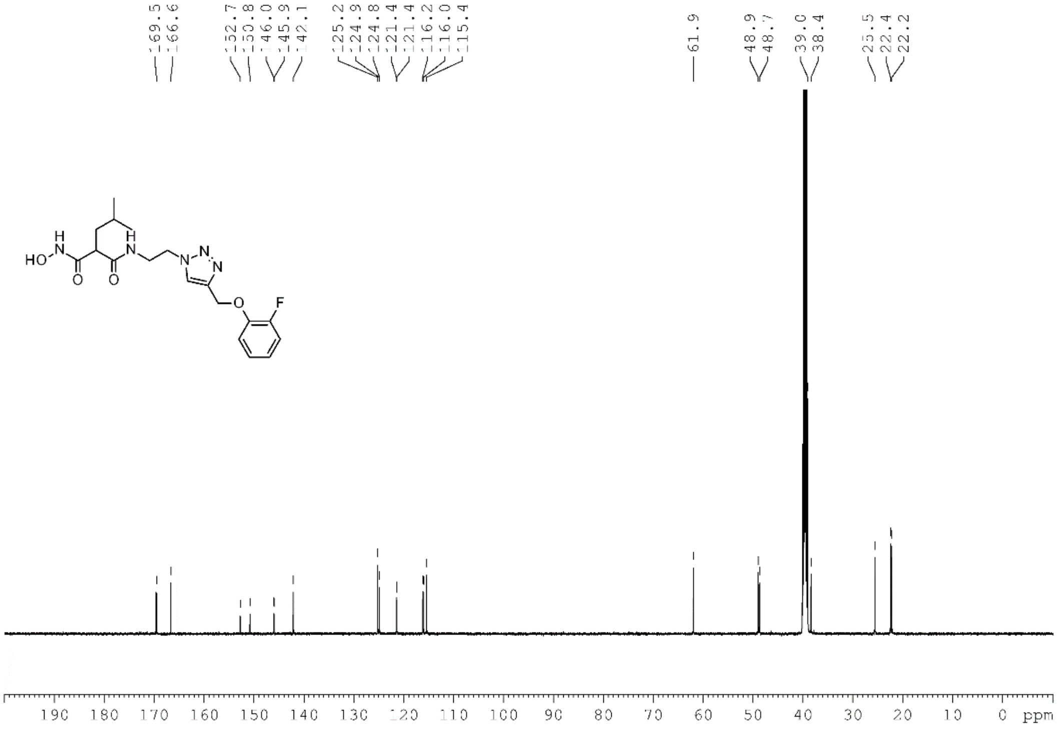
169.5
166.6
152.7
150.8
146.0
145.9
142.1
125.2
124.9
124.8
121.4
121.4
116.2
116.0
115.4
61.9
48.9
48.7
39.0
38.4
25.5
22.4
22.2
HO
N
H
O
F
190 180 170 160 150 140 130 120 110 100 90 80 70 60 50 40 30 20 10 0 ppm

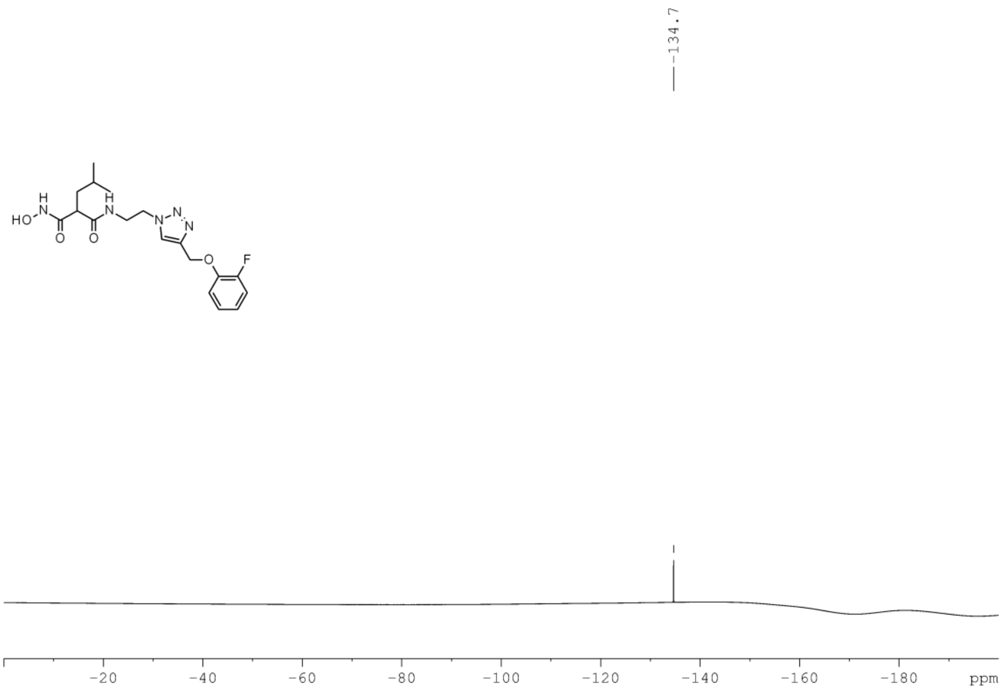
-134.7
-20
-40
-60
-80
-100
-120
-140
-160
-180
ppm
HO
N
H
O
F

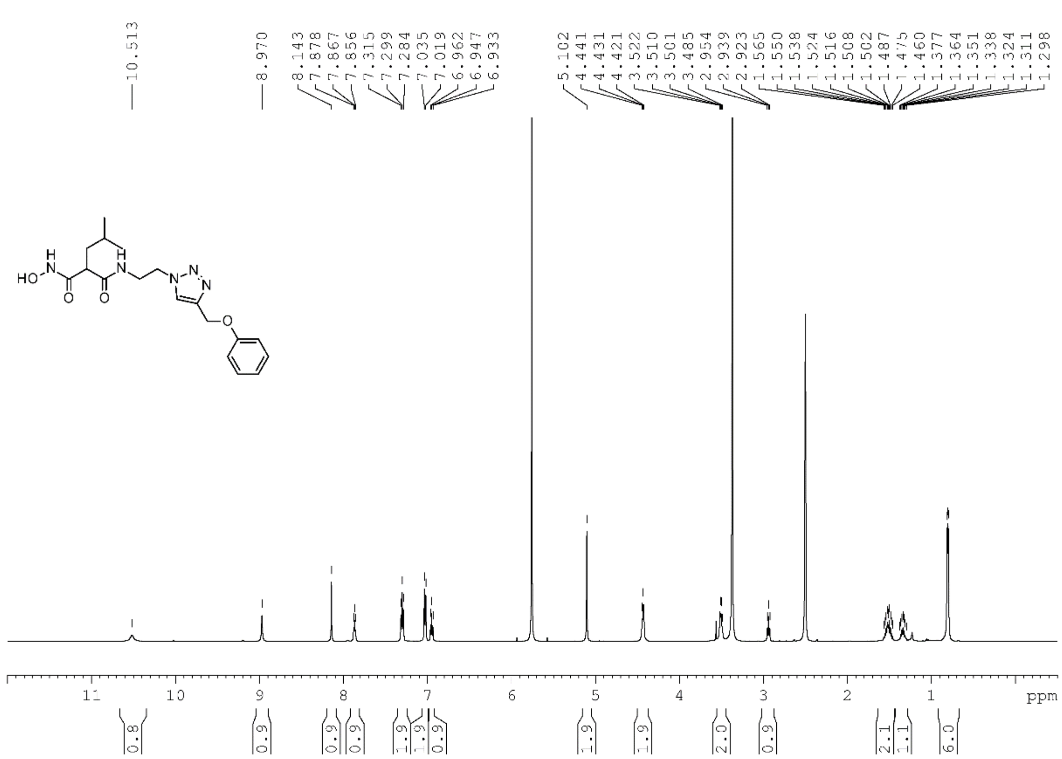
10.513
8.970
8.143
7.878
7.867
7.856
7.315
7.299
7.284
7.035
7.019
6.962
6.947
6.933
5.102
4.441
4.431
4.421
3.522
3.510
3.501
3.485
2.954
2.939
2.923
1.565
1.550
1.538
1.524
1.516
1.508
1.502
1.487
1.475
1.460
1.377
1.364
1.351
1.338
1.324
1.311
1.298
11
10
9
8
7
6
5
4
3
2
1
ppm
0.8
0.9
0.9
0.9
0.9
2.0
0.9
6.0
HO
N
H
O

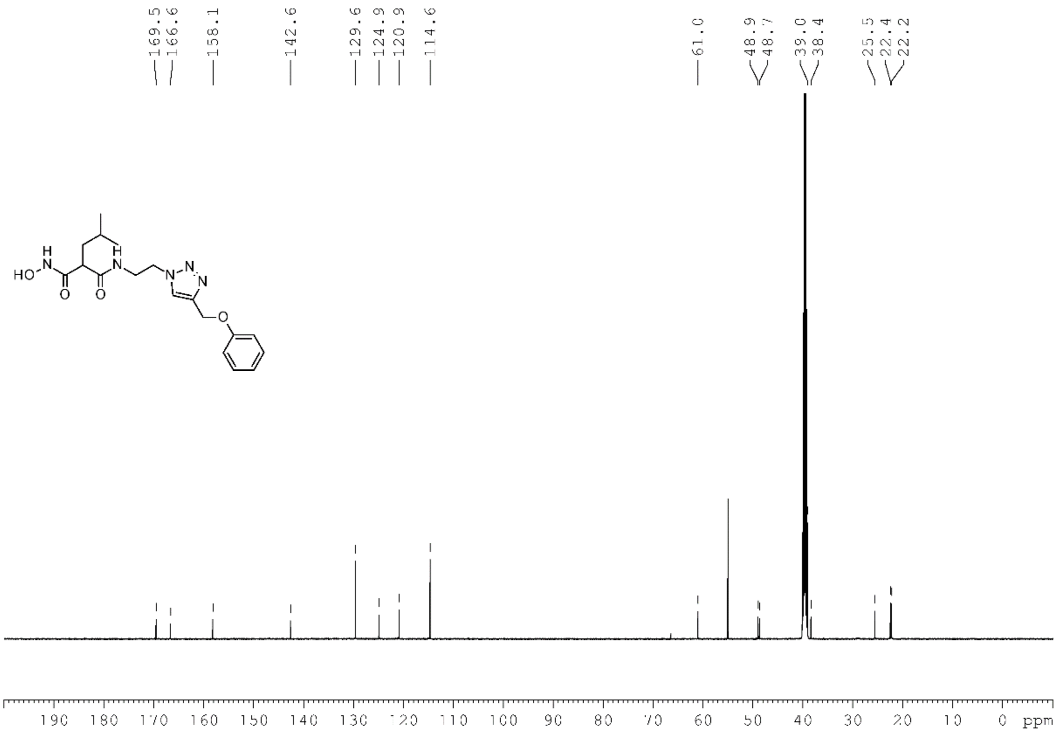
169.5
166.6
158.1
142.6
129.6
124.9
120.9
114.6
61.0
48.9
48.7
39.0
38.4
25.5
22.4
22.2
190 180 170 160 150 140 130 120 110 100 90 80 70 60 50 40 30 20 10 0 ppm

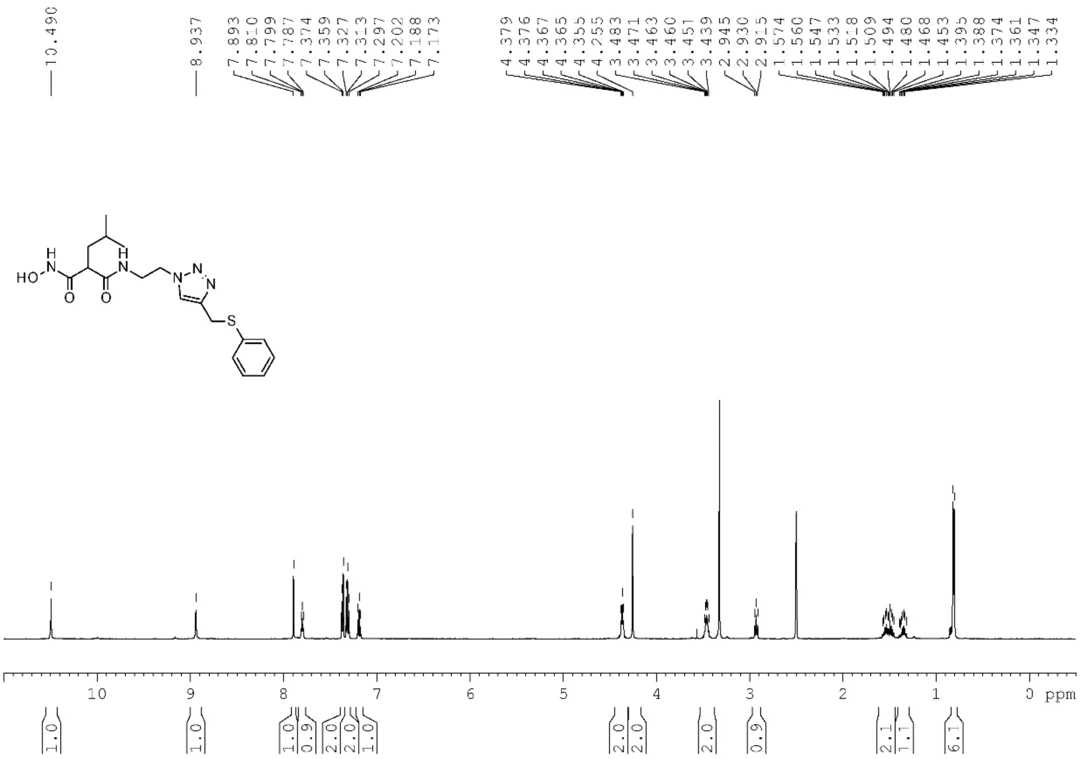
10.490
8.937
7.893
7.810
7.799
7.787
7.374
7.359
7.327
7.313
7.297
7.202
7.188
7.173
4.379
4.376
4.367
4.365
4.355
4.255
3.483
3.471
3.463
3.460
3.451
3.439
2.945
2.930
2.915
1.574
1.560
1.547
1.533
1.518
1.509
1.494
1.480
1.468
1.453
1.395
1.388
1.374
1.361
1.347
1.334
10 9 8 7 6 5 4 3 2 1 0 ppm
1.0
1.0
1.0
0.9
2.0
2.0
1.0
2.0
2.0
2.0
0.9
2.1
1.1
6.1

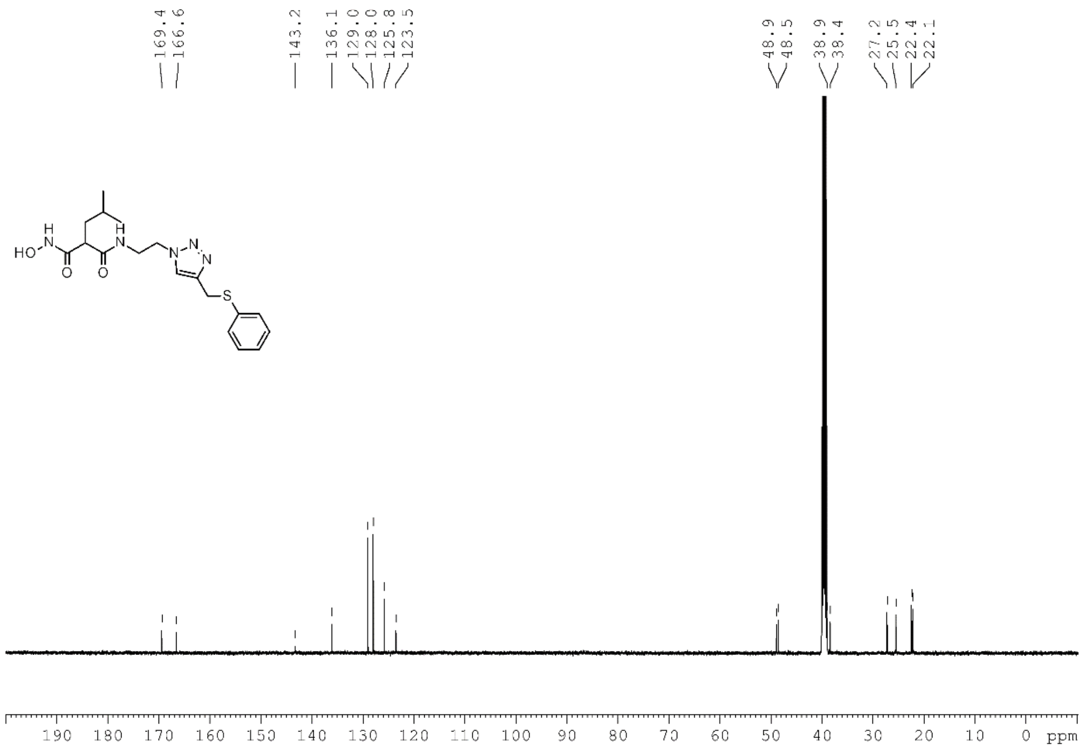
169.4
166.6
143.2
136.1
129.0
128.0
125.8
123.5
48.9
48.5
38.9
38.4
27.2
25.5
22.4
22.1
190 180 170 160 150 140 130 120 110 100 90 80 70 60 50 40 30 20 10 0 ppm

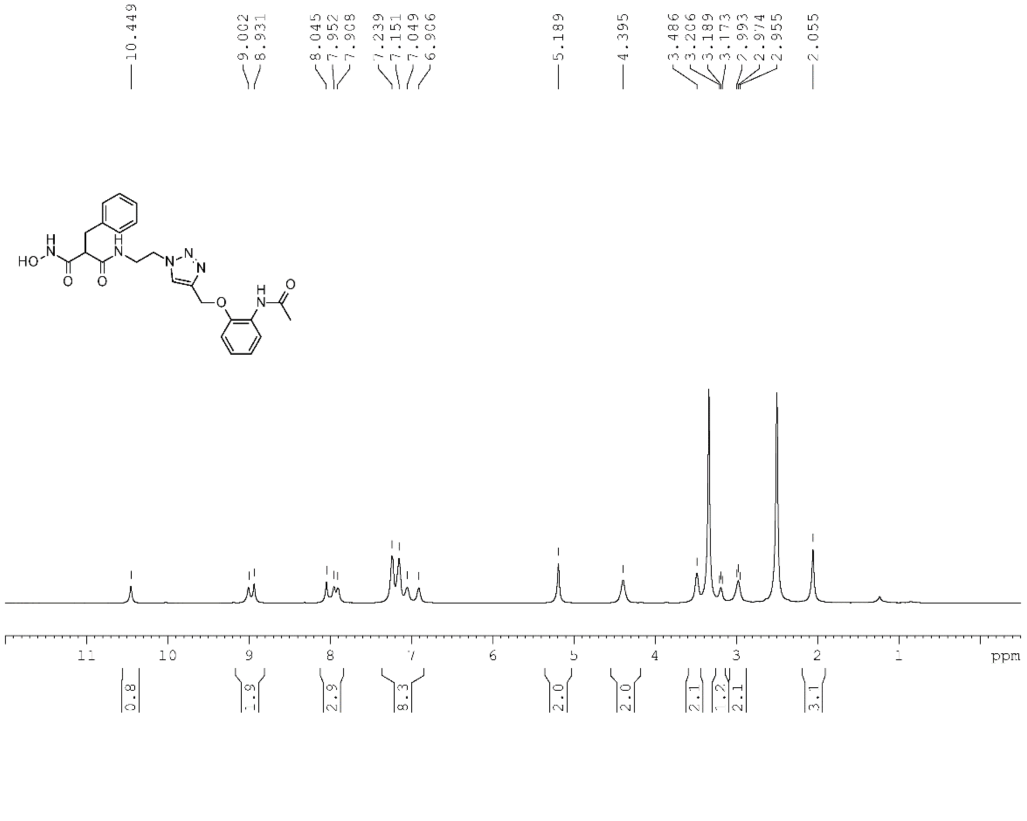
10.449
9.002
8.931
8.045
7.952
7.908
7.239
7.151
7.049
6.906
5.189
4.395
3.486
3.206
3.189
3.173
2.993
2.974
2.955
2.055
11 10 9 8 7 6 5 4 3 2 1 ppm

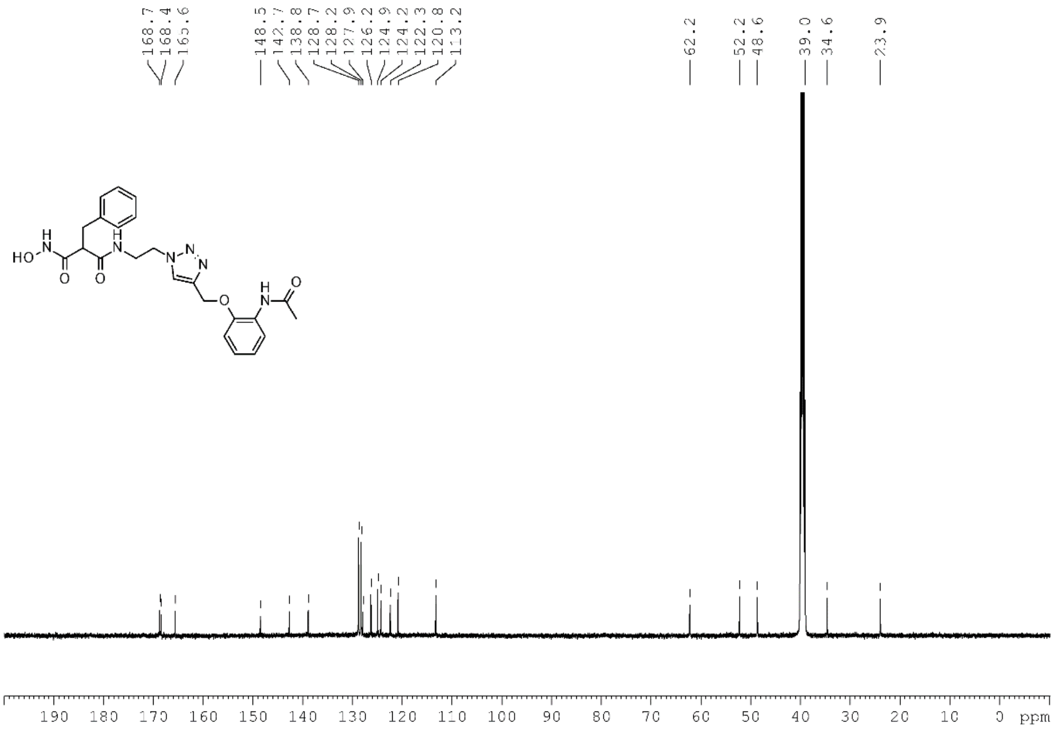

168.7
168.4
165.6
148.5
142.7
138.8
128.7
128.2
127.9
126.2
124.9
124.2
122.3
120.8
113.2
62.2
52.2
48.6
39.0
34.6
23.9
HO
N
H
O
O
N
H
N
N
N
O
N
H
O
190 180 170 160 150 140 130 120 110 100 90 80 70 60 50 40 30 20 10 0 ppm

LC-MS Spectra

Diphosphonate compounds

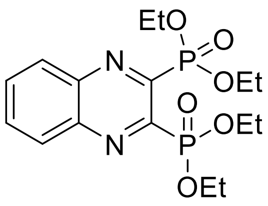

Exact Mass: 402.11096

Compound **7c**

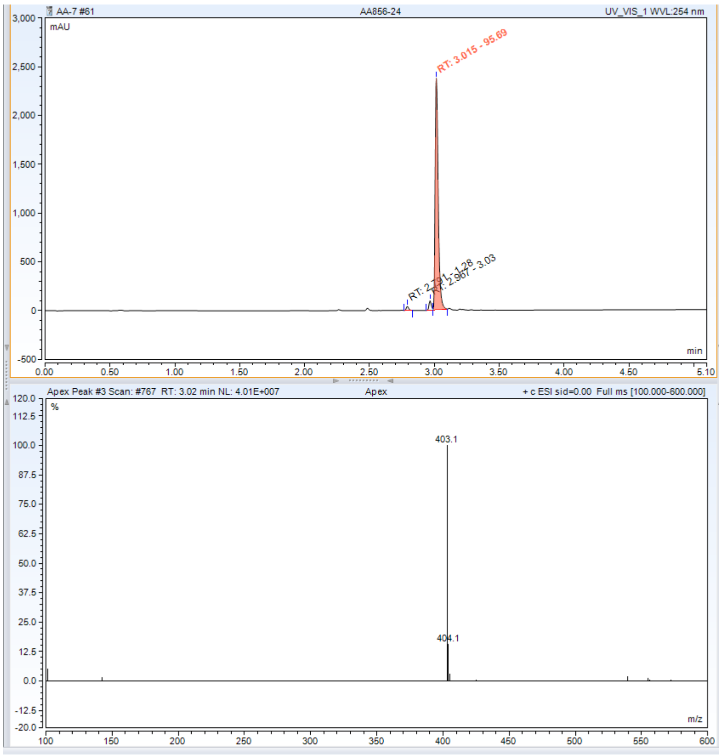

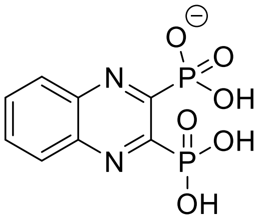

Exact Mass: 288.97848

Compound **7**

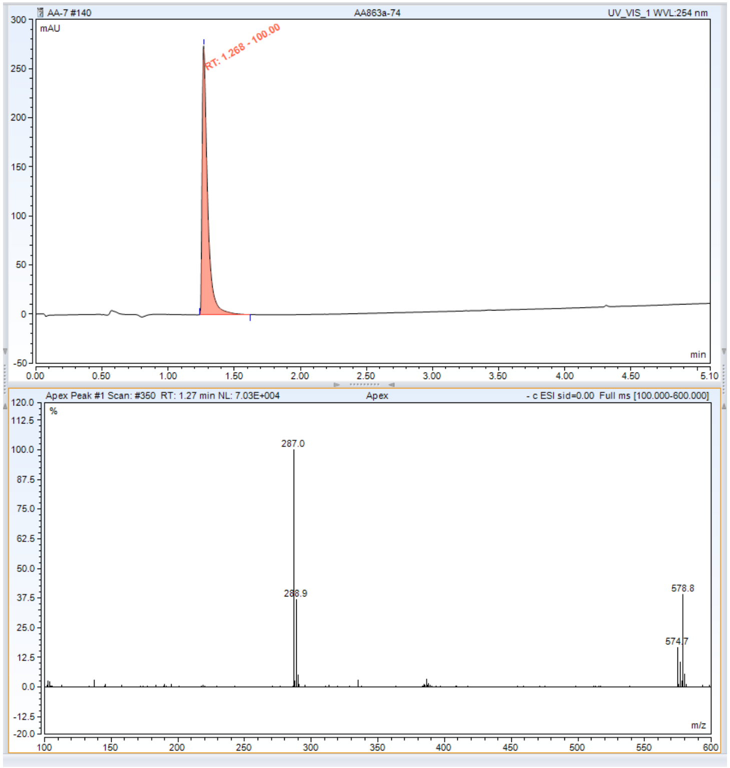

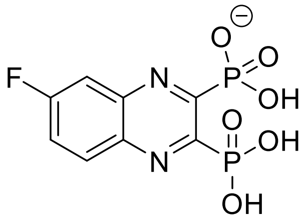

Exact Mass: 306.96906

Compound **8**

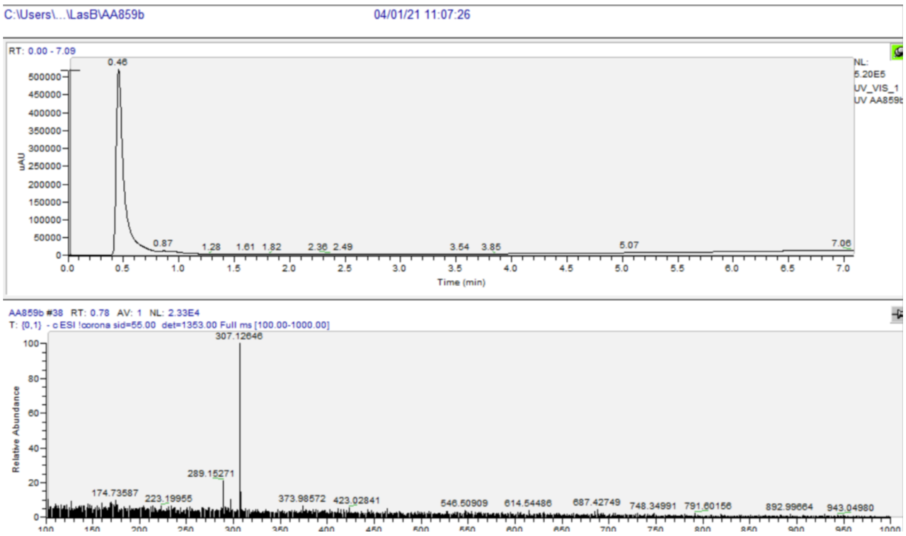

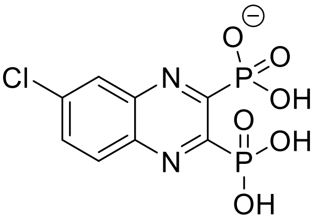

Exact Mass: 322.93951

Compound **9**

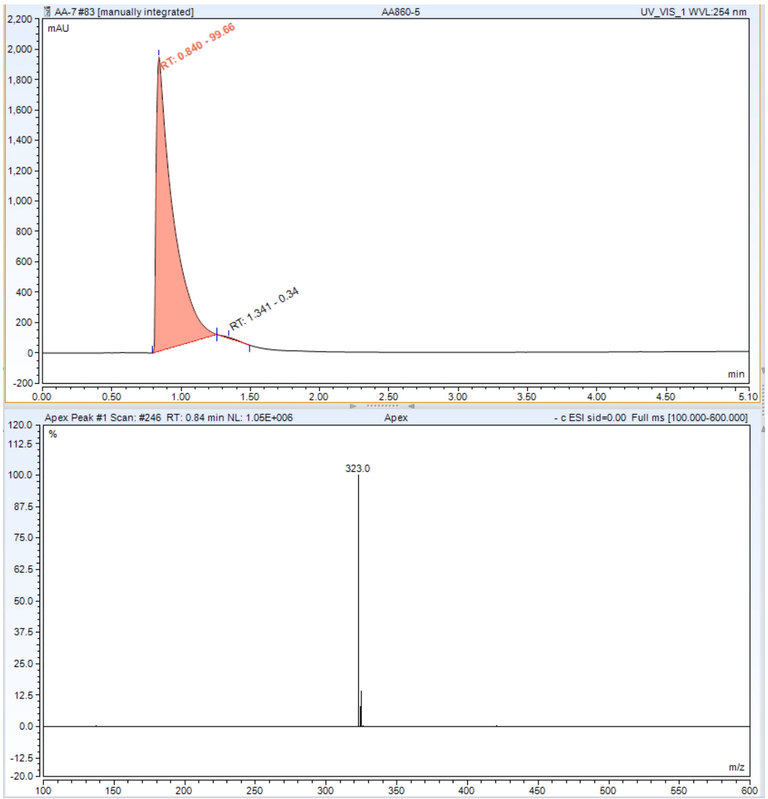

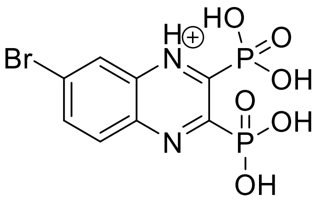

Exact Mass: 368.90355

Compound **10**

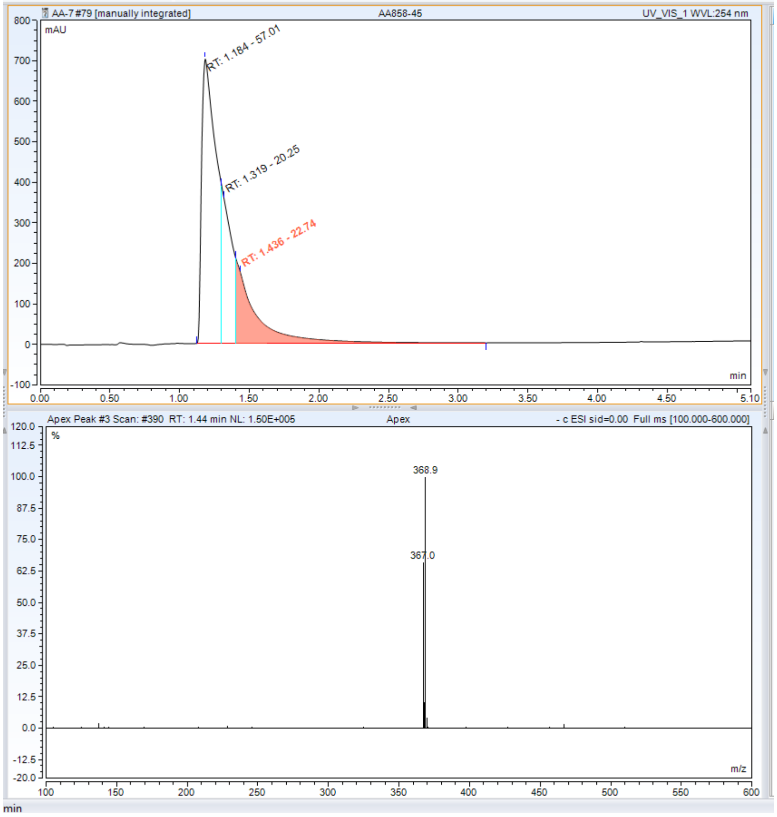

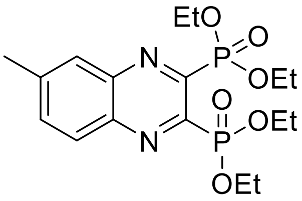

Exact Mass: 416.12661

Compound **11c**

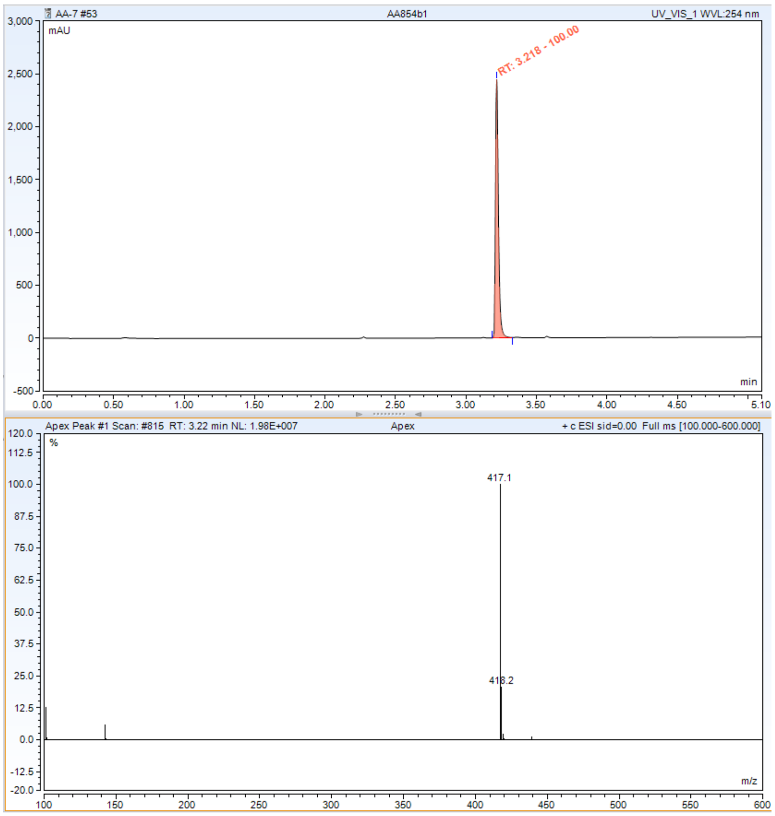

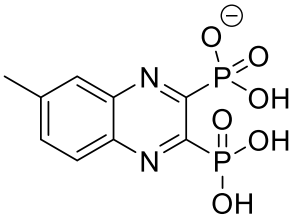

Exact Mass: 302.99413

Compound **11**

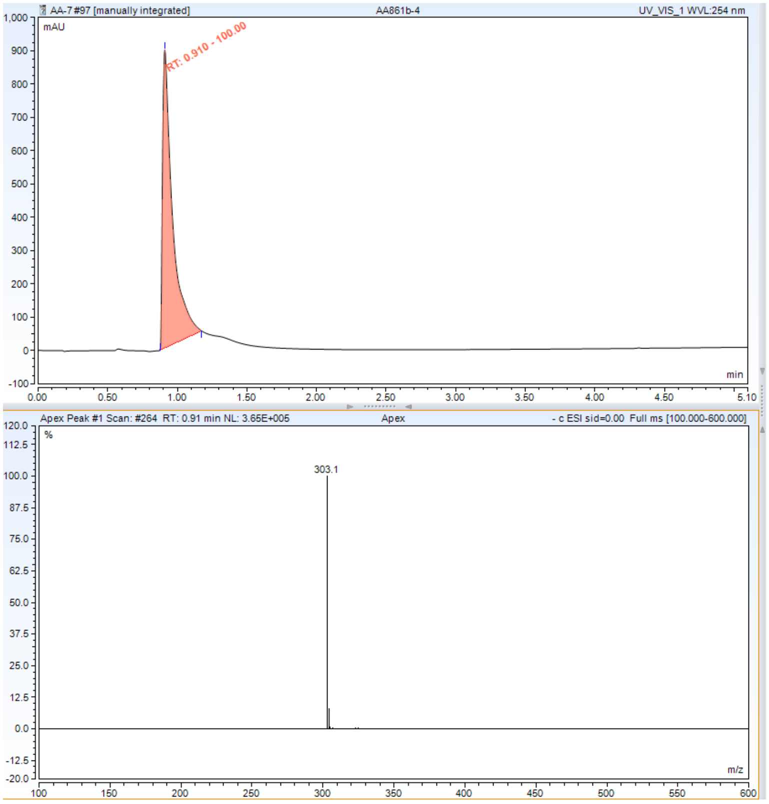

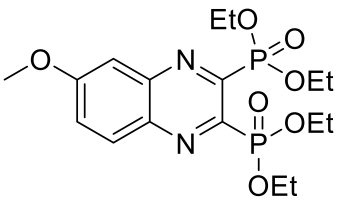

Exact Mass: 432.12152

Compound **12c**

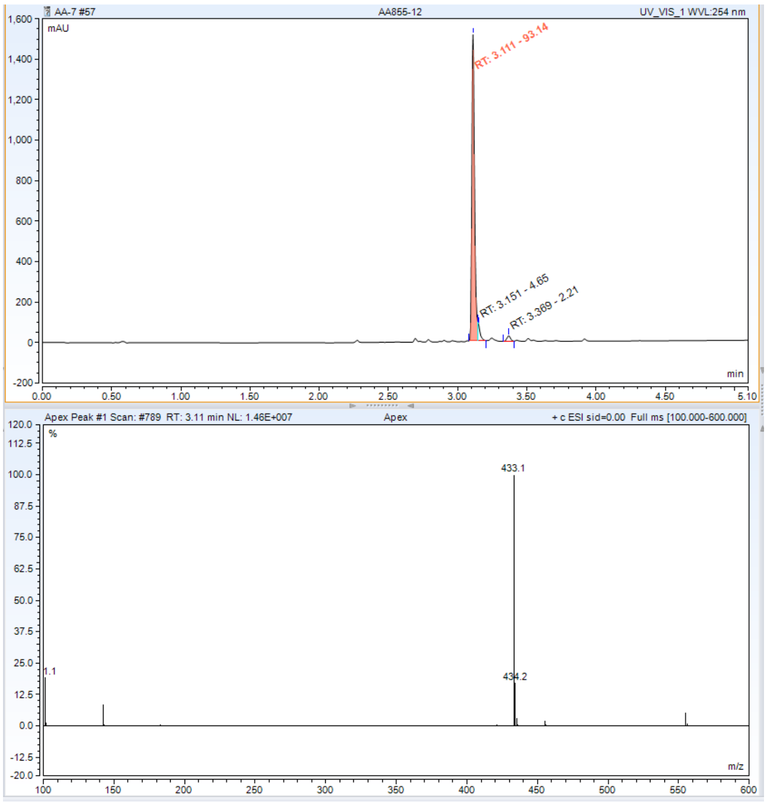

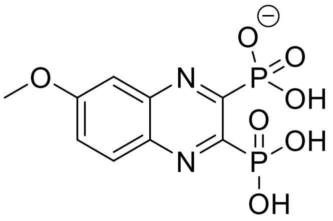

Exact Mass: 318,99

Compound **12**

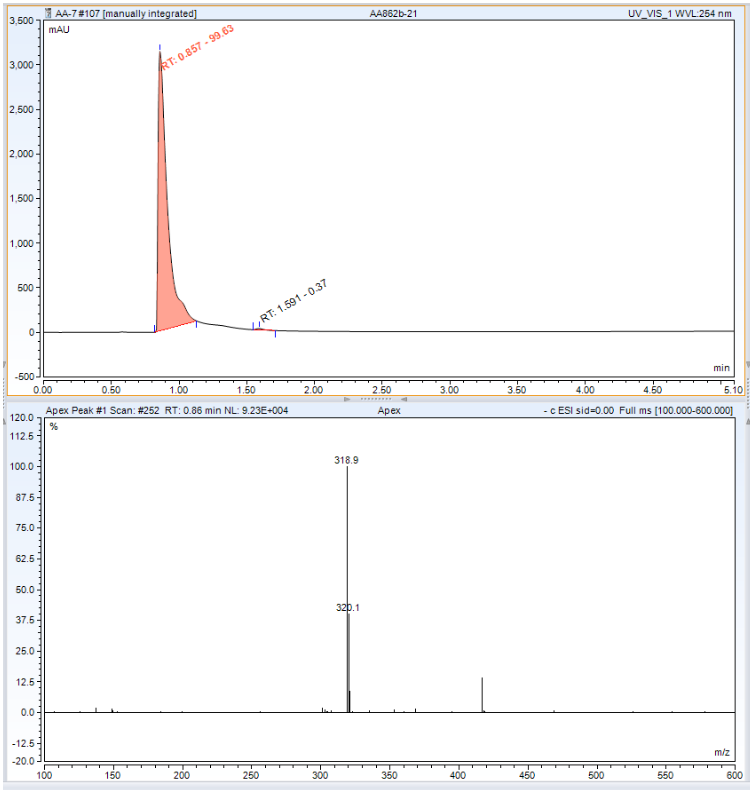

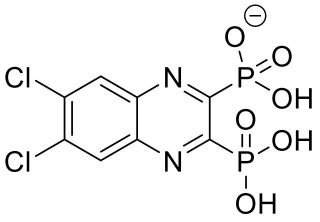

Exact Mass: 356.90054

Compound **13**

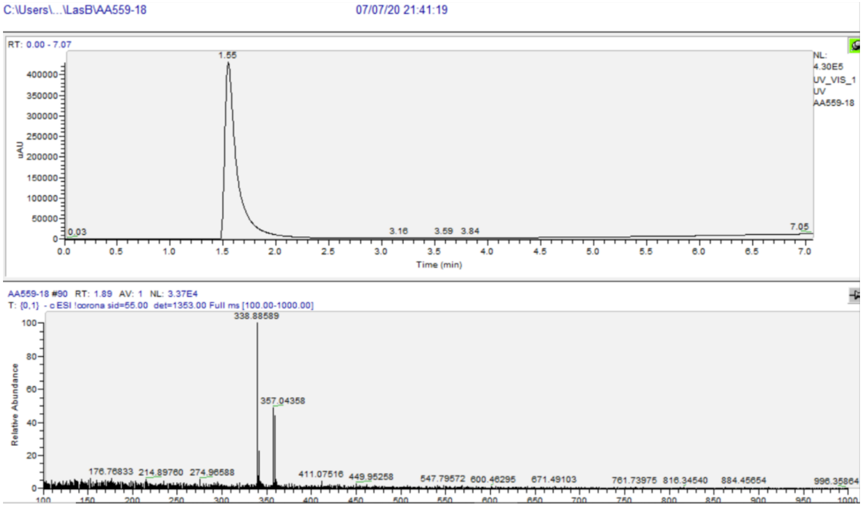

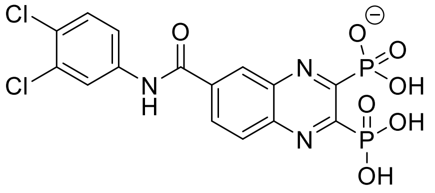

Exact Mass: 475.93765

Compound **14**

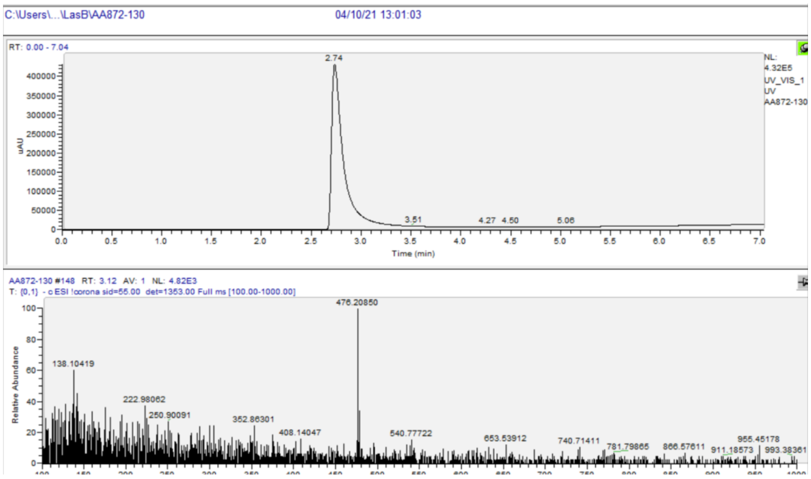

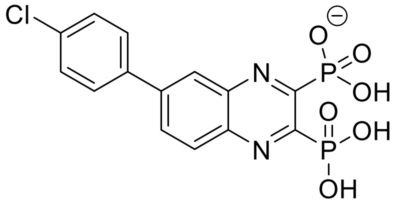

Exact Mass: 398.97081

Compound **15**

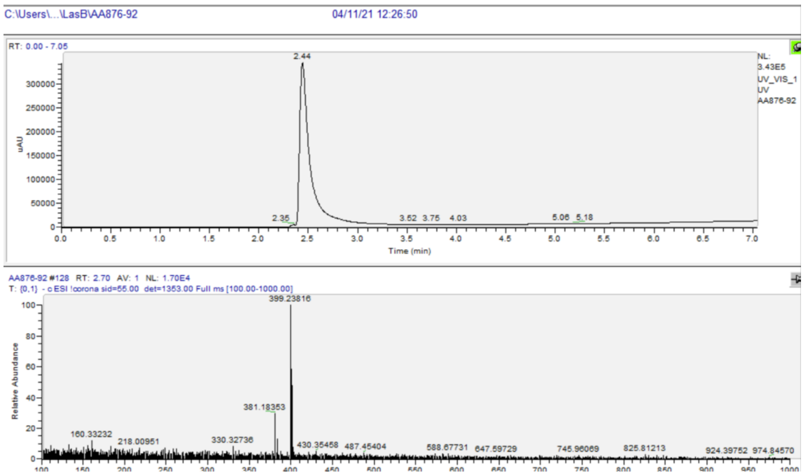

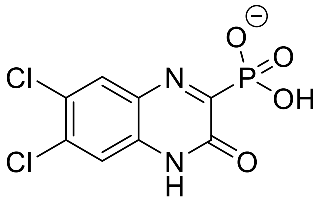

Exact Mass: 292.92912

Compound **16**

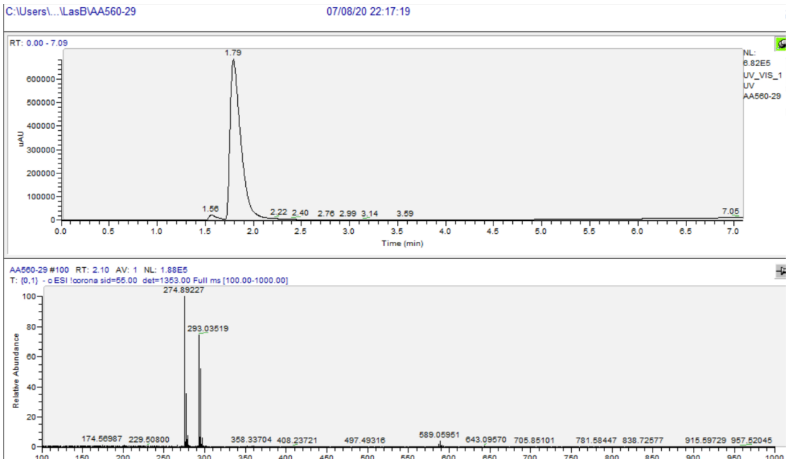

Hydroxamate compounds

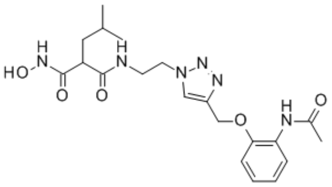

Compound **27**
Exact mass: 432.2121

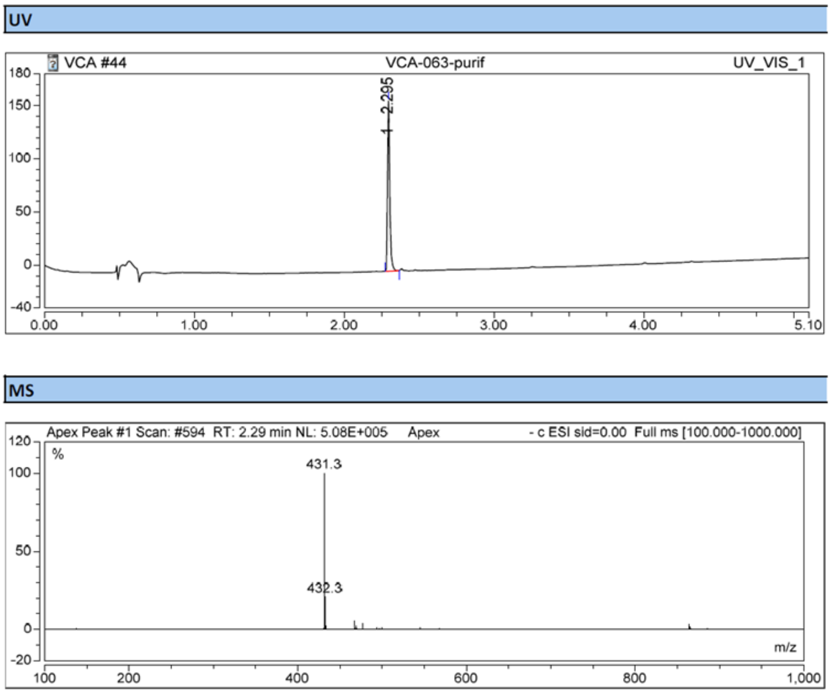

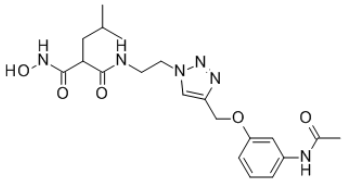

Compound **28**
Exact mass: 432.2121

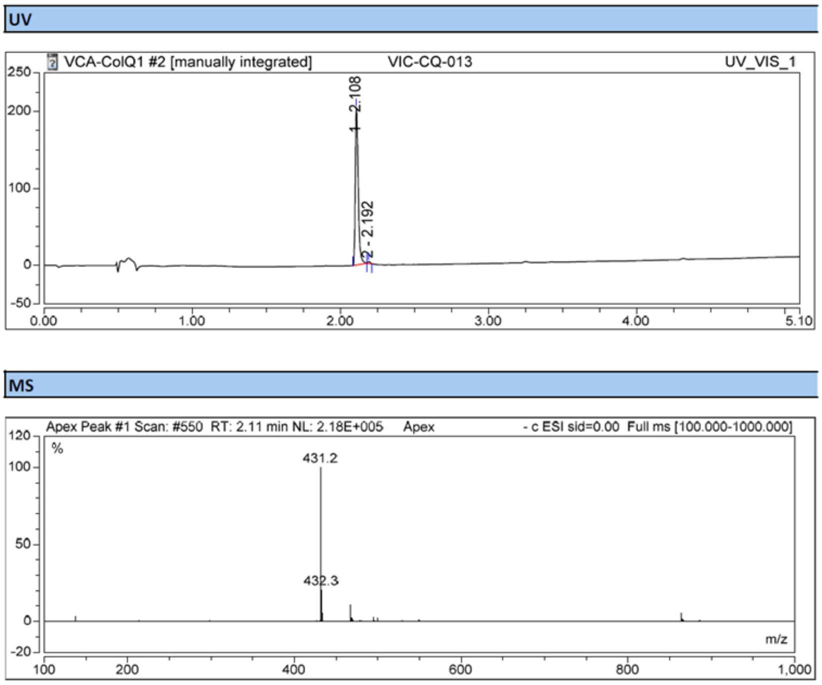

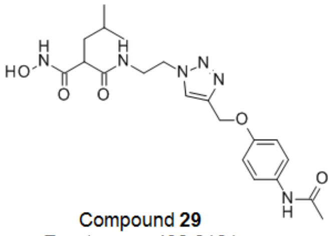

Compound **29**
Exact mass: 432.2121

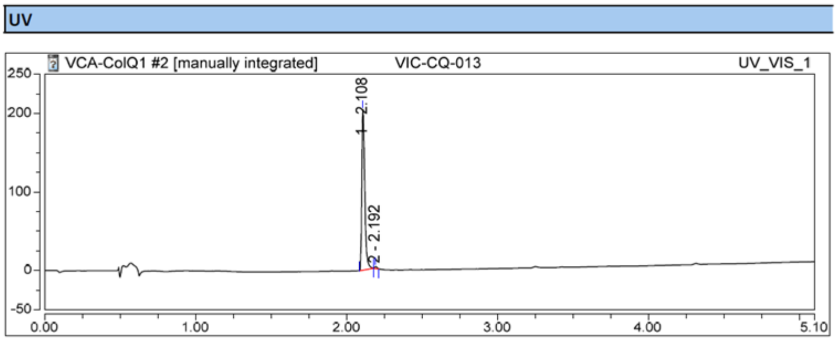

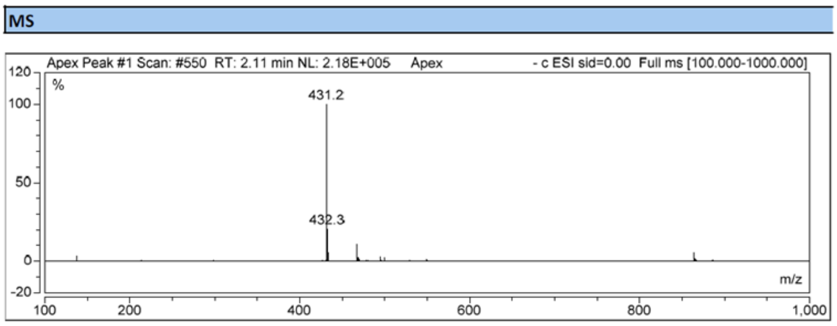

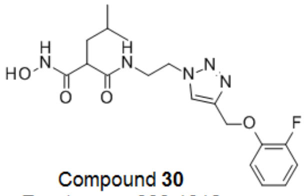

Compound **30**
Exact mass: 393.1812

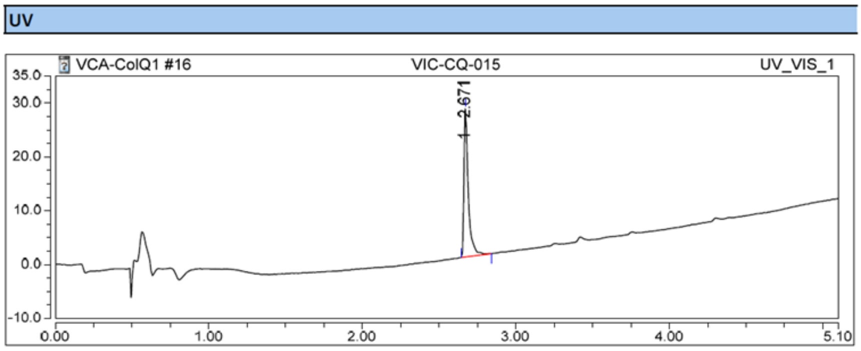

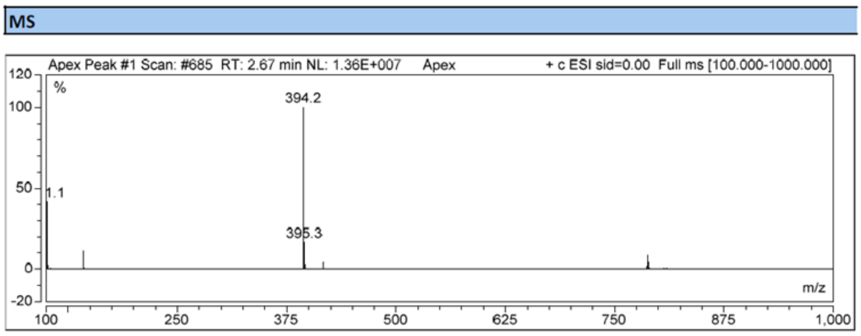

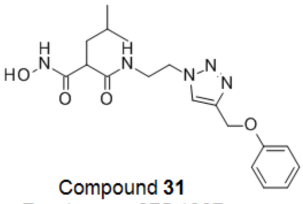

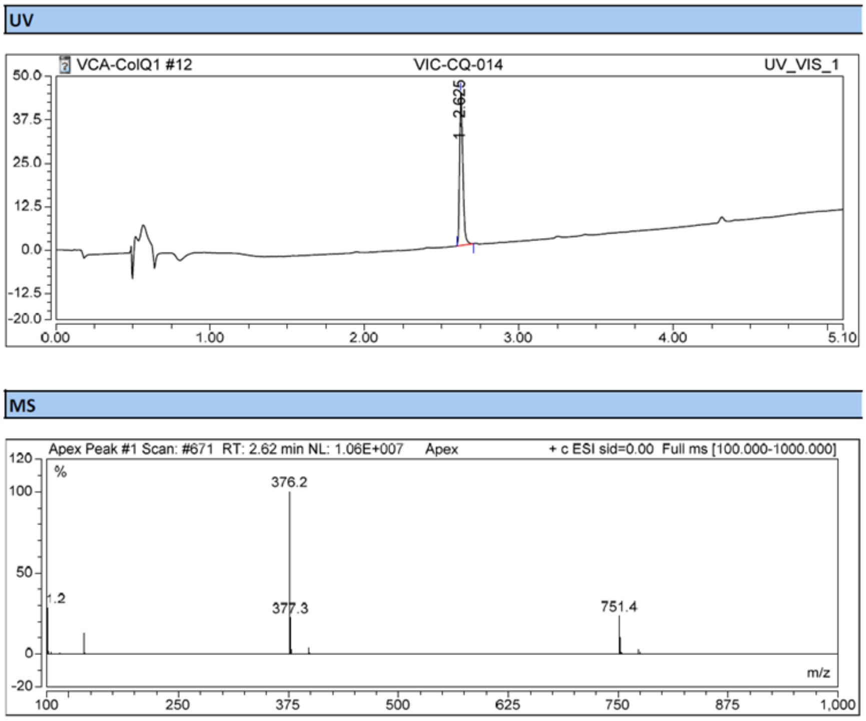

Compound **31**
Exact mass: 375.1907

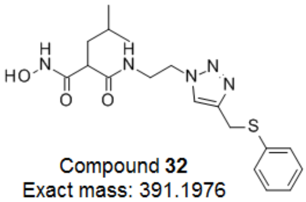

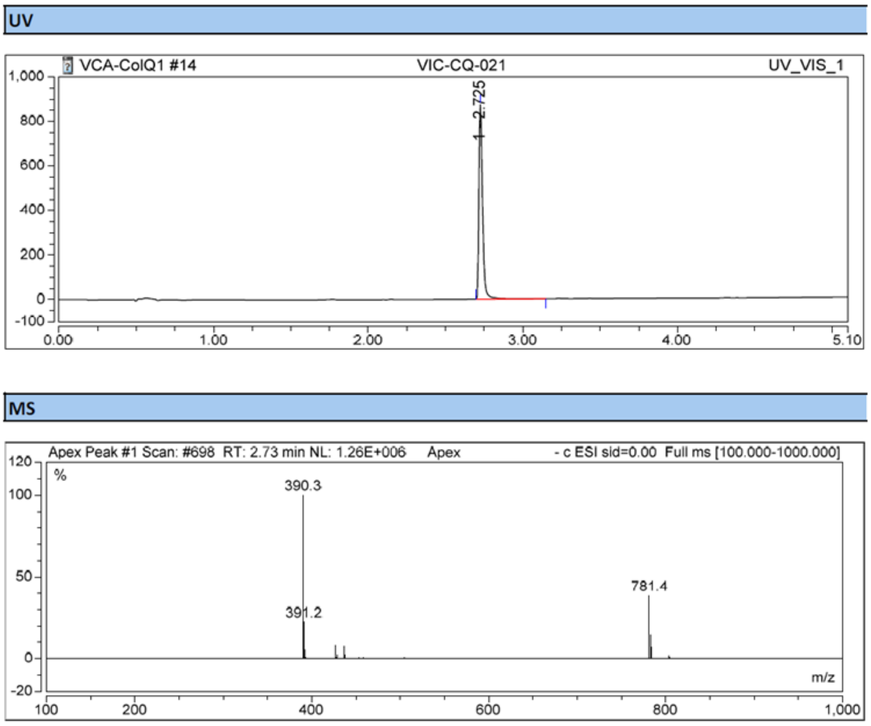

Compound **32**
Exact mass: 391.1976

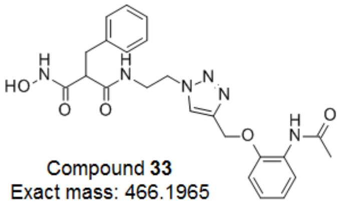

Compound **33**
Exact mass: 466.1965

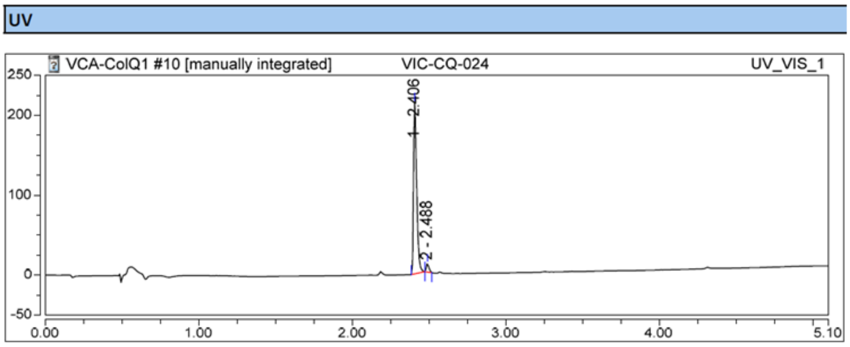

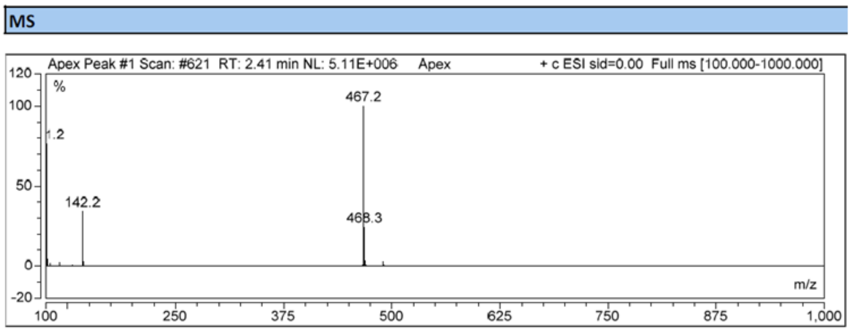

Supplement: Supplementary file 2 — jm2c00785_si_002.pdf [file jm2c00785_si_002.pdf]
